# Imaging biomarkers of dementia: recommended visual rating scales with teaching cases

**DOI:** 10.1007/s13244-016-0521-6

**Published:** 2016-12-21

**Authors:** Lars-Olof Wahlund, Eric Westman, Danielle van Westen, Anders Wallin, Sara Shams, Lena Cavallin, Elna-Marie Larsson, From the Imaging Cognitive Impairment Network (ICINET)

**Affiliations:** 1 Division of Clinical Geriatrics, Department of Neurobiology, Care Sciences, and Society, Karolinska Institutet, Stockholm, Sweden; 2 Diagnostic Radiology, Clinical Sciences, Lund University, Lund, Sweden; 3 Imaging and Function, Skåne University Hospital, Lund, Sweden; 4 Institute of Neuroscience and Physiology, Sahlgrenska Academy at University of Gothenburg, Gothenburg, Sweden; 5 Department of Clinical Science, Intervention, and Technology, Division of Medical Imaging and Technology, Karolinska Institutet, Stockholm, Sweden; 6 Department of Radiology, Karolinska University Hospital, SE-14186 Stockholm, Sweden; 7 Department of Surgical Sciences, Radiology, Uppsala University, Uppsala, Sweden

**Keywords:** Dementia, Imaging, Alzheimer’s disease, MRI, CT

## Abstract

**Abstract:**

The diagnostic work up of dementia may benefit from structured reporting of CT and/or MRI and the use of standardised visual rating scales. We advocate a more widespread use of standardised scales as part of the workflow in clinical and research evaluation of dementia. We propose routine clinical use of rating scales for medial temporal atrophy (MTA), global cortical atrophy (GCA) and white matter hyperintensities (WMH). These scales can be used for evaluation of both CT and MRI and are efficient in routine imaging assessment in dementia, and may improve the accuracy of diagnosis. Our review provides detailed imaging examples of rating increments in each of these scales and a separate teaching file. The radiologist should relate visual ratings to the clinical assessment and other biomarkers to assist the clinician in the diagnostic decision.

***Teaching points*:**

• *Clinical dementia diagnostics would benefit from structured radiological reporting.*

• *Standardised rating scales should be used in dementia assessment.*

• *It is important to relate imaging findings to the clinically suspected diagnosis.*

**Electronic supplementary material:**

The online version of this article (doi:10.1007/s13244-016-0521-6) contains supplementary material, which is available to authorized users.

## Introduction

The prevalence of dementia is increasing due to longer life expectancy, including a large increase of populations aged 80-years and older. A thorough investigation of suspected dementia and pre-dementia stages is of high importance for early diagnosis, caretaking and, if possible, treatment. Brain imaging is included among the basic investigations in the work-up of dementia in many countries. Knowledge on dementia and particularly Alzheimer’s disease has increased significantly in recent years, especially with regard to imaging methods and their impact on differential diagnosis. Nevertheless, this knowledge has not been fully implemented in clinical radiological routine work, most likely due to lack of communication between academia and clinical practice. In this paper,we describe how changes characteristic of common dementia disorders can be assessed in a structured way using computed tomography (CT) and magnetic resonance imaging (MRI). Established visual rating scores offer a practical, fast and inexpensive means of improving the diagnostic accuracy [1].Our review is based on a collaboration between Karolinska Institutet, Uppsala University, Lund University, Gothenburg University and other university hospitals in Sweden, Norway and internationally as part of the imaging cognitive impairment network (ICINET). ICINET was formed initially to standardise imaging dementia assessment, and recommend its use in clinical practice [2].

In normal ageing, cognitive functions may be reduced to varying degree, with pronounced reduction in some cognitive domains usually due to disease processeses leading to disability. Criteria for dementia are met if the disability becomes severe, affecting cognitive domains [3]. One of the most common causes of dementia is Alzheimer’s disease (60-70 %). Hallmarks of the neurodegenerative process are abnormal production and/or reduced clearance of the beta amyloid (Aβ) protein which in its abnormal form is aggregated in so-called plaques [4, 5]. Phosphorylation of tau protein leading to degradation and destruction of cellular support structures is another important sign [6]. These changes in combination with other factors are most probably causing the extensive cell destruction that develops during the course of the disease that in its turn leads to cerebral atrophy of the brain, usually starting in the medial temporal lobes [7–10]. Vascular dementia is another cause of cognitive impairment, considered to be the second most common form of dementia after Alzheimer’s disease and is observed in about 30 % of all dementia patients. Recently, an overlap between Alzheimer’s disease and vascular dementia with small vessel disease has been reported as a possible contributor and cause of dementia [11–13]. Cerebral amyloid angiopathy and hypertensive arteriopathy constitute the two most common small vessel diseases and are thought to be important parts of the dementia disease process [11, 12]. Other neurodegenerative diseases with cognitive impairment are frontotemporal dementia (2 % of all dementias [14]) and Lewy body dementia (4.2 % of all diagnosed dementias [15]). Cognitive impairment may also occur in, for example, depression and as a result of a brain tumour, cerebral haemorrhage and stroke.

The diagnosis of dementia is based on clinical symptoms as well as evidence of amyloid and tau pathology in the case of Alzheimer’s disease [16]. MRI of the brain significantly increases the confidence of a dementia diagnosis. Traditionally, CT and MRI were used to rule out disease that may lead to cognitive impairment, such as for instance intracranial tumour and multiple sclerosis. However, in the modern clinical work-up of dementia, CT and MRI are cornerstones due to their ability to detect patterns of atrophy that may be specific for a neurodegenerative disease, for example medial temporal lobe atrophy constitutes an early sign of Alzheimer’s disease [17, 18]. In addition, imaging substrates of cerebrovascular disease are visualized and often the diagnosis of small vessel disease is added after CT and MRI. The European leukoariosis and disability (LADIS) studies have shown evidence that white matter hyperintensities increase the risk of cognitive decline (11).

**Fig. 1:**
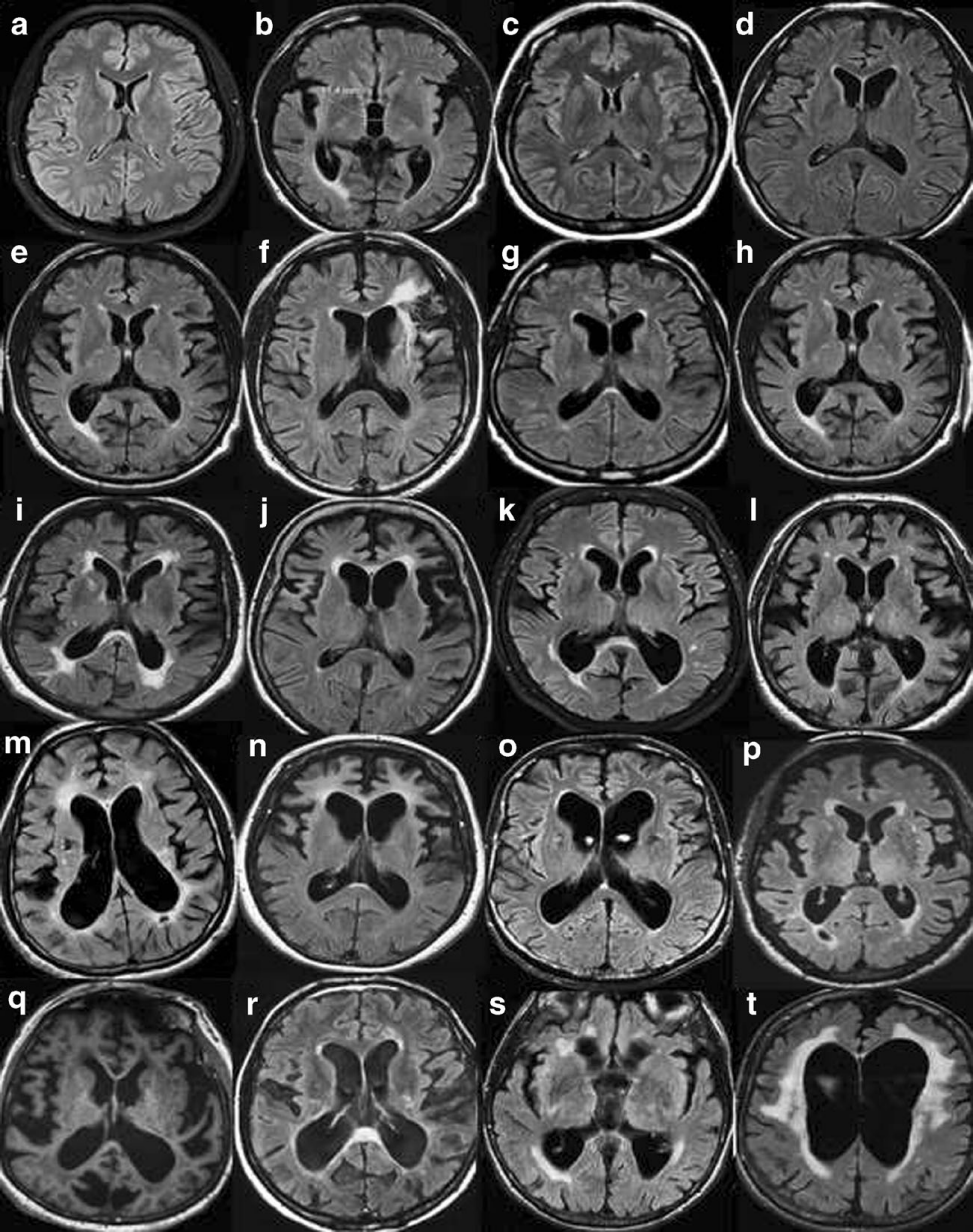

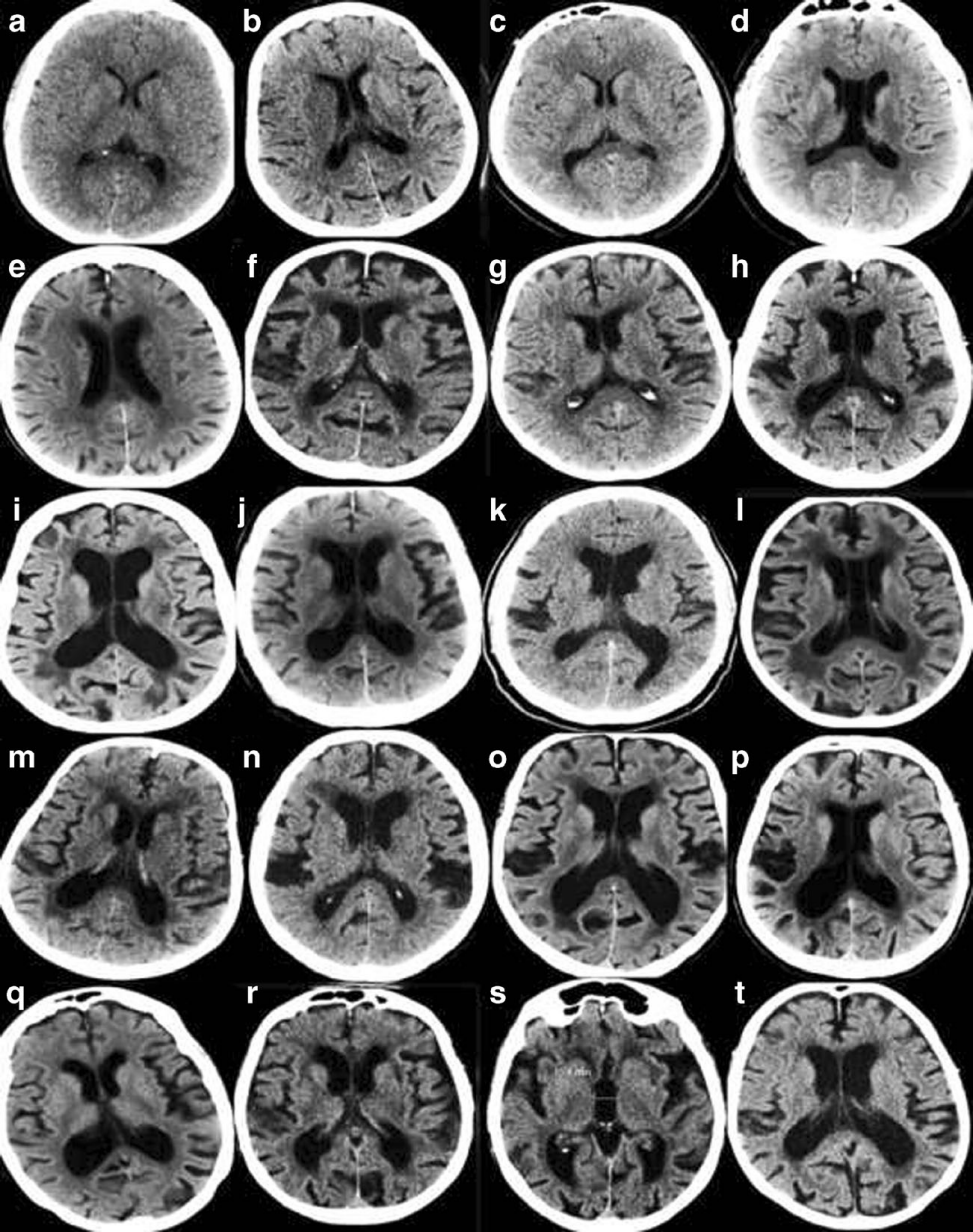
**A**. Widening of the ventricles on MRI. Interpretation key. Numbers equal the corresponding image in the supplementary PDF file. Appropriate wording for widening of the ventricles is: no, mild, moderate or severe widening. **a/1)** There are no visual rating scales for the size of the ventricles. Compare to the widening of a 25-year-old healthy individual. **b/2)** A third ventricle with a diameter of >10 mm is pathological at any age. **c/6)** No widening. **d/16)** No widening. **e/17)** In between no widening and mild widening. The third ventricle is wider than usual. **f/20)** Mild widening. Note the slightly wide frontal horn on the left side due to the frontal infarction. **g/7)** Mild widening **h/9)** Mild widening. **i/15)** Approaching mild widening. **j/12)** Mild bifrontal widening and no widening of posterior horns. **k/11)** Mild bifrontal widening and a left posterior horn with moderate widening. **l/3)** In this case: mild, with a moderate widening of the posterior horns. **m/5)** Mild widening of the frontal horns and moderate of posterior horns. **n/8)** Mild widening, with a moderate widening of frontal horns. **o/19)** In between mild and moderate widening but closer to moderate. **p/13)** Mild central widening, with the right posterior horn a bit wider, but not yet moderate. **q/10)** Mild widening of frontal horns and moderate of the posterior horns, especially the left posterior horn. **r/14)** Moderate widening, especially of posterior horns. **s/18)** Severe dilatation of third ventricle and moderate widening of the posterior horns. **t/4)** Severe widening of the ventricles, especially of the frontal horns. **B.** Widening of the ventricles on CT. Interpretation key. Numbers equal the corresponding image in the supplementary PDF file. **a/1)** No widening. The normative key. **b/7)** No widening, right deep MCA infarction is seen in the image. **c/3)** No widening **d/19)** No widening but wide cavum septi pellucidi et vergae. **e/11)** Nearly no widening. **f/14)** Between no widening and mild. **g/6)** Normal to mild widening. **h/9)** Mild widening. **i/12)** Mild widening. **j/13)** Mild widening. **k/18)** Mild widening of ventricles. **l/17)** Mild widening of lateral ventricles. Cavum septi pellucidi et verge makes ventricles look wider. **m/15)** Mild widening of the posterior horns without exceeding into moderate widening. **n/20)** Mild widening of frontal horns and a mild to moderate widening of the posterior horns. **o/10)** Mild widening with a moderate widening of the left posterior horn. **p/8)** Mild widening with a moderate widening of the right posterior horn. **q/16)** Mild widening of frontal horns and moderate widening of posterior horns. **r/4)** Mild to moderate widening **s/5)** A pronounced widening of the third ventricle. Maybe this indicates atrophy of the basal ganglia and thalami. **t/2)** Moderate widening

**Fig. 2:**
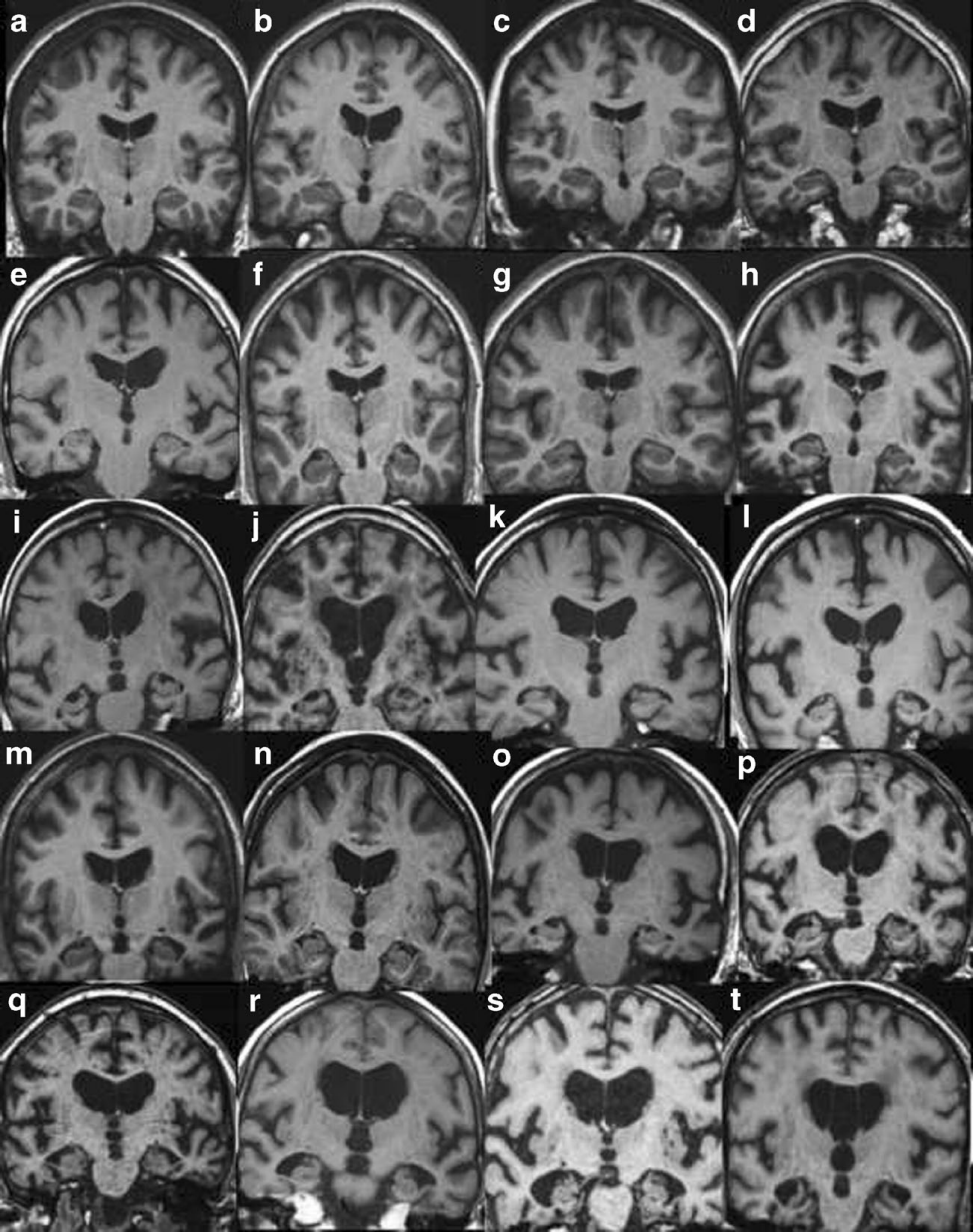

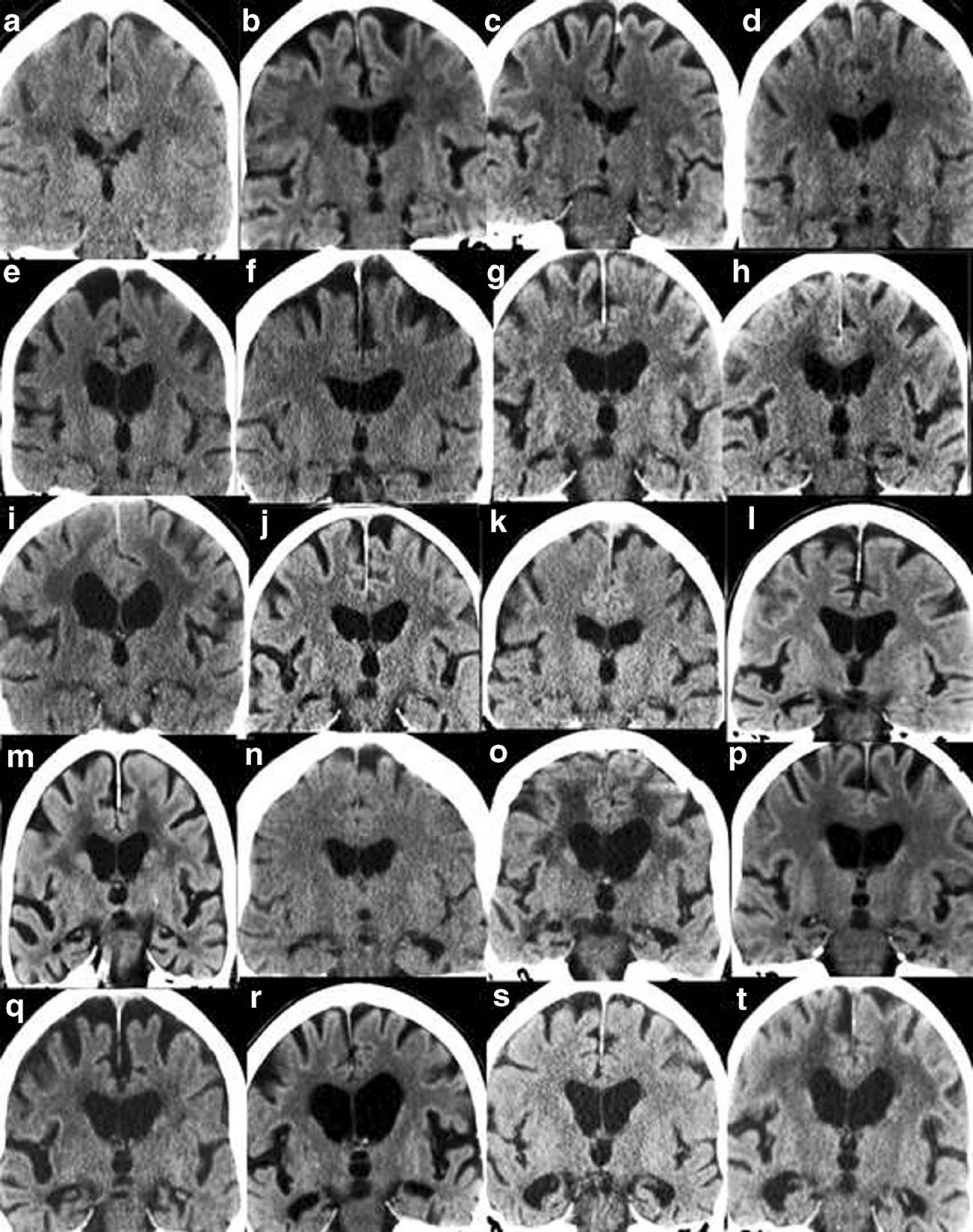
**A.** Rating of medial temporal atrophy (MTA) on MR. Interpretation key. Answers given as (right/ left). Numbers equal the corresponding image in the supplementary PDF file. **a/6)** MTA0/0. **b/16)** MTA0 right and MTA0-1 on the left side. **c/2)** MTA0/1. **d/4)** MTA1/0-1. Both are normal. **e/5)** MTA1/1. **f/17)** MTA1/1. **g/11)** MTA1/1. Note the small cyst in the right hippocampus. **h/8)** MTA1/1. **i/1)** MTA2/2. Artefacts are not disturbing the rating process. The parahippocampal gyri are not atrophied. The sulci are closed. **j/10)** MTA2/2. **k/15)** MTA2/2. On the right side the image is too far behind the rating point. Left is optimal here. **l/18)** MTA2/2 **m/14)** MTA1/3. On the right side MTA is turning into MTA2 and on the left side MTA just passed from MTA2 into MTA3. **n/–12)** MTA2/2.The left MTA is turning into MTA3. **o/13)** MTA2/2. Here you need to have surrounding images to be sure of the grading. Right side might turn into MTA3. **p/3)** MTA3/2. A fetal band/septum on the left side. Cut it in your mind and the hippocampus will be released. **q/9)** MTA2-3 /2. Here it is difficult to rate. You need to have several images in a row, but even then it is difficult. The hippocampi are atrophic but there is no passage of cerebrospinal fluid around the hippocampi in this image **r/7)** MTA3/3. The widening of the ventricles are pronounced and sometimes the distinction between widening of ventricles and hydrocephalus of other reasons is difficult. In this case the entorhinal cortex and parahippocampal gyri are atrophied as well which makes atrophy as a cause more likely. **s/19)** MTA4/ 3–4. This image is noisy and it’s difficult to distinguish the entorhinal cortex from the hippocampi and parahippocampal gyri. **t/20)** MTA4/4.The right side is more atrophic than the left side. **B.** Rating of MTA on CT. Interpretation key. Answers given as (right/ left**).** Numbers equal the corresponding image in the supplementary PDF file. **a/19)** MTA0/0. **b/10)** MTA1/0. **c/16)** MTA0-1/1. **d/7)** MTA1/0. Basal artefacts and an acute infarction in the left temporal lobe. **e/14)** MTA1/1. **f/18)** MTA1/2. **g/11)** MTA2/2. Maybe too anterior on the right side for rating. **h/12)** MTA1/2-3. Difficult to rate on the left side. Hippocampus is thin. MTA3 is more likely. **i/20)** MTA2/2. **j/3**) MTA2/2. It is too anterior to rate the right side, parts of amygdala are still remaining and on the left side it is almost too far posteriorly. **k/6)** MTA1/2.Basal artefacts make it more difficult to rate. Left side is MTA2 but is turning towards MTA3. **l/1)** MTA3/0.Asymmetric MTA. Note the wider Sylvian fissure on the right side. **m/4)** MTA2-3.More atrophy on the left side. Thin hippocampi, wide temporal horns, wide sulci beneath, wide hippocampal sulci. **n/15)** MTA2/3. Difficult to be sure on a single image. **o/8)** MTA2/3. Right side is between 2 and 3 and left side between 3 and 4. Note the differences in lateral ventricle size. **p/2)** MTA3/3. Difficult to rate on just one image. The hippocampi adheres to the ventricular wall on both sides. The hippocampi are thin and the fissura hippocampi are wide. The temporal horns are starting to widen. Parahippocampal gyri are thin and the sulci beneath it is wide. **q/13)** MTA3/3.The hippocampus on the left side has a strange formation - maybe it is not fully rotated. **r/17)** MTA3/3.Too anterior on both sides. Parts of amygdala are remaining bilaterally. Hippocampi are thin but the sulci beneath are not wide. **s/5)** MTA4/4. Right side is a bit too anterior for rating. **t/9)** MTA4/4

**Fig. 3:**
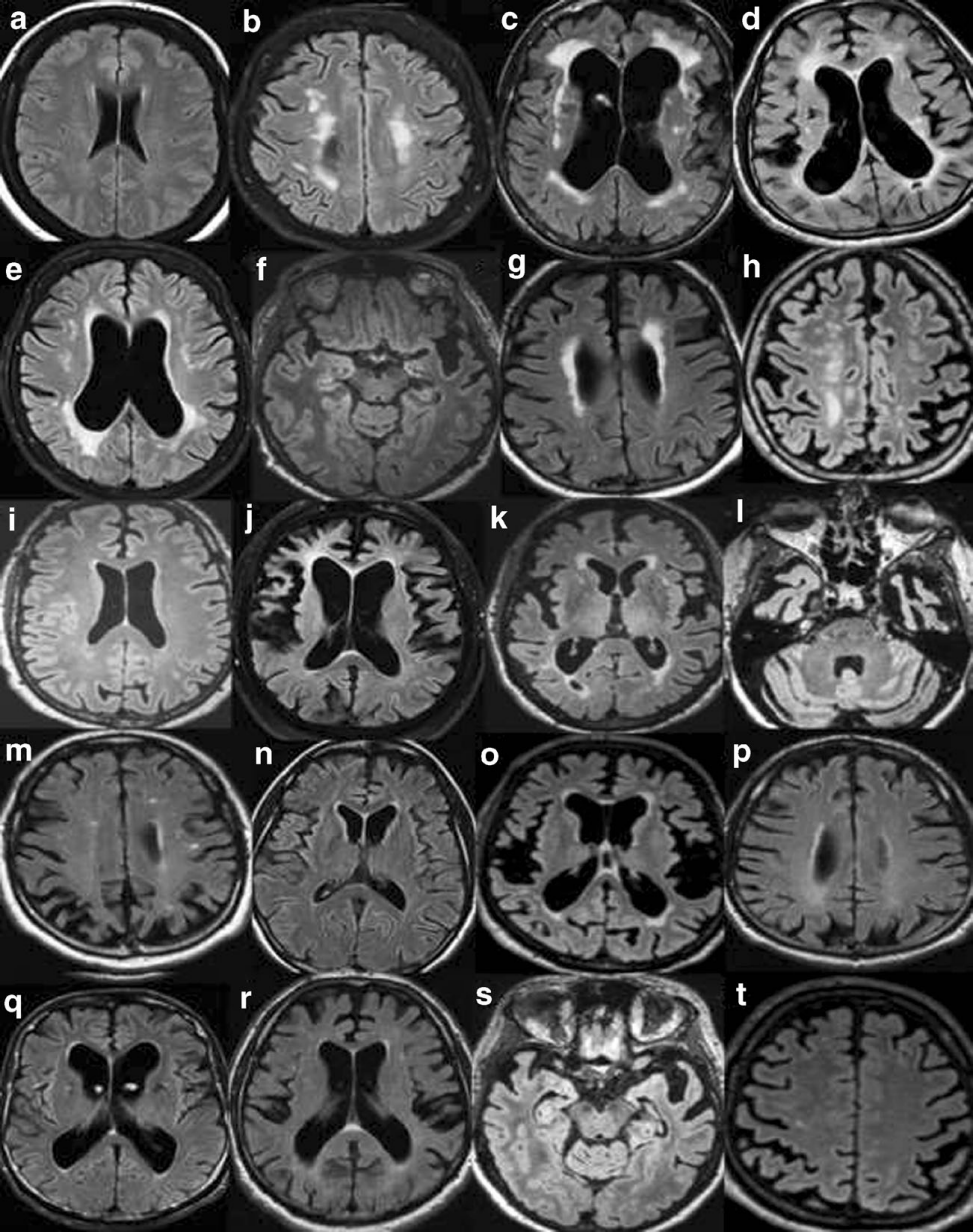

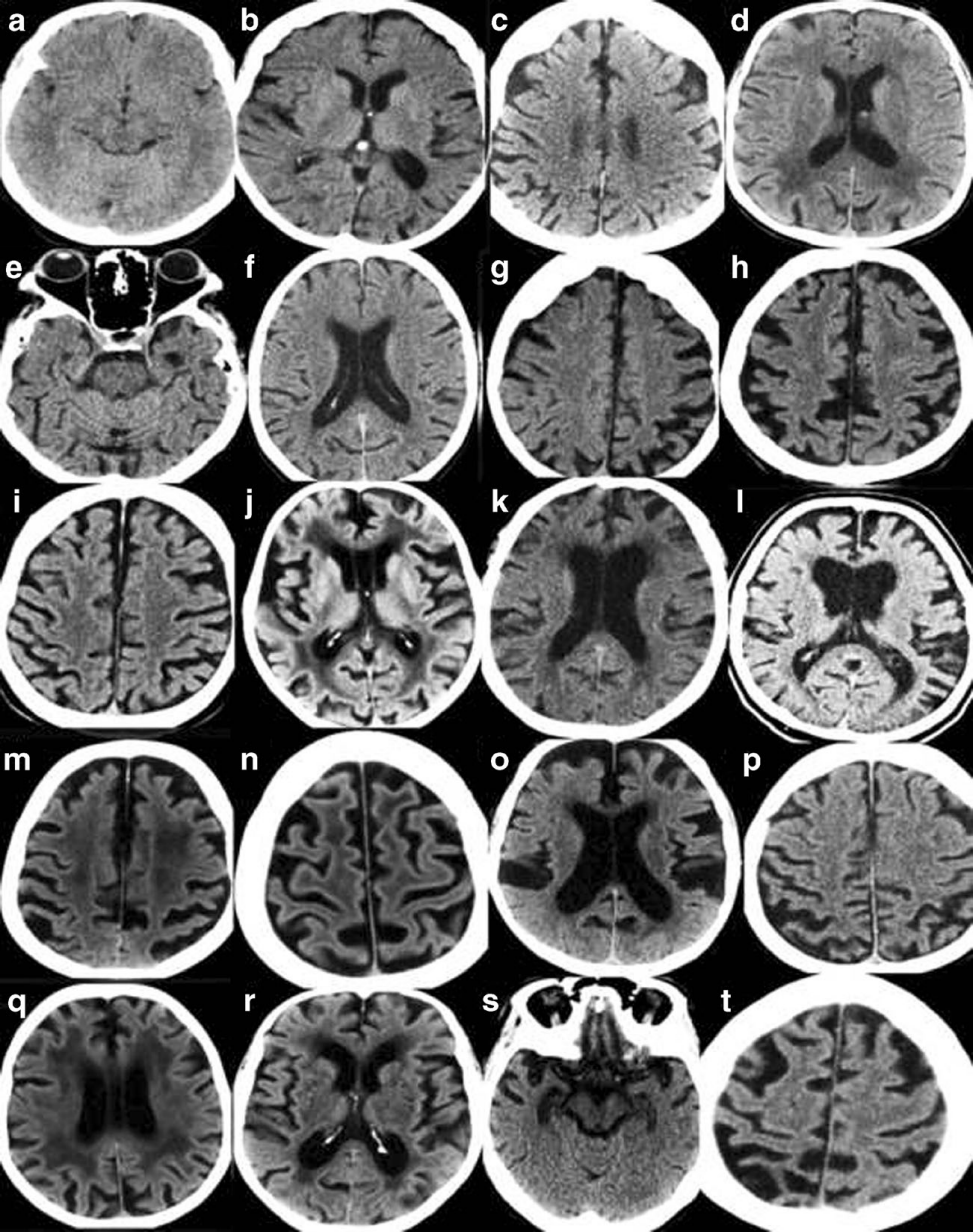
**A**. Global cortical atrophy (GCA) rating on MR. Interpretation key. Numbers equal the corresponding image in the supplementary PDF file. **a/1)** GCA0. **b/17)** GCA0-1. **c/7)** GCA0-1. Note the powerful widening of the lateral ventricles and especially the frontal horns. Normal pressure hydrocephalus? **d/8)** GCA0-1 with bifrontal GCA1. Note the widening of the posterior horns of the lateral ventricles. **e/14)** Bifrontal GCA1 and biparietal GCA0. Note the wide lateral ventricles. **f/11)** GCA0. Some atrophy of one or two of the temporal gyri and widening of the Sylvian fissure, especially on the left side, GCA2. **g/2)** GCA1 in the parietal lobe. GCA1 and 2 in the frontal lobes. **h/4)** GCA1. GCA2 in the left side at the junction between the frontal and parietal lobes. **i/5)** GCA1-2, but mostly GCA2. **j/16)** GCA1 going towards GCA2. **k/9)** GCA1. Maybe atrophy in the frontal midsagital region with mild atrophy of the anterior cingulate gyri, GCA2? **l/6)** GCA2 **m/13)** BifrontalGCA1 and biparietal GCA2. **n/20)** GCA2, more atrophy left parietal**. o/18)** Bifrontal GCA2 and biparietal GCA1. **p/19)** GCA0 except for anterior gyri in both temporal lobes, GCA 2–3 left temporal and GCA1-2 right temporal. **q/3)** GCA2 in the left frontal lobe and GCA2-3 in the right. A few gyri with GCA3.GCA0-1 in the parietal lobes. Note the widening of the frontal horns of lateral ventricles and the atrophy of the anterior cingulate gyri. **r/10)** BifrontalGCA 2–3 and biparietal GCA0-1. Note atrophy of the cingulate gyrus and wide frontal horns. **s/12)** GCA3 in the temporal lobe on the left side. GCA1 on the right side and GCA2 in the cerebellum. **t/15)** GCA2 and maybe GCA3 in the Sylvian fissure. Biparietal GCA1. **B.** Global cortical atrophy (GCA) rating on CT. Interpretation key. Numbers equal the corresponding image in the supplementary PDF file. **a/1)** GCA0 **b/17)** GCA0-1. **c/5)** GCA1. **d/2)** GCA1. **e/8)** GCA1. **f/10)** GCA1. **g/18)** GCA1 in the right hemisphere and GCA2 in left hemisphere. **h/9)** GCA1 in the more frontal part of the frontal lobes. GCA2 in general. **i/19)** In-between GCA1-2, but closer to GCA2. **j/13)** In-between GCA1-2, GCA2 bifrontal. Note the cavum septi pellucidi. **k/3)** Bifrontal GCA2 and biparietal GCA1.Note the wide frontal horns. **l/6)** Bifrontal GCA2, biparietal GCA1. **m/7)** GCA2 fronto-parietal. **n/20)** GCA2. **o/16)** GCA2, frontal lobe left.GCA1 in the right frontal lobe and GCA0 in the parietal lobes. Note the atrophy in the anterior cingulate gyri. **p/11)** Biparietal GCA2 and bifrontal GCA1. **q/14)** Another image of the same patient, GCA2. **r/12)** Note the side difference. GCA2 along the left hemisphere, GCA1 on the right side. **s/15)** GCA2 in supraorbital part of the frontal lobes and in the anterior part of the temporal lobes.GCA0 in the more posterior part of the temporal lobes. **t/4)** GCA3 in a few parietal gyri on the right side. Bifrontal GCA2 and GCA1 left parietal

**Fig. 4:**
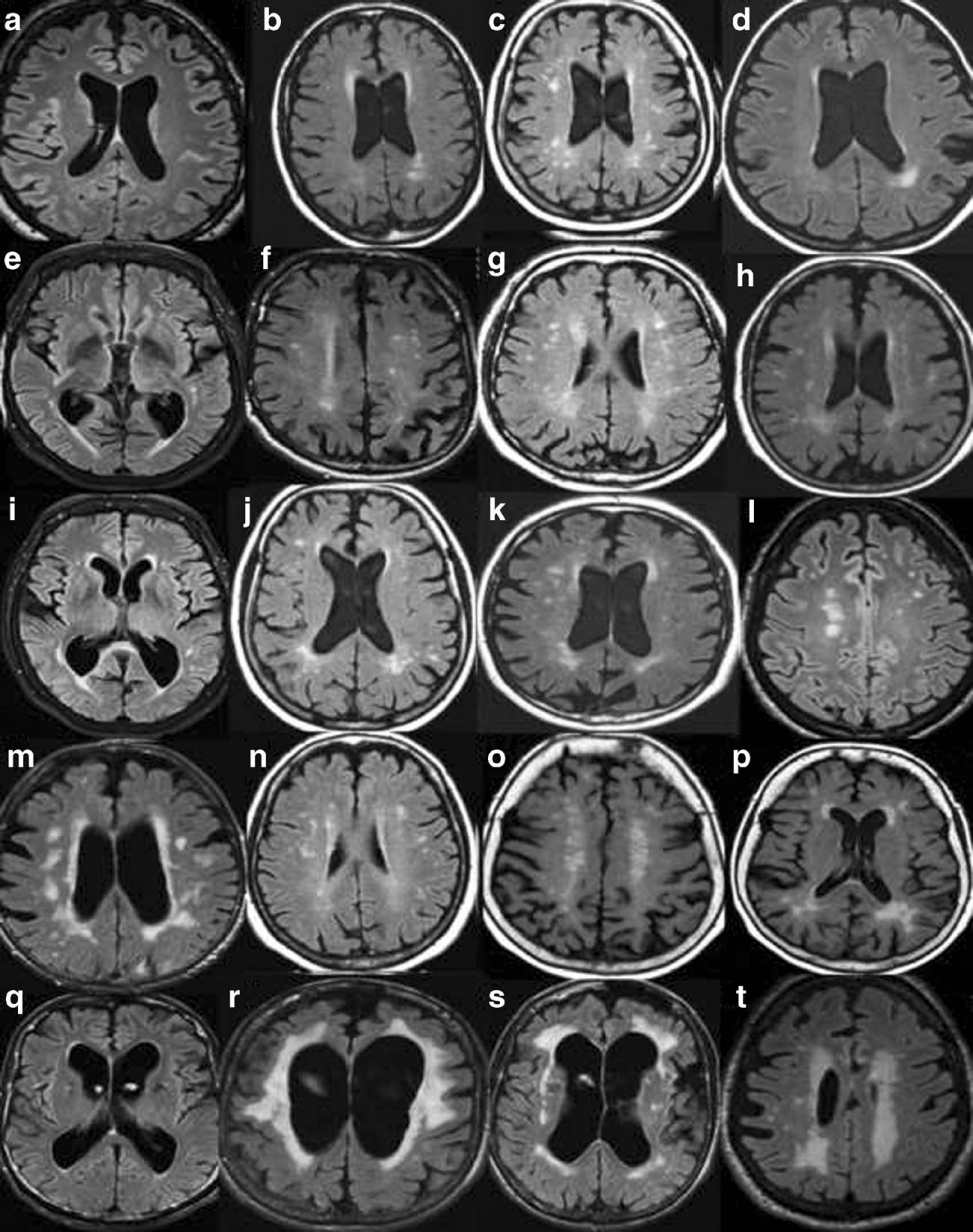

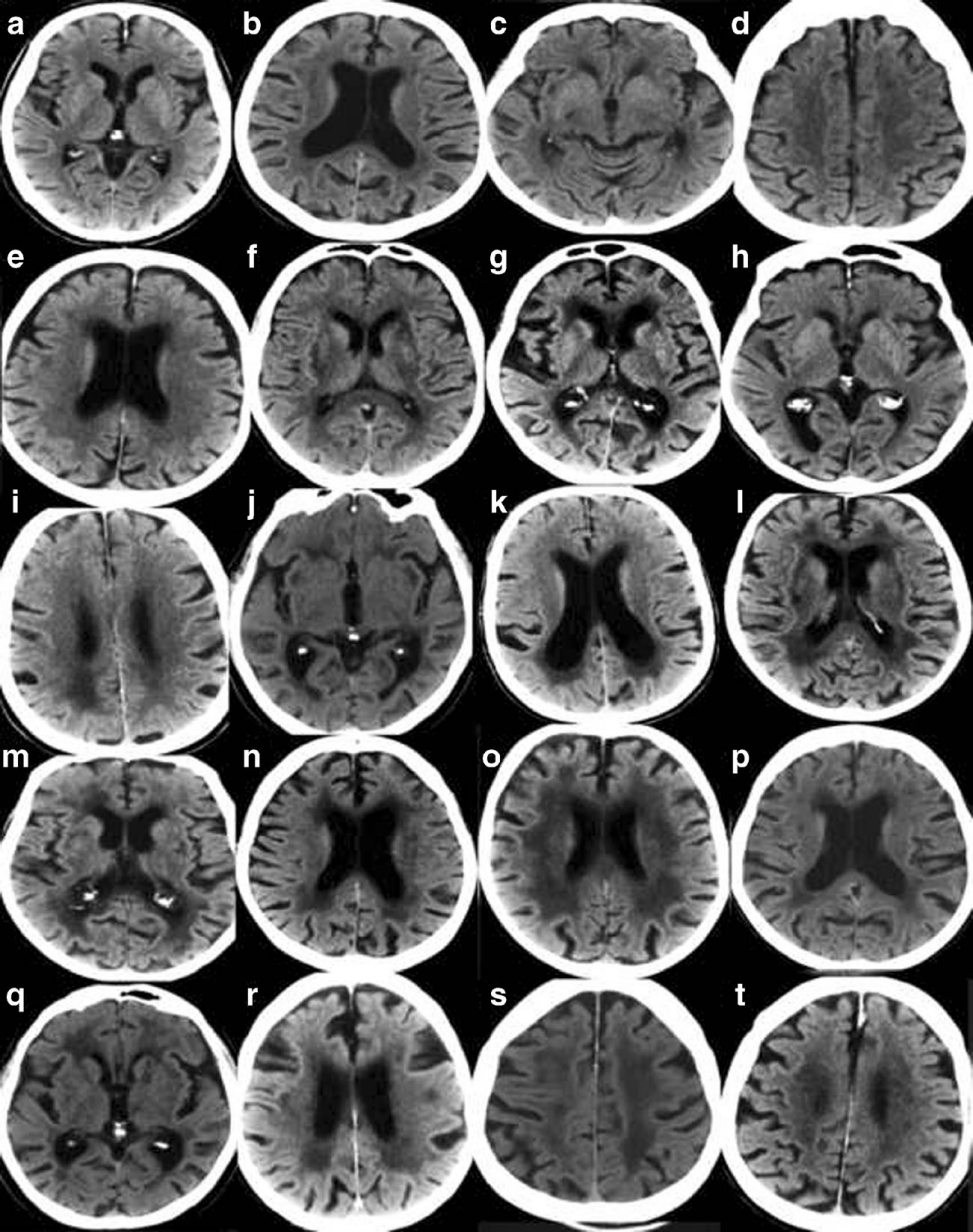
**A.** Rating of white matter hyperintensities (WMH) according to the Fazekas scale on MRI. Interpretation key. Numbers equal the corresponding image in the supplementary PDF file. **a/2)** Fazekas 0. Entire cerebrum is without punctate WMH. WMH in the pons and in the cerebellum are not included in the rating – but should be mentioned in the referral. **b/19)** Fazekas 1. **c/14)** Fazekas 1. Still punctate WMH. **d/15)** Fazekas 1. One single lesion < 2 cm and some punctate lesions. **e/1)** Fazekas 1. Small punctate WMH grouped but separated along the left posterior horn of the lateral ventricle. **f/3)** Fazekas 1. Grouped but not linked WMH. **g/9)** Fazekas 1. Many separate WMH. **h/12)** Fazekas 1. **i/18)** Fazekas 1. All WMH are seen as separate dots. No connecting bridges. **j/17)** Fazekas 2, Connecting bridges. **k/20)** Fazekas 2. Connecting bridges close to the right posterior horn. **l/4)** Fazekas 2. WMH are linked, but can still be seen as separate lesions. **m/7)** Fazekas 2. The WMH are beginning to confluate. Individual WMH are still seen. **n/13)** Fazekas 2. Connecting bridges between lesions. **o/10)** Fazekas 2. Borderline to Fazekas 3 with bridging between WMH still visible, starting to confluate. **p/11)** Fazekas 2–3. **q/8)** Fazekas 3. Borderline Fazekas 2, but the WMH on the left side measures more than 2 cm and grouped WMH are confluent to some extent. **r/5)** Fazekas 3. **s/6)** Fazekas 3. Confluent lesions around the frontal horns and confluent thin lesions in the right external capsule, and not so pronounced in the same area on the left side. **t/16)** Fazekas 3 **.B.** Rating of white matter hyperintensities (WMH) according to the Fazekas scale on CT. Interpretation key. Numbers equal the corresponding image in the supplementary PDF file. **a/2)**Fazekas 0–1. No lesions are seen, however punctate lesion may exist. **b/7)** Fazekas 1. Punctate lesions frontal bilaterally, more obvious right frontal. **c/11)** Fazekas 1. Lesion in the external capsule bilaterally. **d/17)** Fazekas 1.Single lesion frontal left. **e/15)** Fazekas 1. Just small caps around the frontal horns and 2 smaller diffuse punctate lesions in the frontal lobe on the right side. **f/20)** Fazekas 2. Lesions in the external capsules with connecting bridges. **g/3)** Fazekas 2. Small lesions close to frontal and posterior horns and small lesions in the external capsules.**h/4)** Fazekas 2. Small lesions in the external capsule, bilaterally. **i/13)** Fazekas 2. **j/18)** Fazekas 2. Lesions in the external capsules. **k/12)**Fazekas 2–3 depending on the size of the lesions surrounding posterior horns. **l/1)**Fazekas 3. Confluating lesions around the frontal and posterior horns and in both external capsules.**m/5)** Fazekas 3.**n/6)** Fazekas 3. **o/8)**Fazekas 3. **p/9)** Fazekas 3. **q/10)** Fazekas 3. **r/14)** Fazekas 3. **s/19)** Fazekas 3. **t/16)** Fazekas 3

In order to maximize the yield of brain imaging in the clinical work-up of patients with cognitive impairment, it is important that imaging findings are reported consistently and according to established, validated rating scales [1, 19]. Here, we present a comprehensive overview of the most important findings on routine CT and MRI in dementia including visual scoring according to established rating scales. It should be emphasized that the assessment also can be performed on CT, which is still the most widely used modalility in routine dementia investigations. We suggest that these methods are incorporated into clinical protocols and are used for routine clinical image interpretation. Training to use the scales and available reference images is important for consistent accurate results of the visual evaluation [19]. Therefore we also provide image examples using the different rating scales (Figs. 1, 2, 3 and 4) and separate teaching material.

## MRI protocol

Three basic MRI-sequences should preferebely used for the visual assessment : a T1-weighted 3D sequence to assess structural changes, a T2-weighted fluid attenuated inversion recovery (FLAIR) and a T2-weighted turbo spin echo sequence to detect other pathological changes, primarily white matter changes. We also propose the addition of a sequence for assessing microbleeds that could aid in the diagnosis of small vessel disease, preferably the susceptibility weighted imaging (SWI) sequence.

## Structured assessment of CT and MRI

A structured radiological report with description of the imaging findings is required to provide optimal information to the referring clinician. The width of the sulci and the ventricles, the degree of medial temporal lobe atrophy, white matter changes and the occurrence of infarctions, mass effect or other changes, leading to secondary dementia, must be included. Comparison with previous radiological examinations is very important since relatively rapid progression of atrophy supports the suspicion of neurodegenerative dementia.

**Table:** 

**A radiological report should describe:**
• Medial temporal lobe atrophy (MTA) (Scheltens score with explanation)
• General or local widening of sulci (Global Cortical Atrophy (GCA) stage with explanation)
• Width of ventricles
• White matter hyperintensities (WMH) (score according to Fazekas scale with explanation)
• Size and position of infarcts
• Other changes (tumour, normal pressure hydrocephalus, subdural hematoma etc.)
• Comparison with previous examinations (progression of atrophy or white matter changes etc.)
• CONCLUSION: assessment of findings in relationship to clinical suspicion and other examinations such as CSF, PET or SPECT.

### Medial temporal lobe atrophy (MTA) – Scheltens scale

A rating scale for visual assessment of medial temporal atrophy (MTA) was developed by Philip Scheltens’ research group in Amsterdam in 1992 [20, 21]. This scale has been used in a large number of studies and is also included in the research criteria for the diagnosis of Alzheimer’s disease [22]. The height of the hippocampus, as well as the width of the temporal horns and choroid fissure are assessed on standardized coronal images.The assessment is conducted according to a five-point scale of 0–4, where MTA 0 and MTA 1 are considered normal. In MTA 0, the width of the choroid fissure and temporal horn, and the height of the hippocampal formation are normal; in MTA 1, only the width of the choroid fissure is slightly increased. MTA2-4 represent increasing degrees of atrophy. MTA 2 has increased width of the choroid fissure and temporal horn, and slightly decreased height of the hippocampus. MTA 2 is pathological in patients younger than 70 years of age. MTA 3 has severly increased width of the choroid fissure and temporal horn, and decreased height of the hippocampal formation. MTA 3 is pathological in all patients under 80 years of age. MTA 4 represents severe increase in width of the choroid fissure and temporal horn, and a severly decreased hippocampal height. MTA 4 must always be perceived as pathological, regardless of the patient’s age (Fig. 1) [23].Several studies have investigated the cut-off for normal and pathological MTA as well as the effect of demographic variables, such as age, gender and education; average values of the left and the right side have been proposed [24, 25]. However average values may not be optimal for use in the clinical routine and more suited for research purposes.

The MTA scale was originally developed for the assessment of MR images, but current high quality CT scans can also be used (Fig. 2) [26]. The MTA scale has demonstrated significant correlation with manual measurements of the hippocampus, and increased clinical relevance when seen in association with cognitive function [23]. The sensitivity and specificity are comparable to automated methods for volume measurement and calculations of volume of cortical thickness [27]. Inter- and intrarater reliability are very high in experienced raters, but may be slightly lower for raters if no prior consensus between raters has been established on cases [28].

### Global cortical atrophy – GCA Scale

The global cortical atrophy (GCA) scale was first developed by Pasquier et al. in 1996 for the purpose of assessing cerebral atrophy in patients with poststroke dementia [29]. The scale was subsequently further refined and adapted to enable quicker assessment in dementia [19]. Visual assessment of cortical atrophy reflects not only the degree of general cortical atrophy but also the degree of lobar and regional atrophy, which should be commented upon in the radiological report [30]. This is of particluar importance since atypical forms of AD are recognized with more frontal or posterior atrophy, rather than the temporal atrophy seen in more typical cases [31].

Widening of sulci may be secondary to atrophy of the cortex and/or the white matter, why the term cortical atrophy in a strict meaning should only be used when the cortical thickness has been measured. Widening of sulci and gyral volume loss can be assessed using a 4-point scale, GCA 0–3 [32]. Normal sulci have GCA grade 0, slight widening of sulci classifies as GCA 1, gyral volume loss is categorized as GCA 2 and pronounced widening of sulci with severe volume loss, so called “knife blade atrophy” is labelled GCA 3 (Fig. 3). Cortical atrophy can also be assessed with substansial agreement between CT and MRI [26].

### White matter changes – Fazekas’ scale

The Fazekas’ scale was first constructed in 1987 in order to standardize the visual assessment of white matter changes seen on MRI. The scale has been used in a large number of publications on white matter changes and is included here because of its simplicity and applicability on CT and MRI, and we consequently recommend its use in the clinical dementia assessment. The Fazekas’ scale includes assessment on axial T2-weighted or T2 FLAIR images for the whole brain and has four increments. Caps and bands, a rim of white matter hyperintensity around the ventricles, are seen as a normal finding in the brain. Grade 0 has no or occasional punctate white matter changes and grade 1 has multiple punctate white matter changes, which can be seen in all ages and is common in patients older than 65 years of age. The presence of a small number of such changes usually lack clinical significance. Grade 2 implies incipient confluence or bridging of punctate changes and grade 3 consists of confluent white matter changes. Grade 2 is regarded as pathological in patients younger than approximately 70 years of age, while grade 3 is always pathological (Fig. 4). A modified version of the Fazekas’ scale, the age related white matter changes (ARWMC) scale [33] includes analysis in further topographical regions.

## Extended imaging assessment

### Posterior atrophy – Koedam scale

The Koedam scale was developed 2011, to enable easy visual rating of posterior atrophy, that is atrophy of the parietal lobe including the precuneus, which may be a feature of Alzheimer’s disease [34]. Posterior atrophy has been suggested to be of specific importance in patients with early Alzheimer’s disease and no or minimal medial temporal atrophy [34]. Visual assessment is done in all three planes – axial, sagittal and coronal, with focus on the parietal cortex, the precuneus and the parieto-occipital sulcus. The scale has 4 increments with 0 = no atrophy, 1 = minimal atrophy, 2 = moderate atrophy, 3 = severe atrophy.

### Small vessel disease – The STRIVE criteria

Markers of small vessel disease have become increasingly important for the evaluation of patients with dementia, and have been suggested to have a contributory as well as a causative role in the neurodegenerative disease process. The “standards for research into small vessel disease” were established in 2013 summarizing the scales and criteria and proposing a terminology to be used in the realm of small vessel disease imaging. Important small vessel disease markers are: 1. Cerebral microbleeds, seen as punctate foci of hypointensity on susceptibility sensitive sequences. The location of cerebral microbleeds is also of importance to mention in reports as deep bleeds represent underlying hypertensive arteriopathy, and lobar cerebral amyloid angiopathy. 2. Cortical superficial siderosis implies gyriform linear hypointensities that have been suggested to be a sensitive markers of cerebral amyloid angiopathy. 3. Lacunes and recent small subcortical infarcts are other terms that preferably should be used depending on imaging manifestations. Although white matter changes are part of the small vessel disease spectrum, they are discussed separately above.

## Conclusion of the radiological report

Providing that the imaging has been conducted as part of a dementia investigation, a description of the findings, using the rating scales above, should be followed by an assessment of whether the findings are pathological or not taking the age of the patient into account. In addition, the radiologist should conclude if the described pattern could be consistent with the clinically suspected dementia disorder. Atrophy of the medial temporal lobes can support a clinical suspicion of Alzheimer’s disease, especially if there is a progression compared with previous imaging studies. It should, however, be noted that atrophy of the medial temporal lobes may also be found in other dementia disorders , e.g. Lewy Body Dementia and frontotemporal dementia. Vascular dementia can be considered unlikely if signs of cerebrovascular ischemia, strategical infarcts or microbleeds are missing. The final impression will thus provide a summary of the general imaging assessment, and also relate to clinical suspicion, and, if available, other examinations performed, such as a PET scan (e.g. glucose metabolism) or a SPECT (regional blood flow). This will improve the accuracy of the diagnosis of dementia disorders in clinical practice.

## Electronic supplementary material

Below is the link to the electronic supplementary material.

ESM 1 (PDF 846 kb) (PDF 801 kb)

ESM 2 (PDF 891 kb) (PDF 998 kb)

ESM 3 (PDF 882 kb) (PDF 787 kb)

ESM 4 (PDF 821 kb) (PDF 709 kb)
