# Supplementary figures and images for: Imaging biomarkers of dementia: recommended visual rating scales with teaching cases
Source: Insights Imaging. 2016 Dec 21;8(1):79–90. doi: 10.1007/s13244-016-0521-6 (PMC5265189; doi:10.1007/s13244-016-0521-6)

Healthy, 25 year of age

1

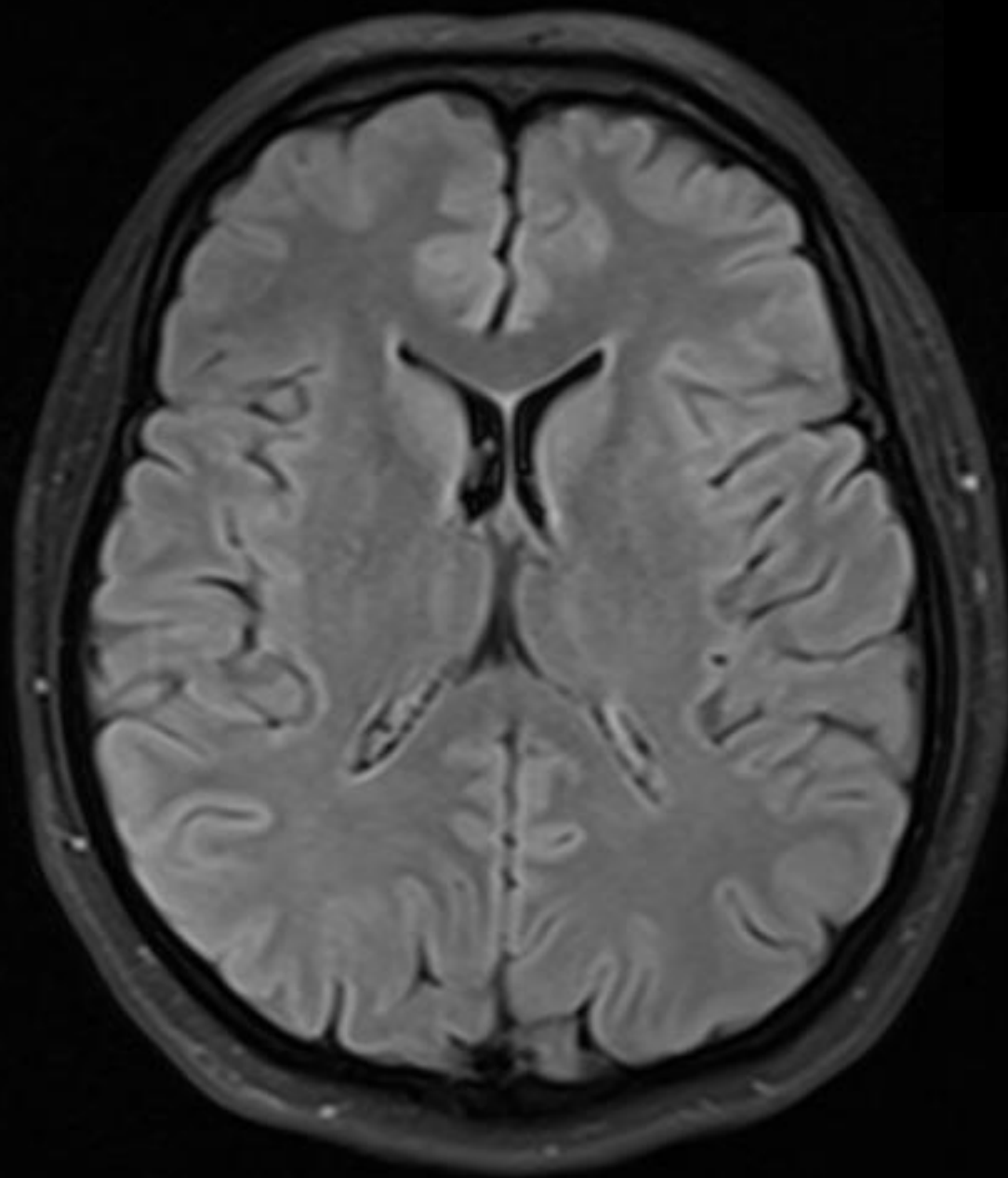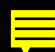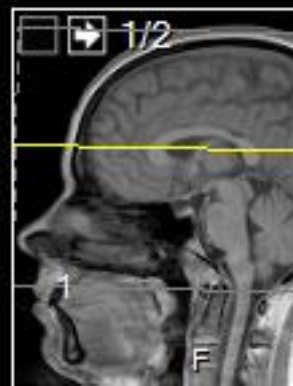

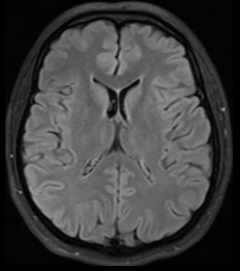

2

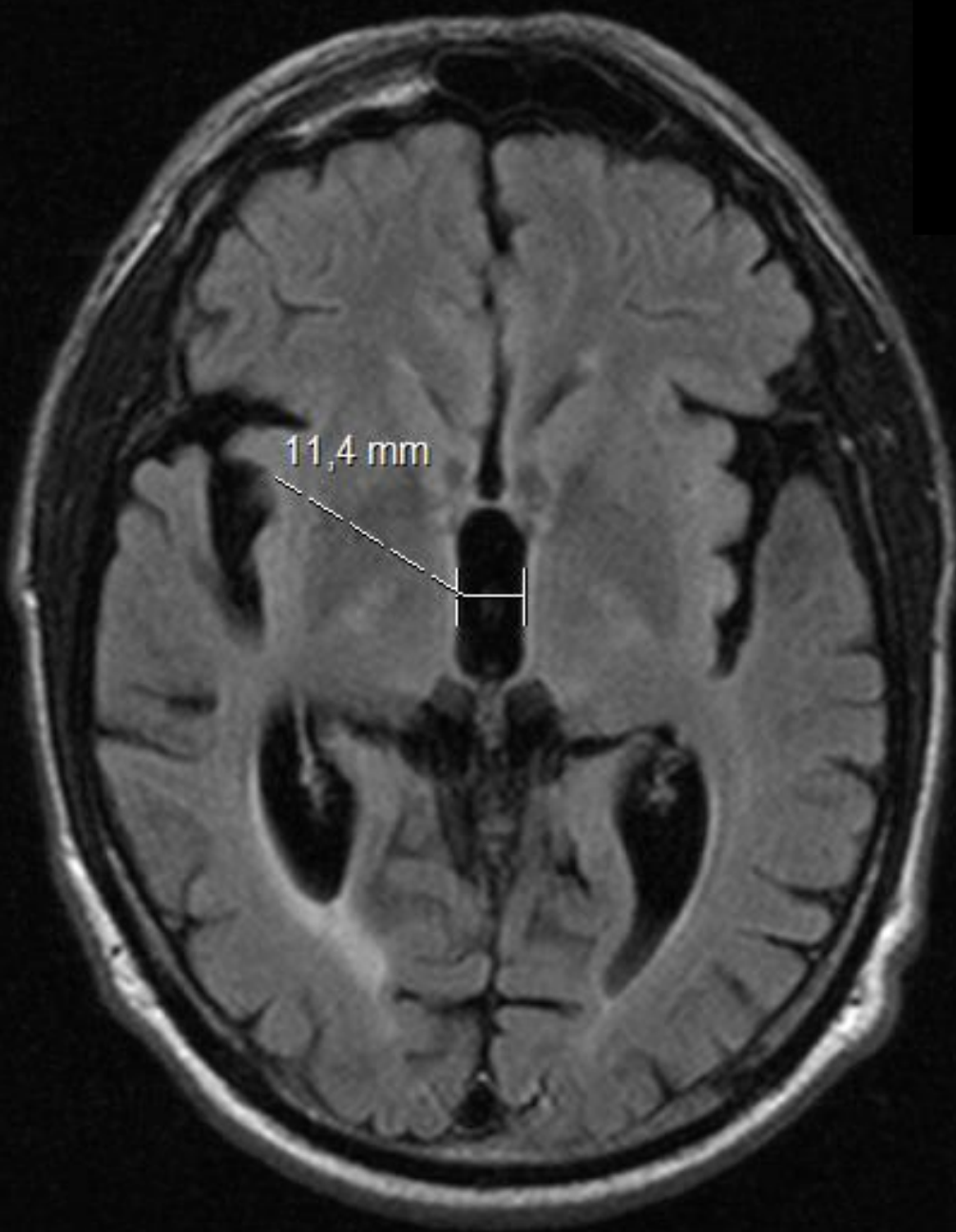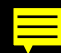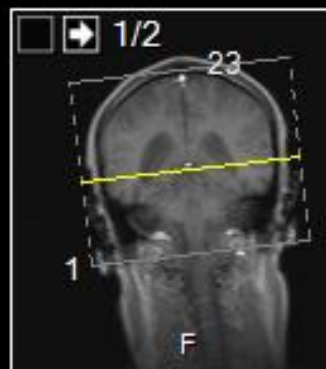

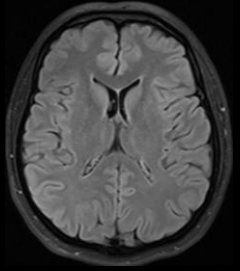

3

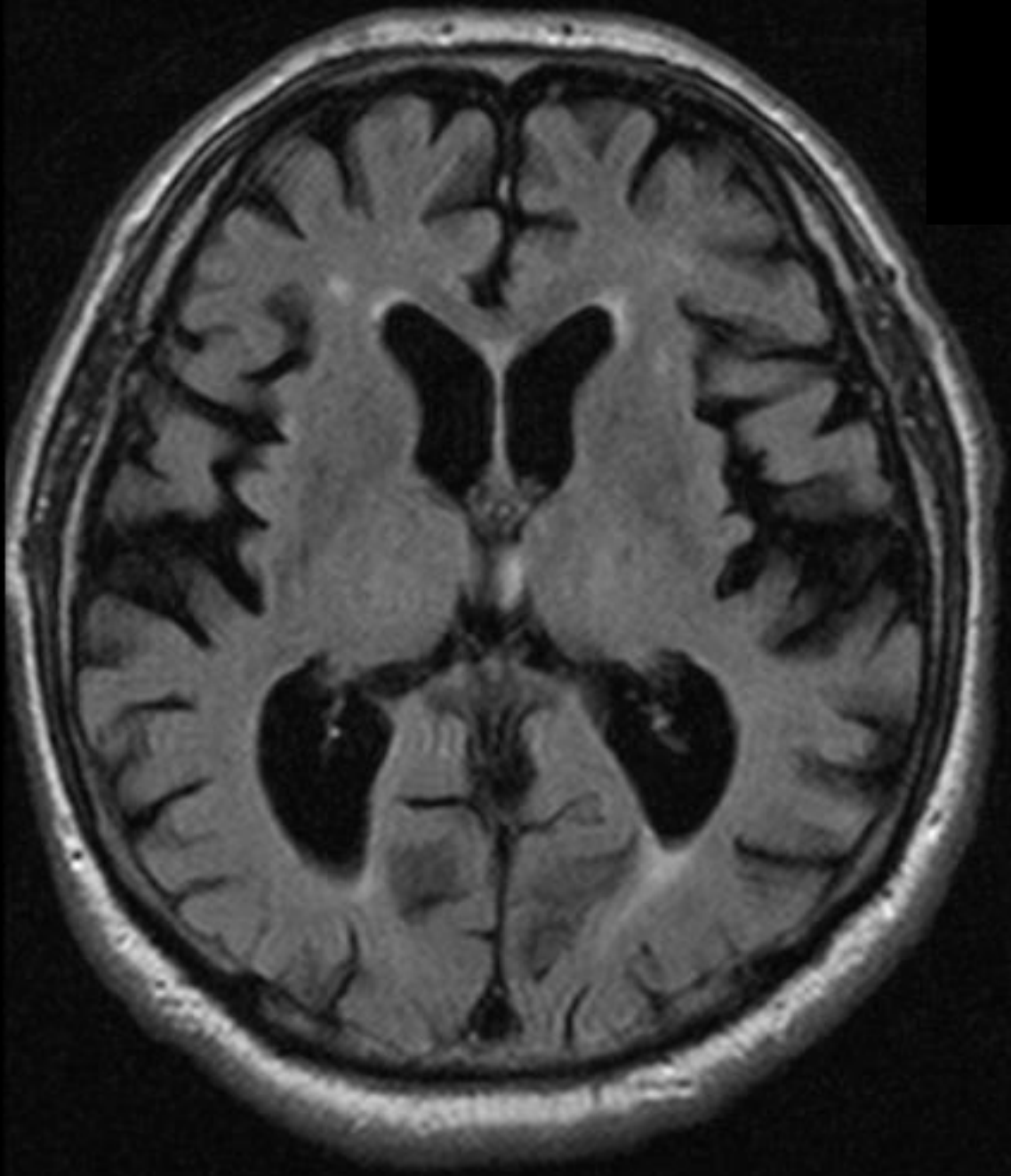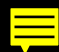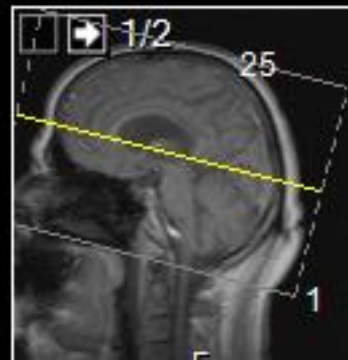

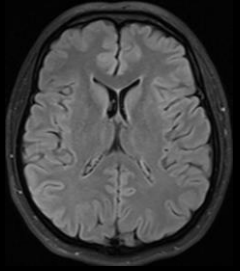

4

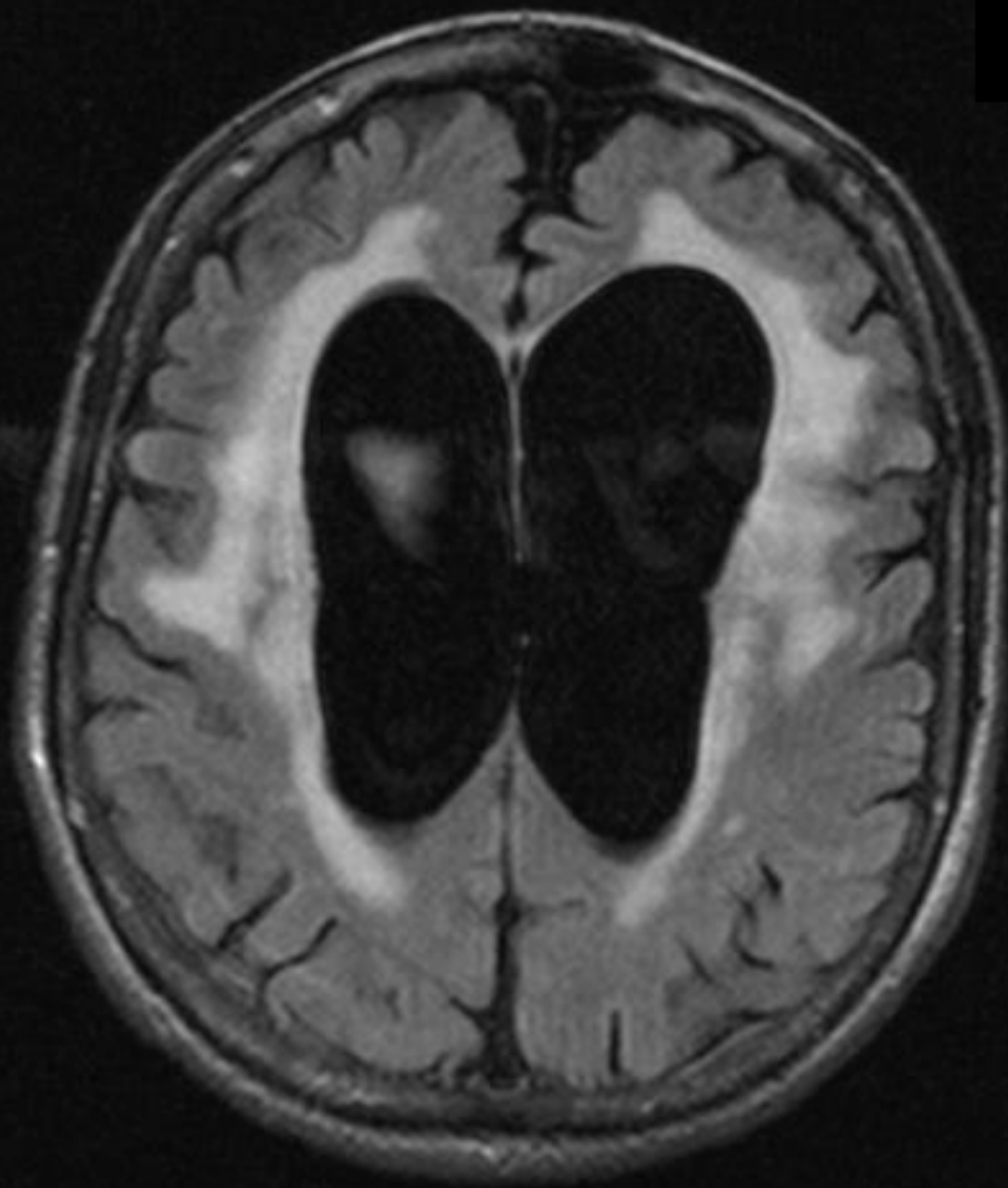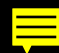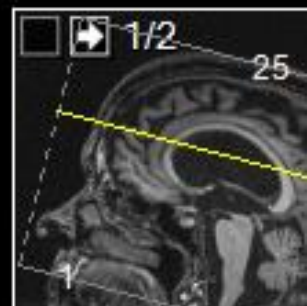

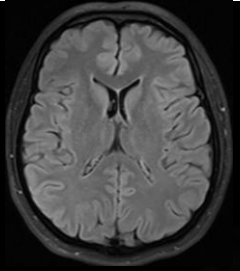

5

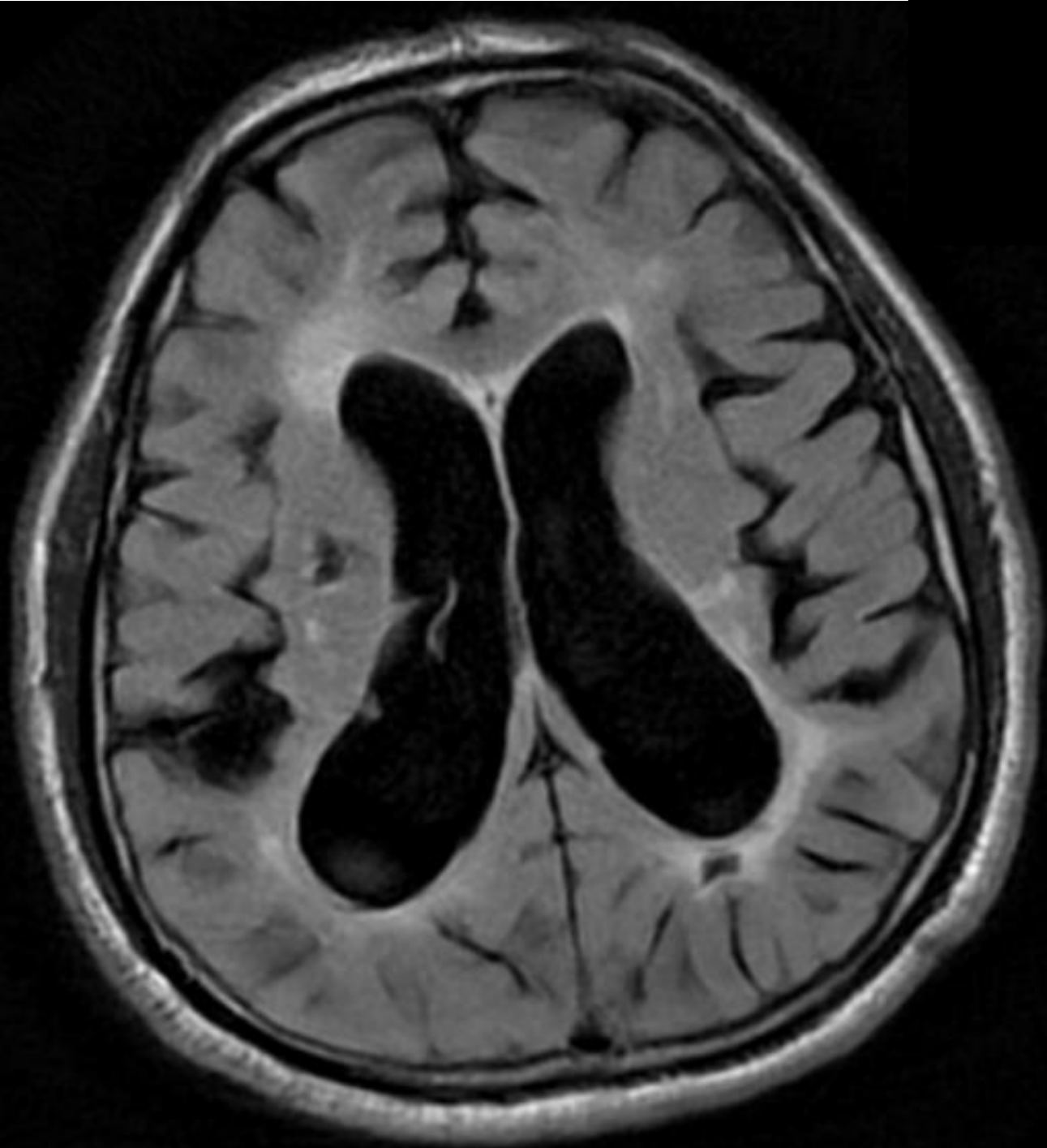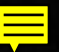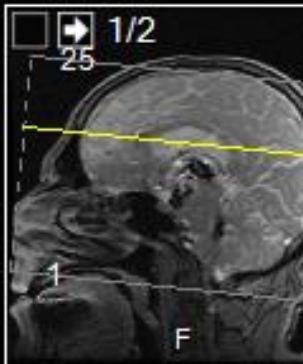

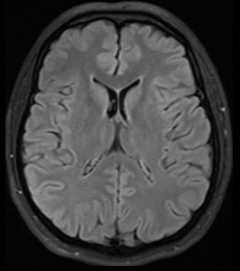

6

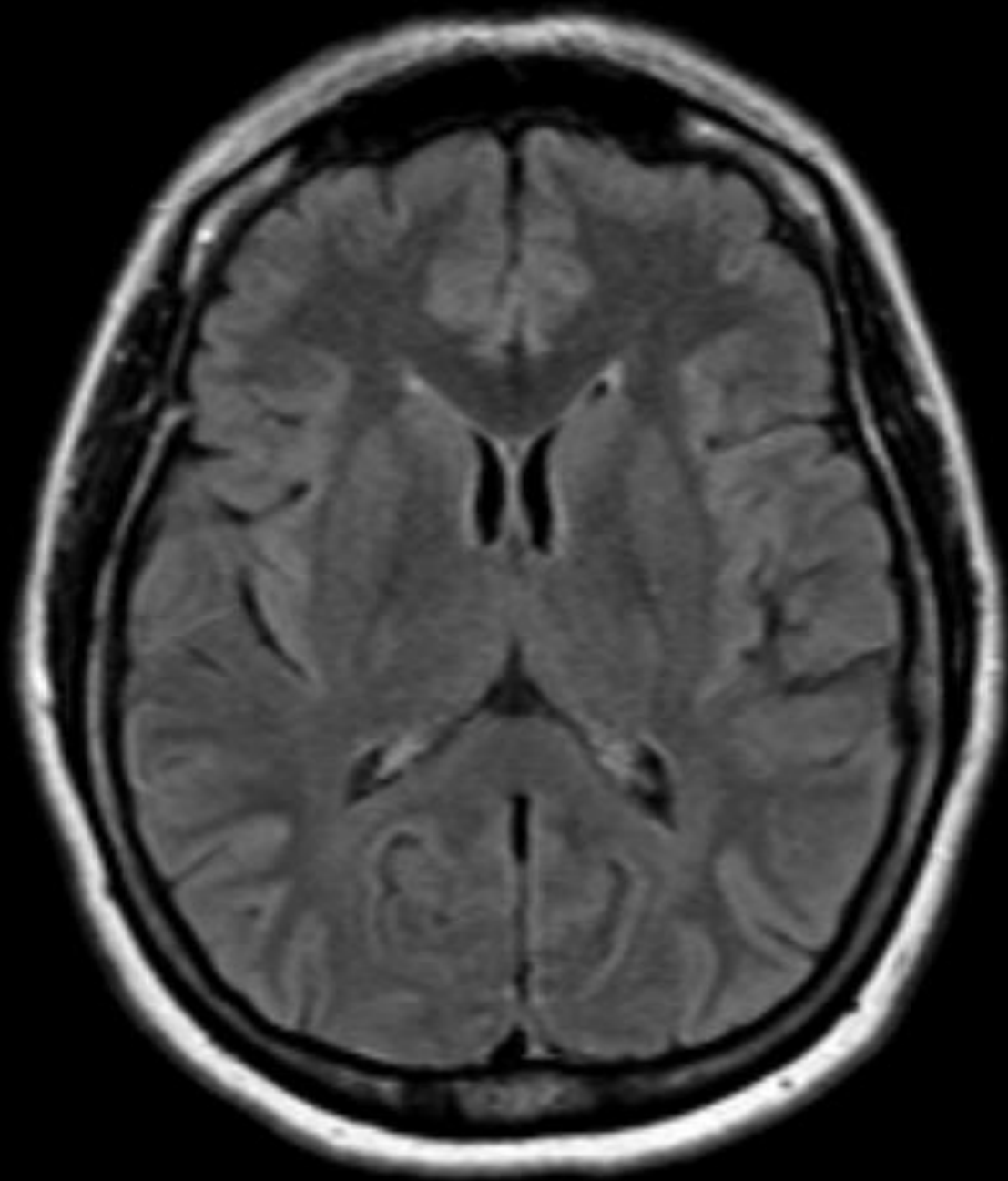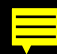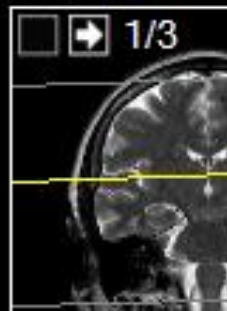

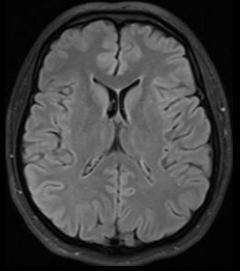

7

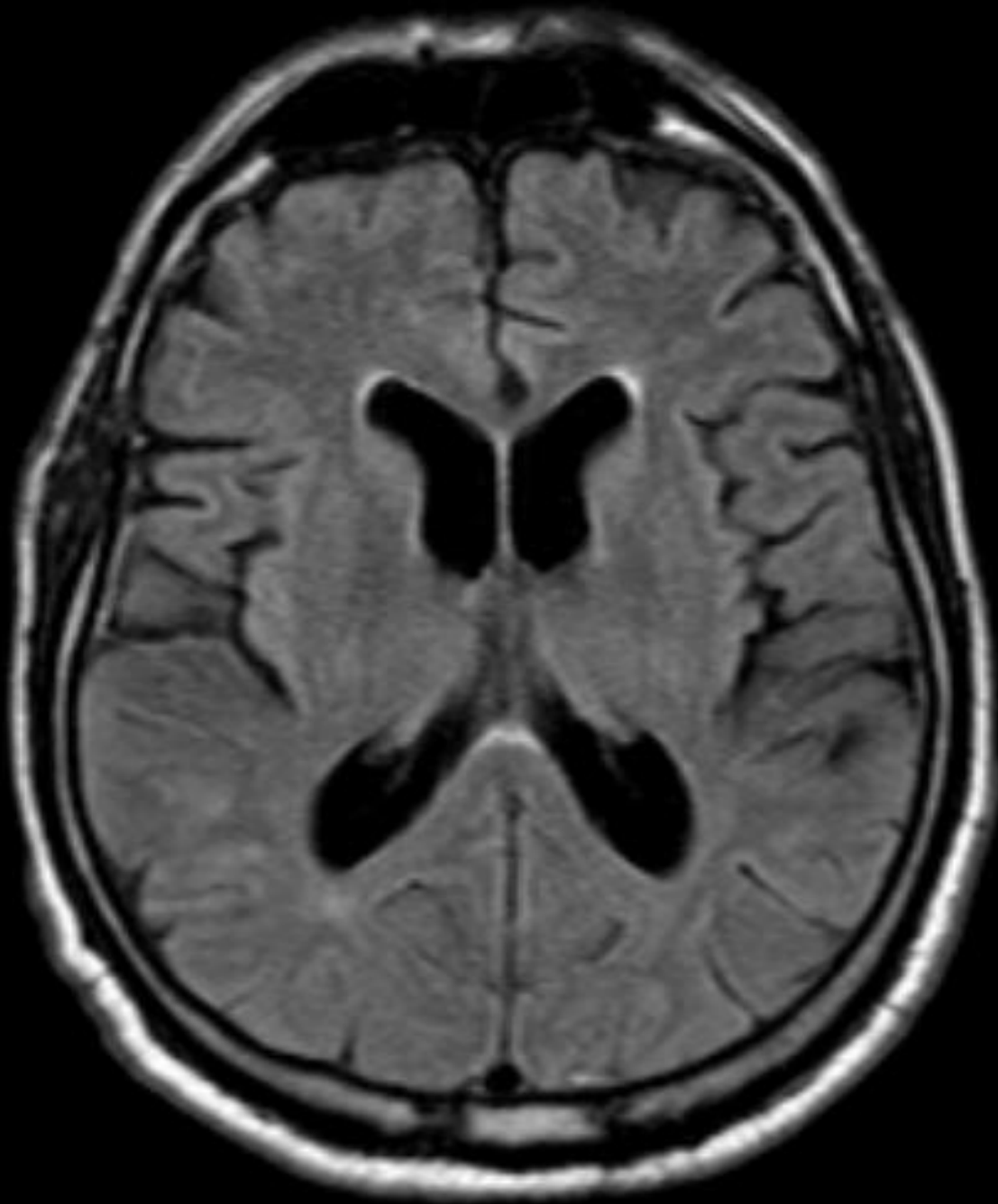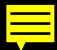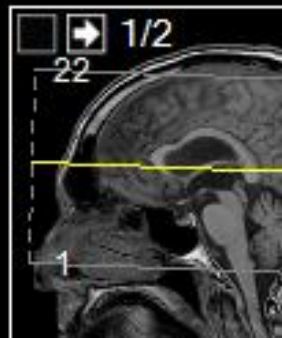

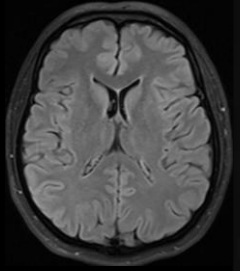

8

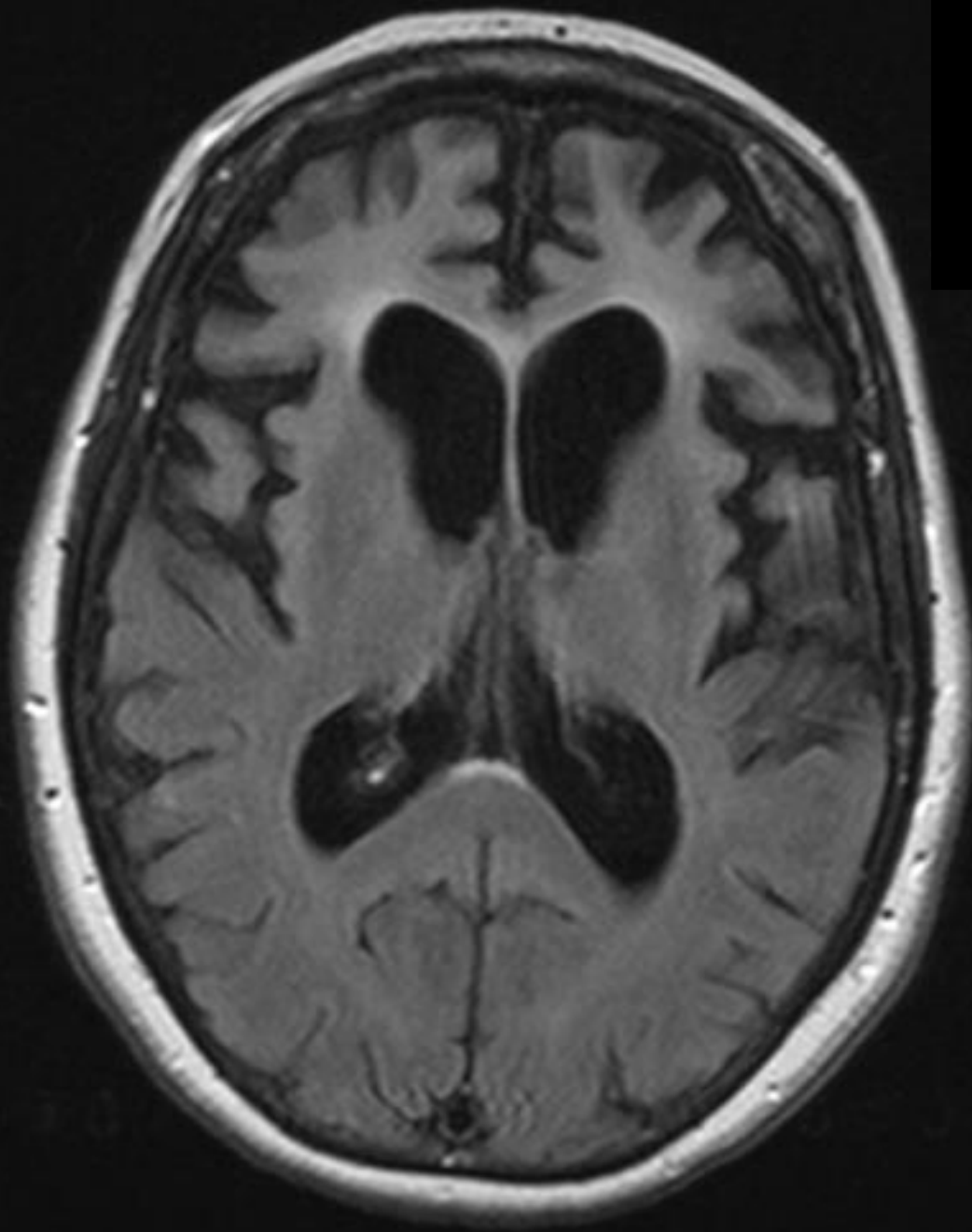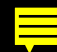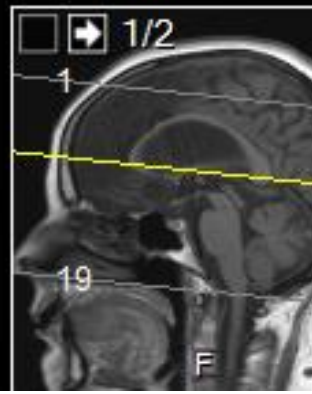

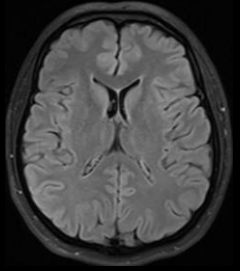

9

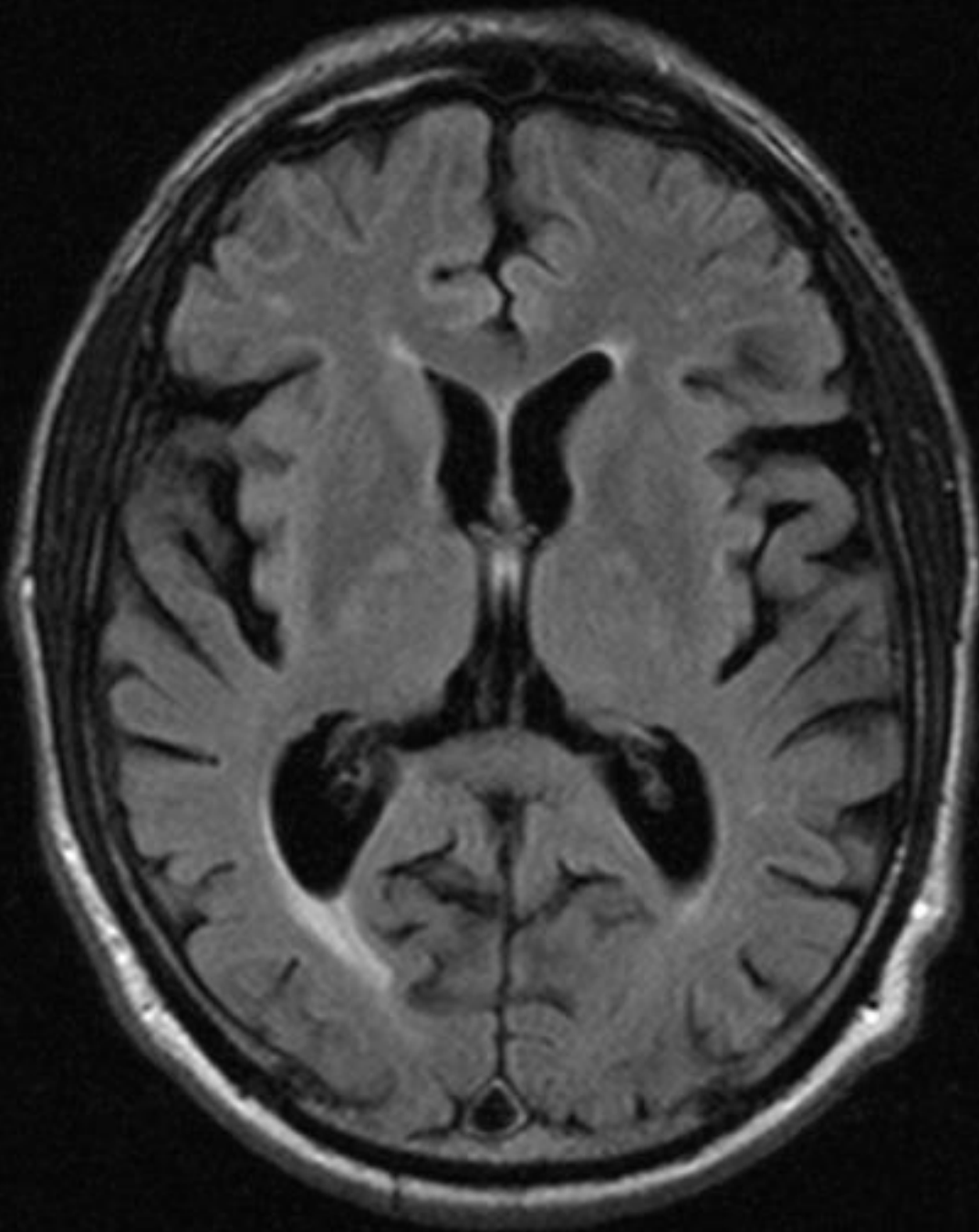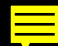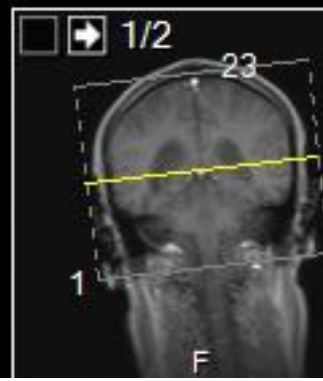

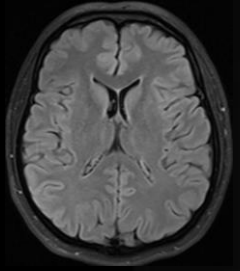

10

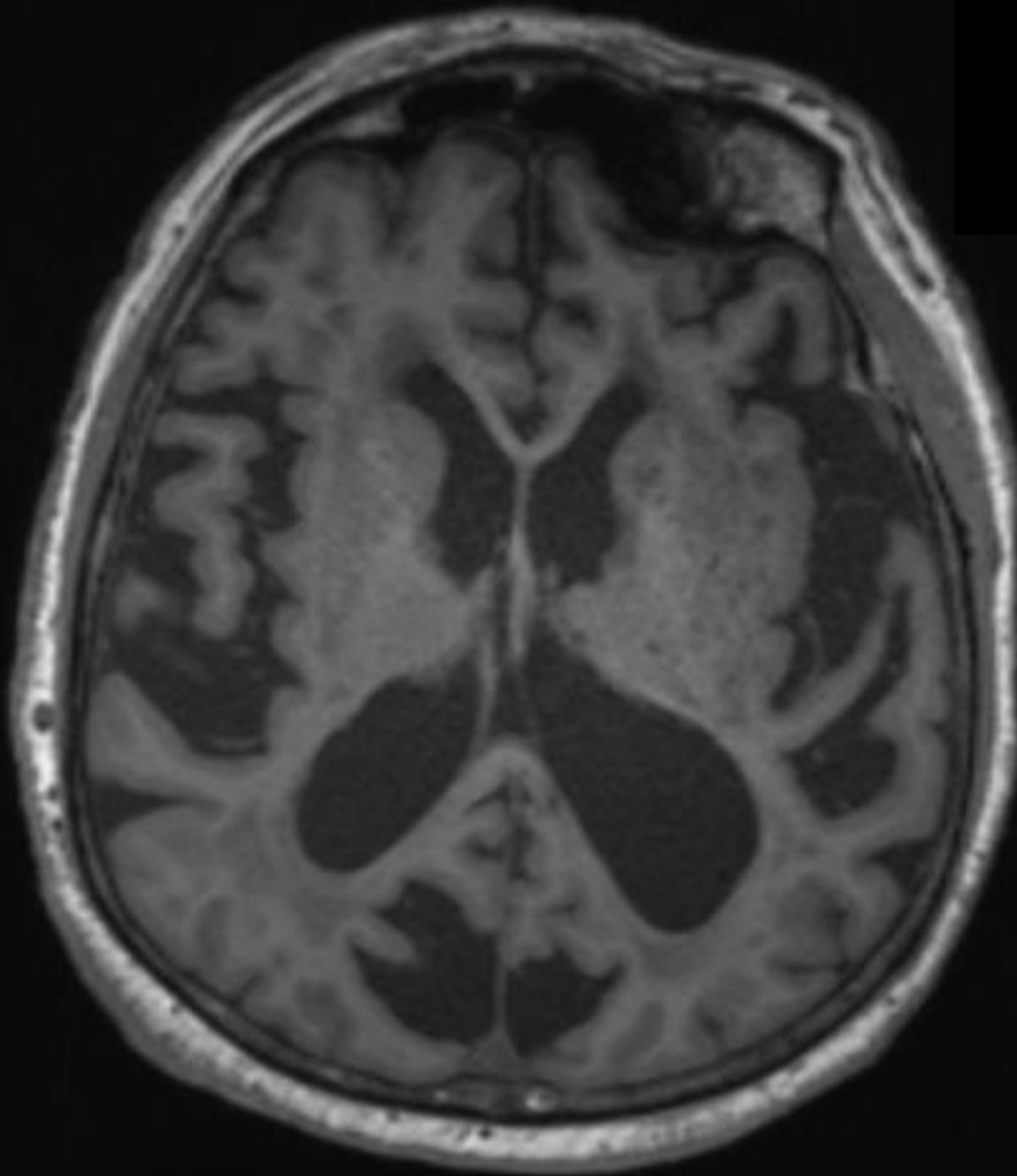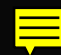

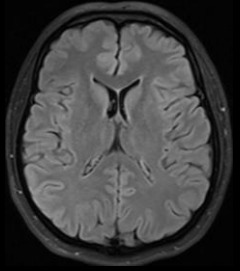

11

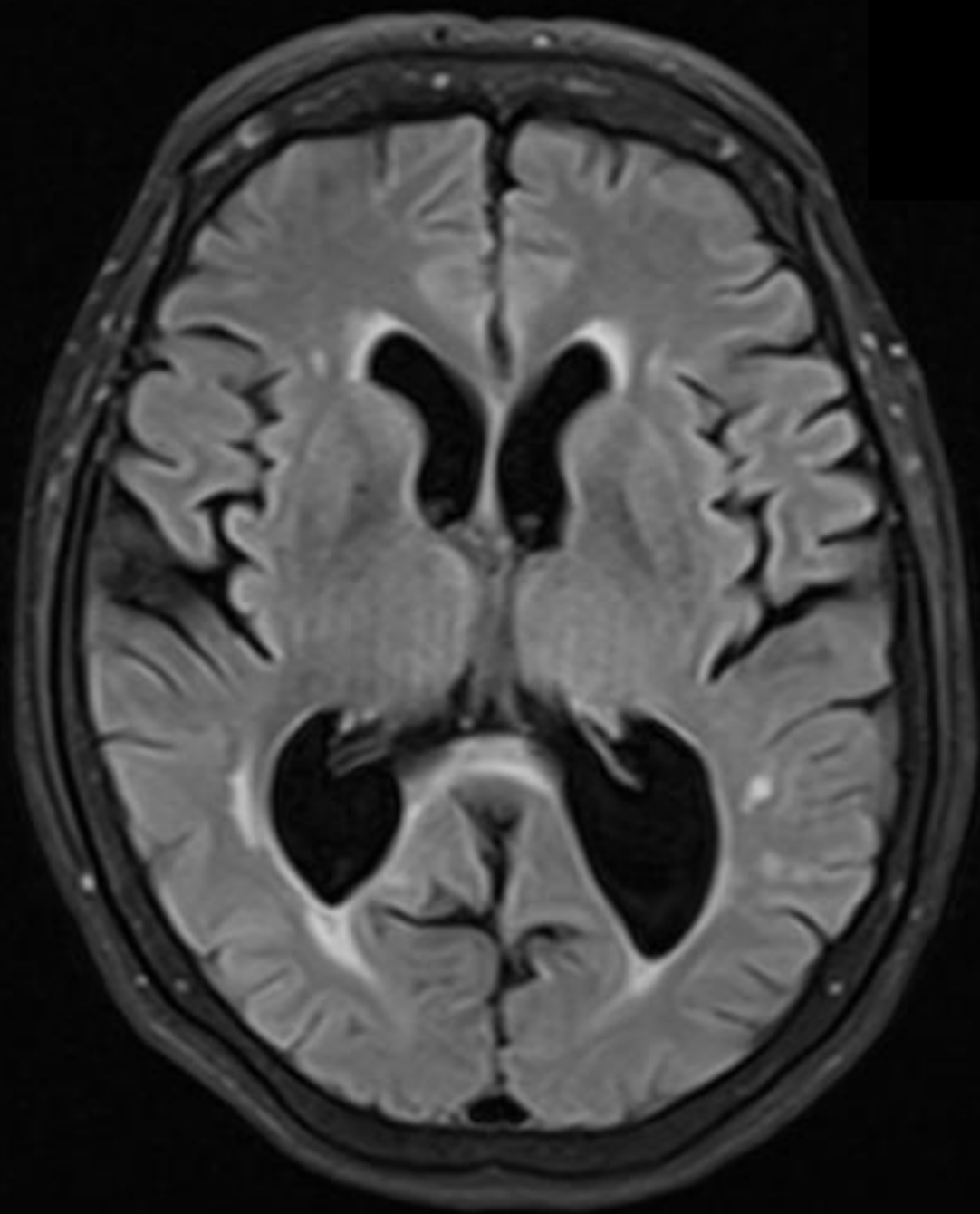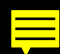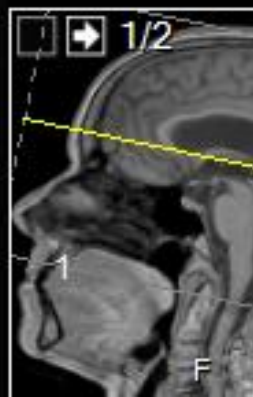

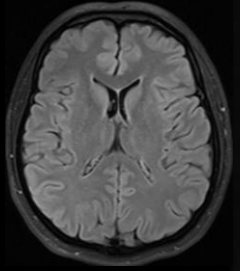

12

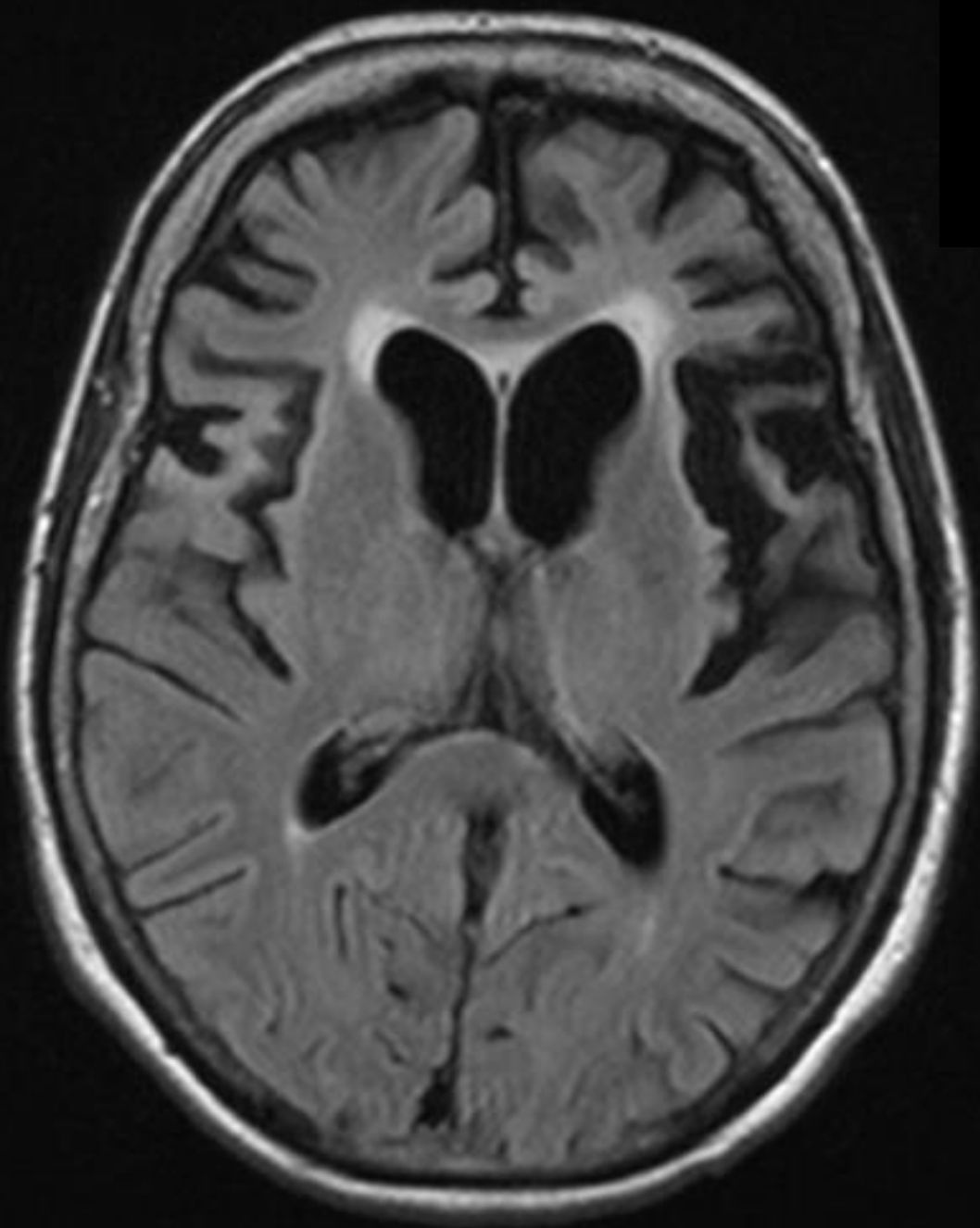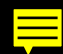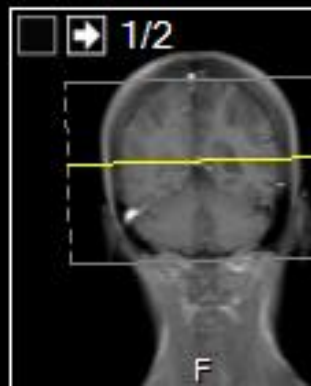

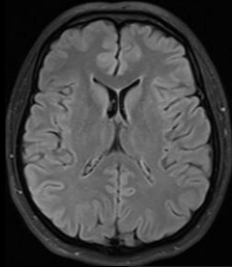

13

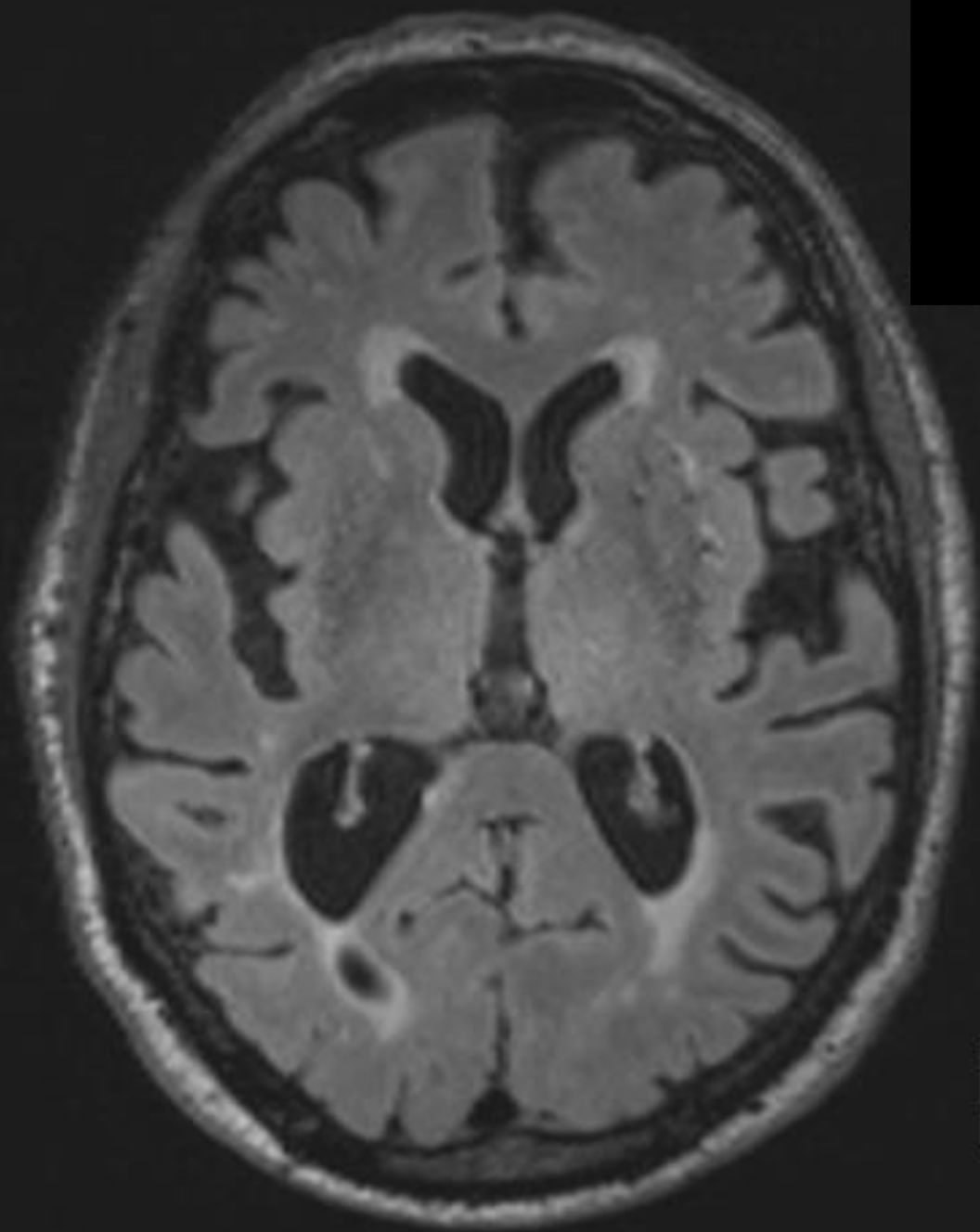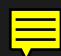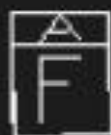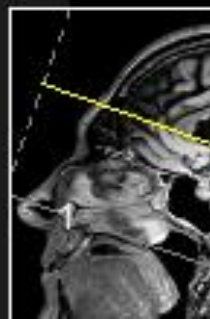

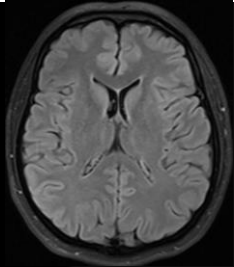

14

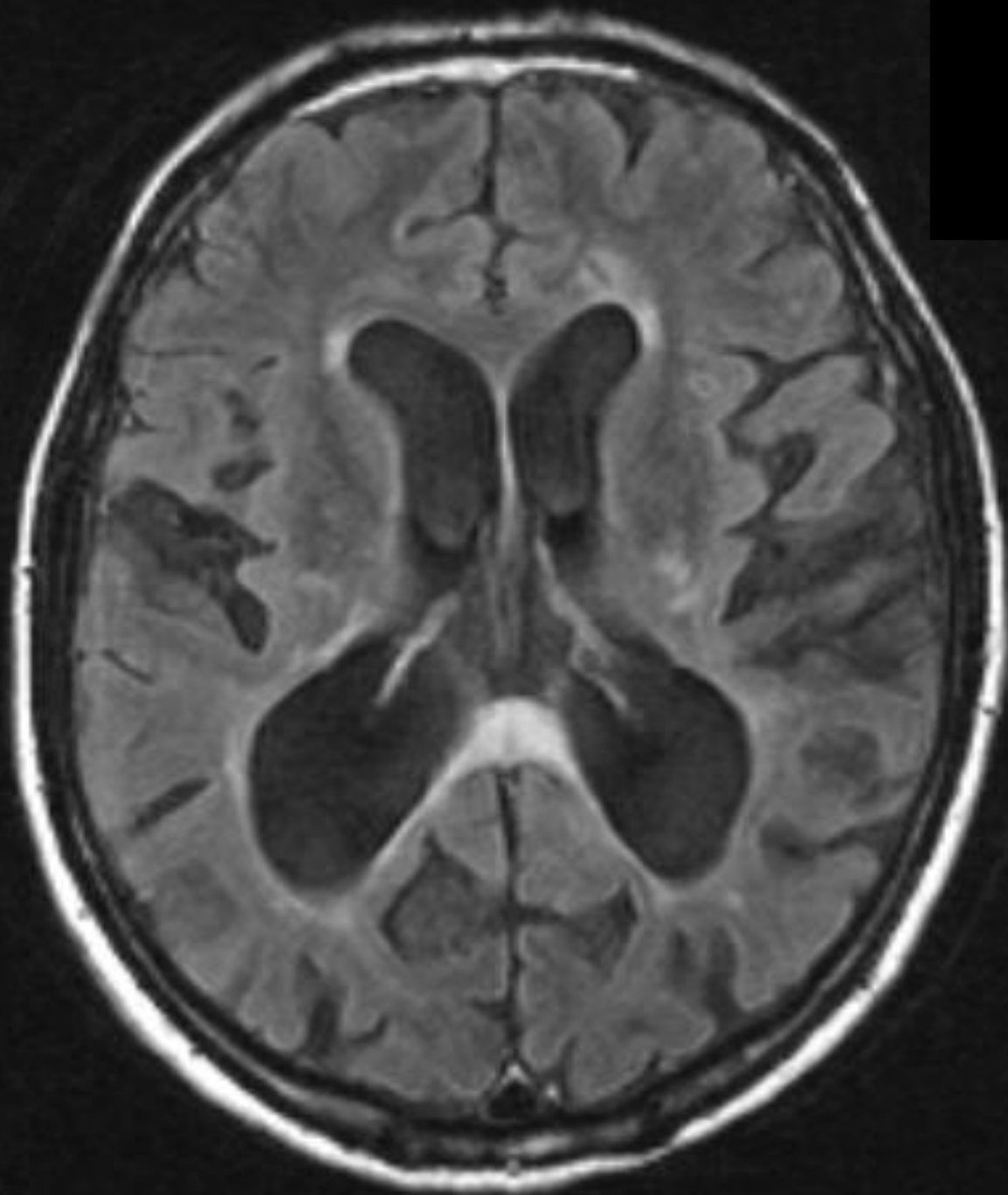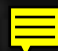

1/5

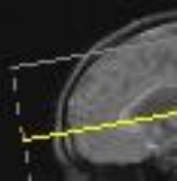

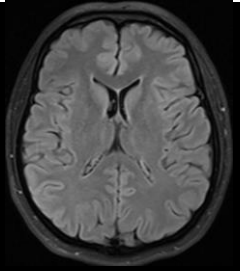

15

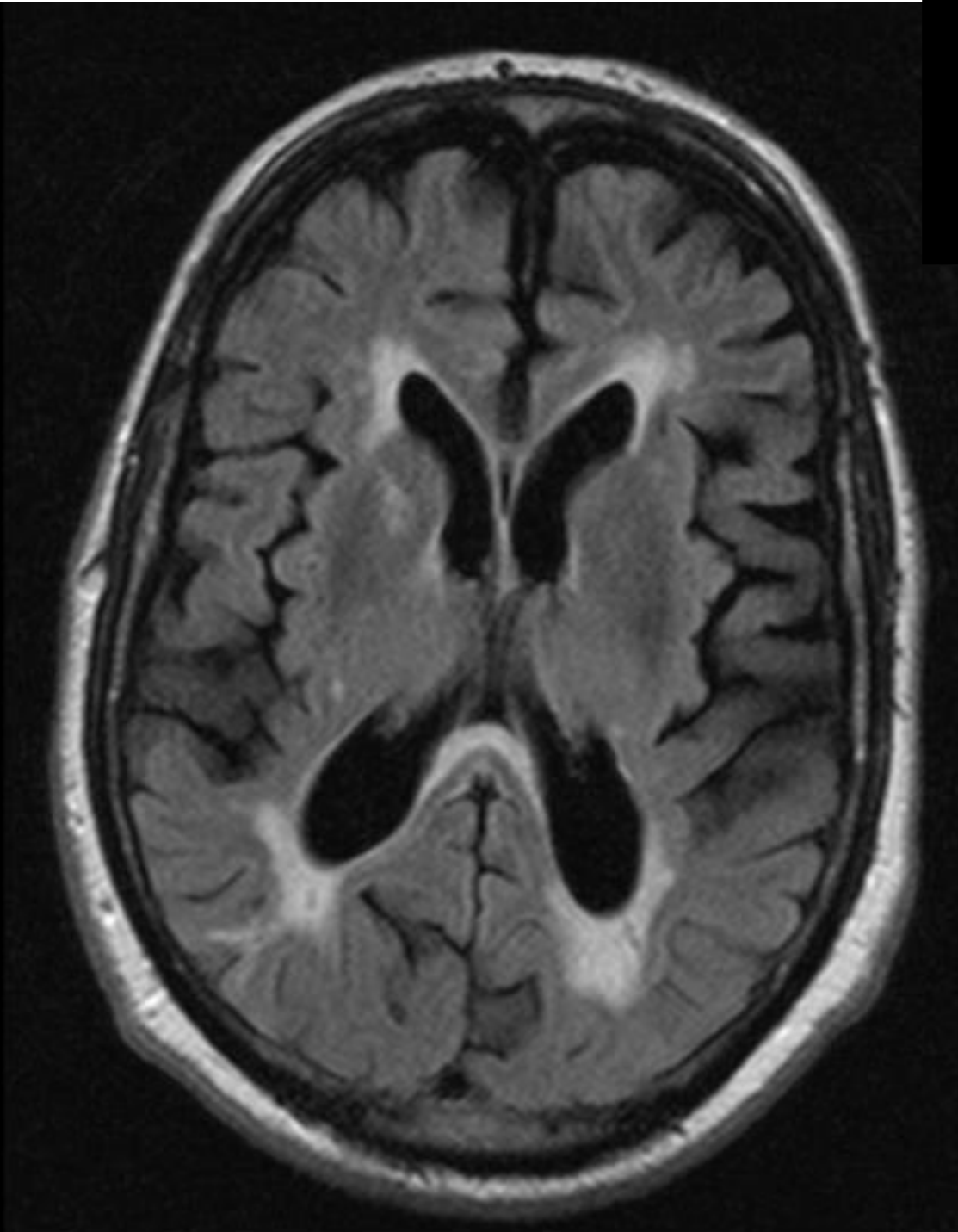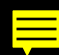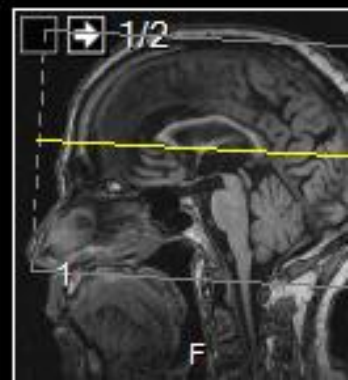

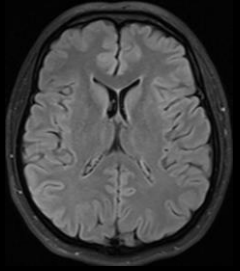

16

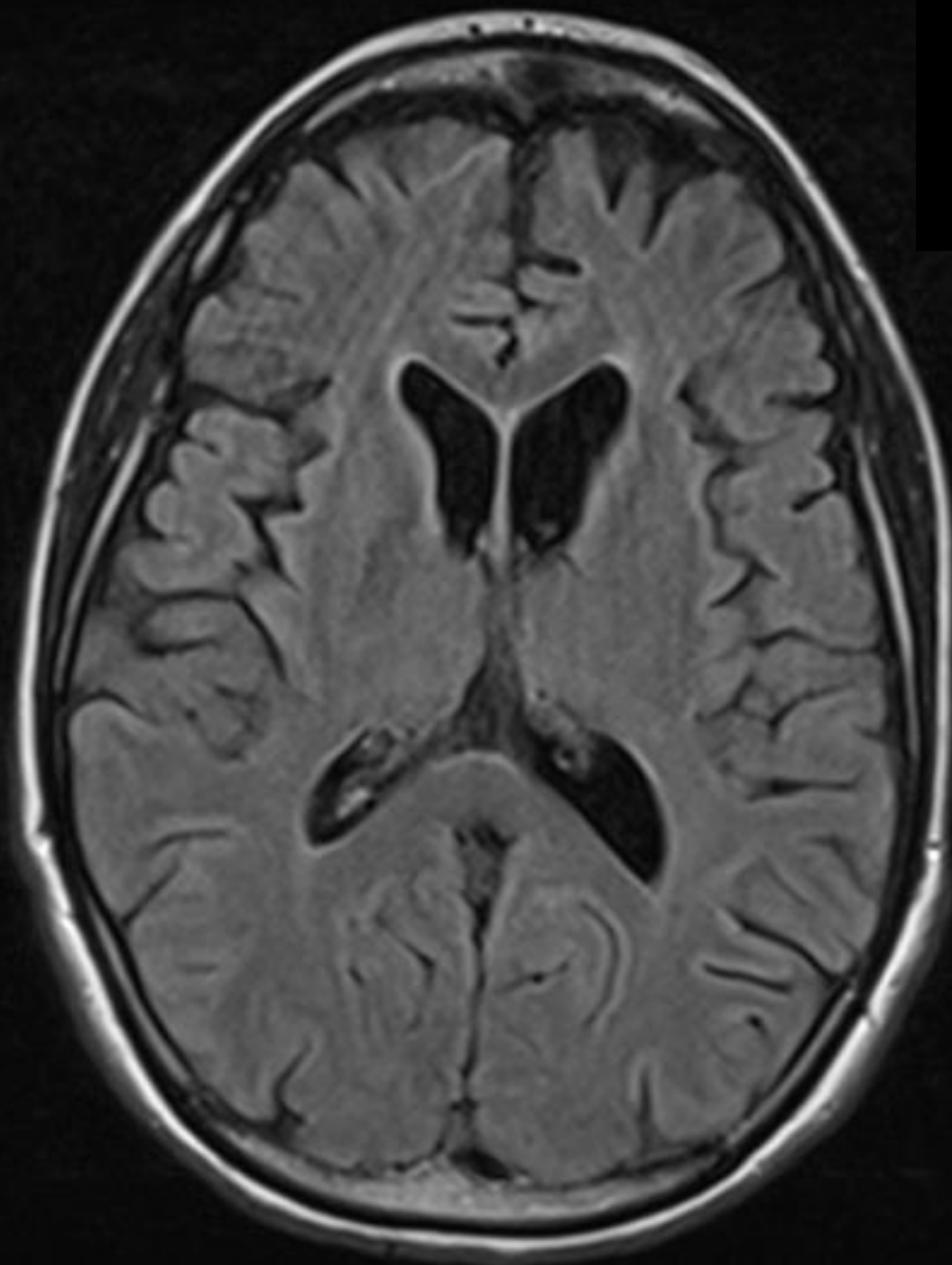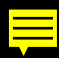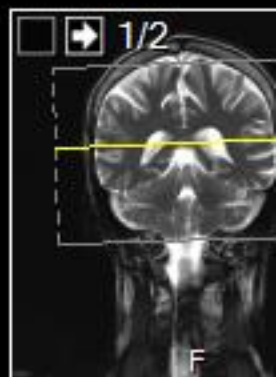

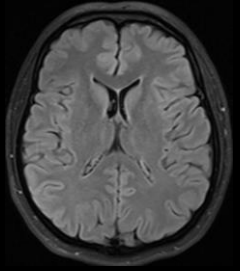

17

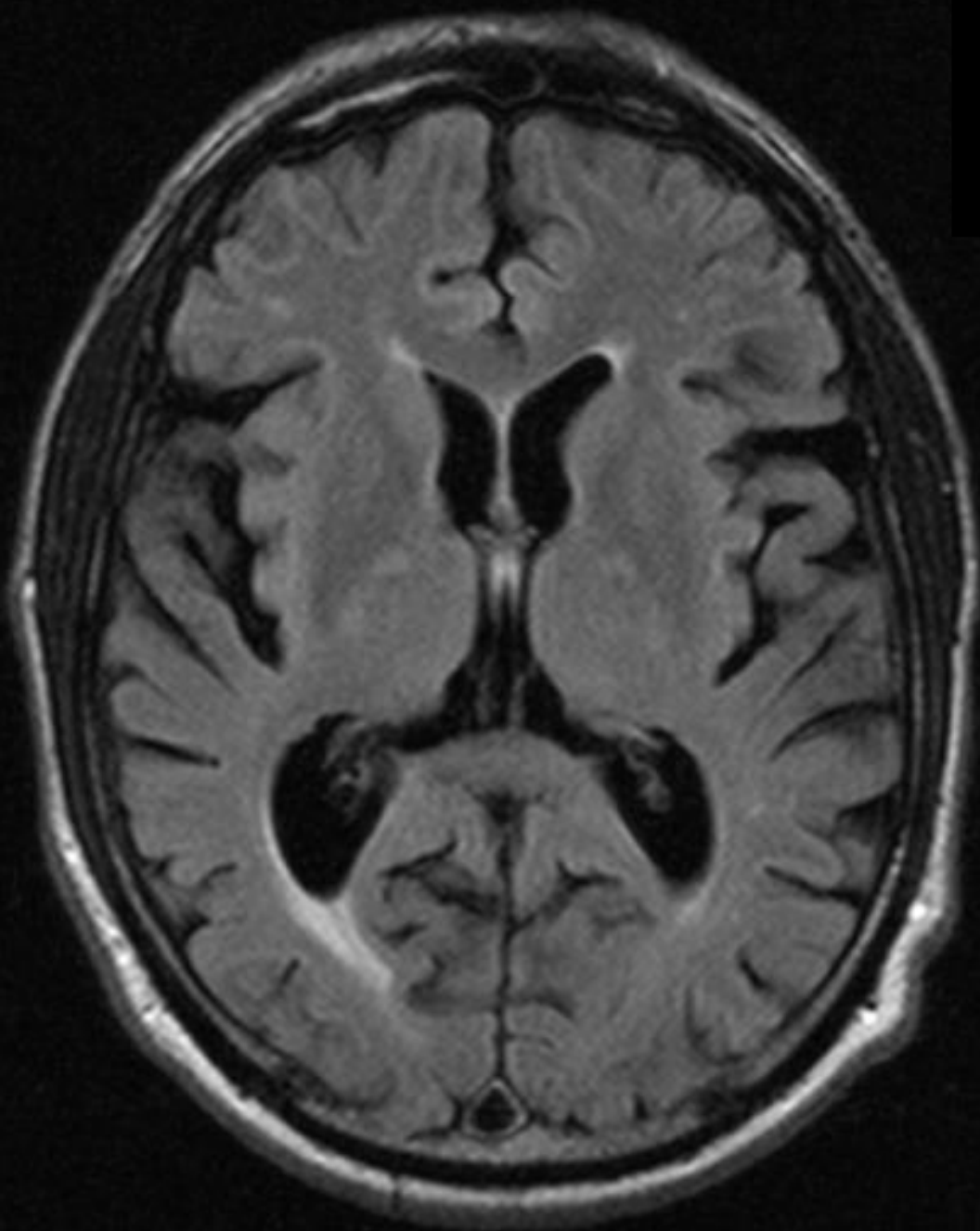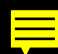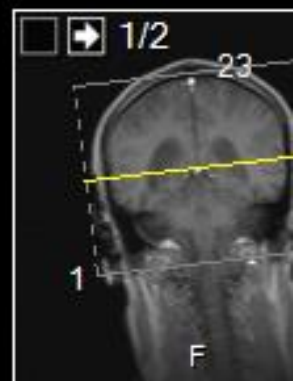

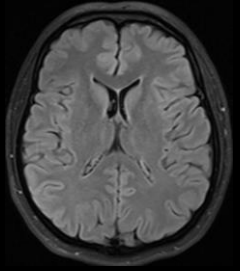

19

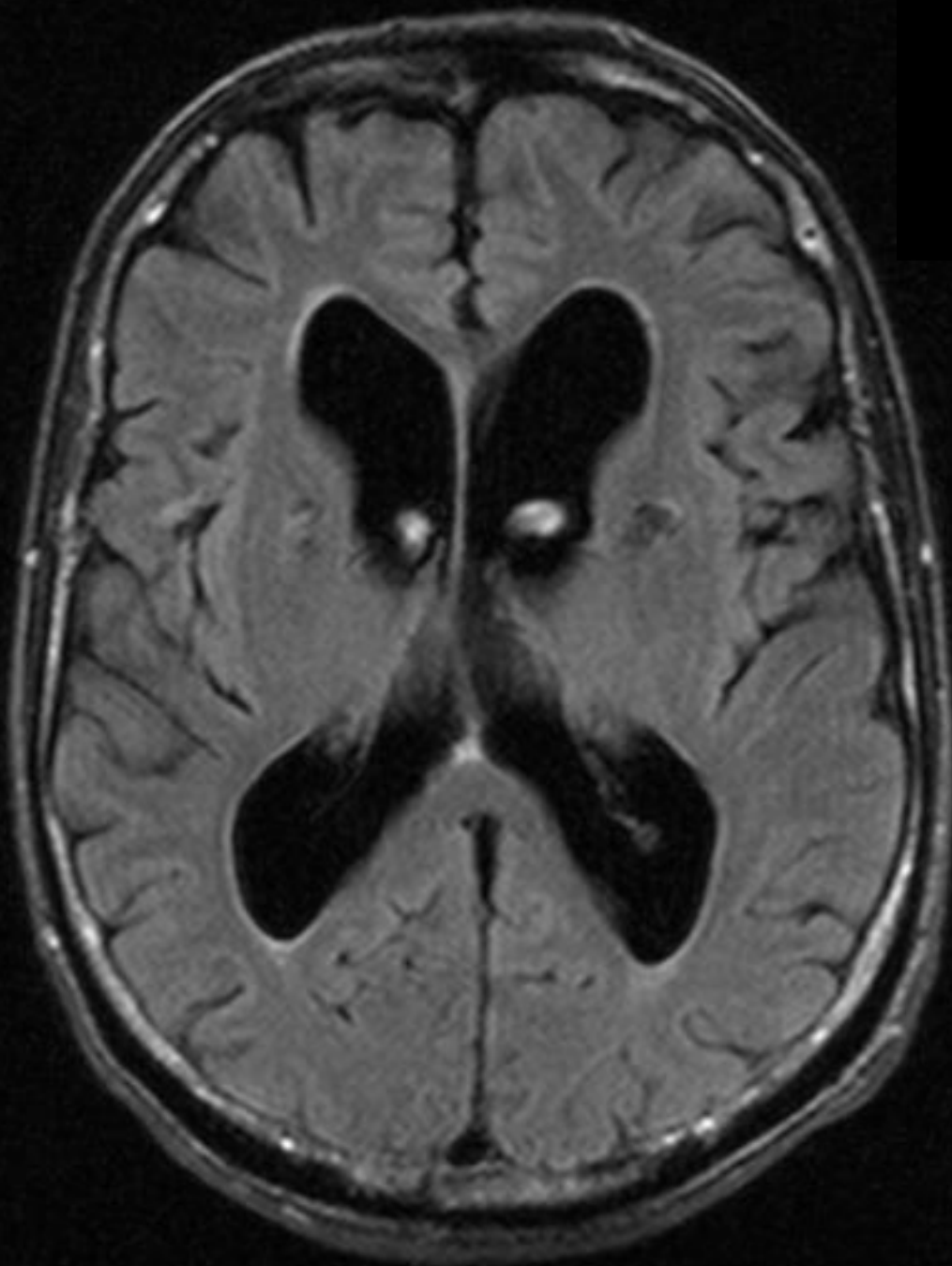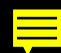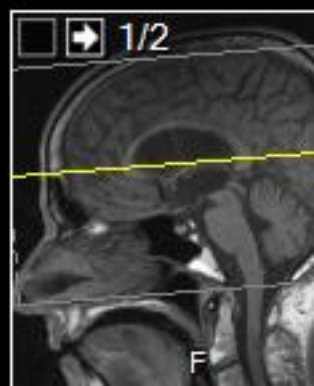

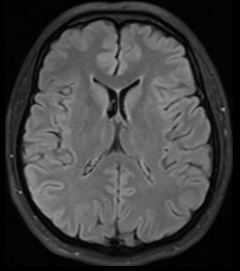

18

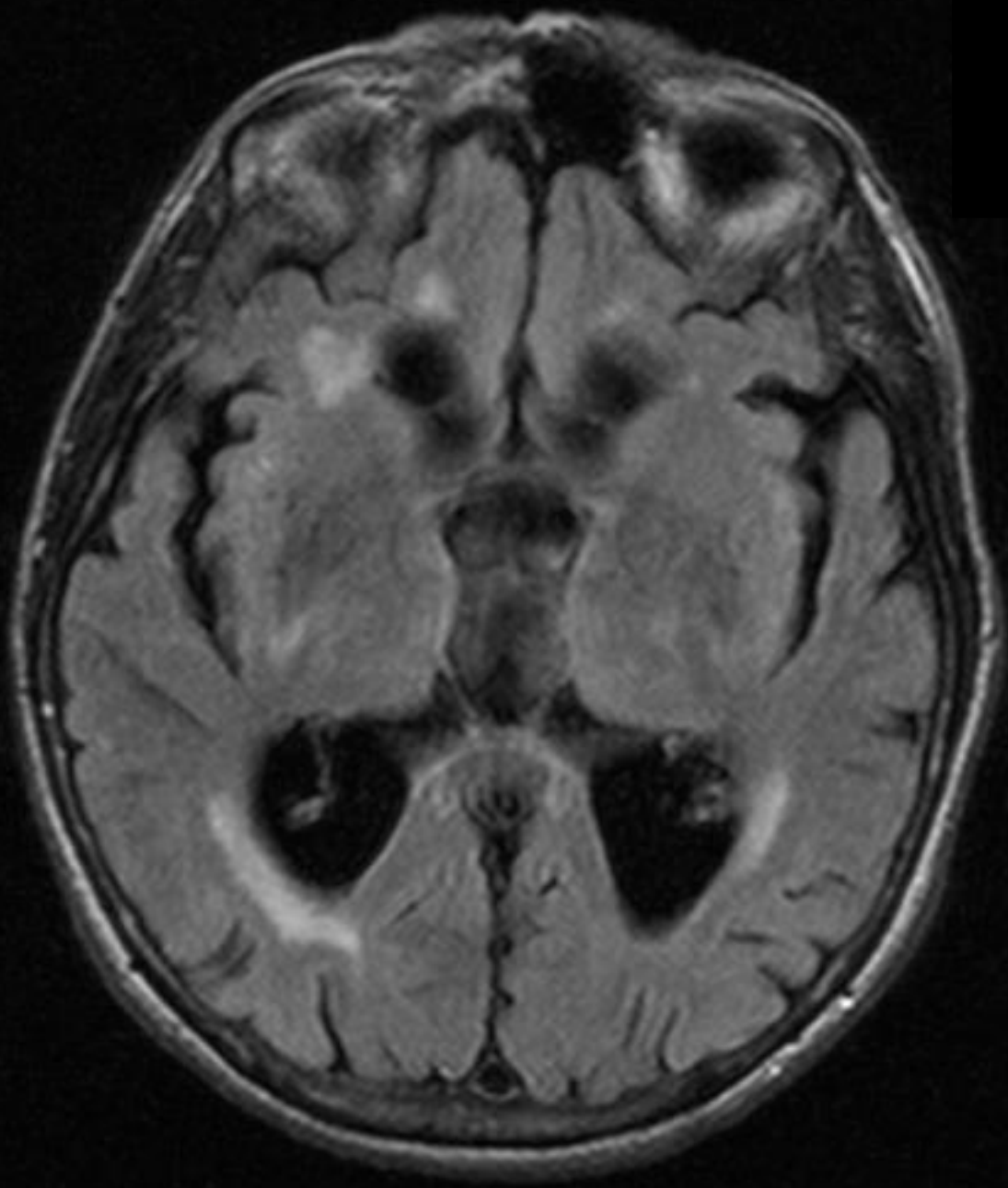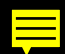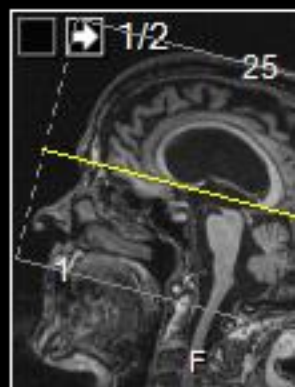

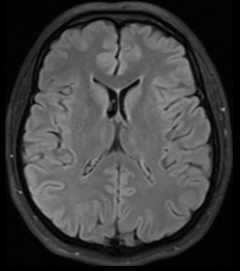

20

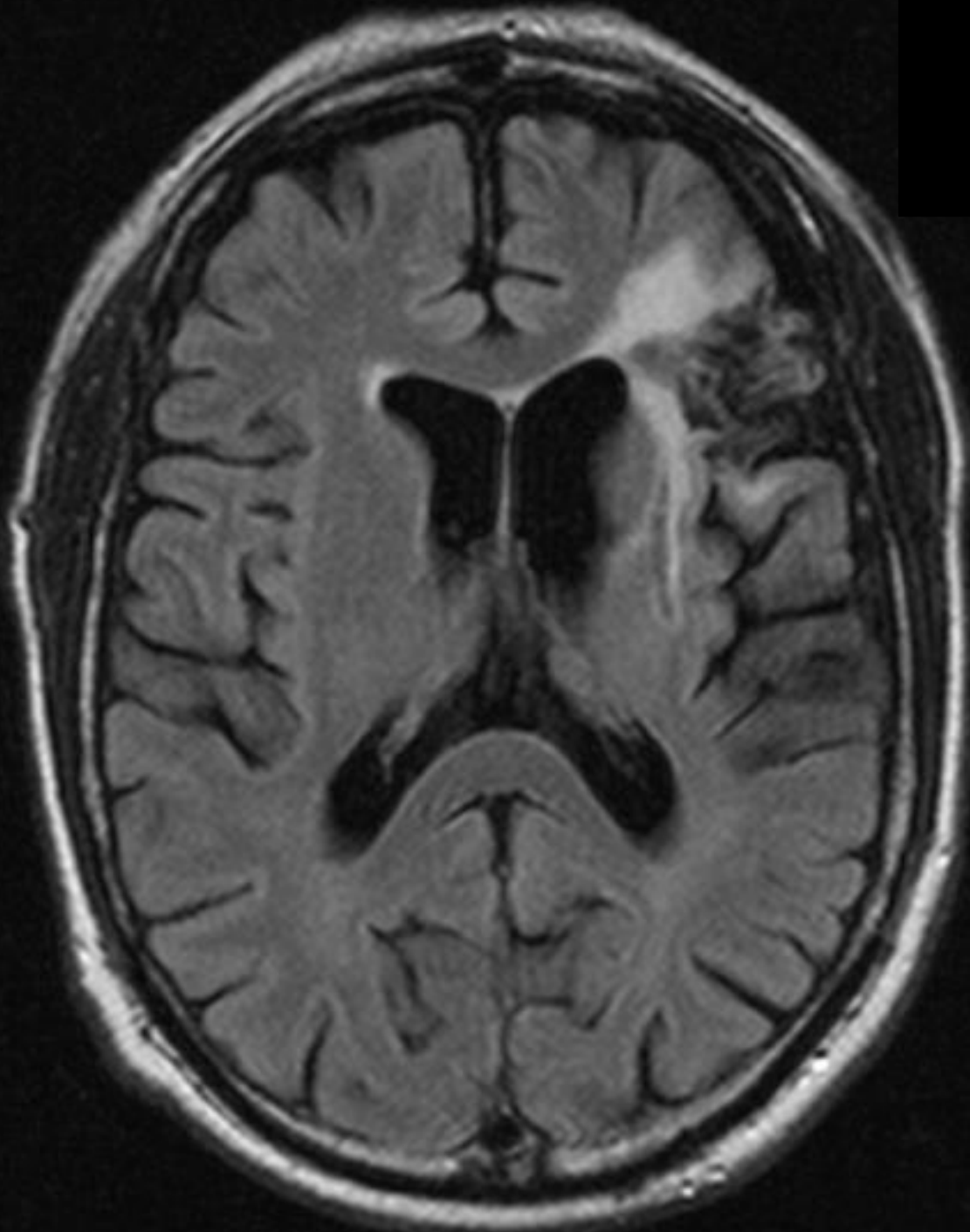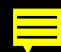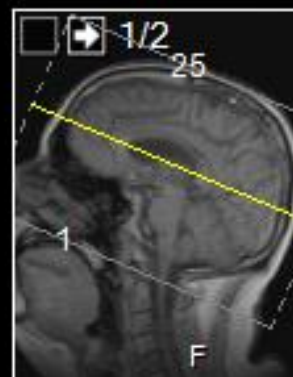

Supplement: Supplementary file 1 — (PDF 846 kb) [file 13244_2016_521_MOESM1_ESM.pdf]

Healthy, 25 years of age

1

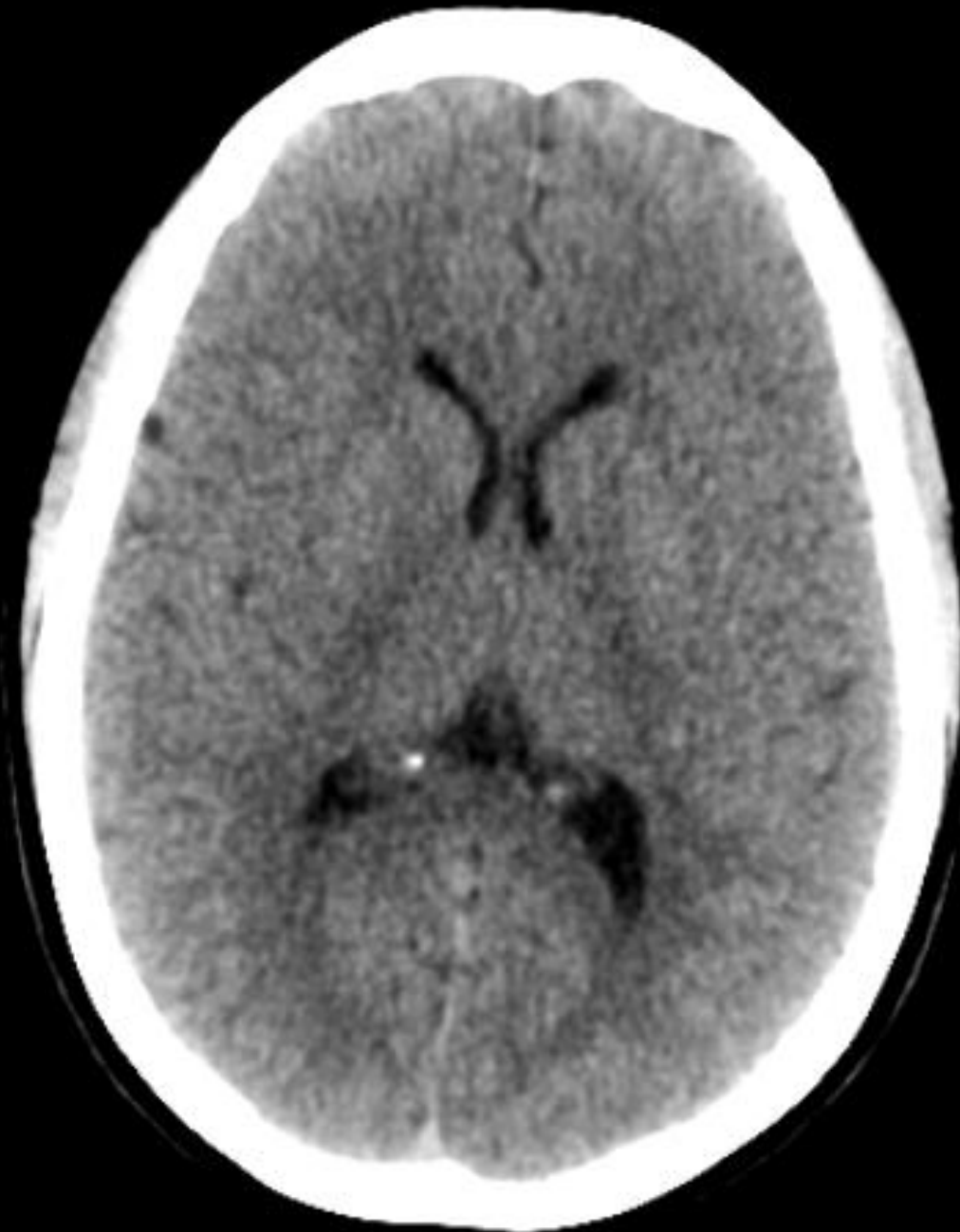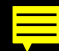

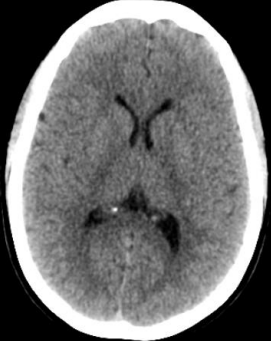

2

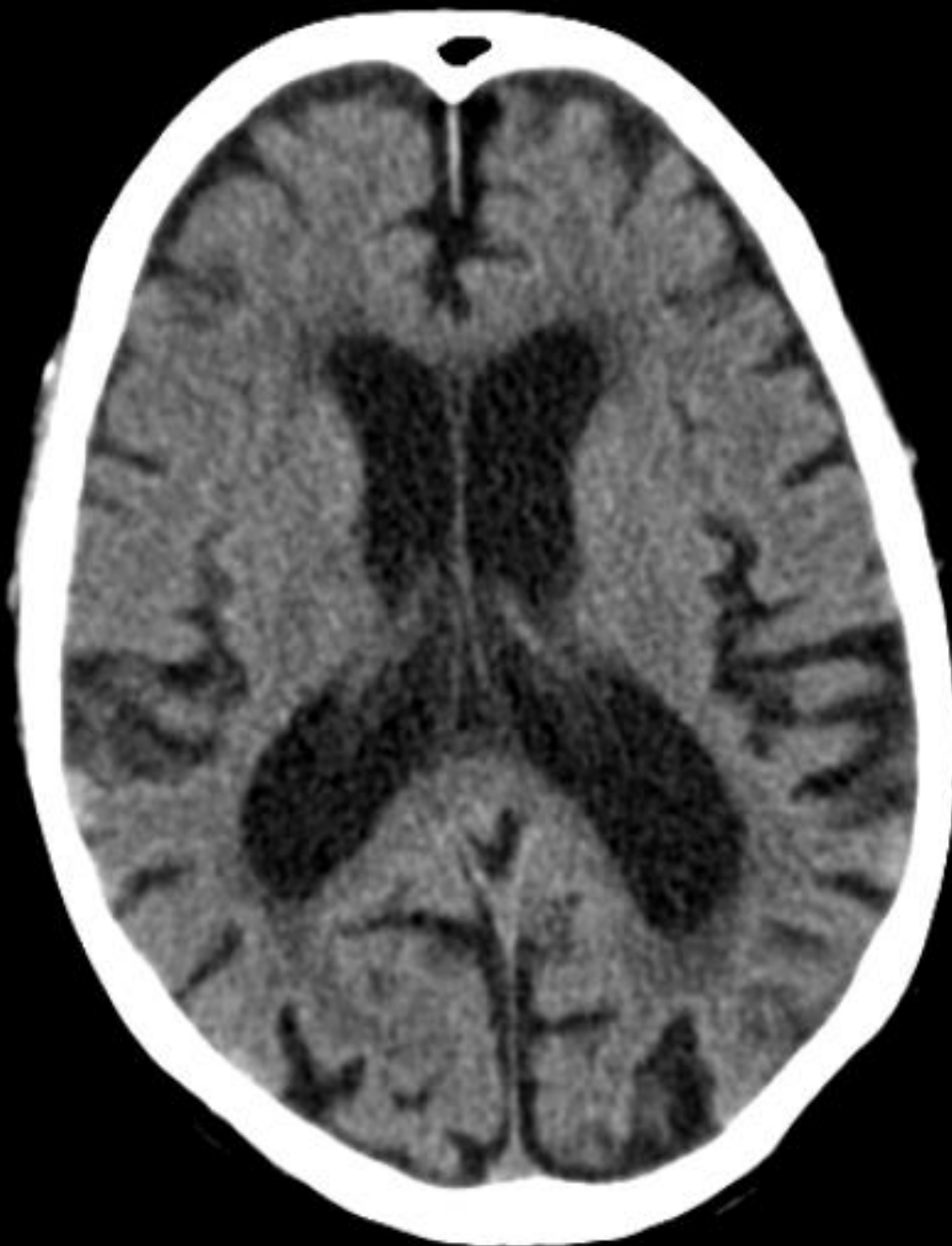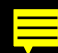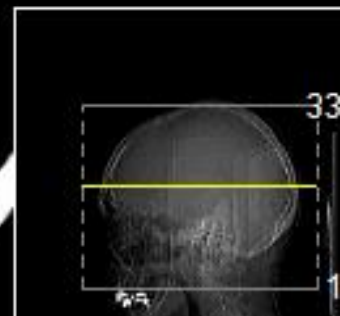

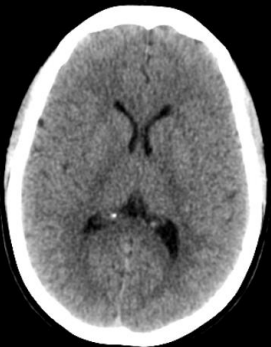

3

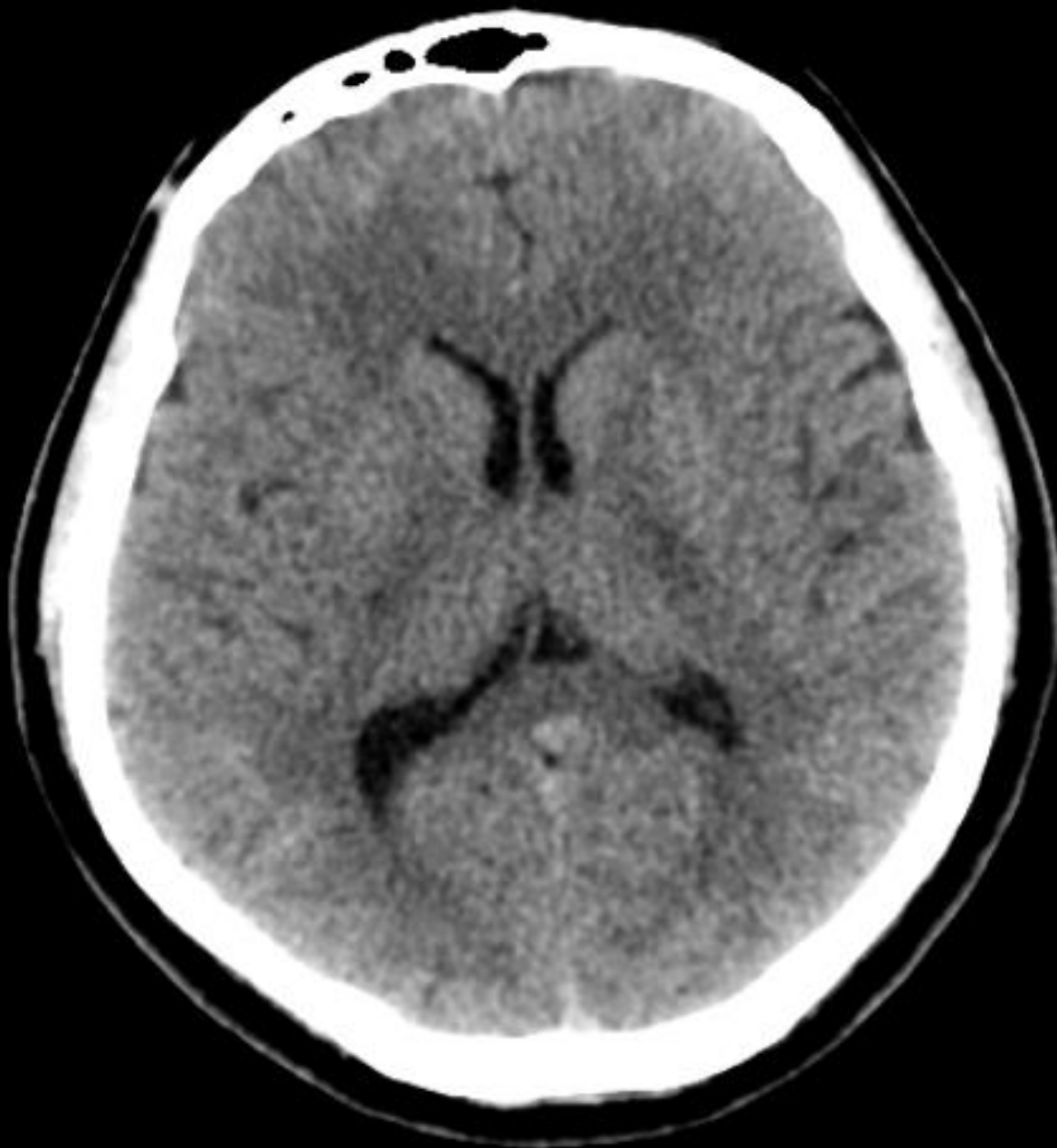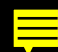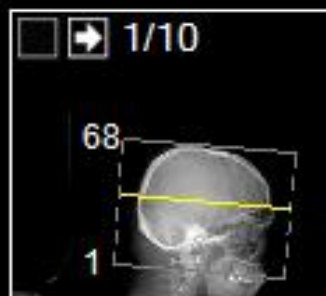

F

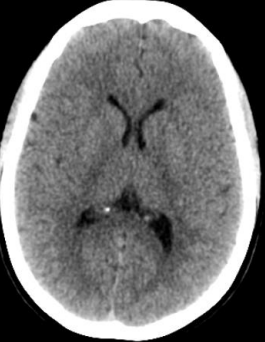

4

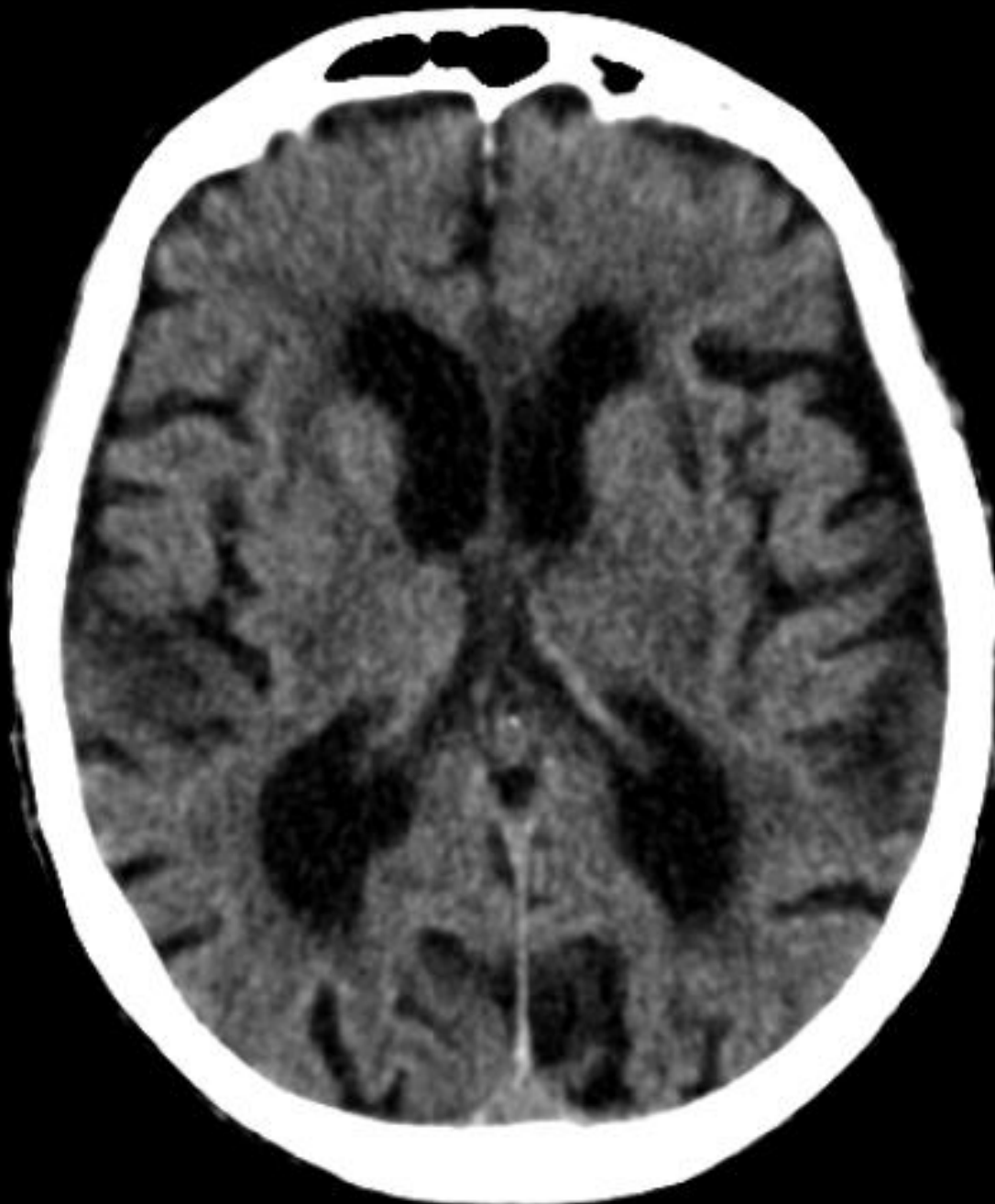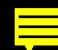

1/10

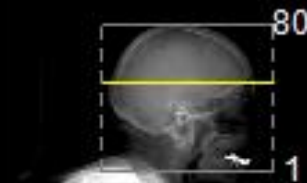

F

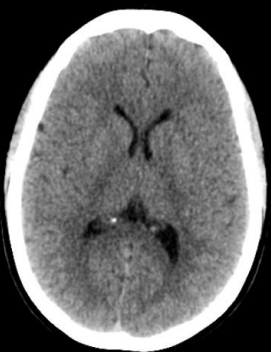

5

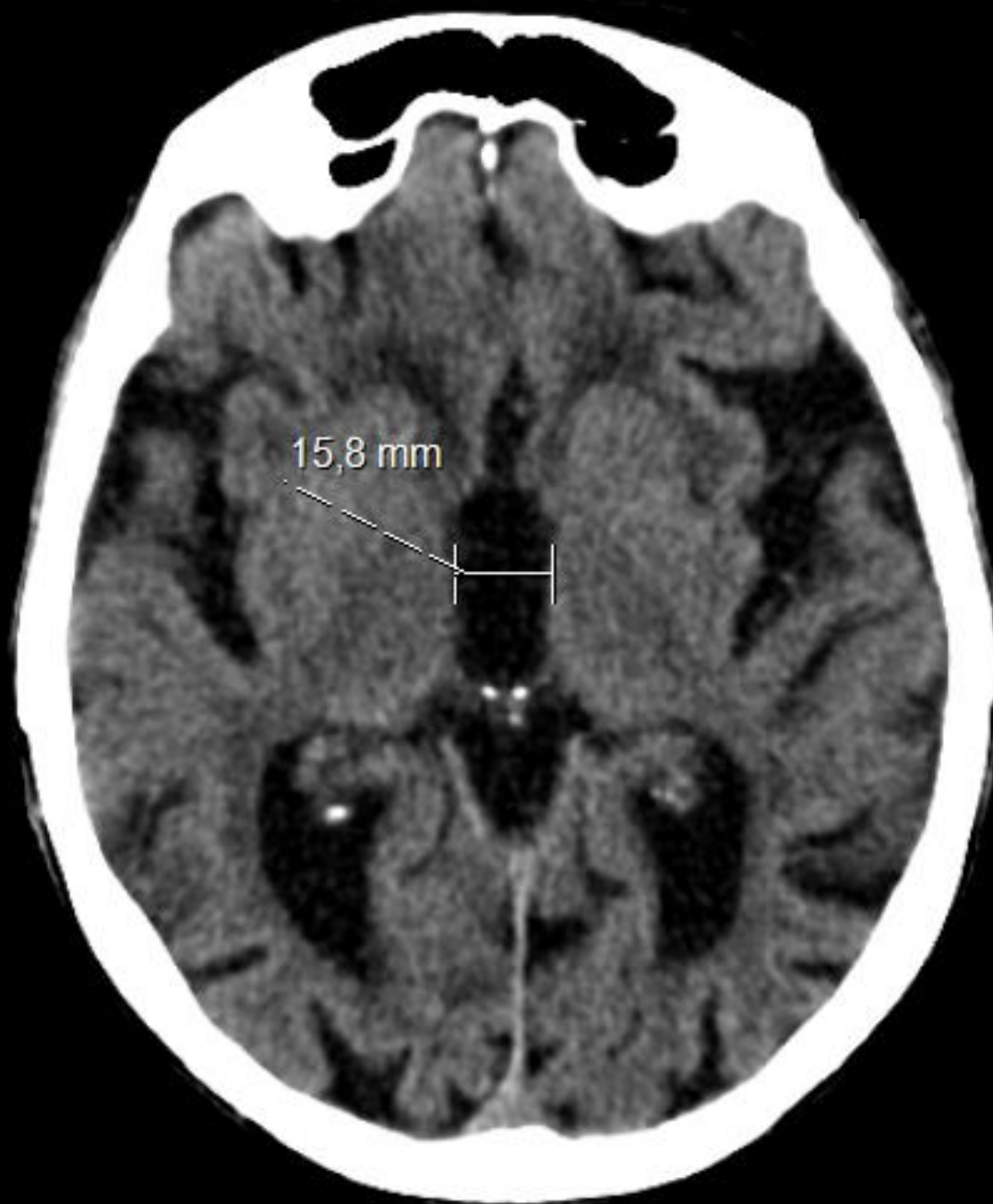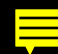

1/10

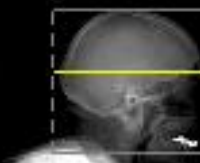

F

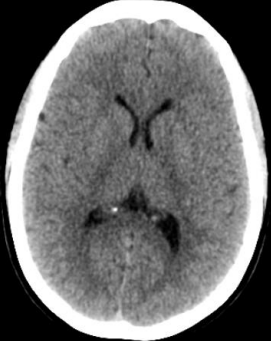

6

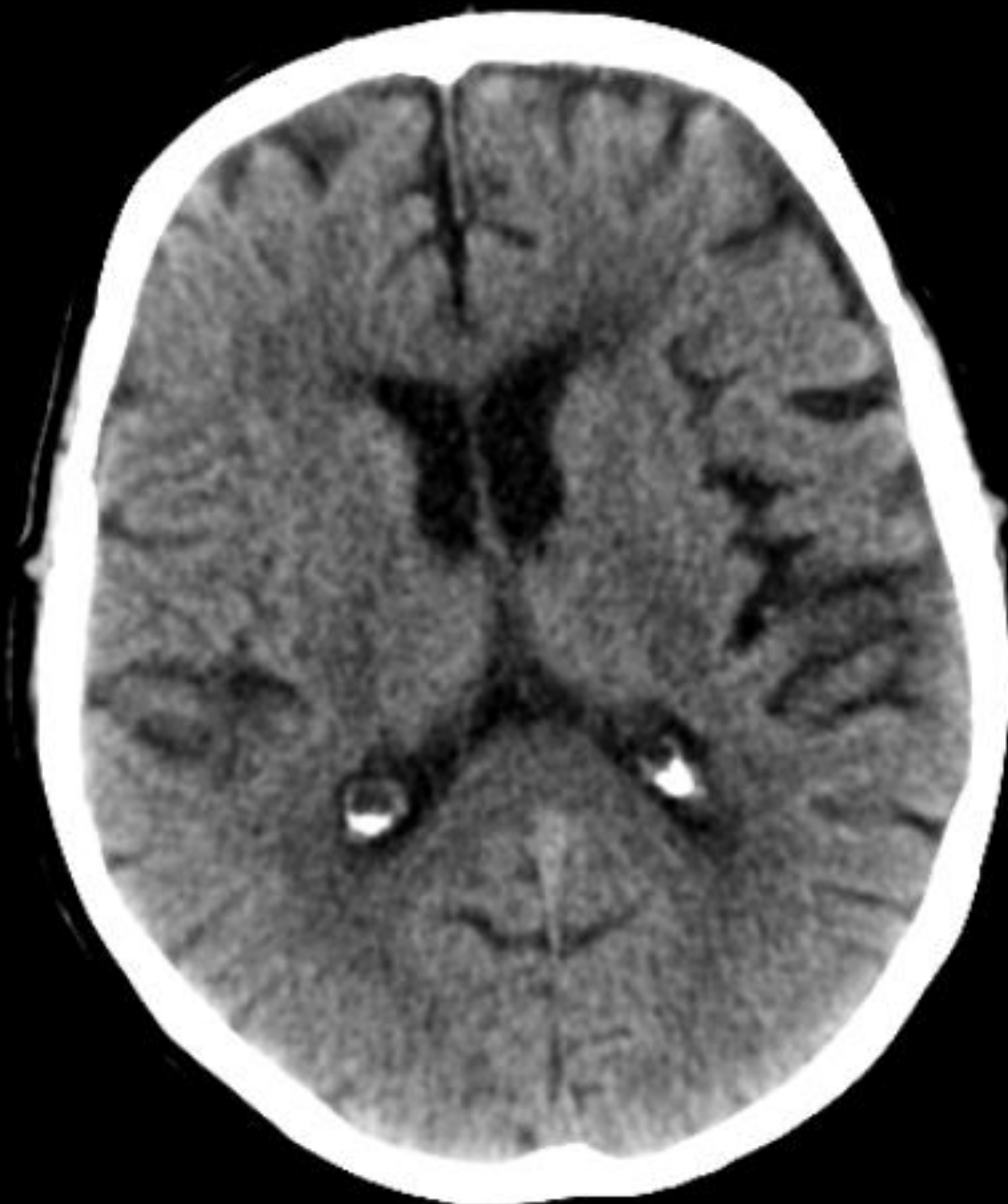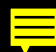

1/5

72

1

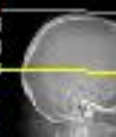

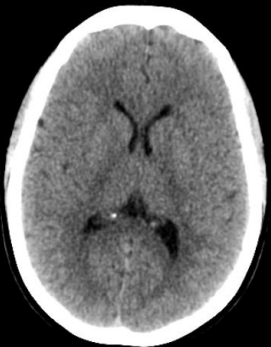

7

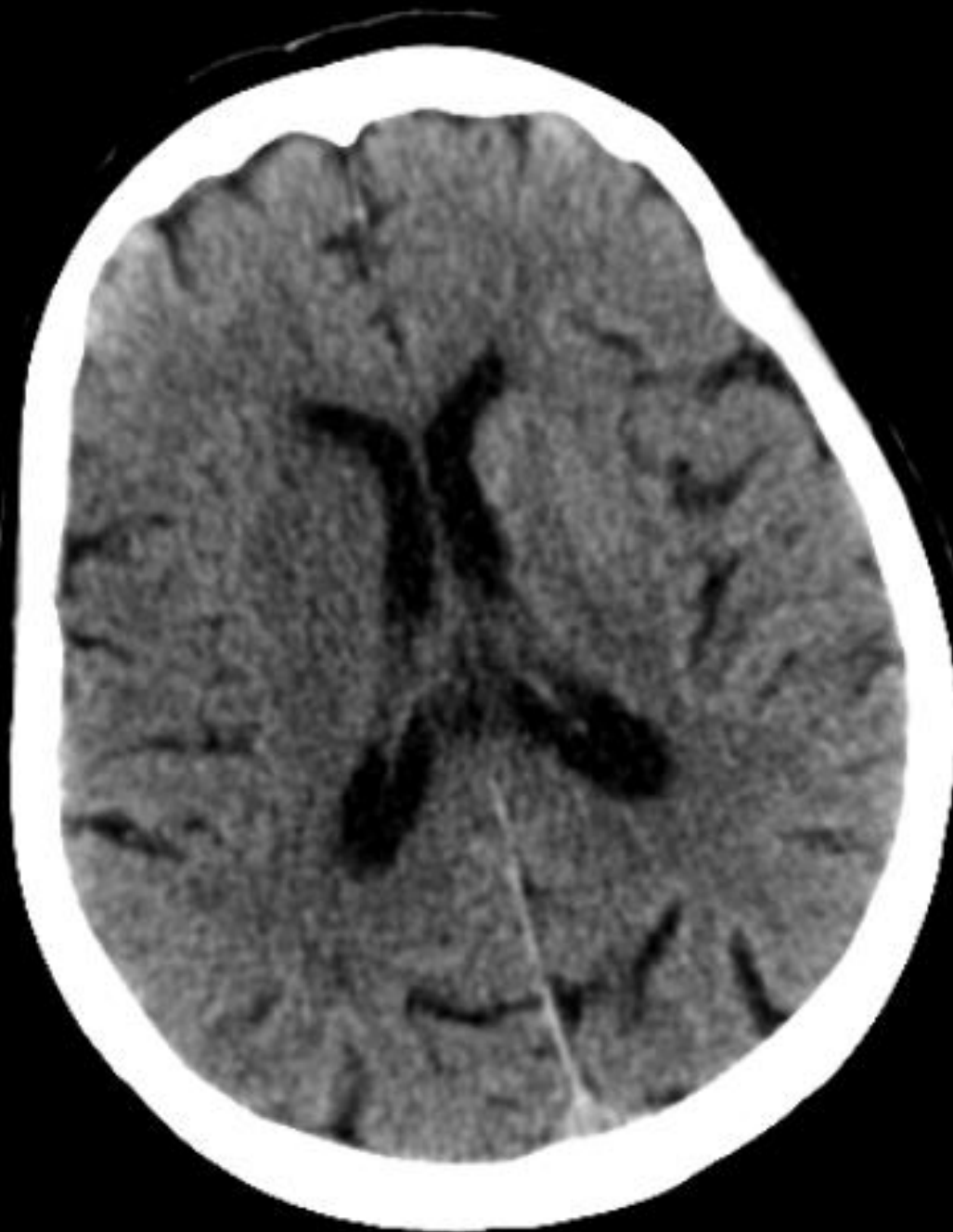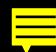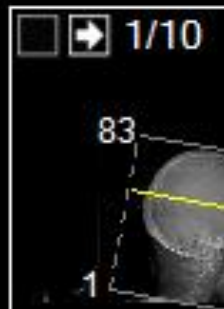

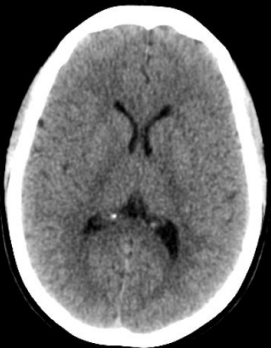

8

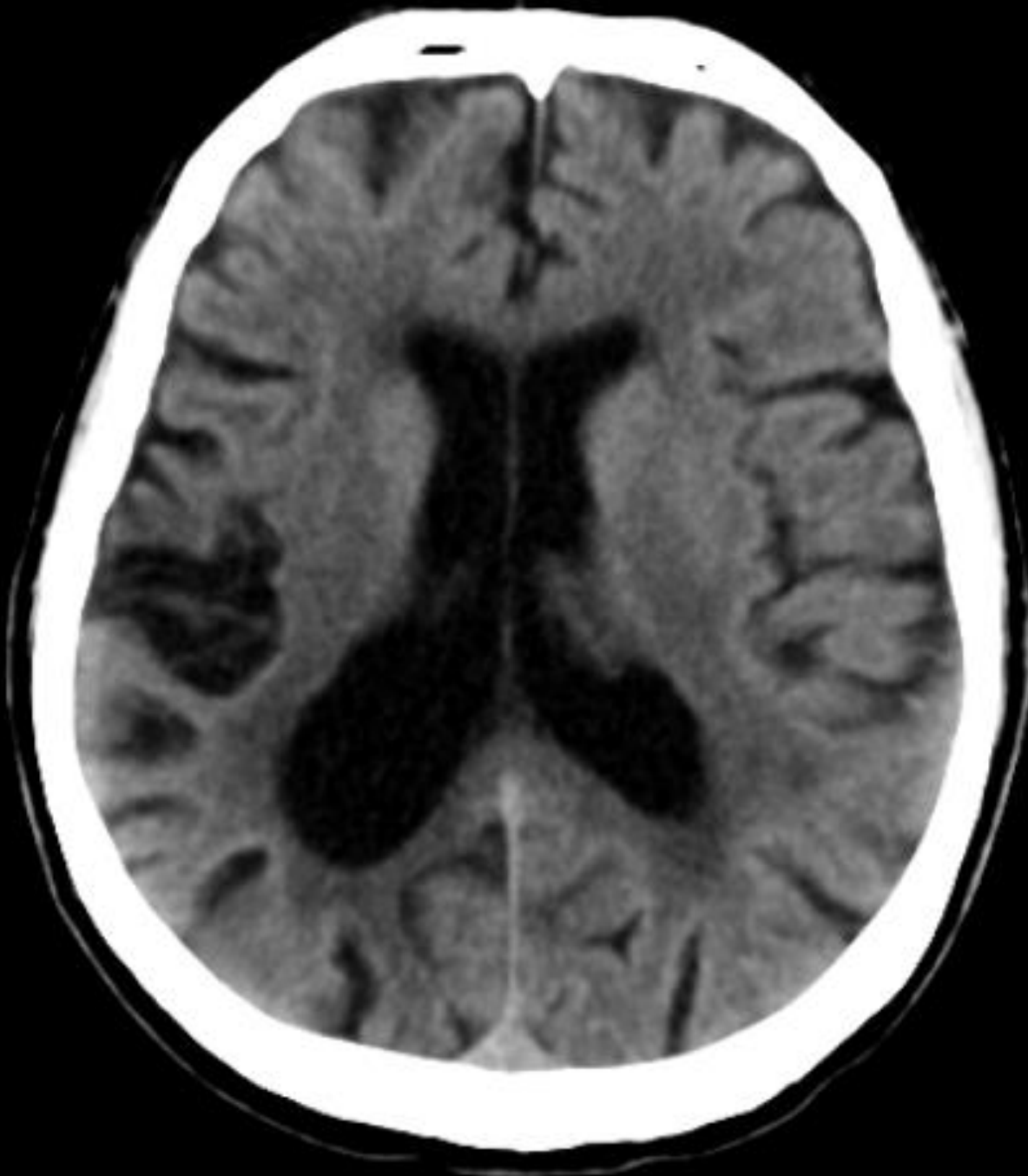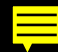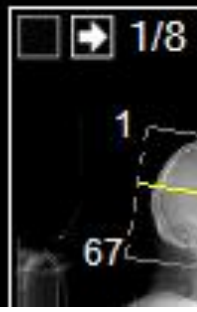

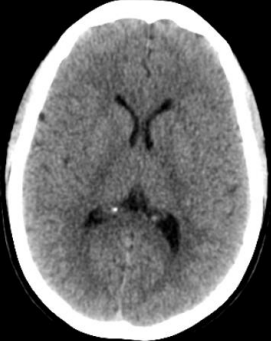

9

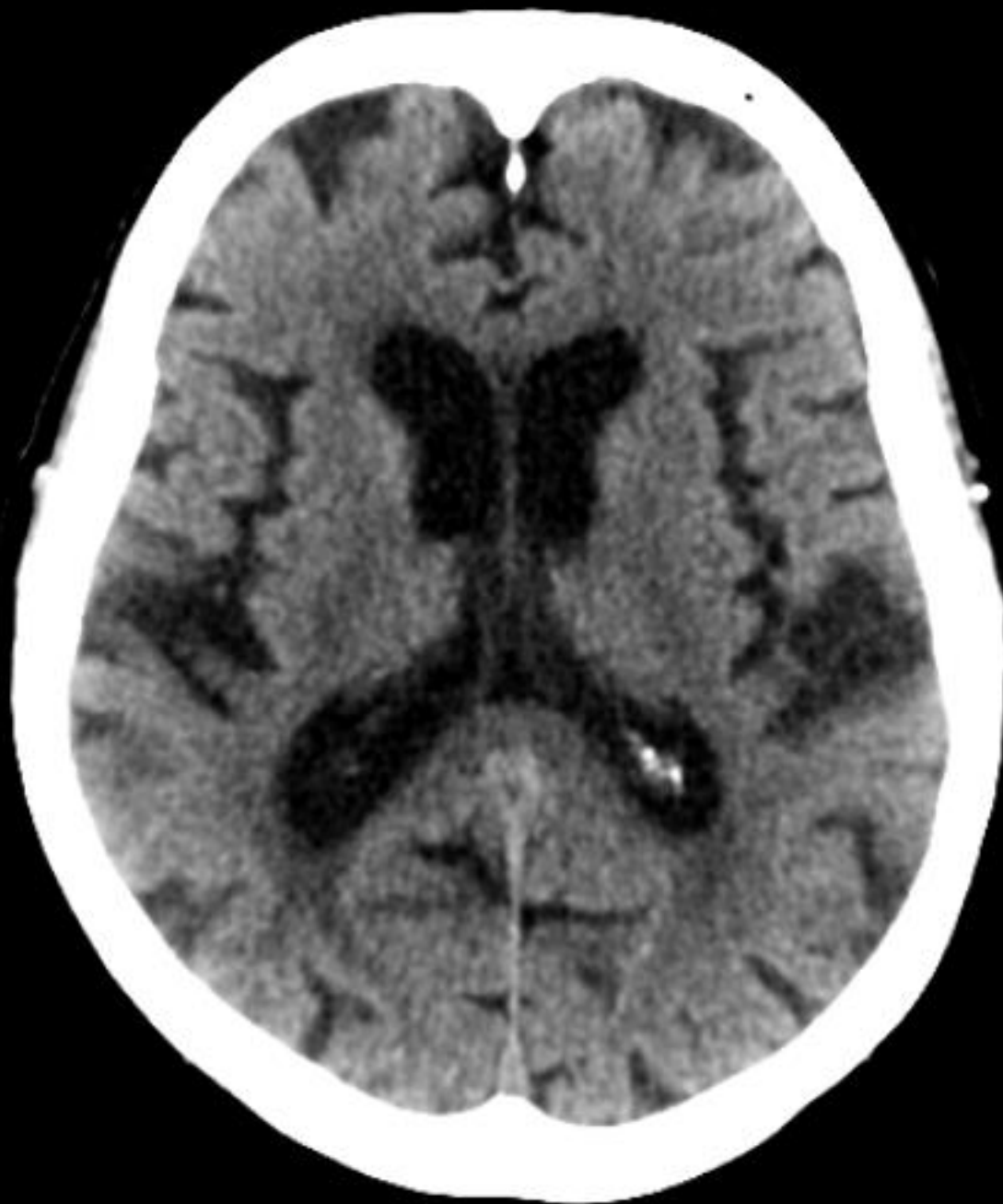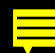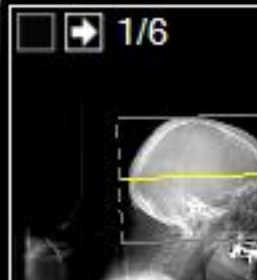

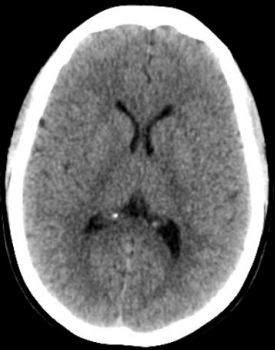

10

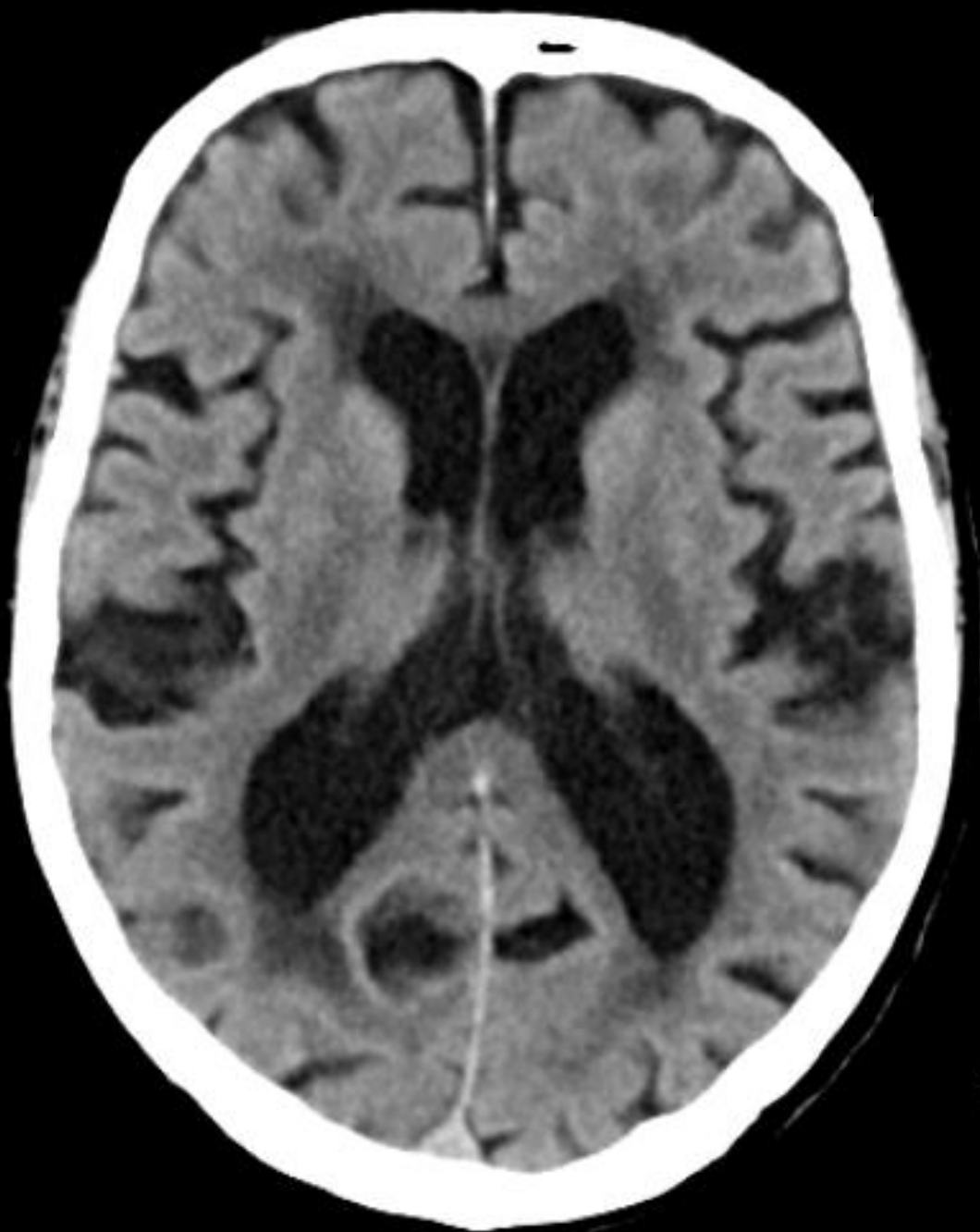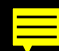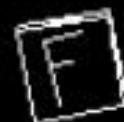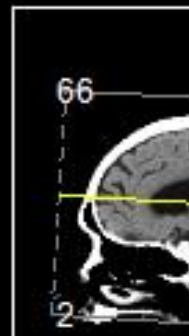

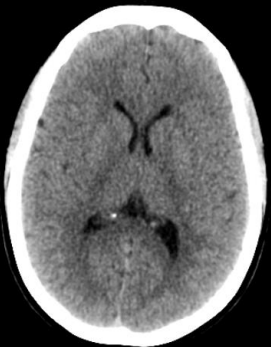

11

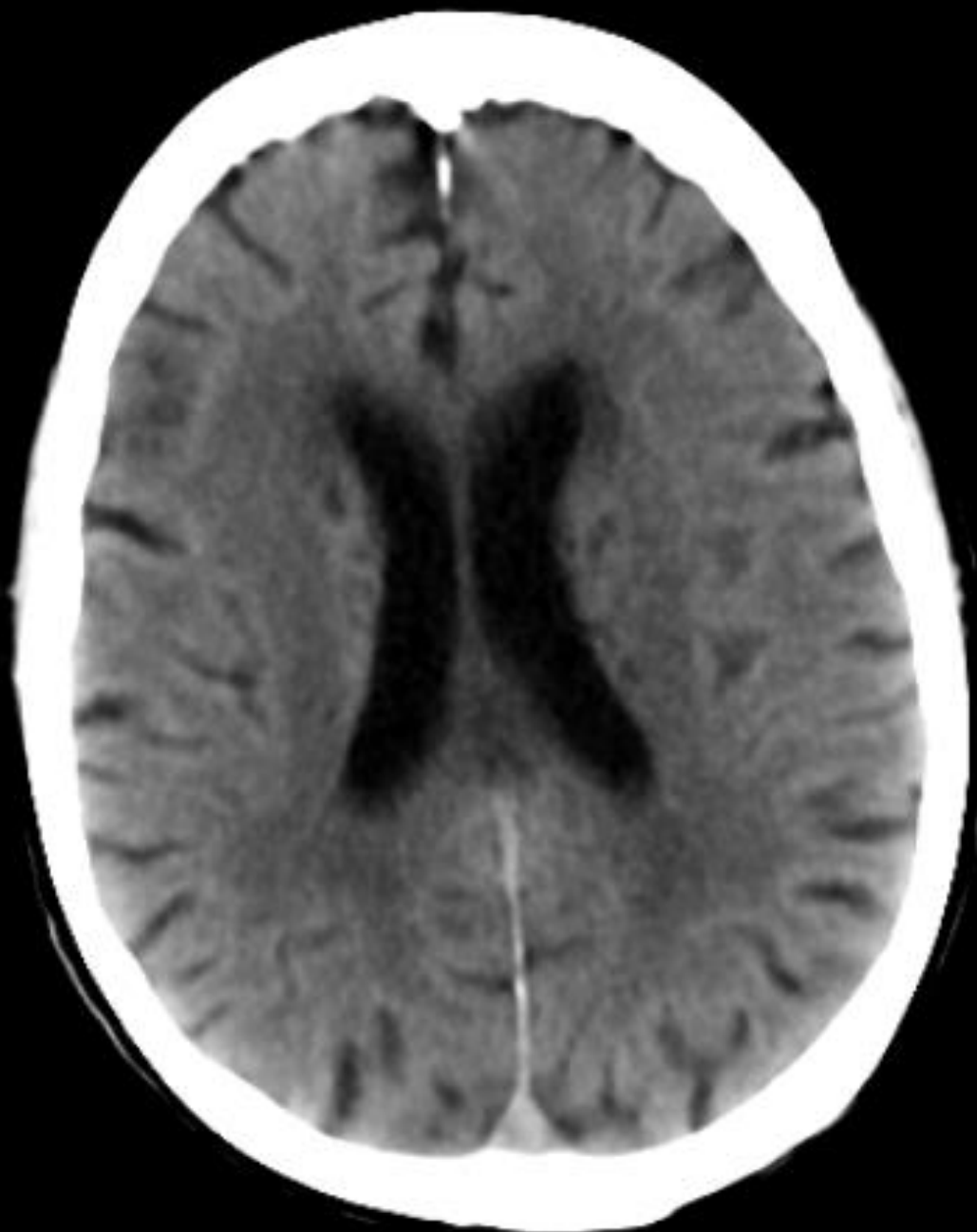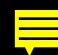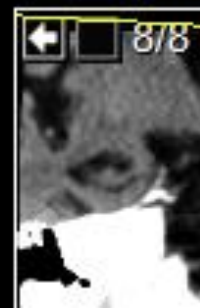

8/8

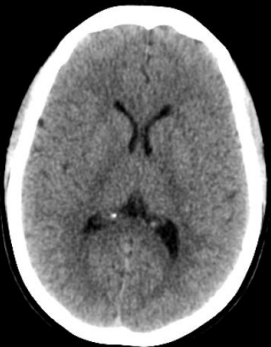

12

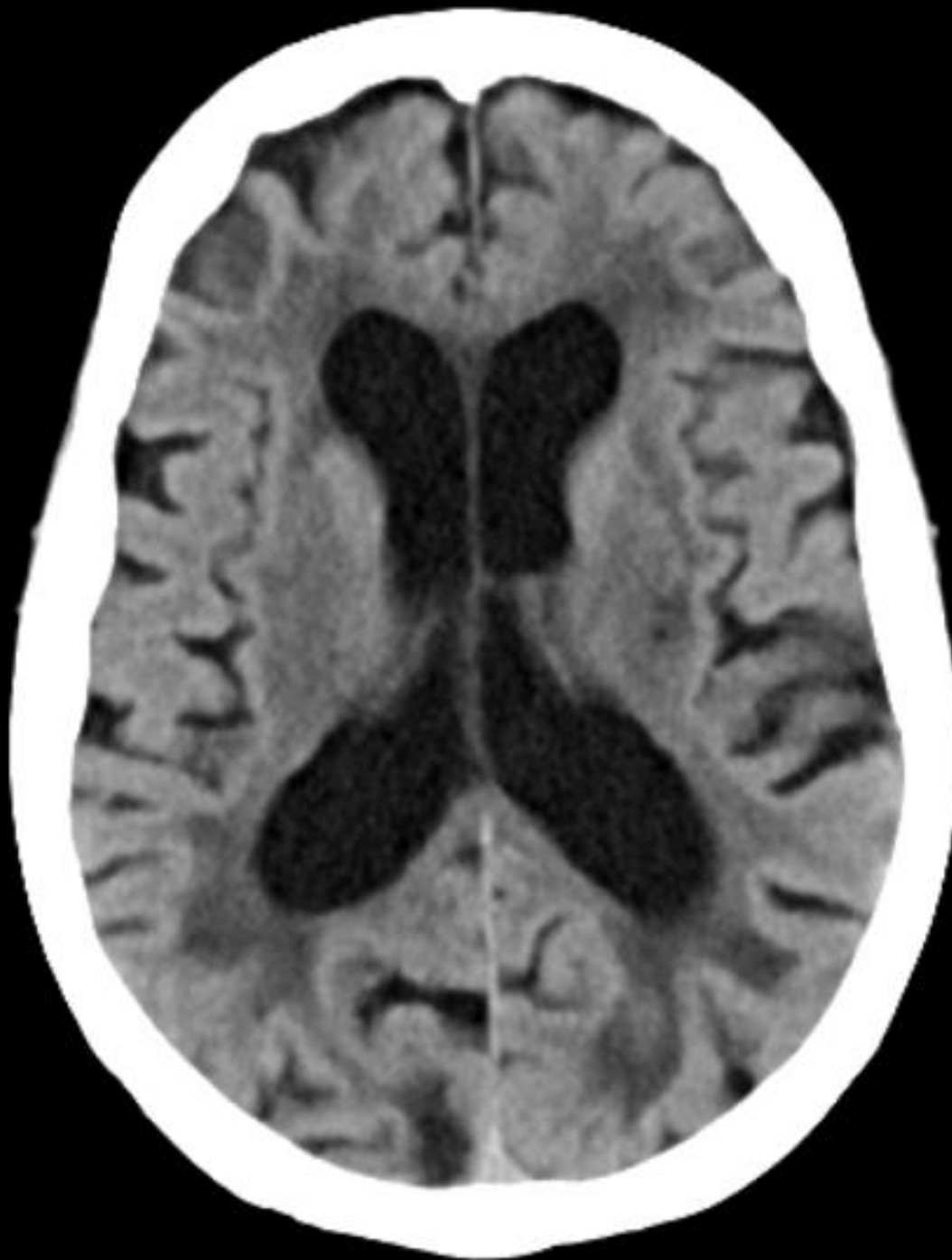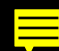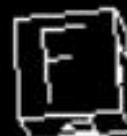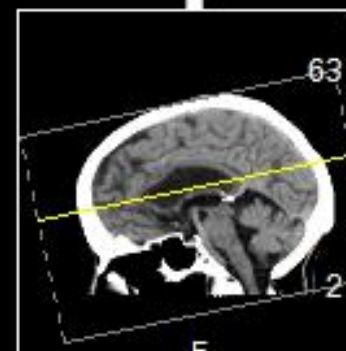

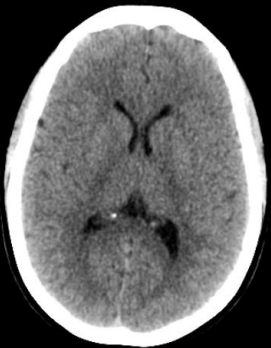

13

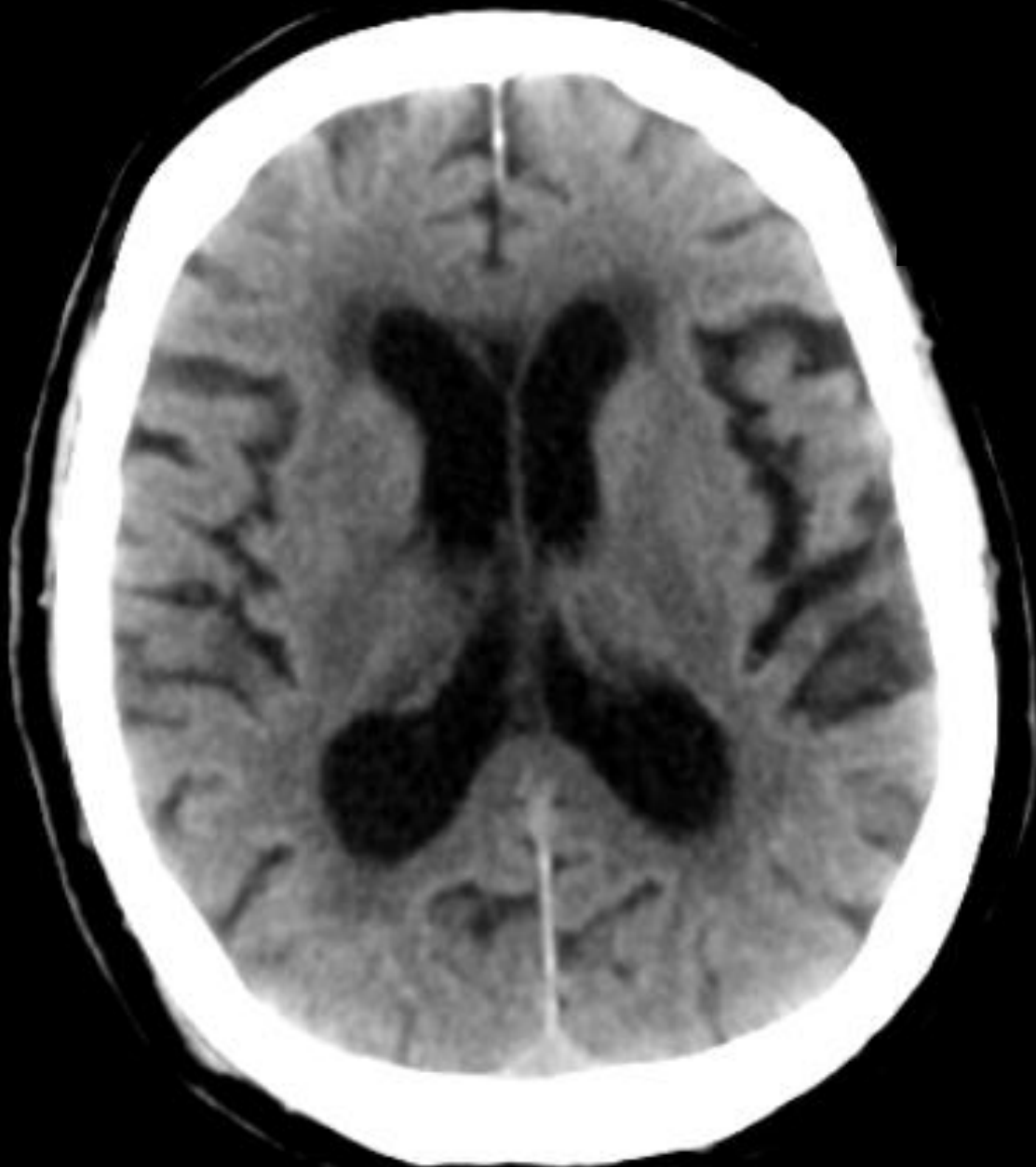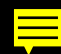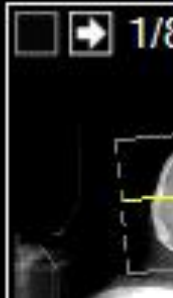

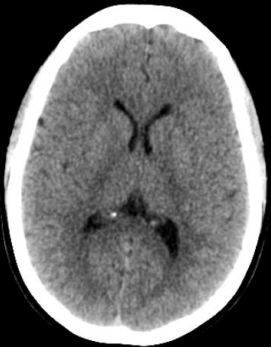

14

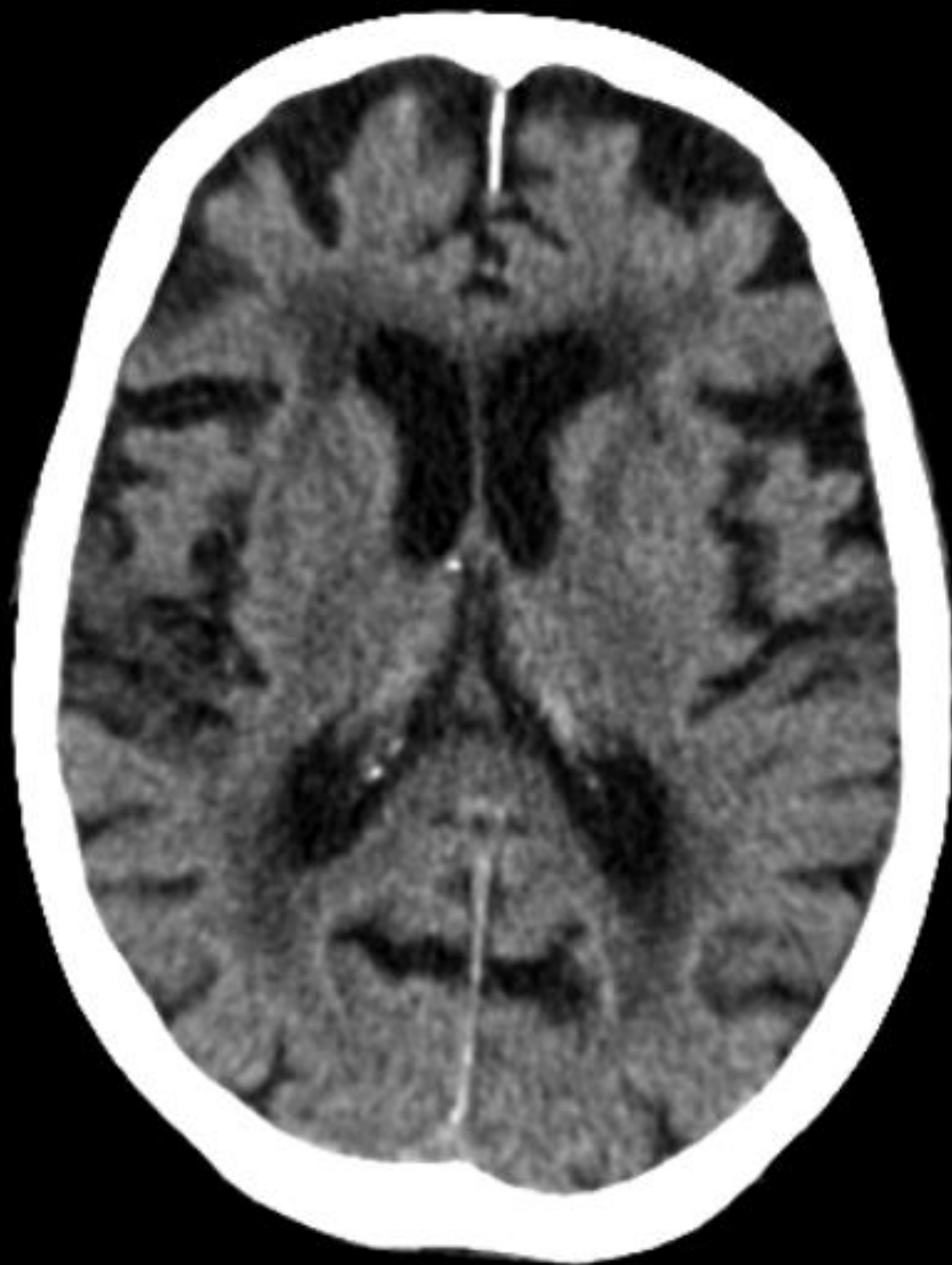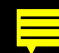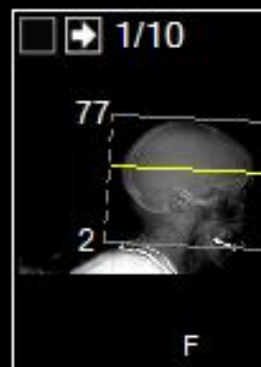

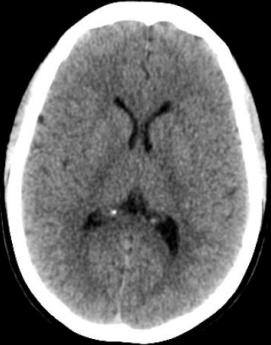

15

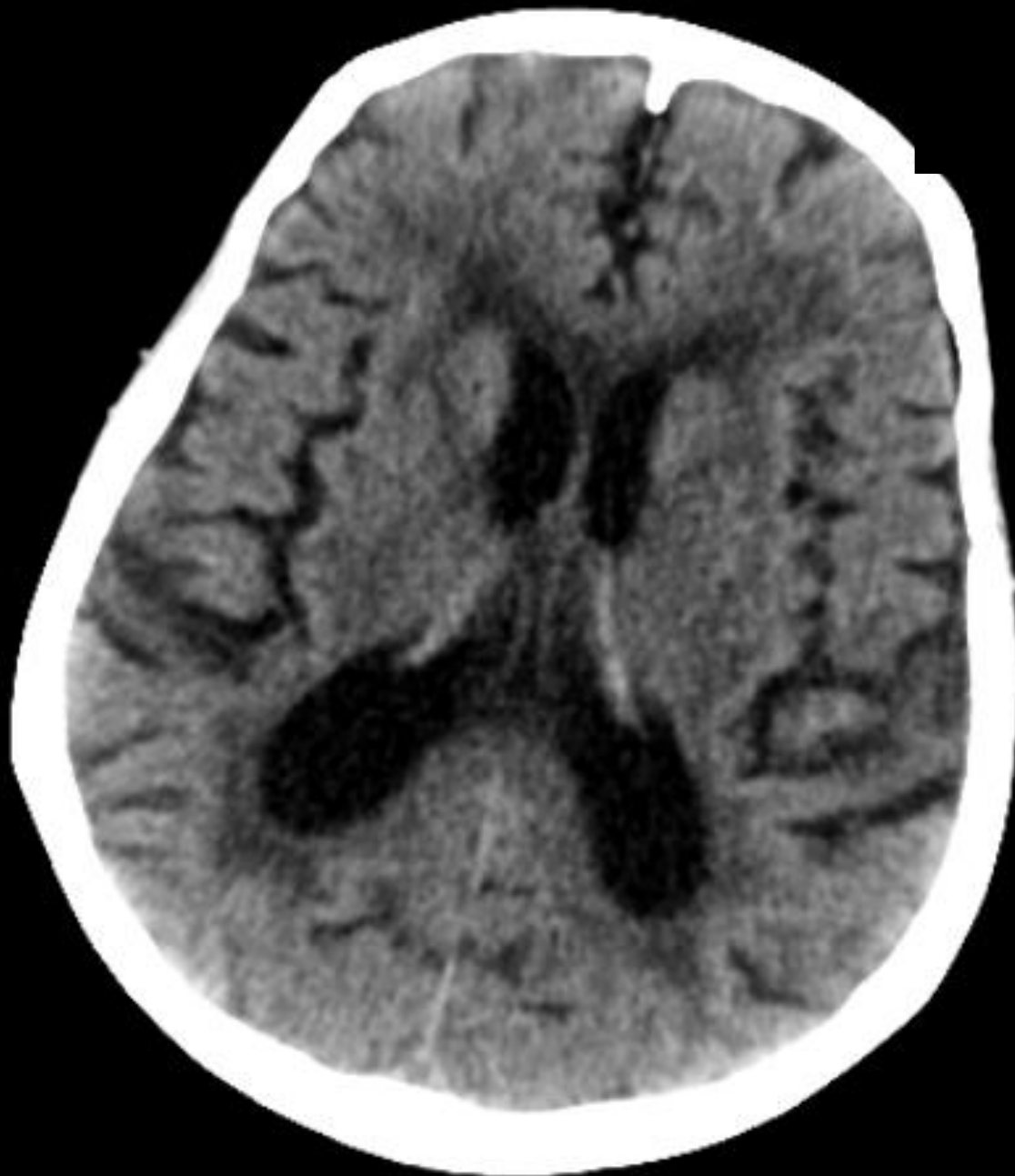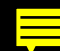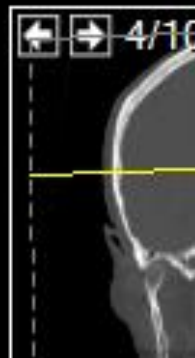

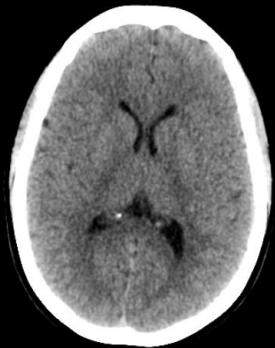

16

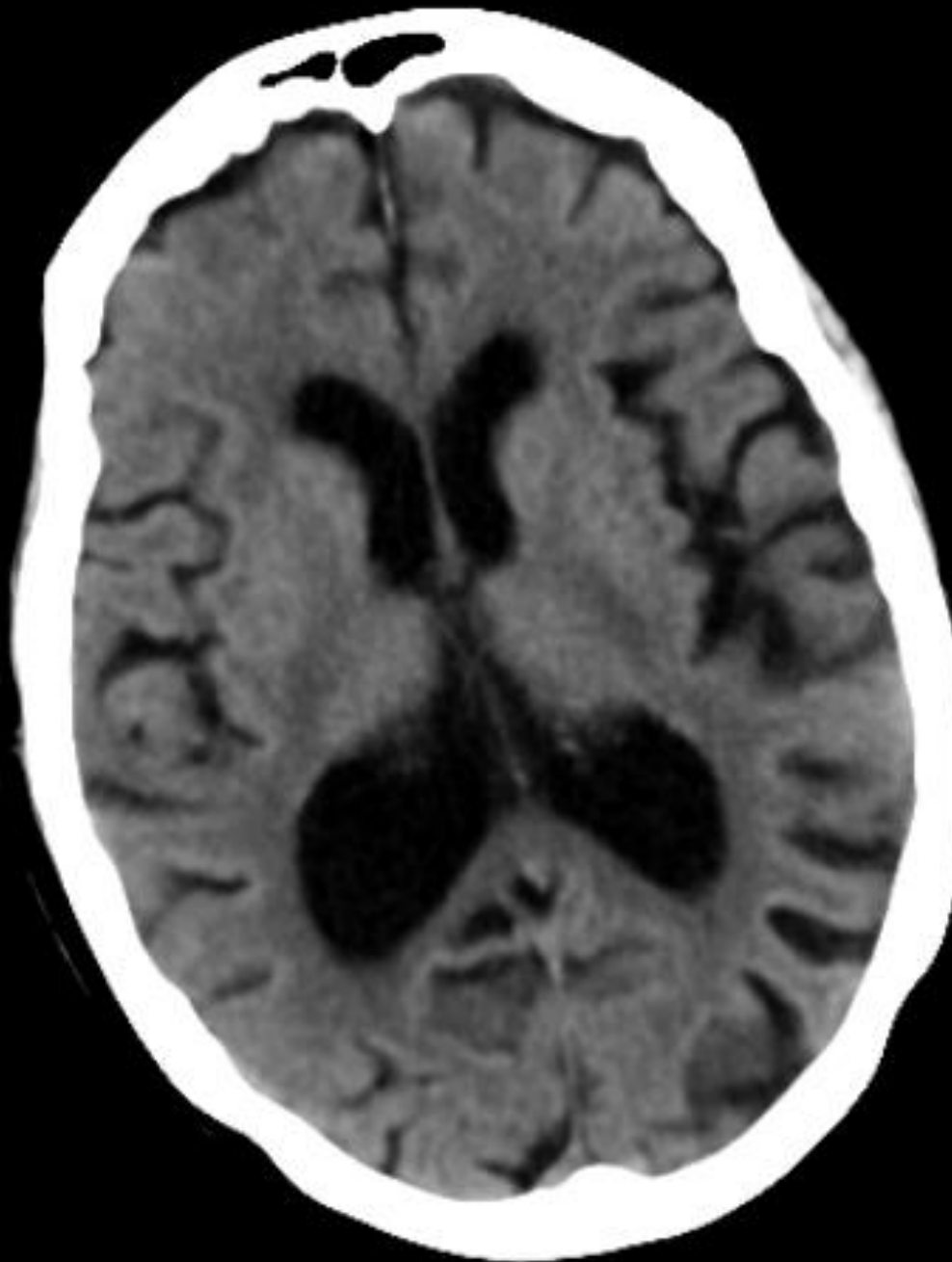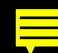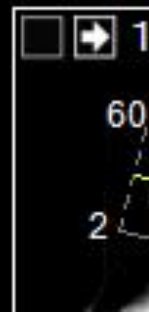

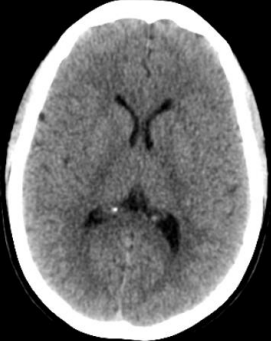

17

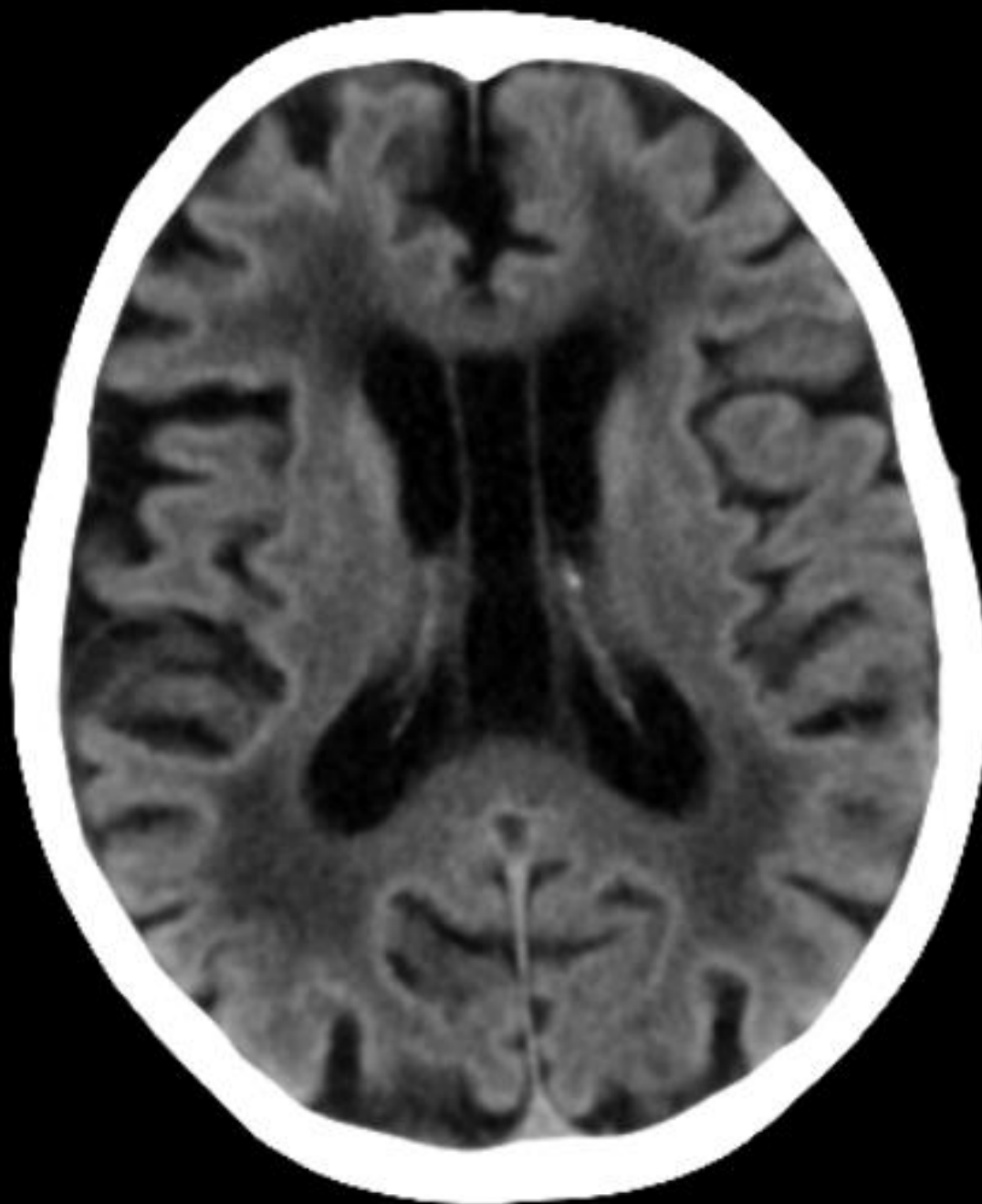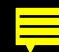

1/8

63

2

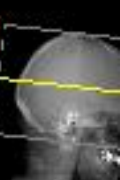

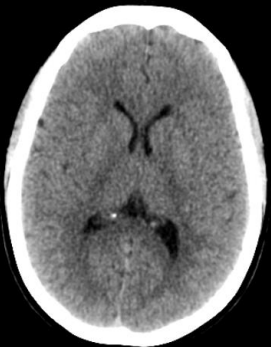

18

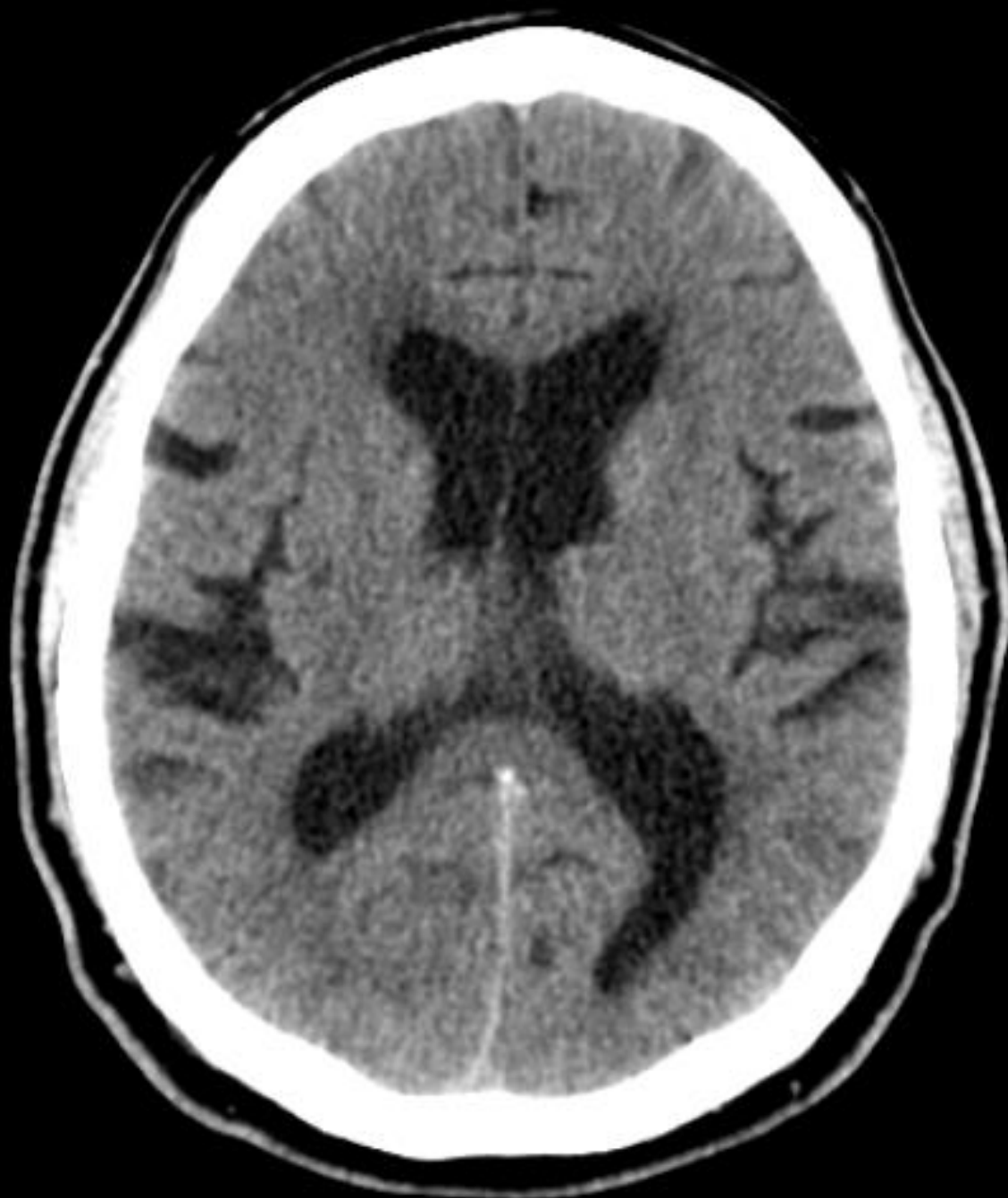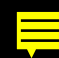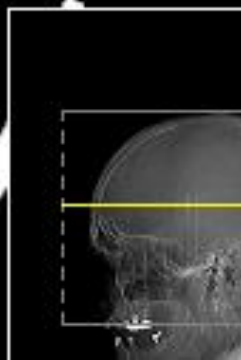

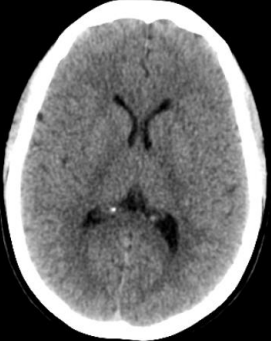

19

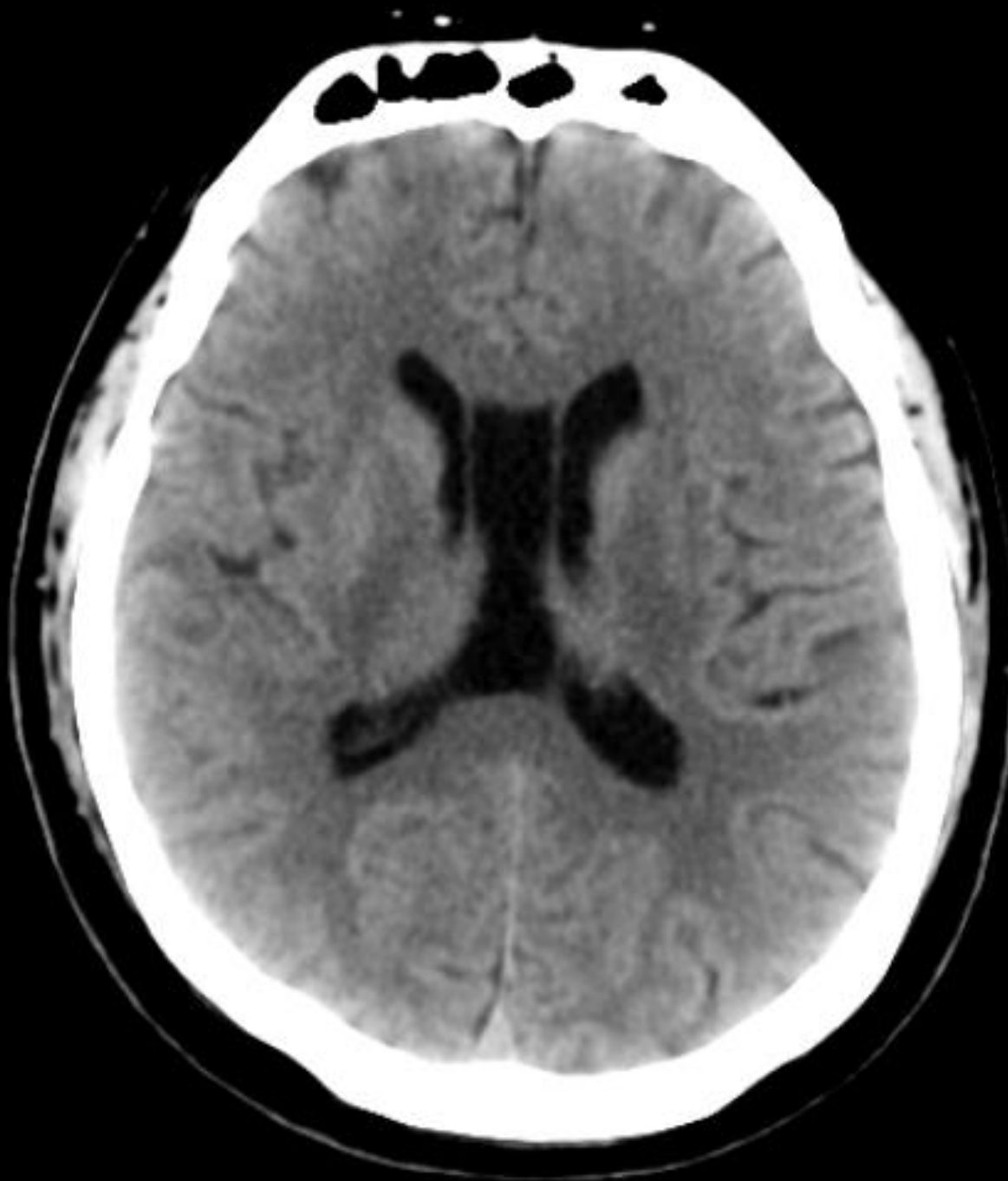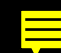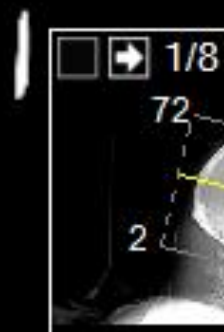

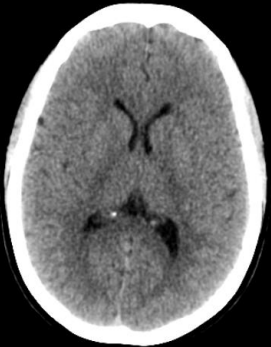

20

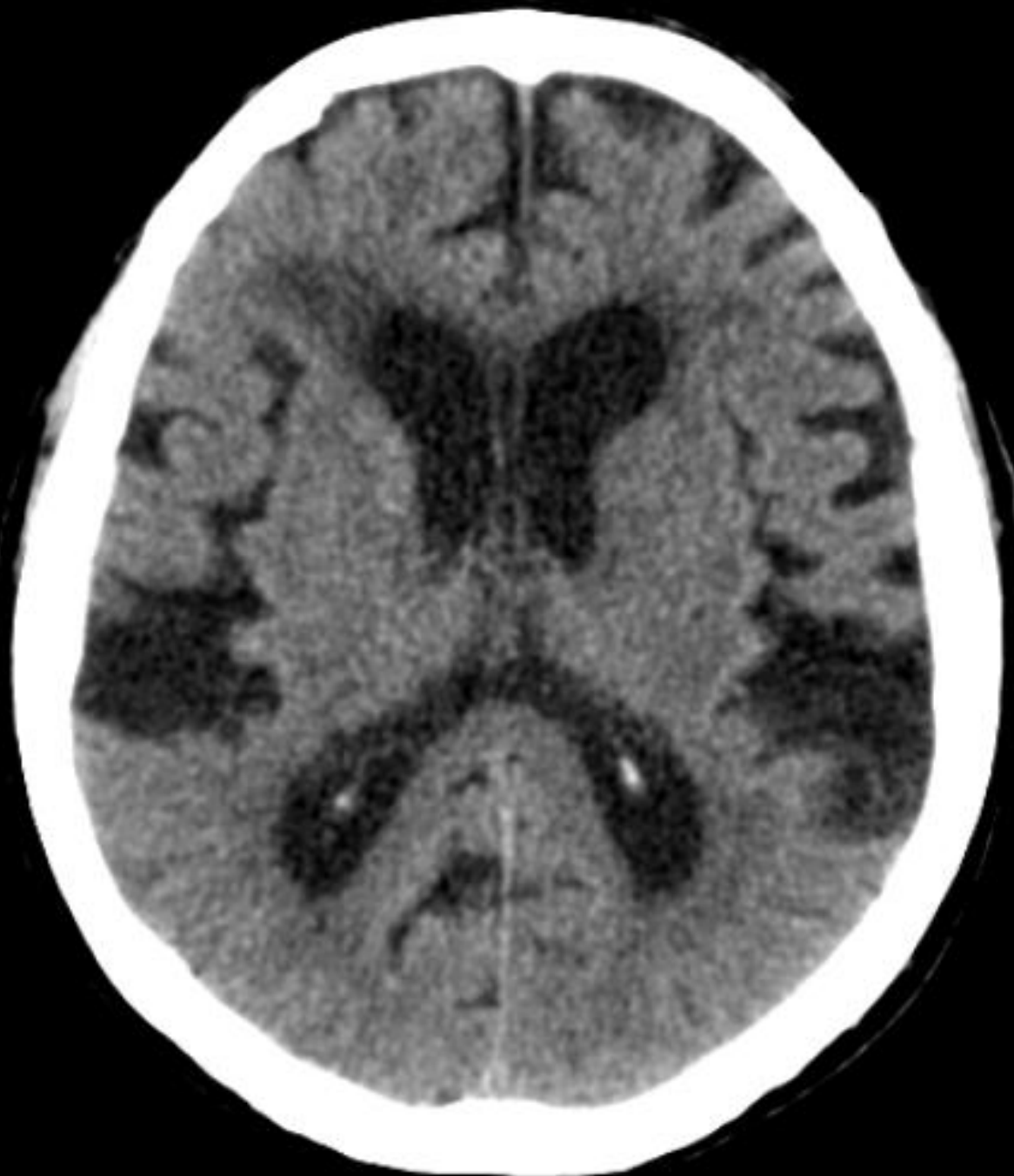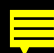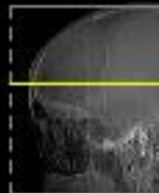

Supplement: Supplementary file 2 — (PDF 801 kb) [file 13244_2016_521_MOESM2_ESM.pdf]

1

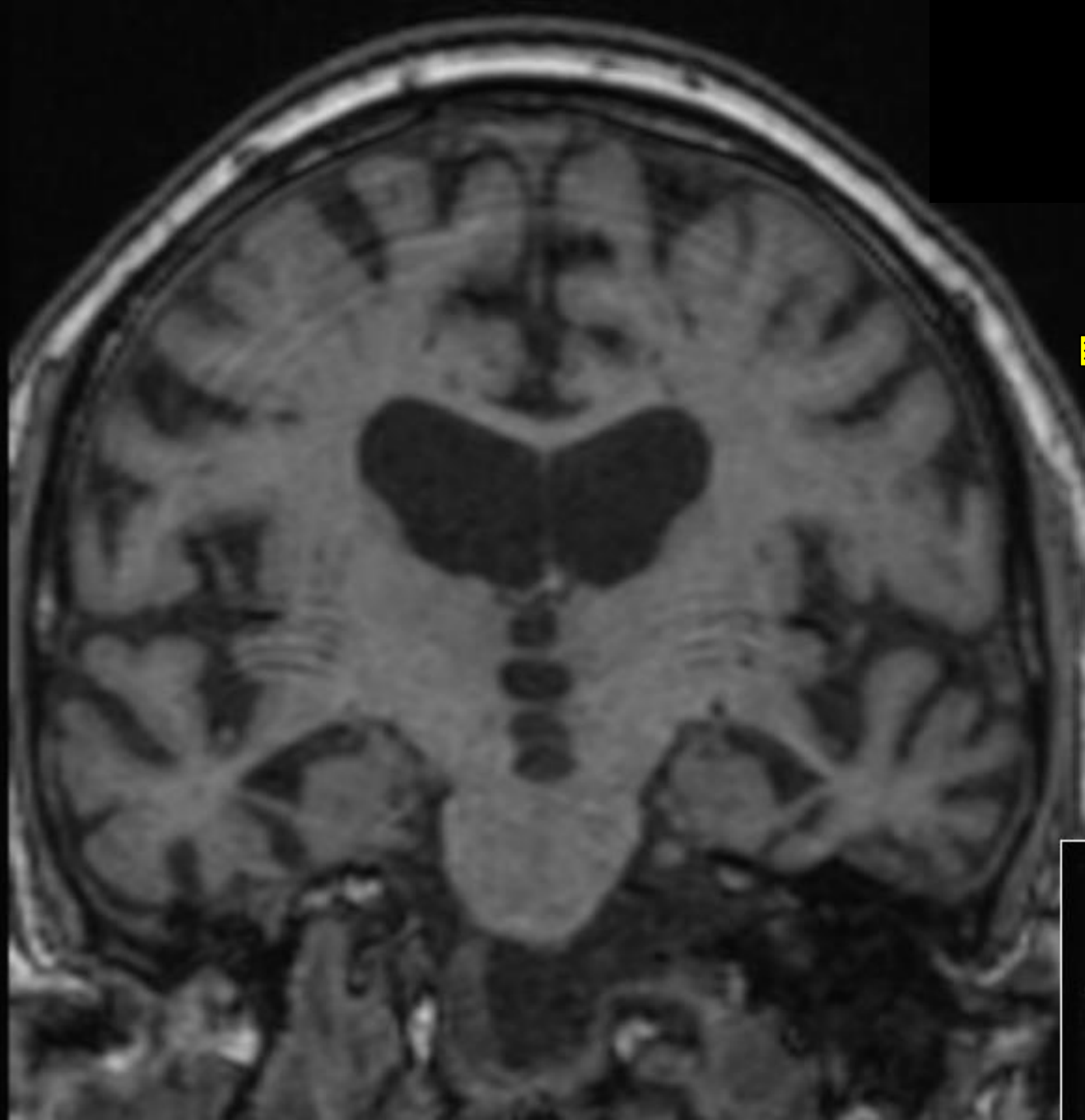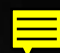

Cor>Tra 4  
>Sag -0

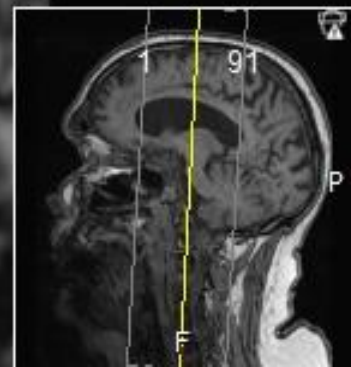

2

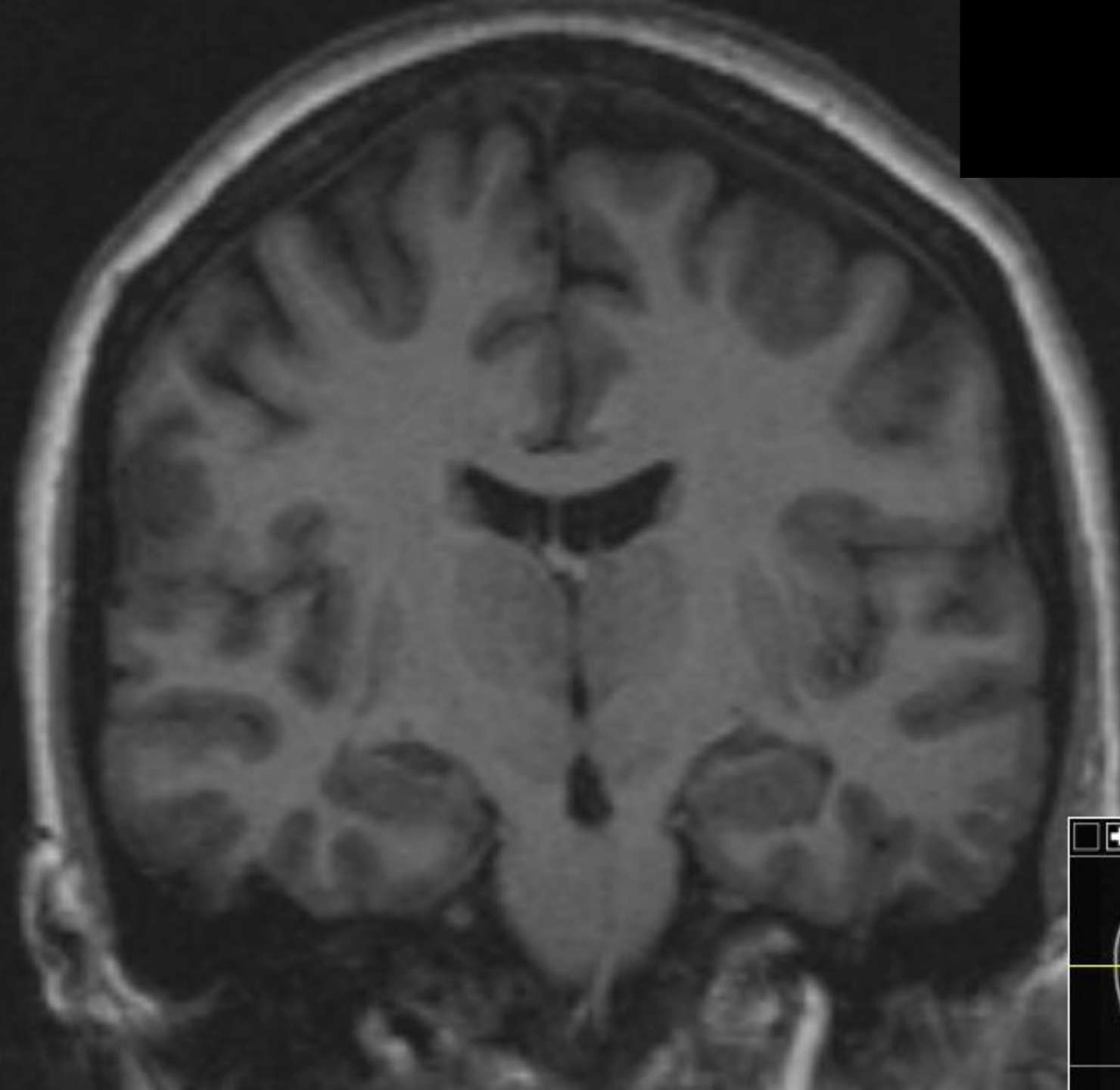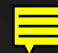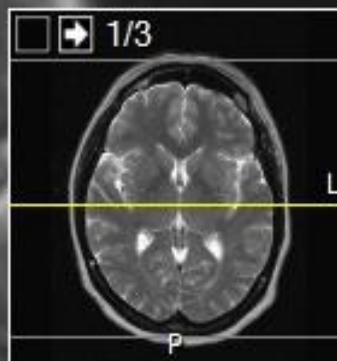

3

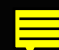

Cor>Tra -  
>Sag -1

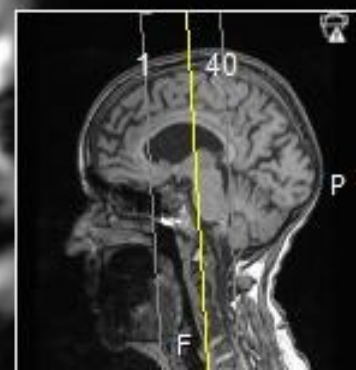

4

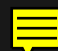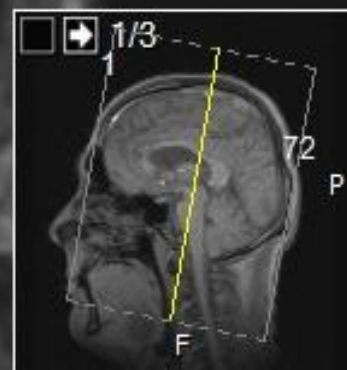

5

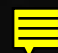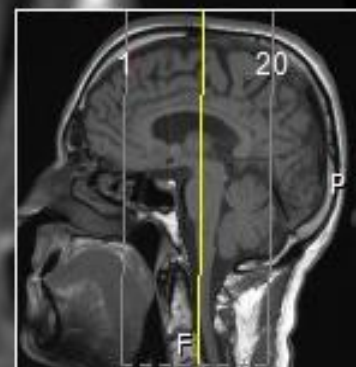

6

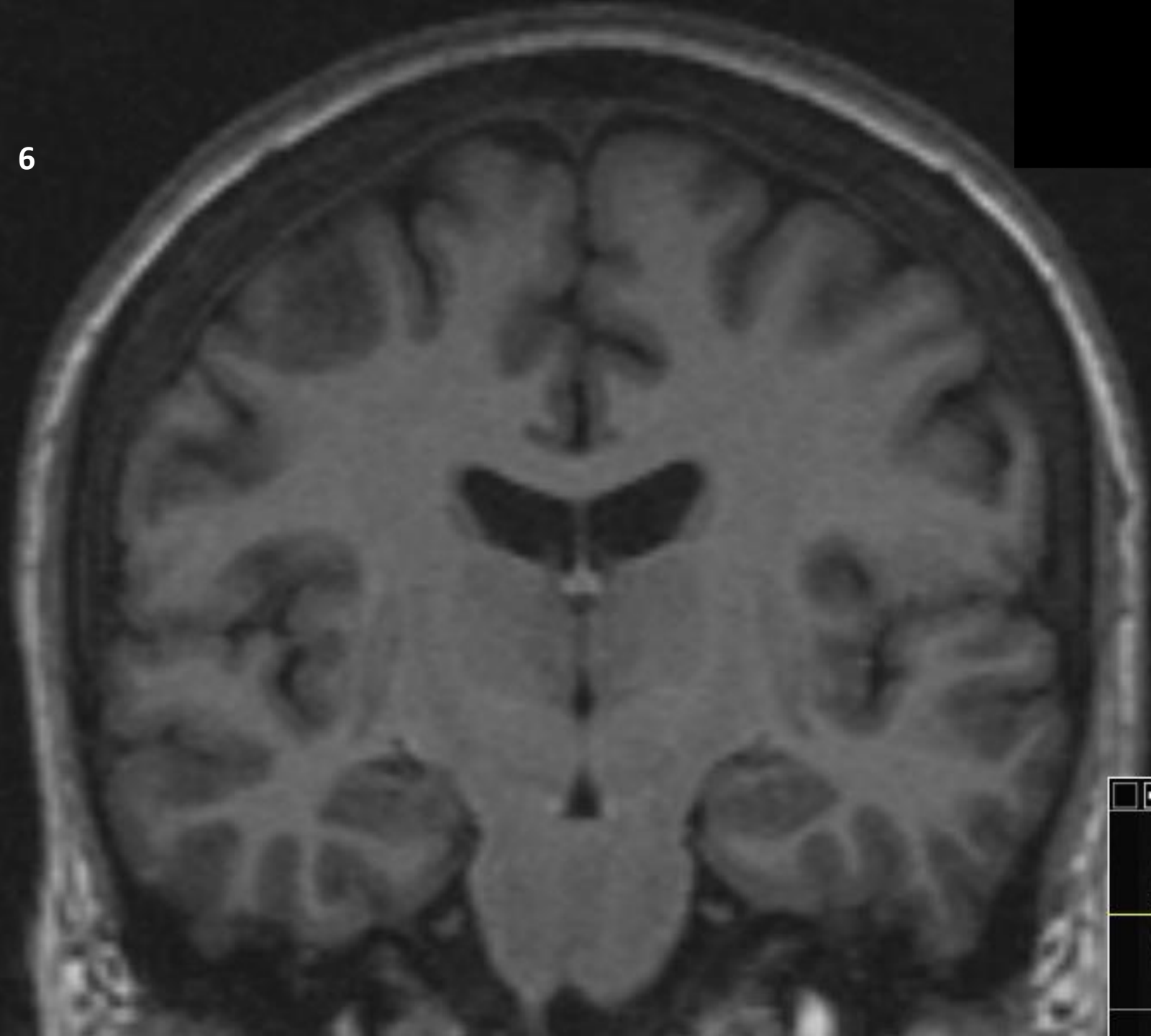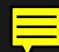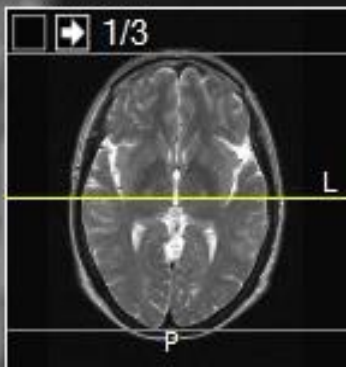

7

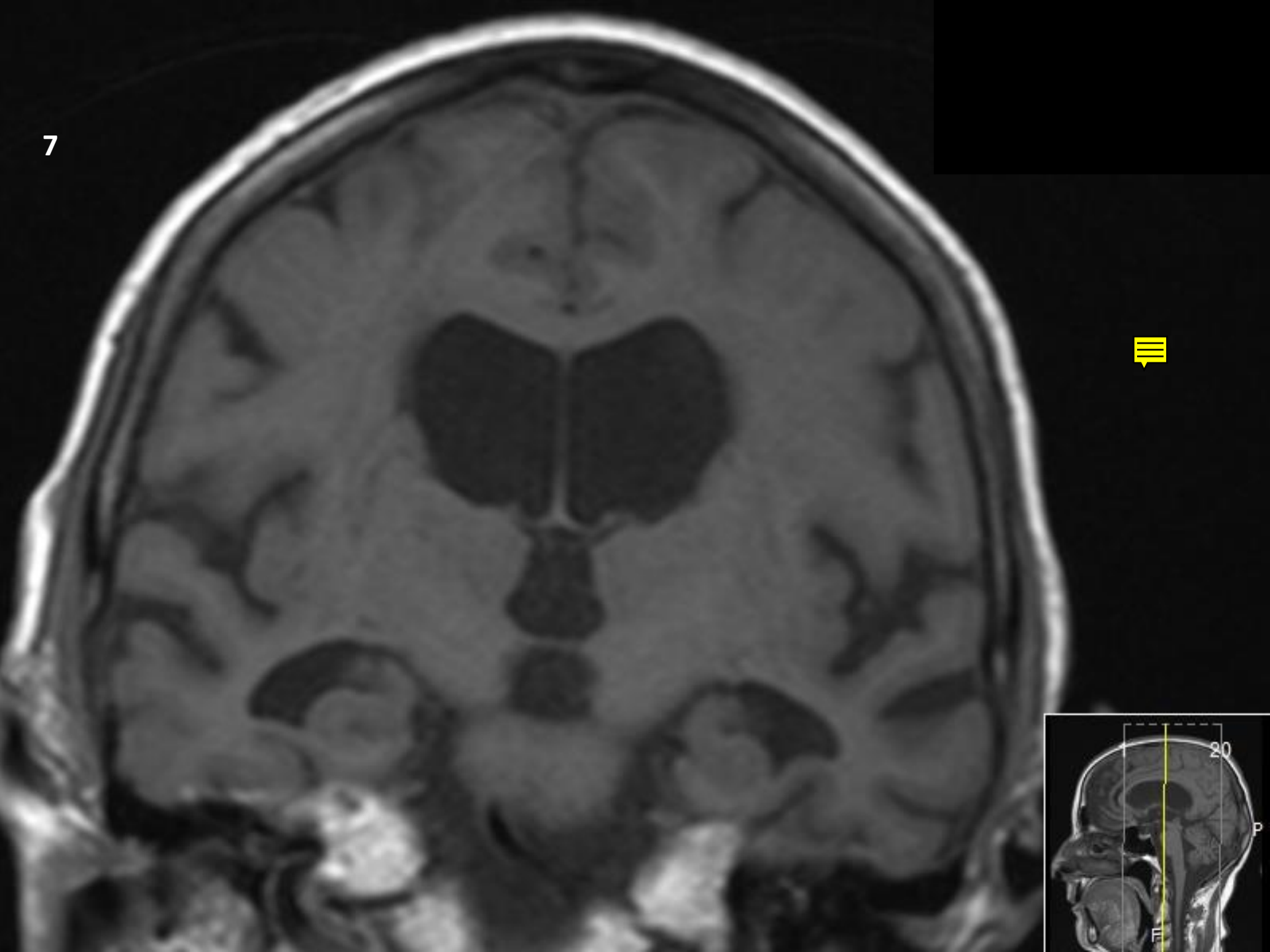

8

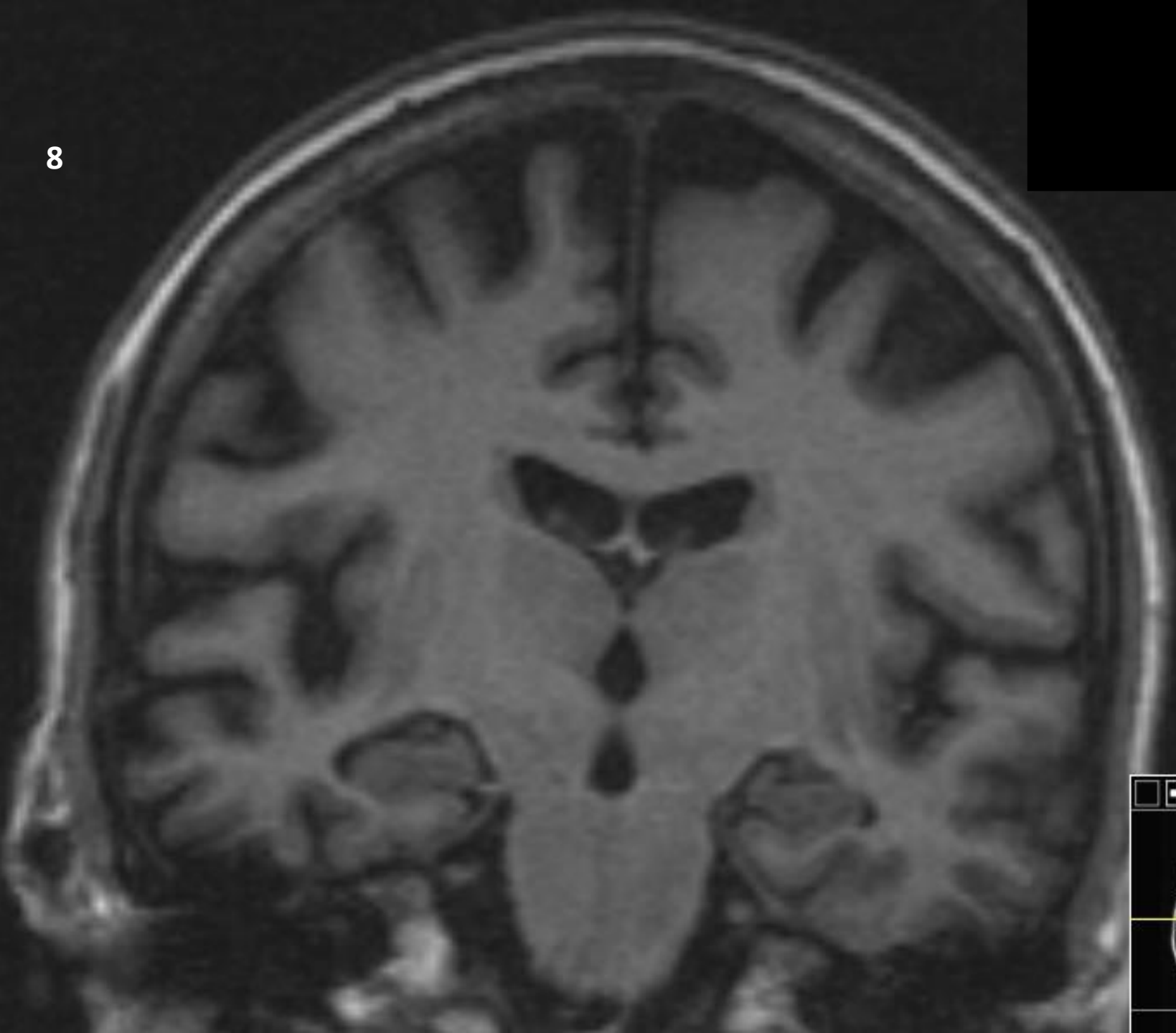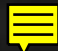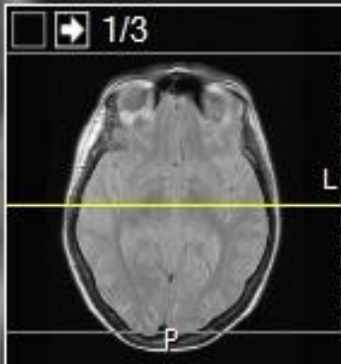

9

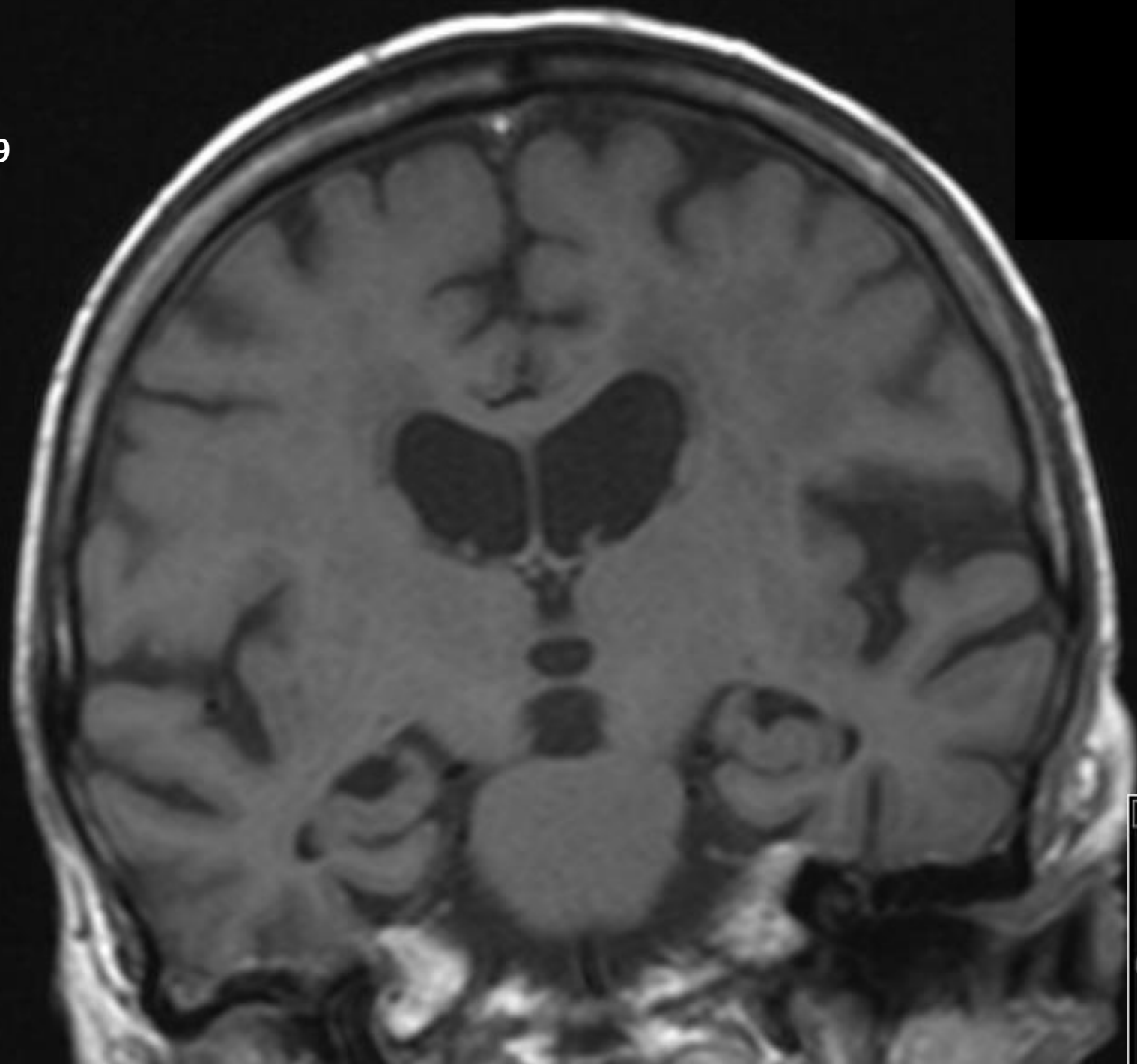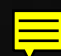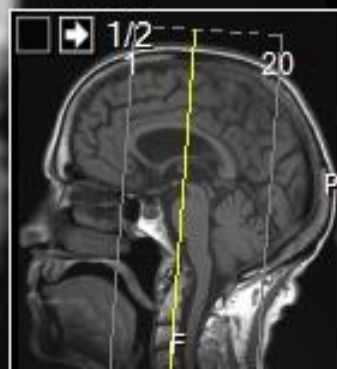

10

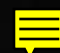

Cor>Sag -10  
>Tra -6

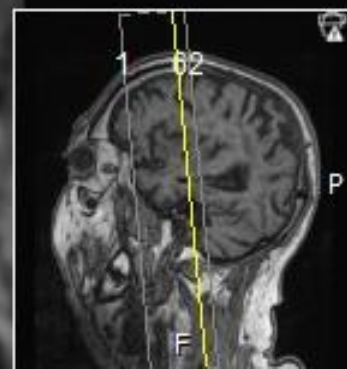

11

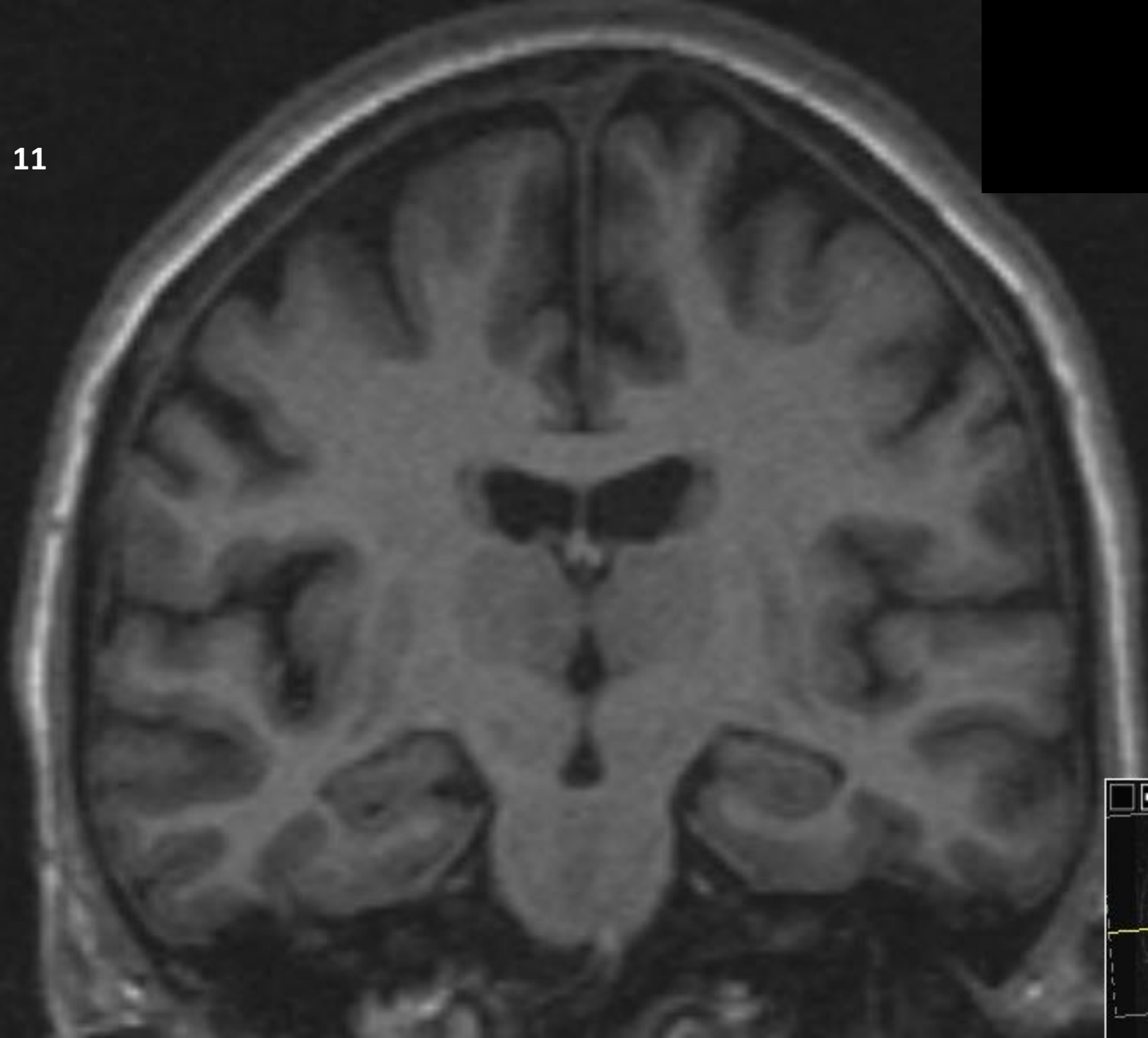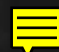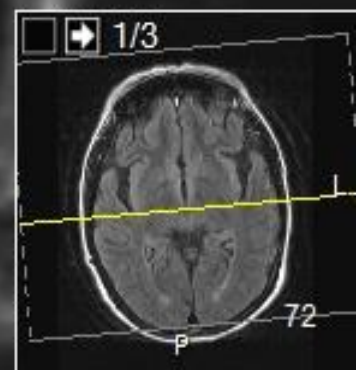

12

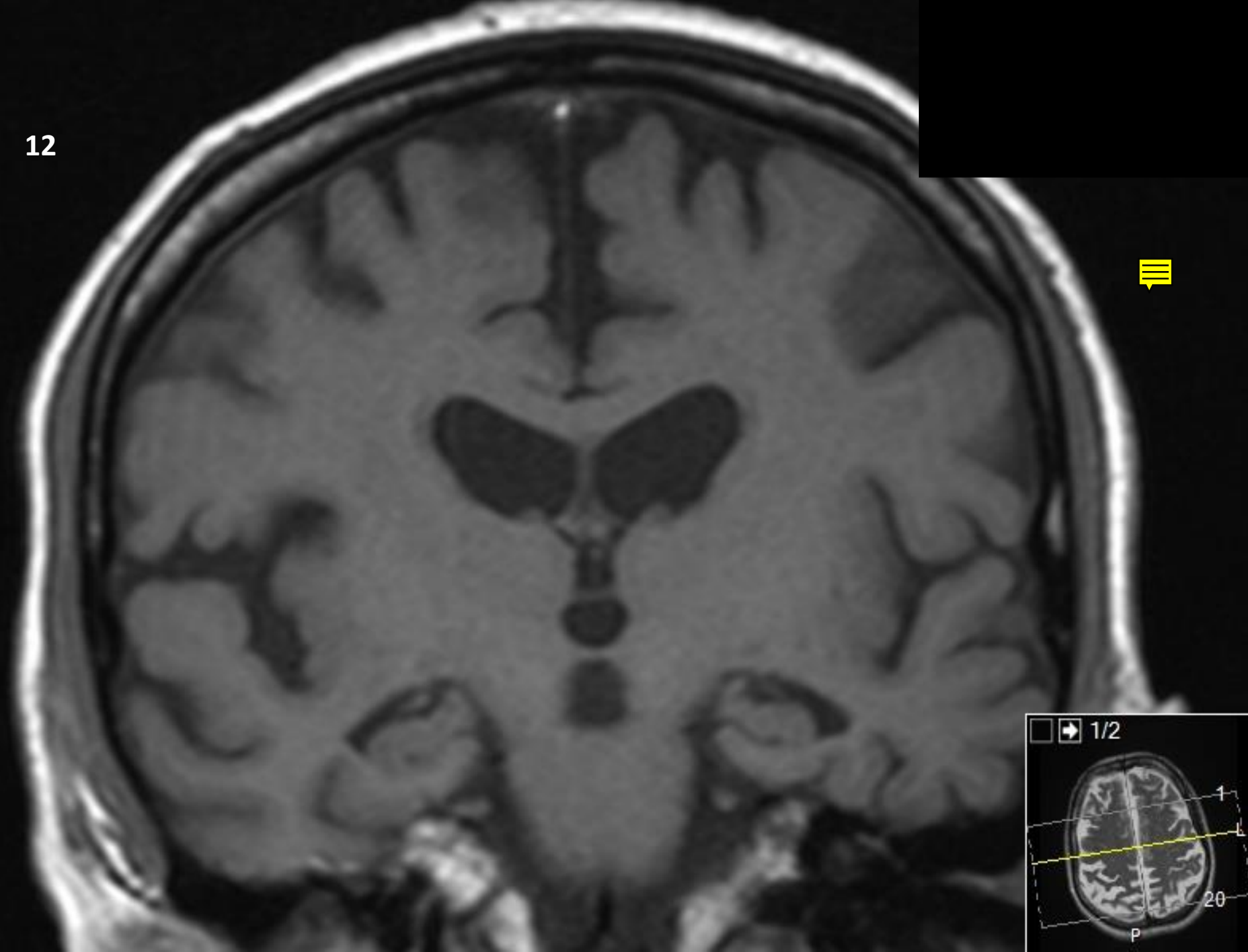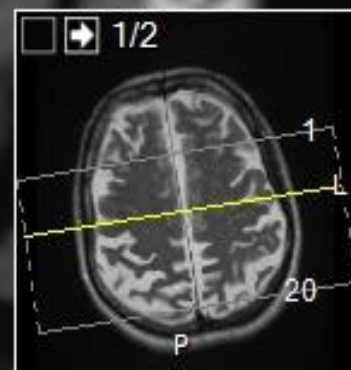

13

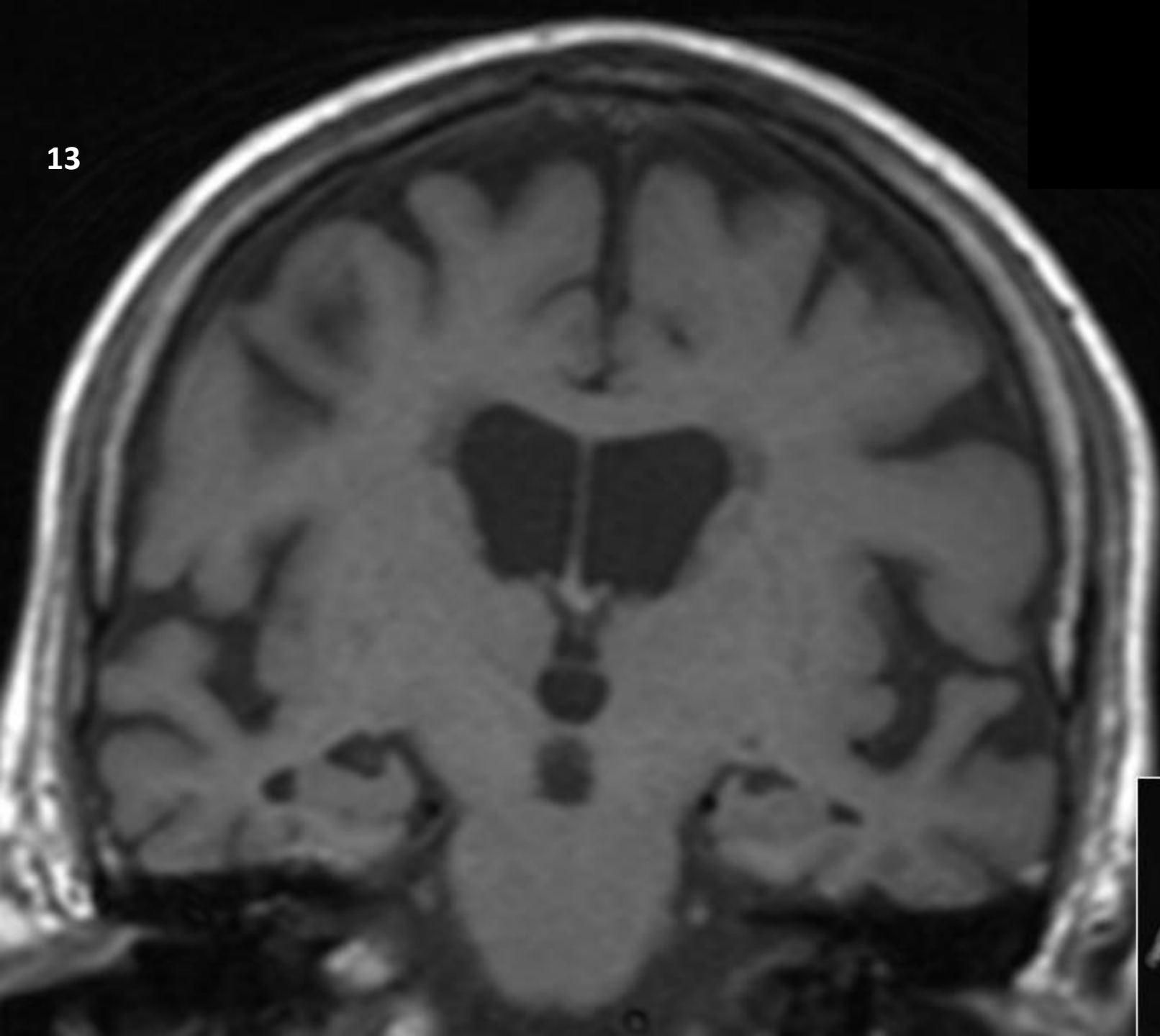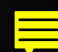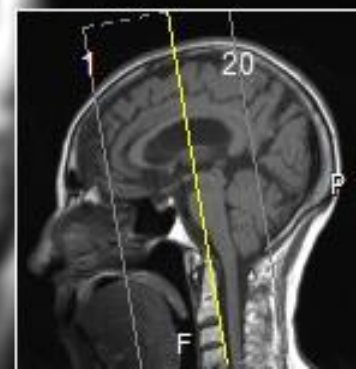

14

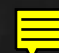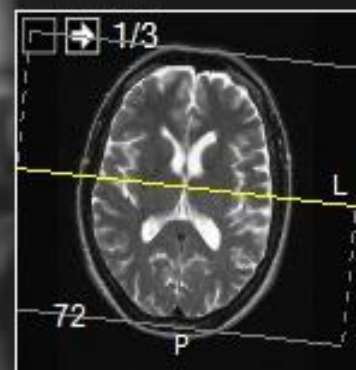

15

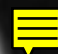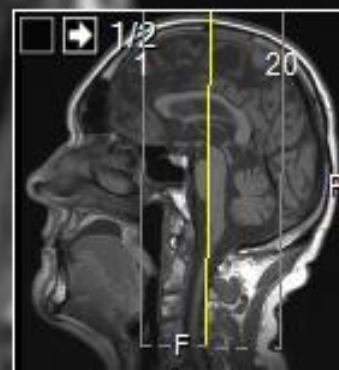

16

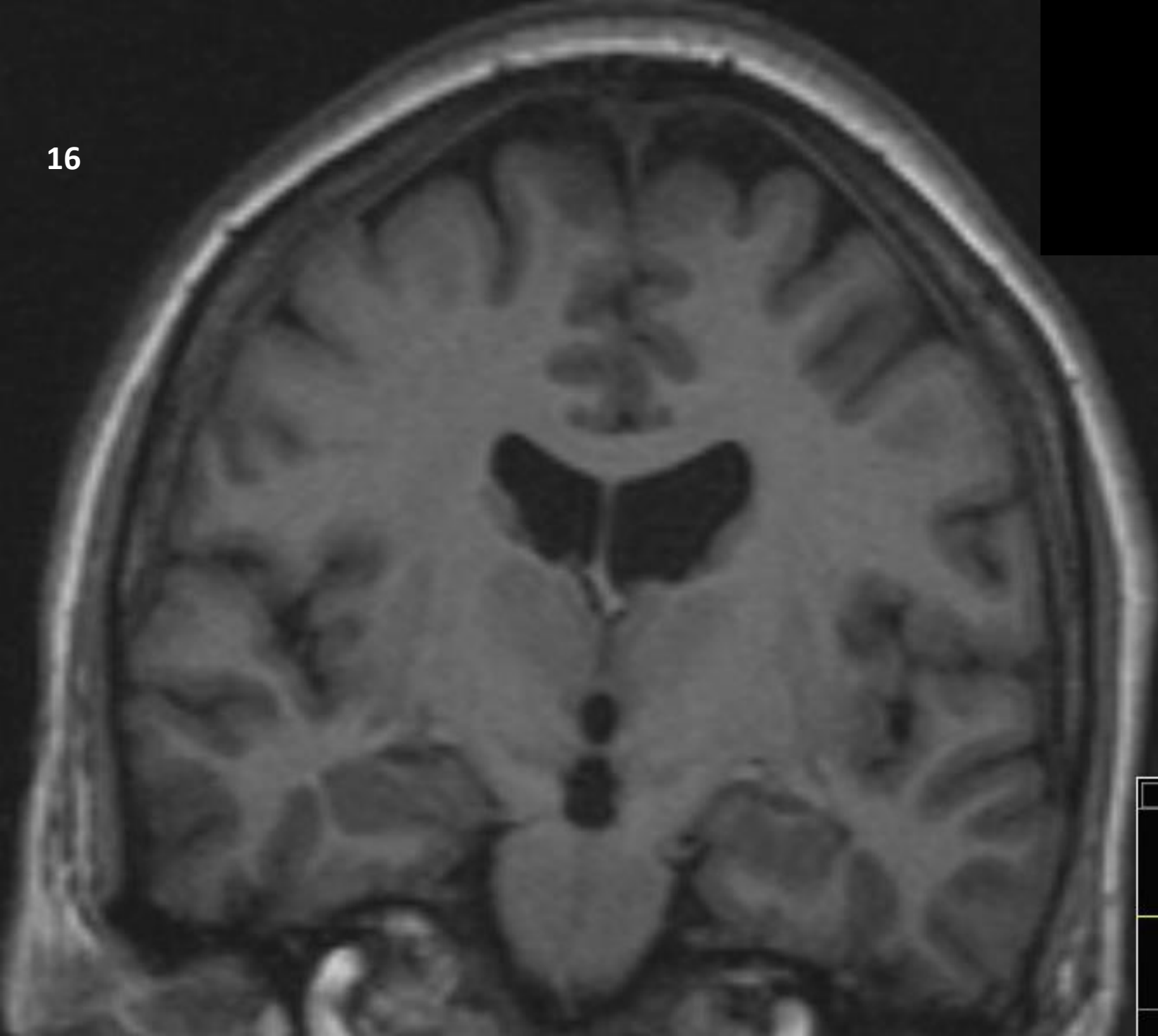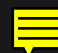

1/3

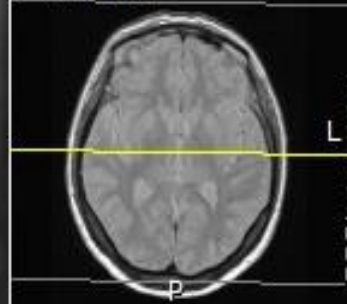

17

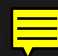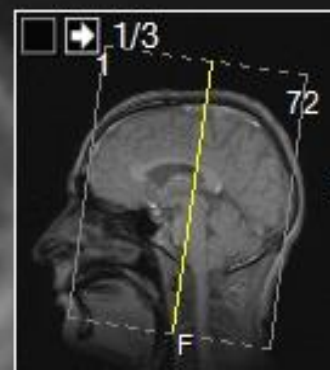

18

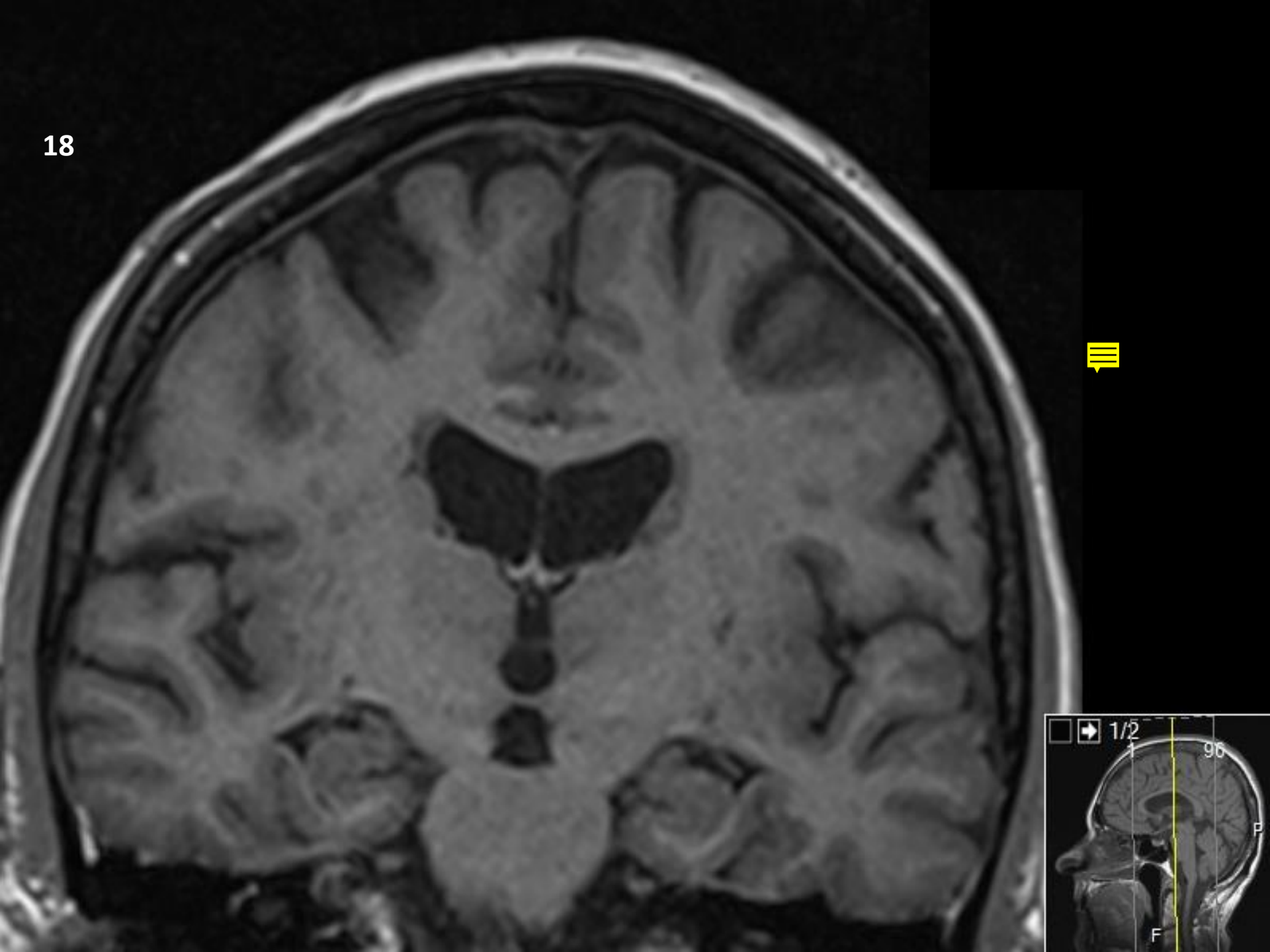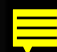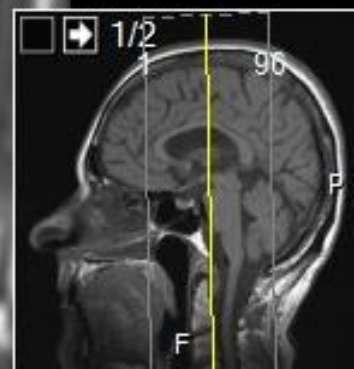

19

Cor>Tra 4  
>Sag 1

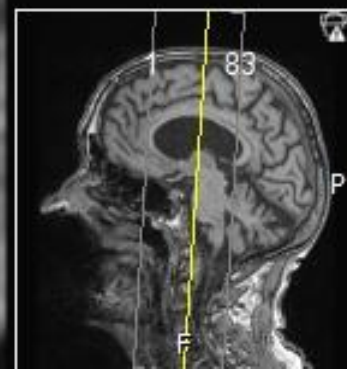

20

Cor>Sag 1  
>Tra 1

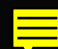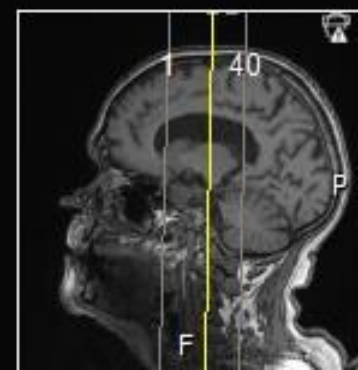

Supplement: Supplementary file 3 — (PDF 891 kb) [file 13244_2016_521_MOESM3_ESM.pdf]

1

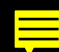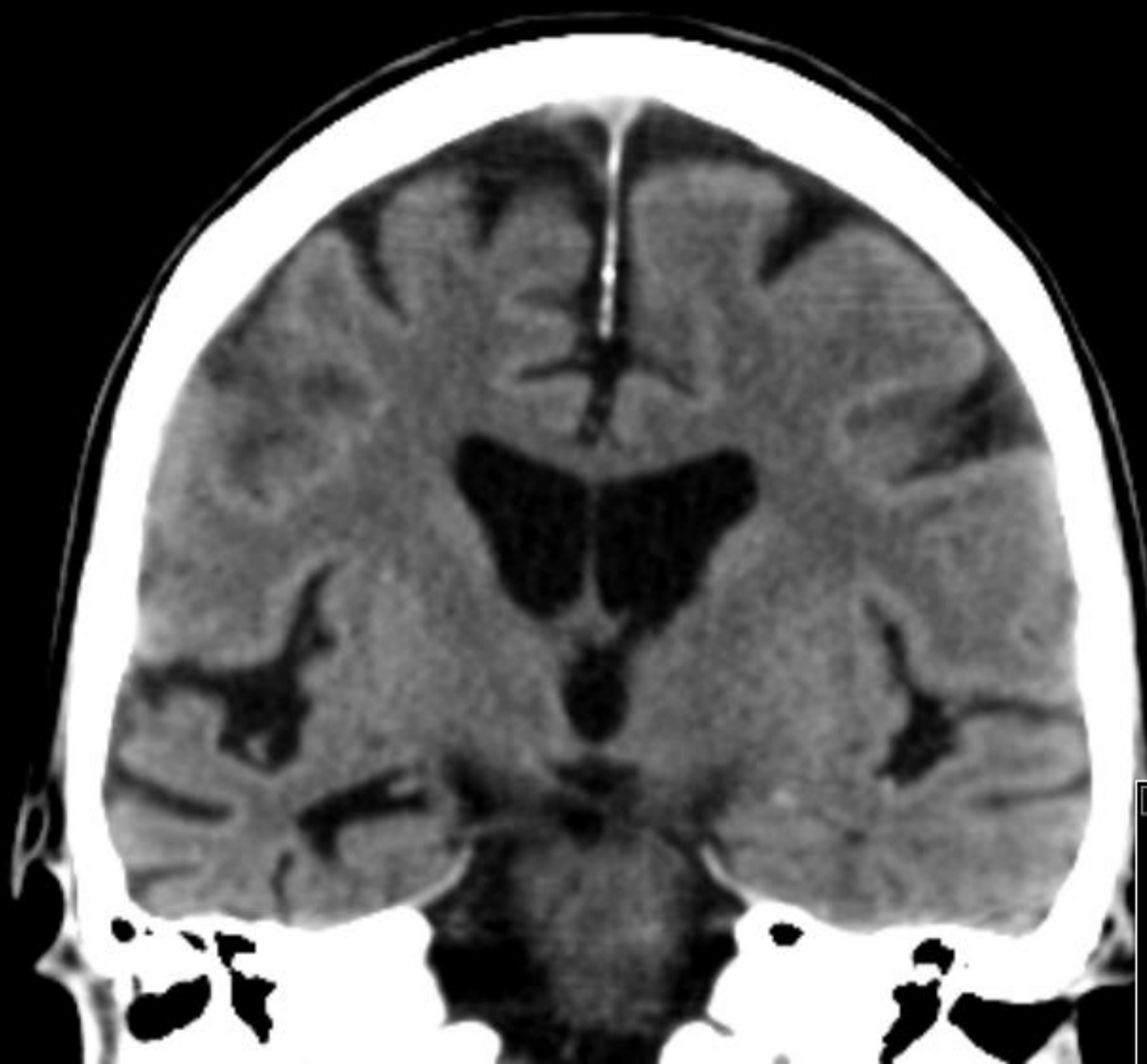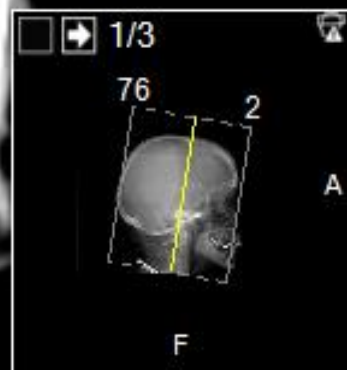

2

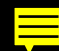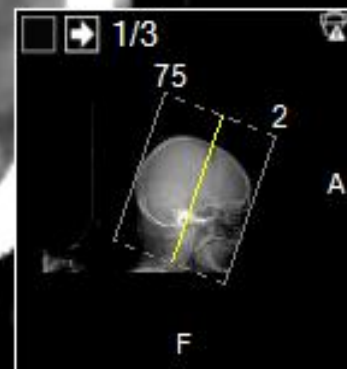

3

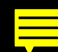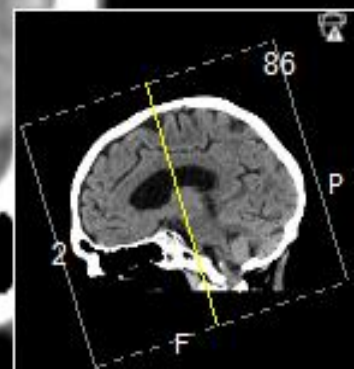

4

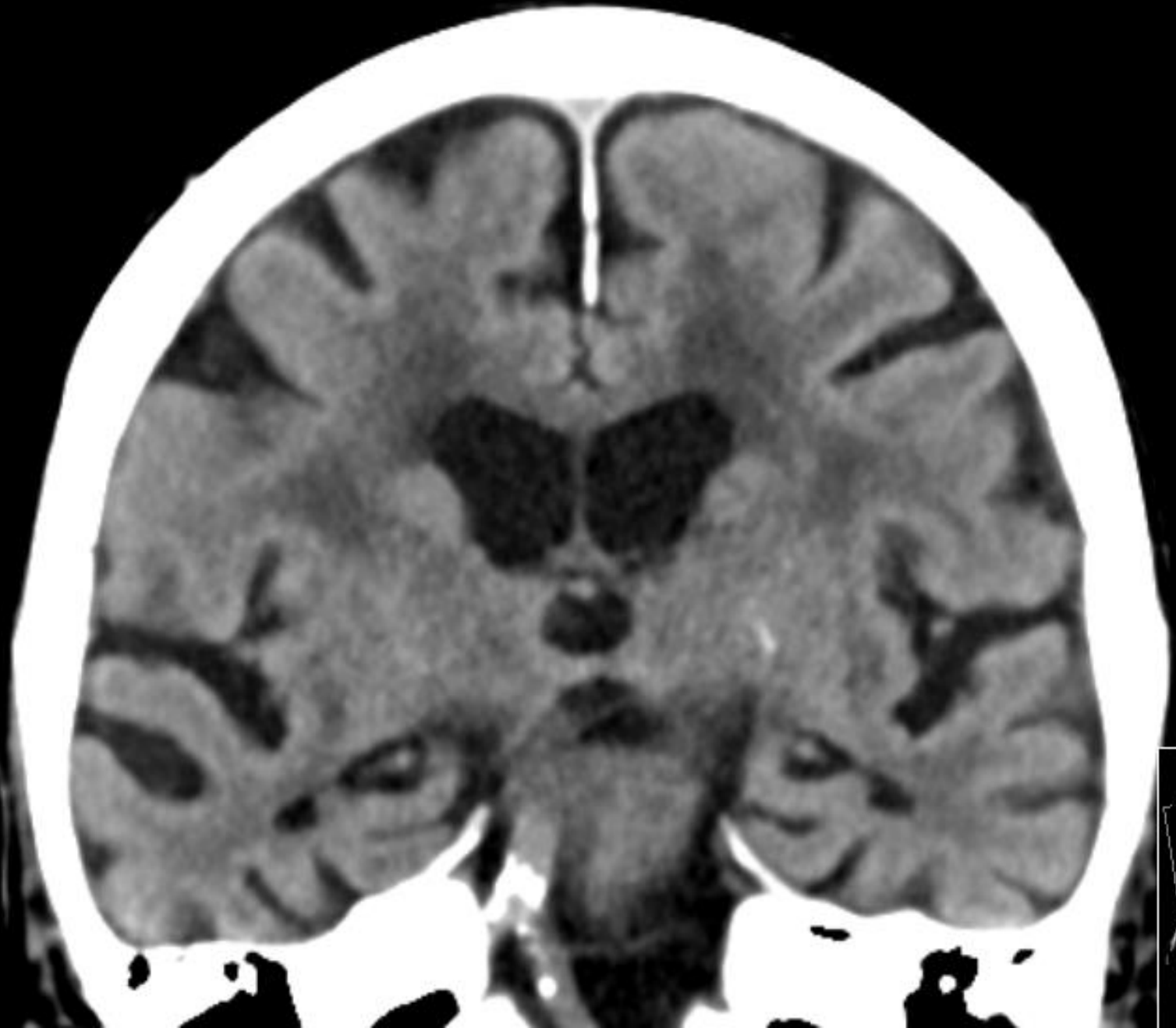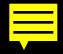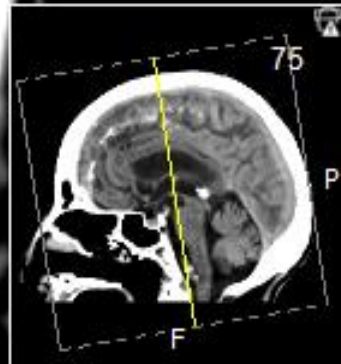

5

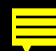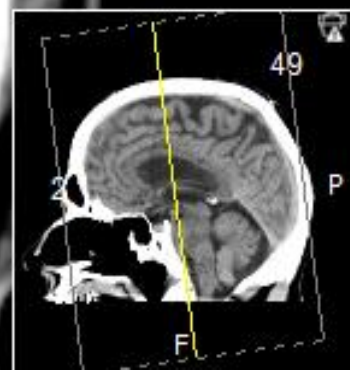

6

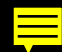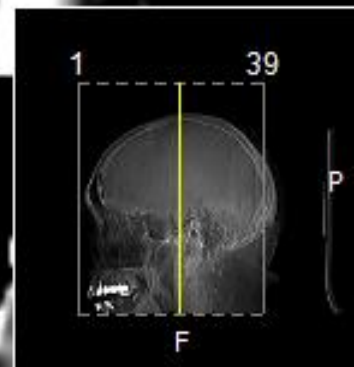

7

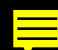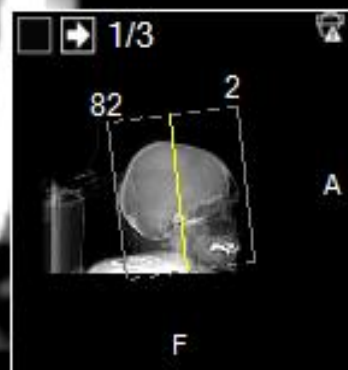

8

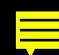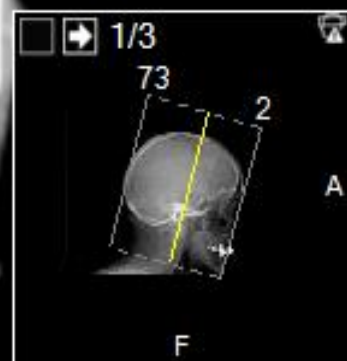

9

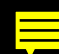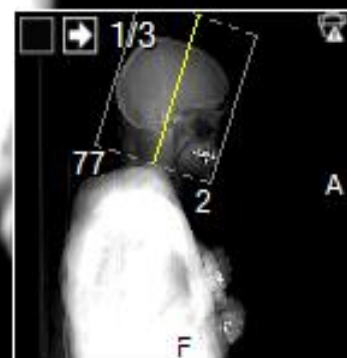

10

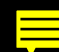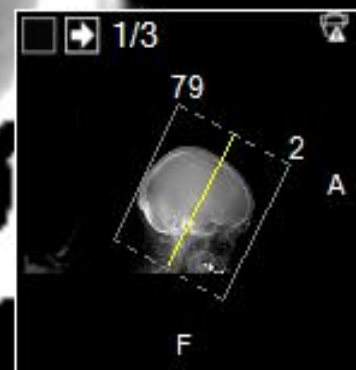

11

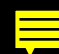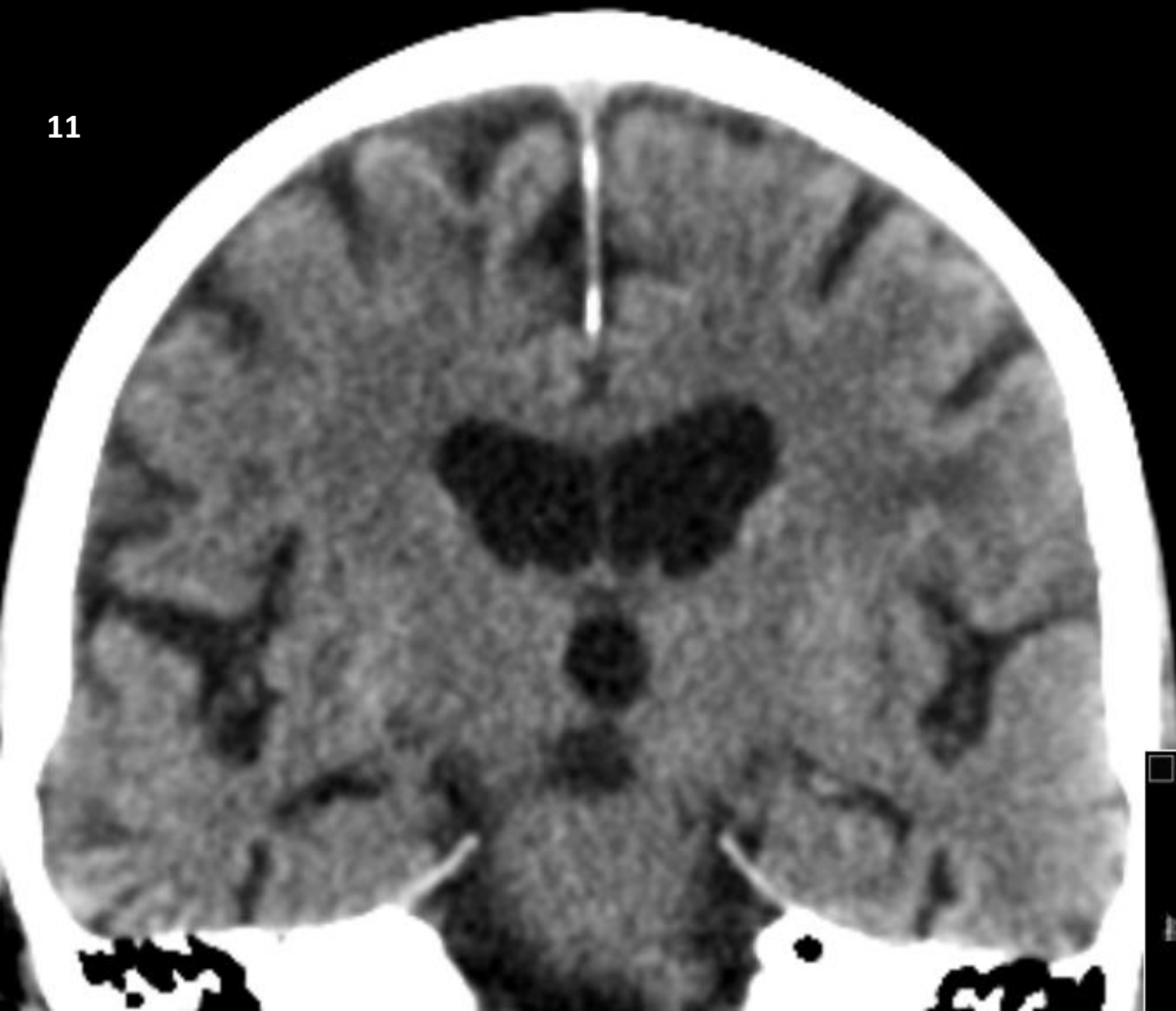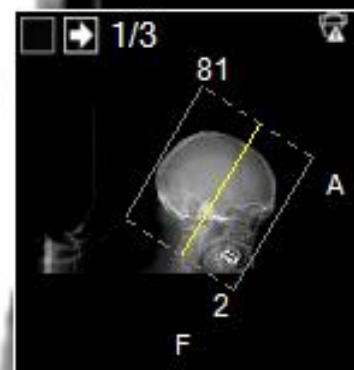

12

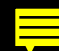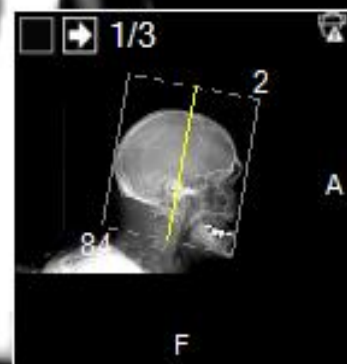

13

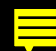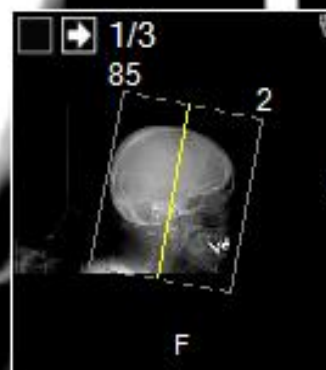

14

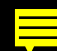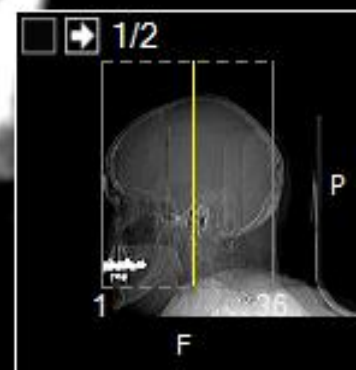

15

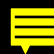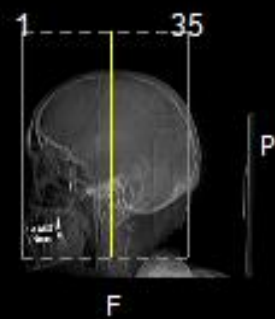

16

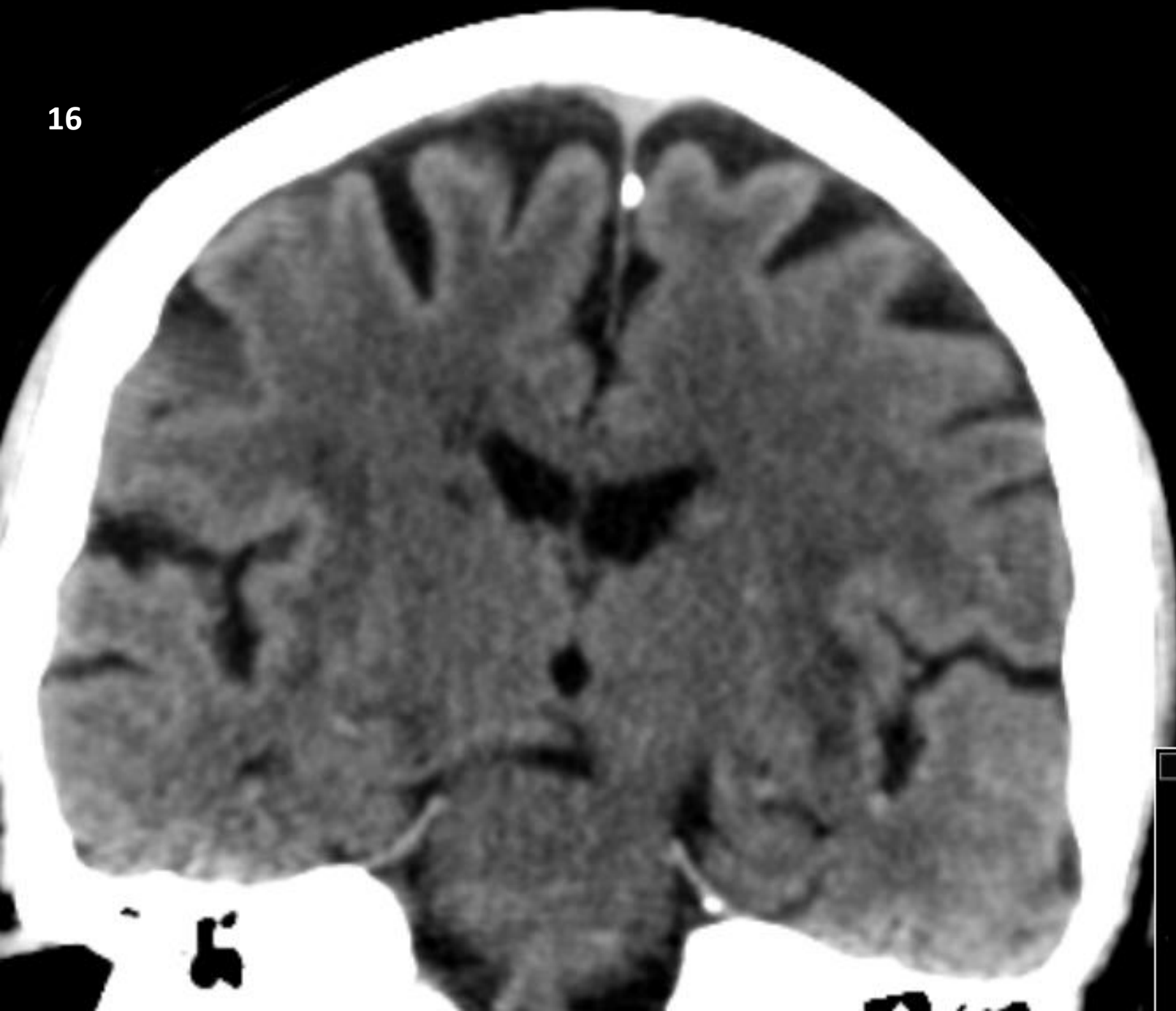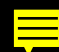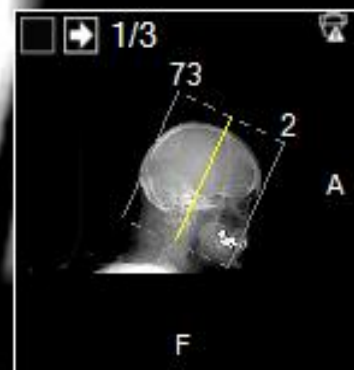

17

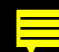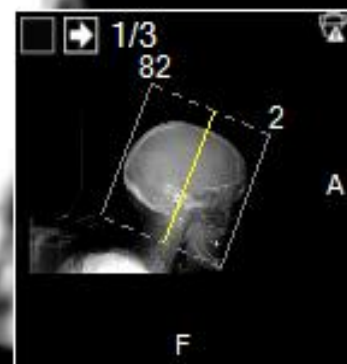

18

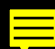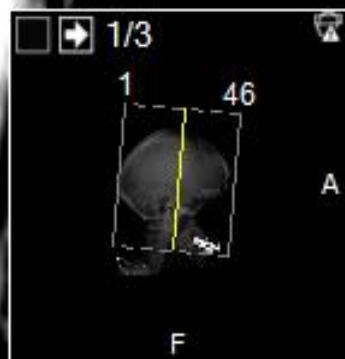

19

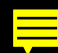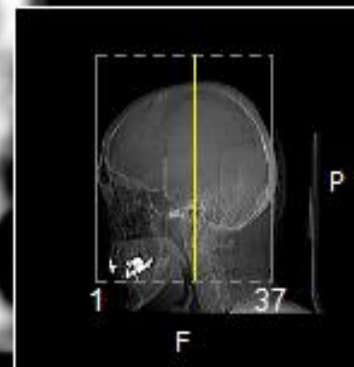

20

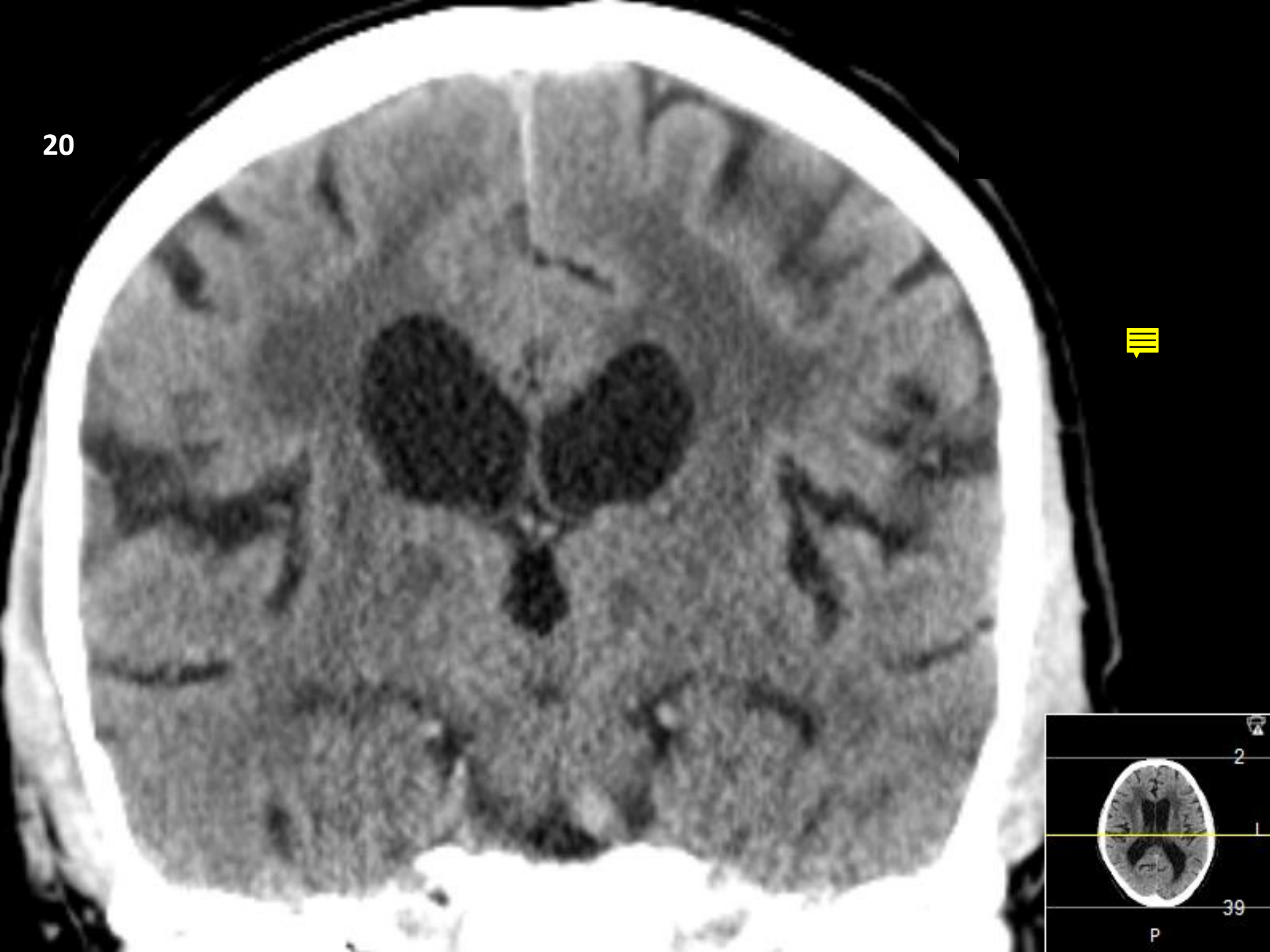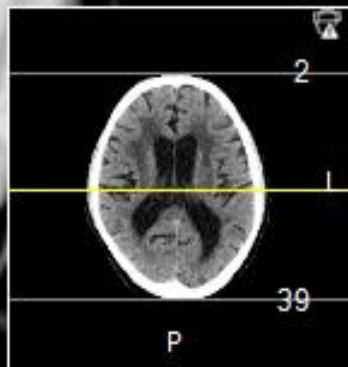

Supplement: Supplementary file 4 — (PDF 998 kb) [file 13244_2016_521_MOESM4_ESM.pdf]

1

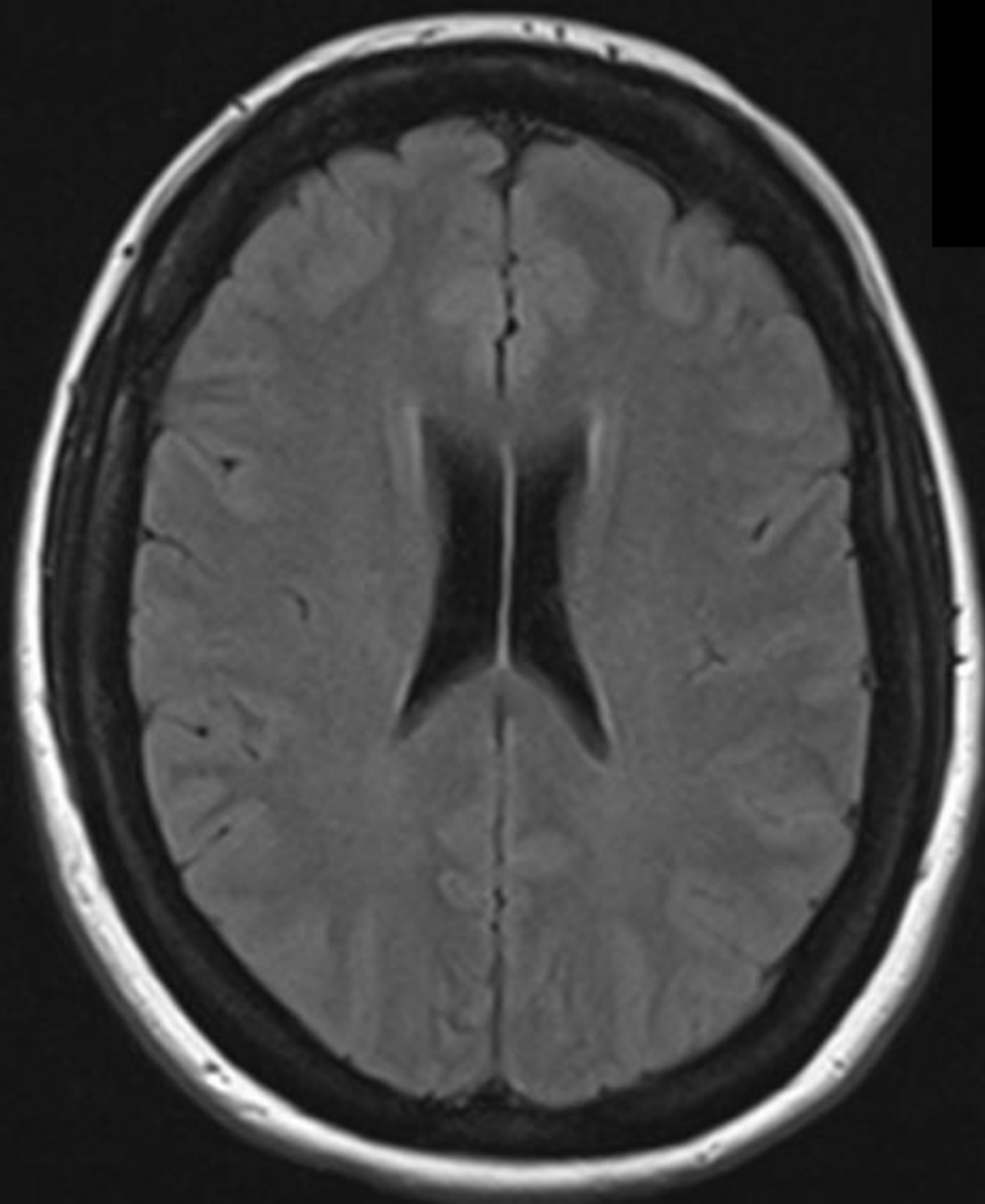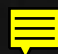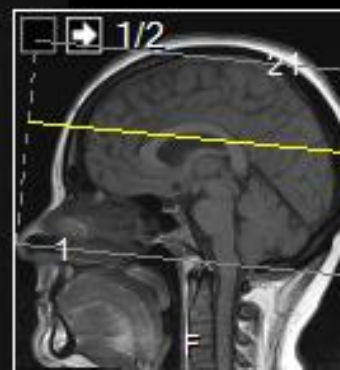

2

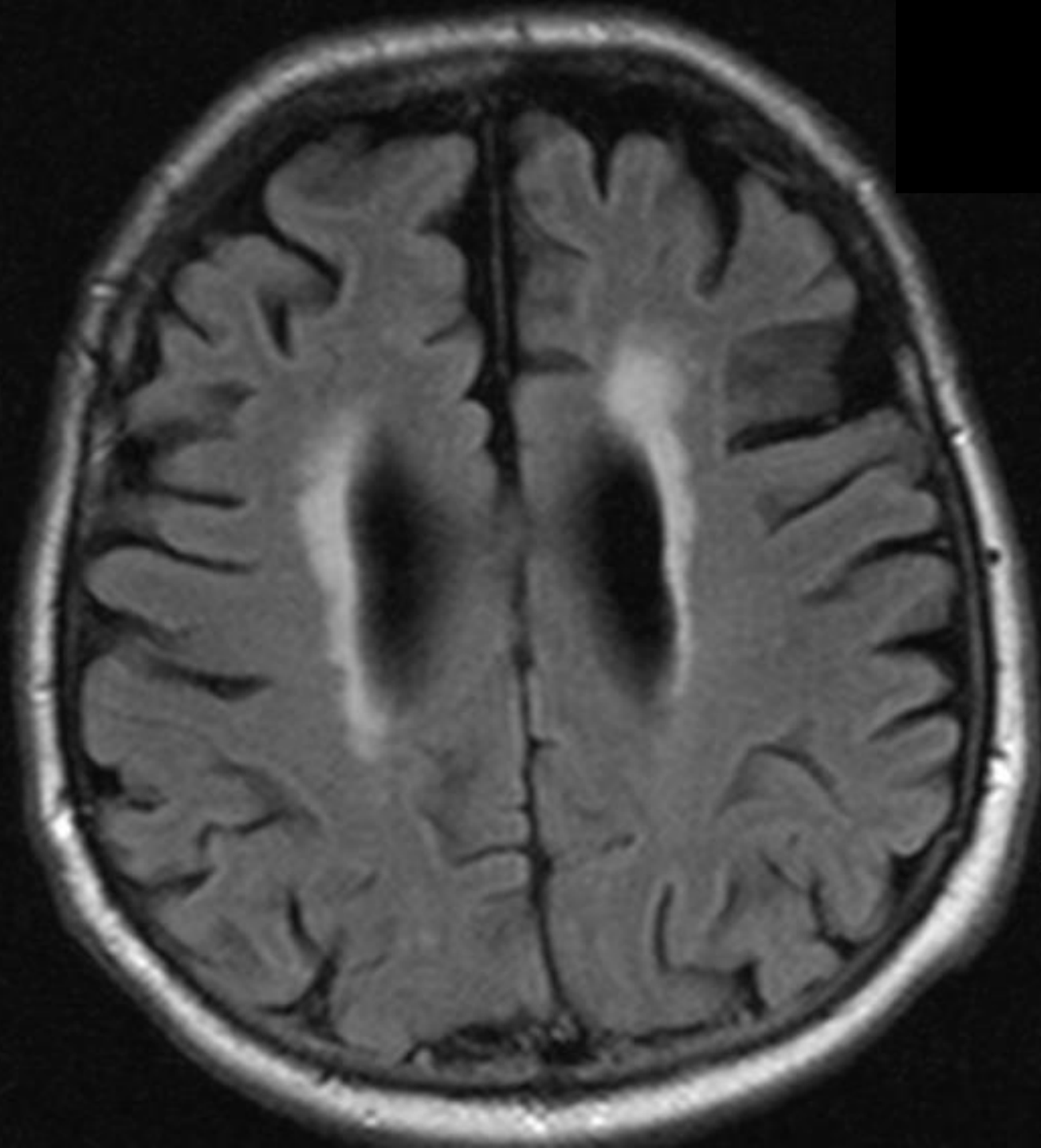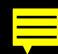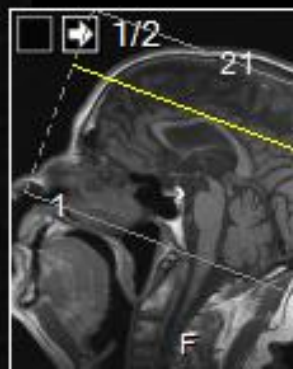

3

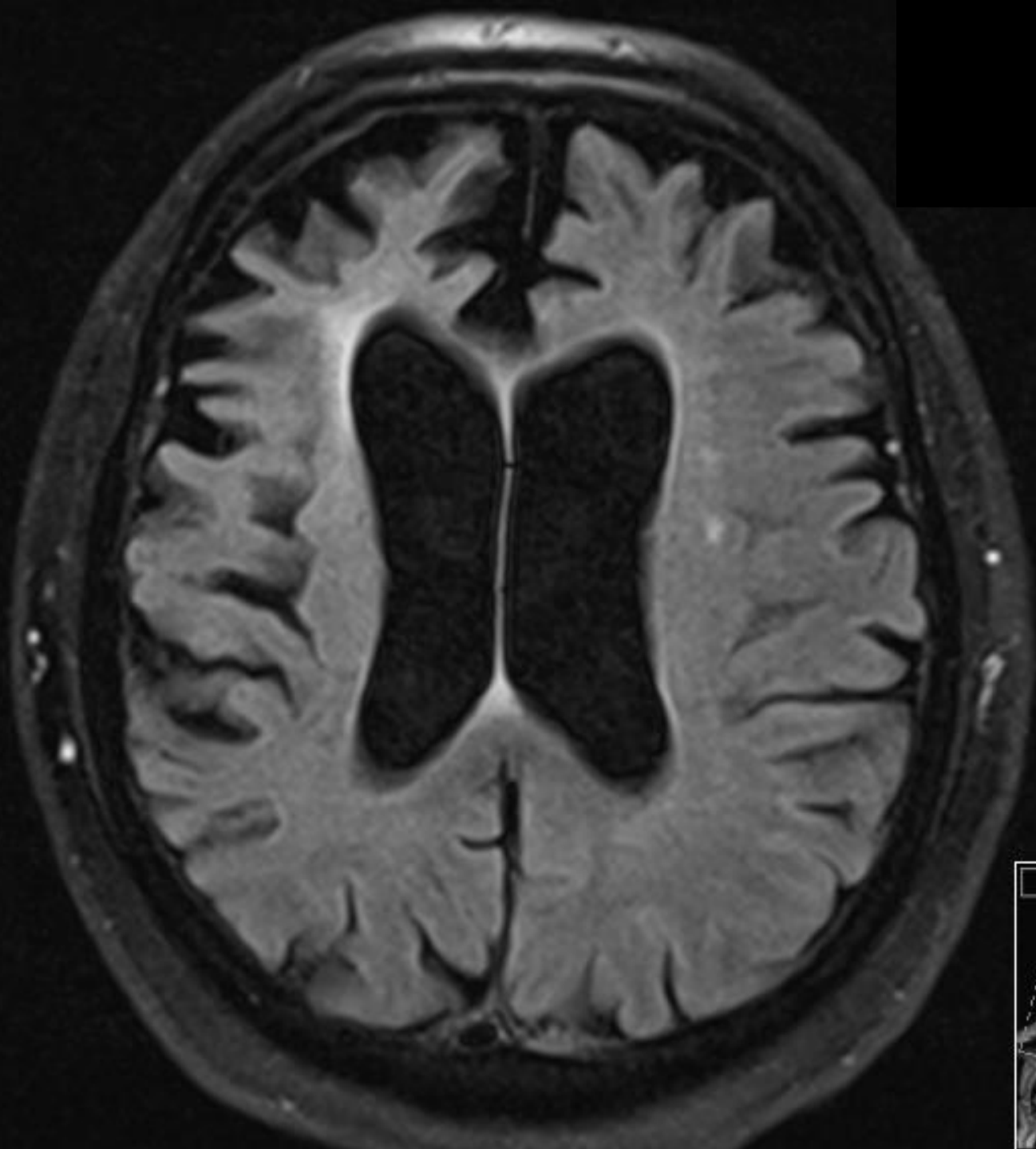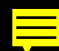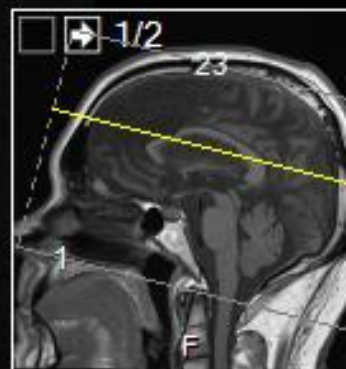

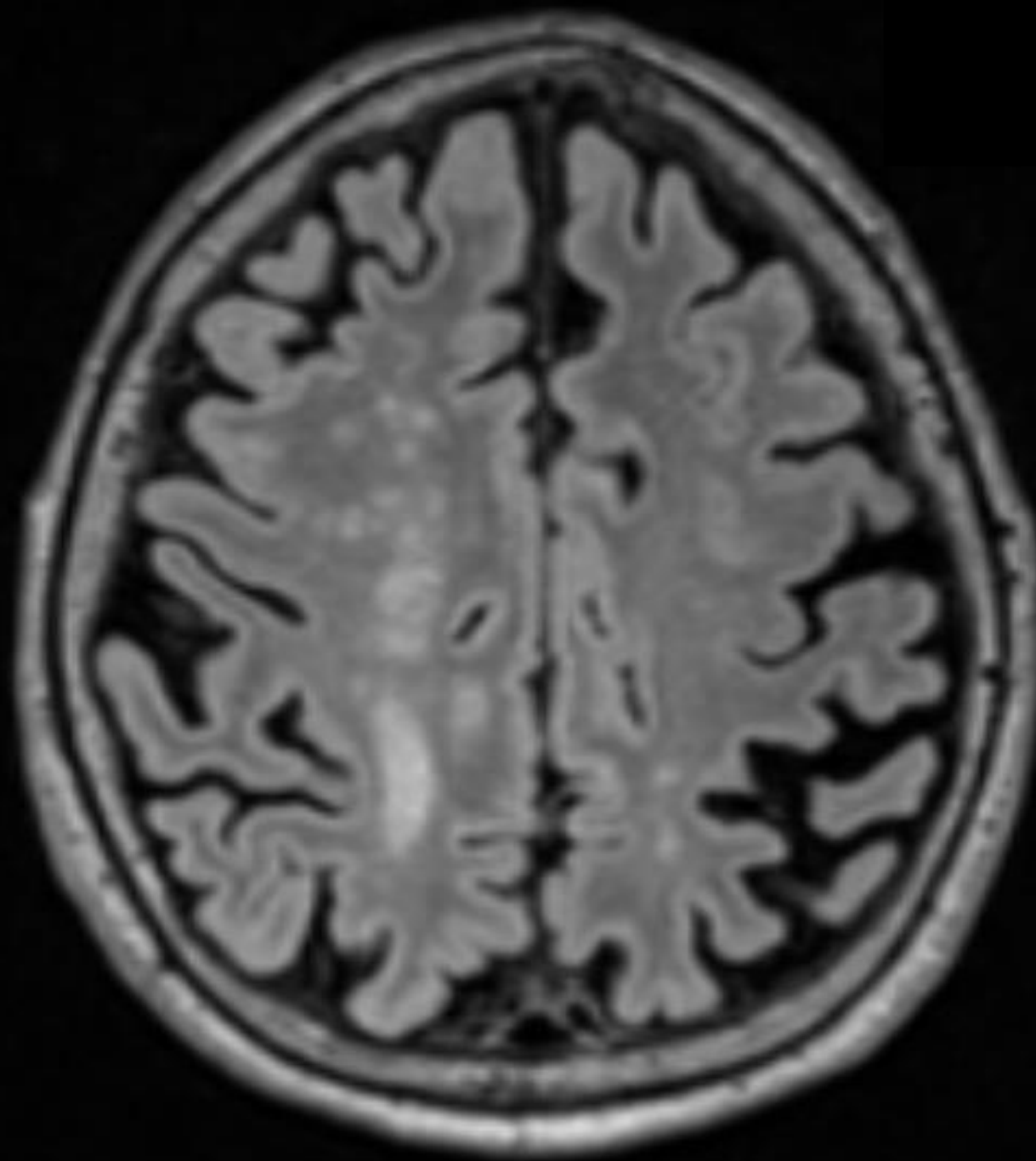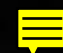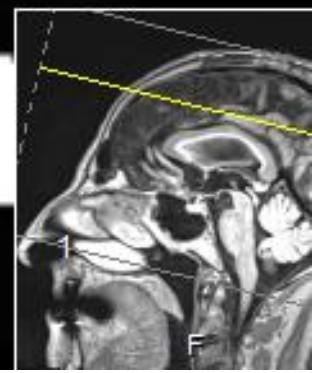

5

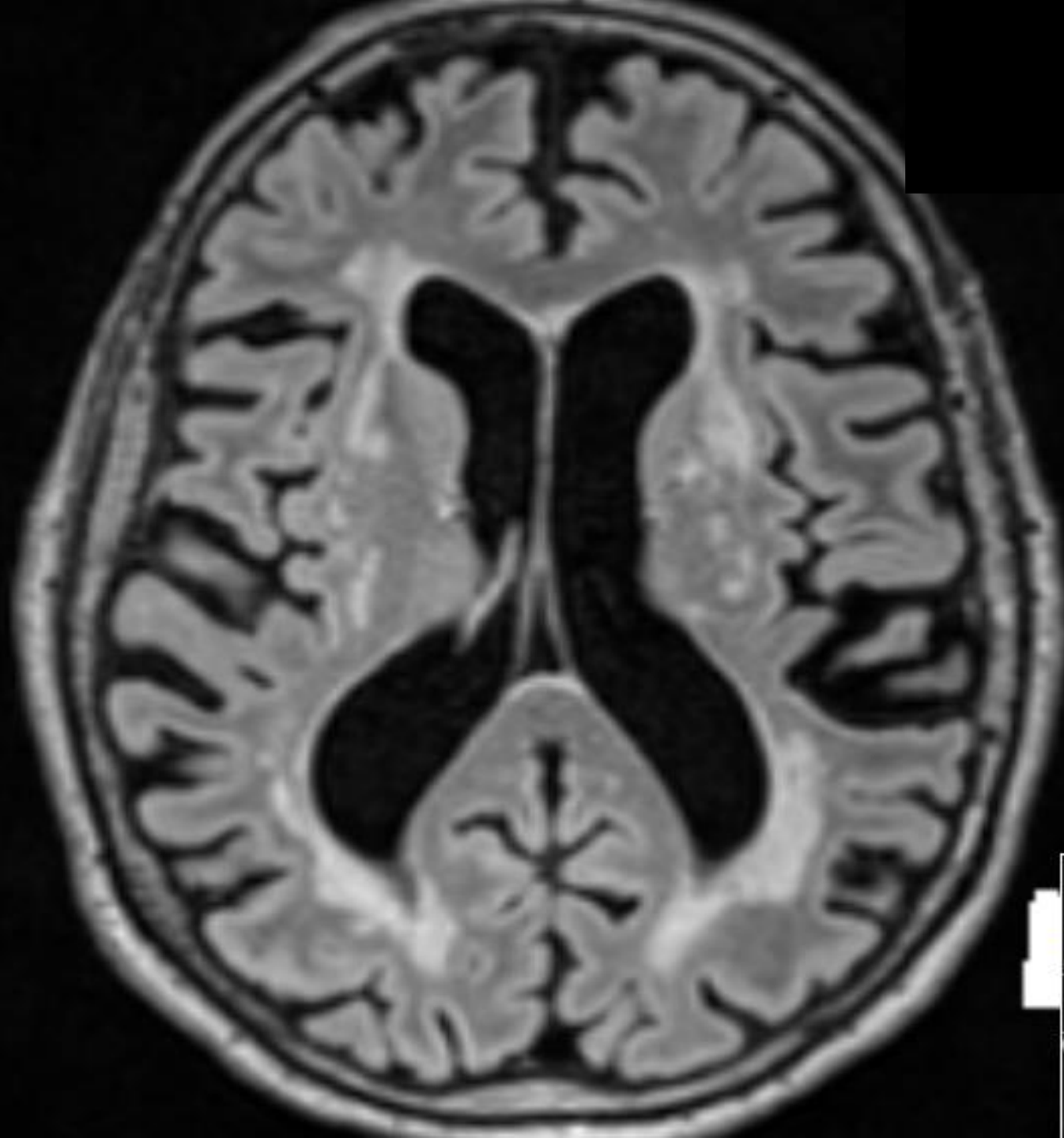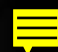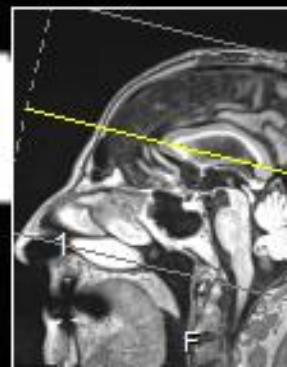

6

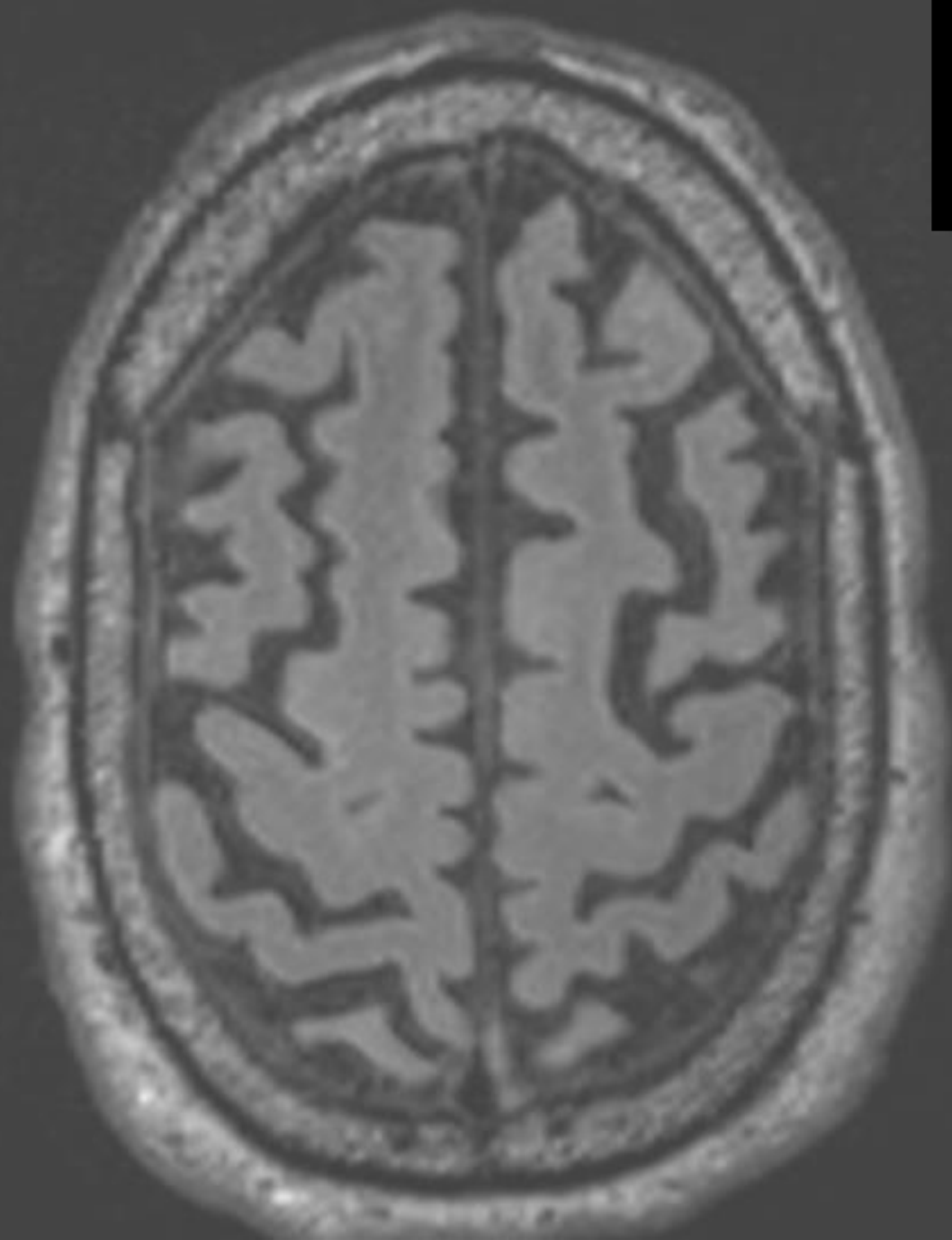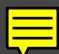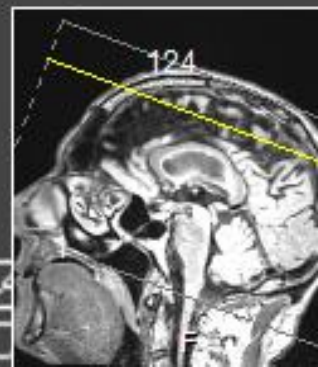

7

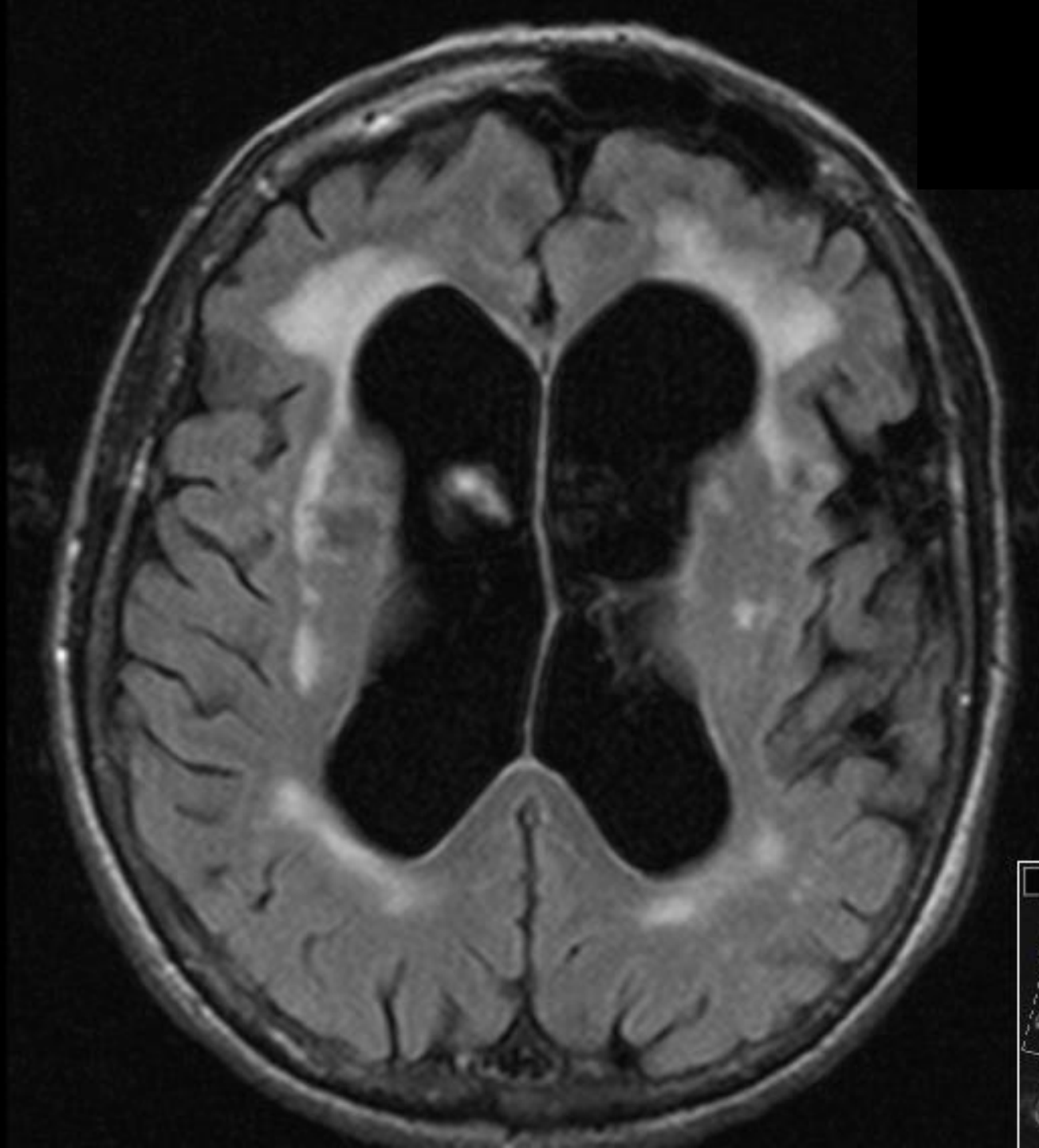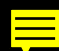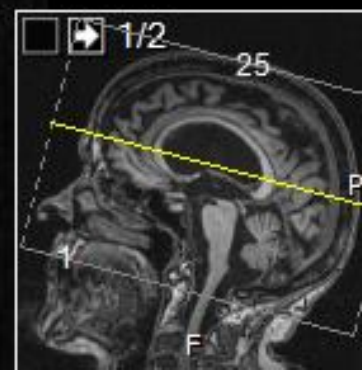

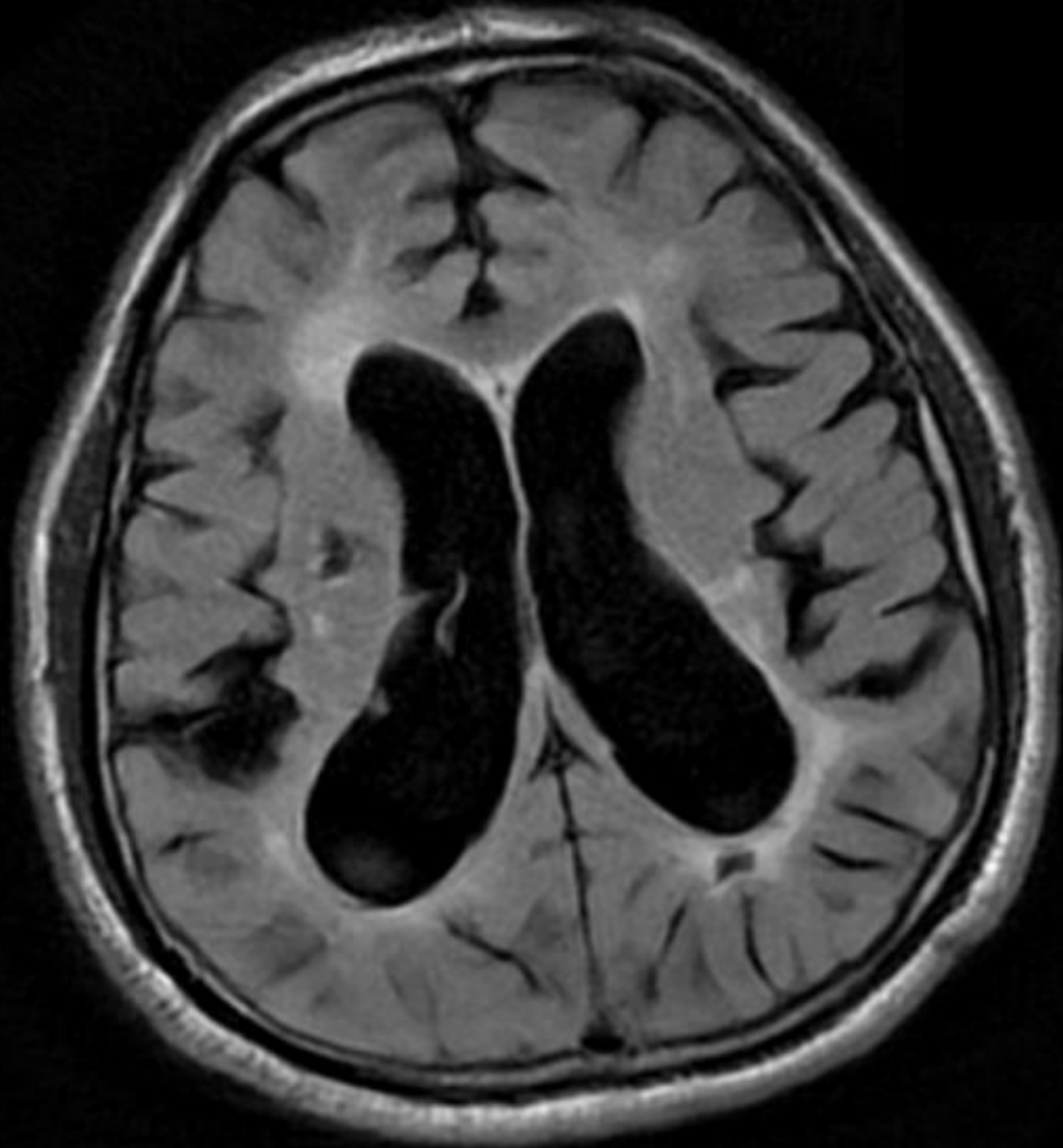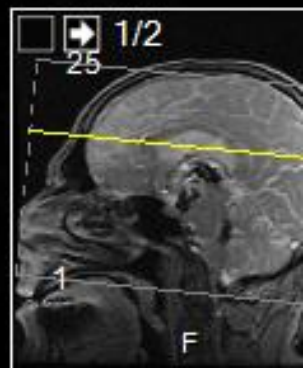

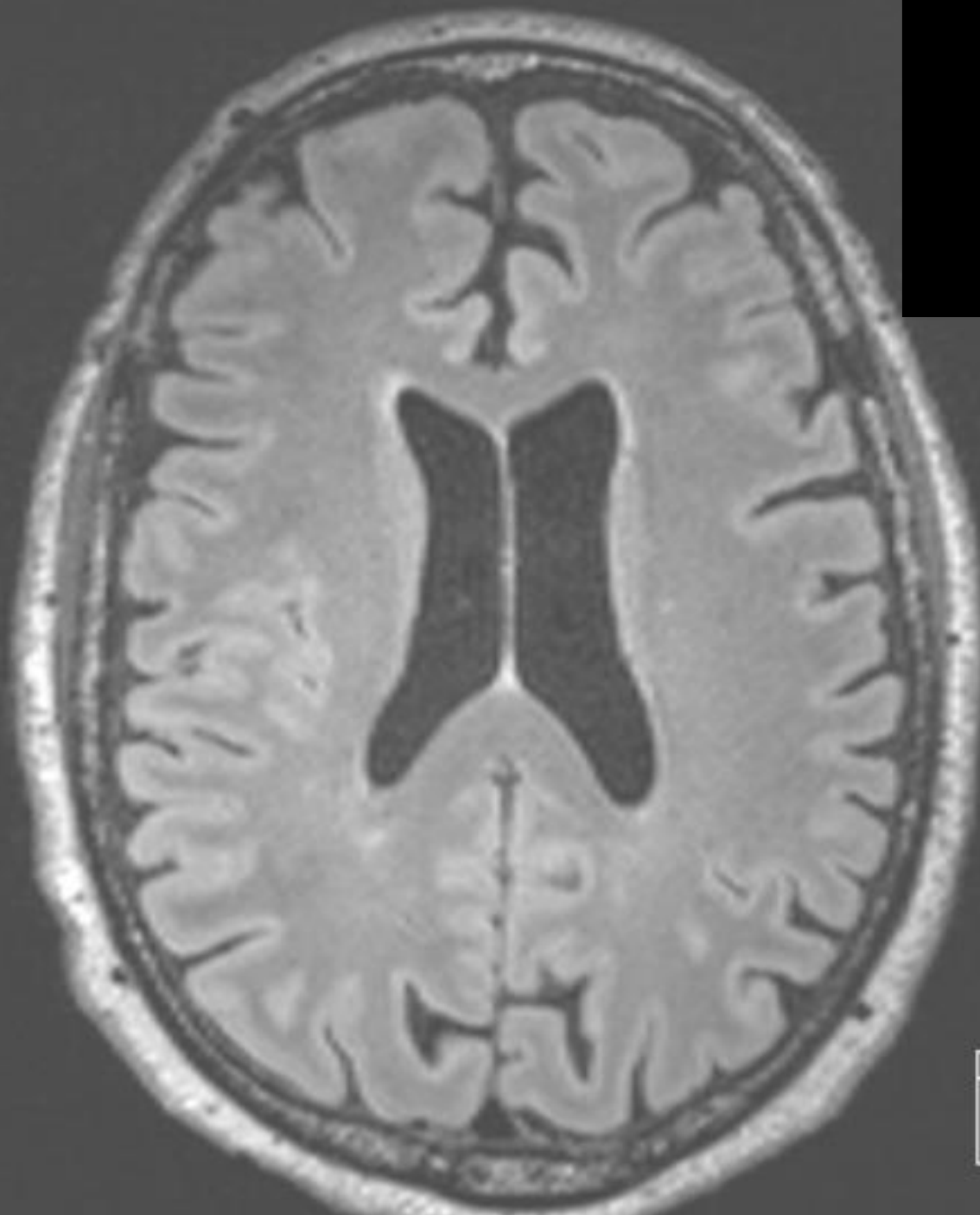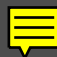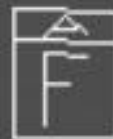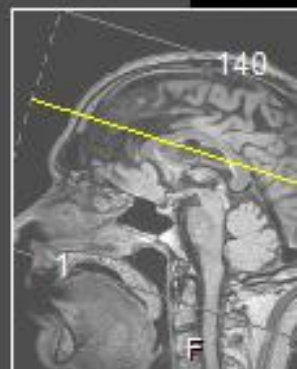

10

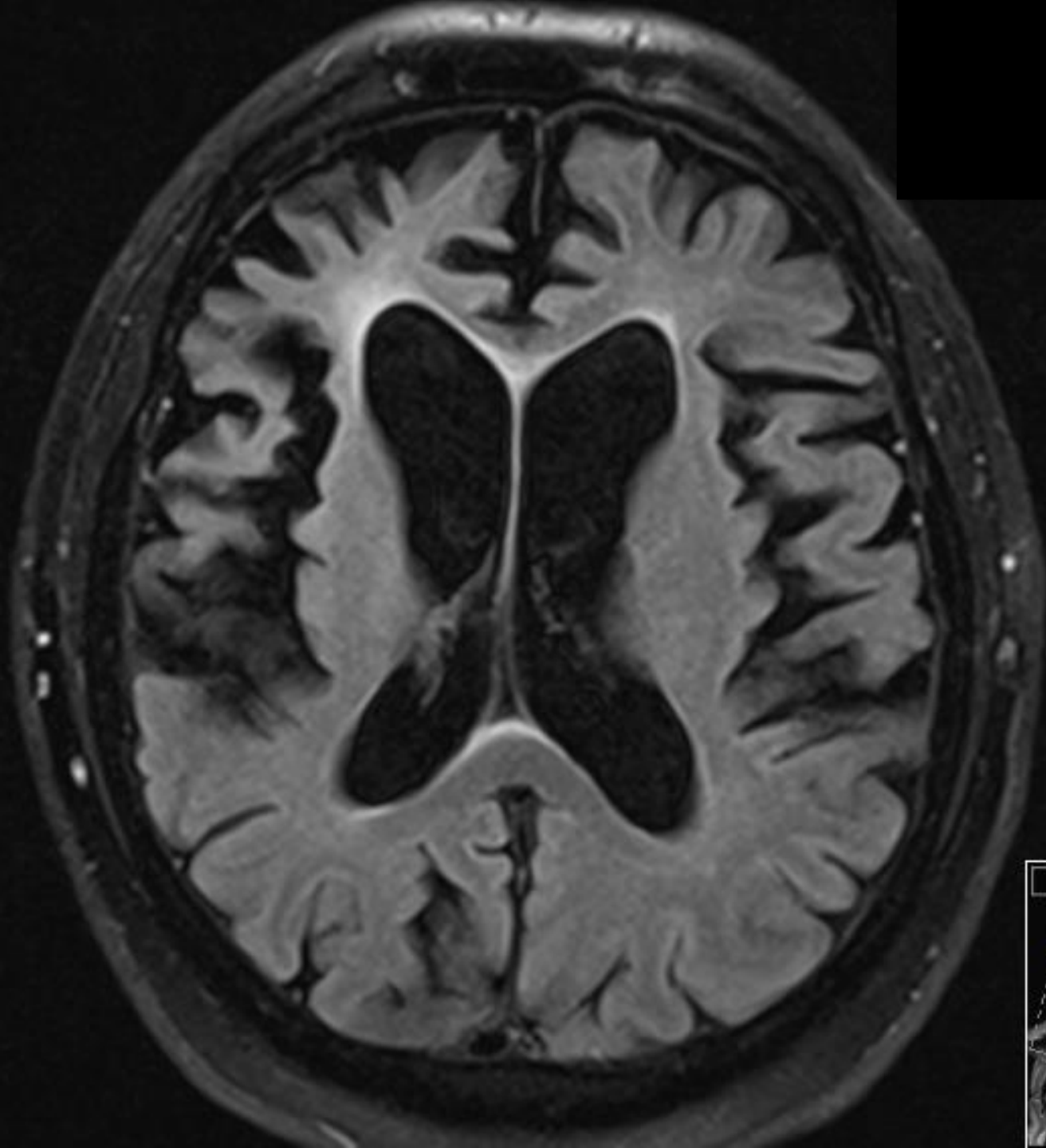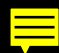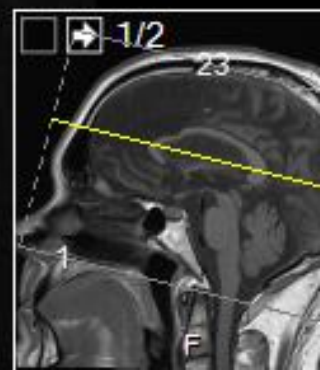

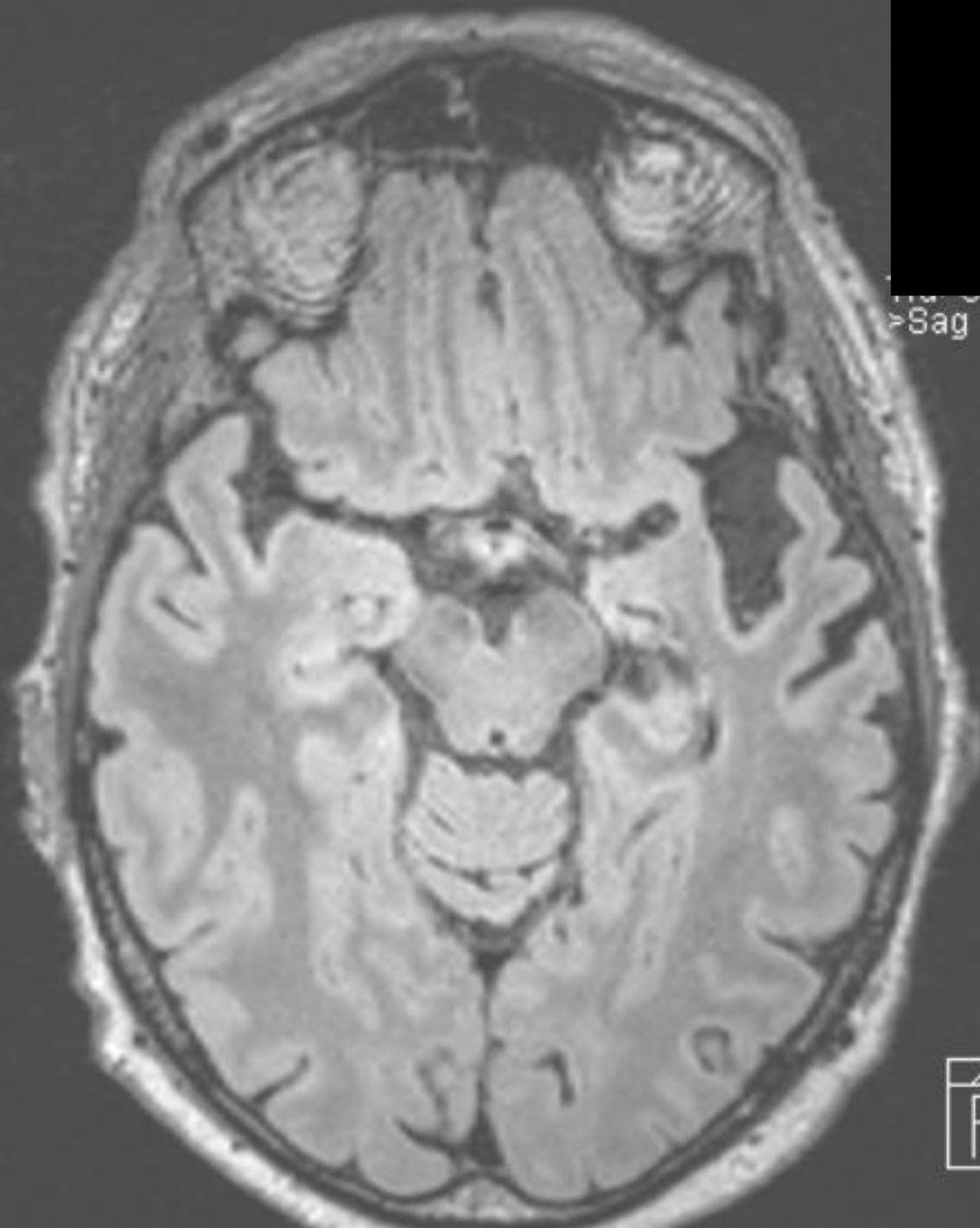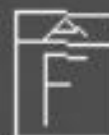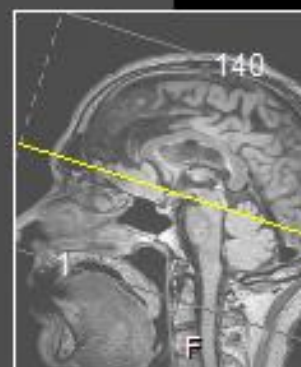

12

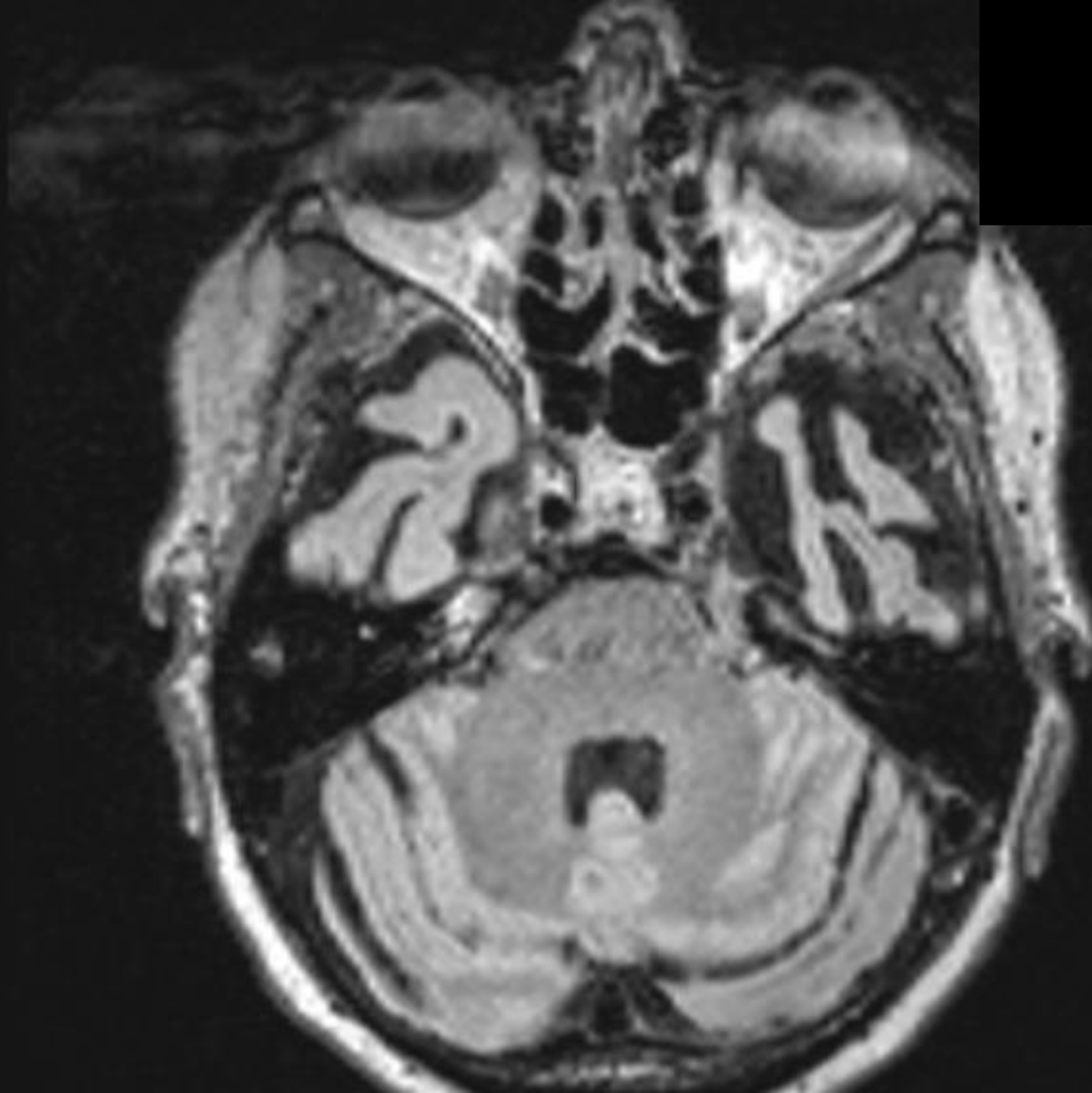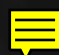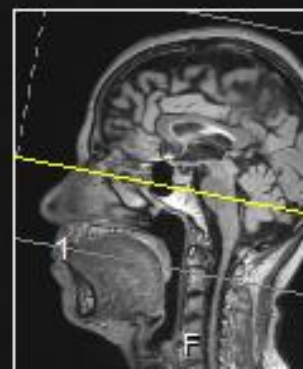

13

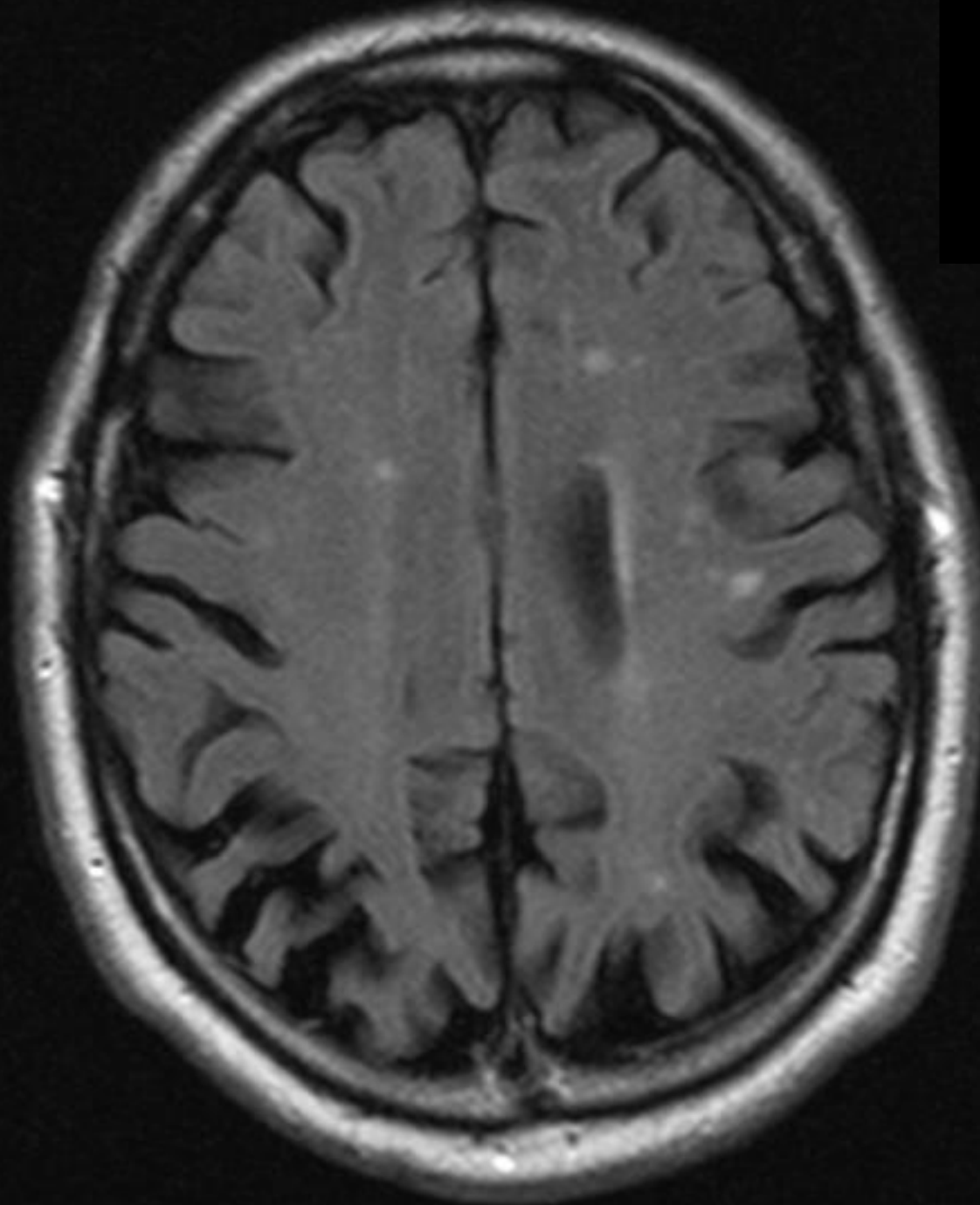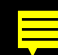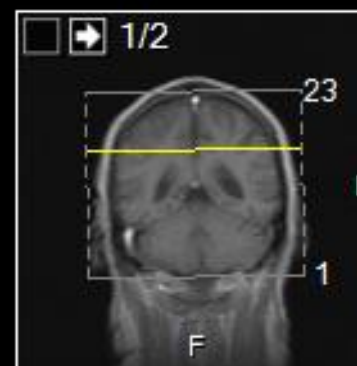

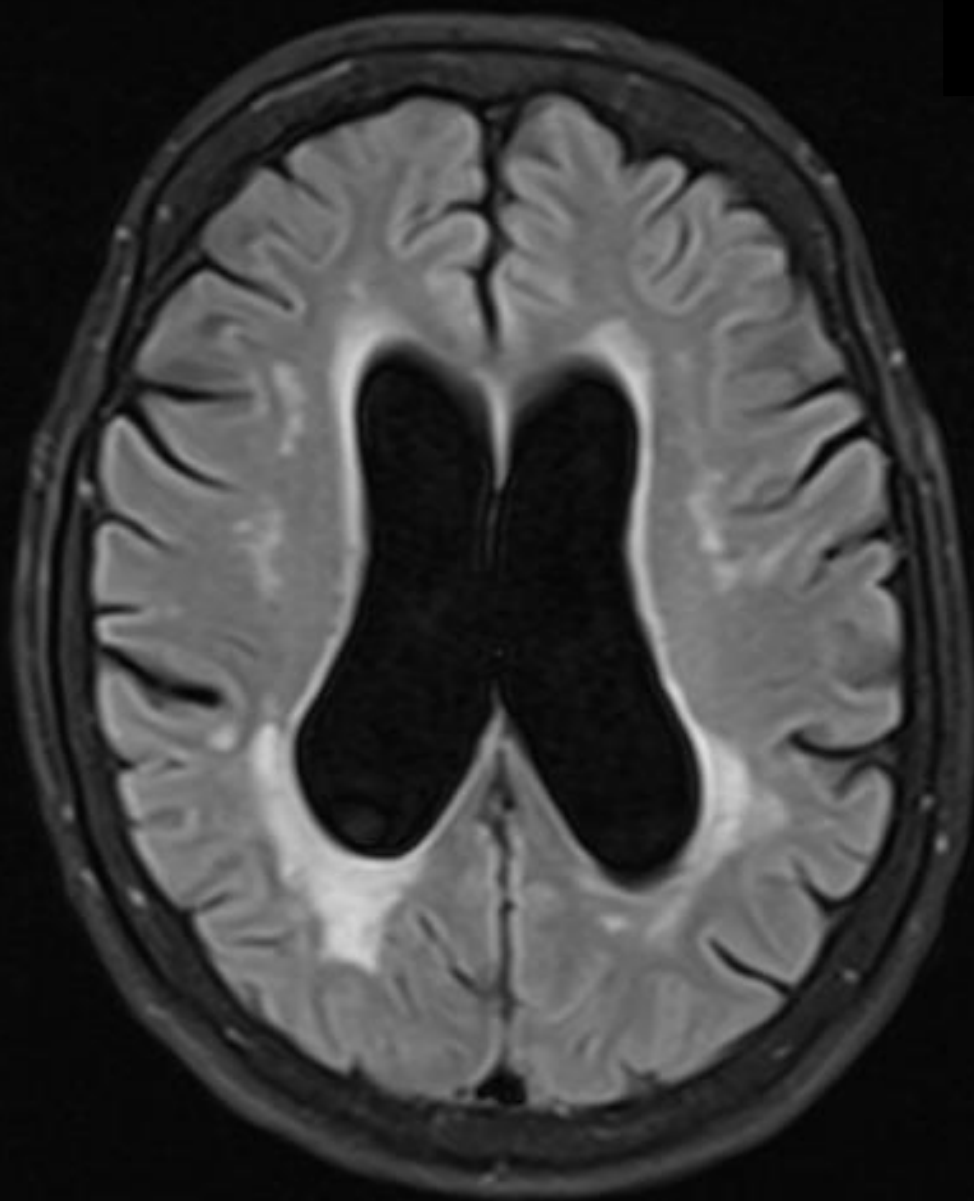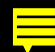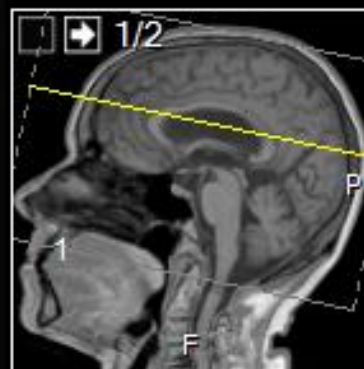

15

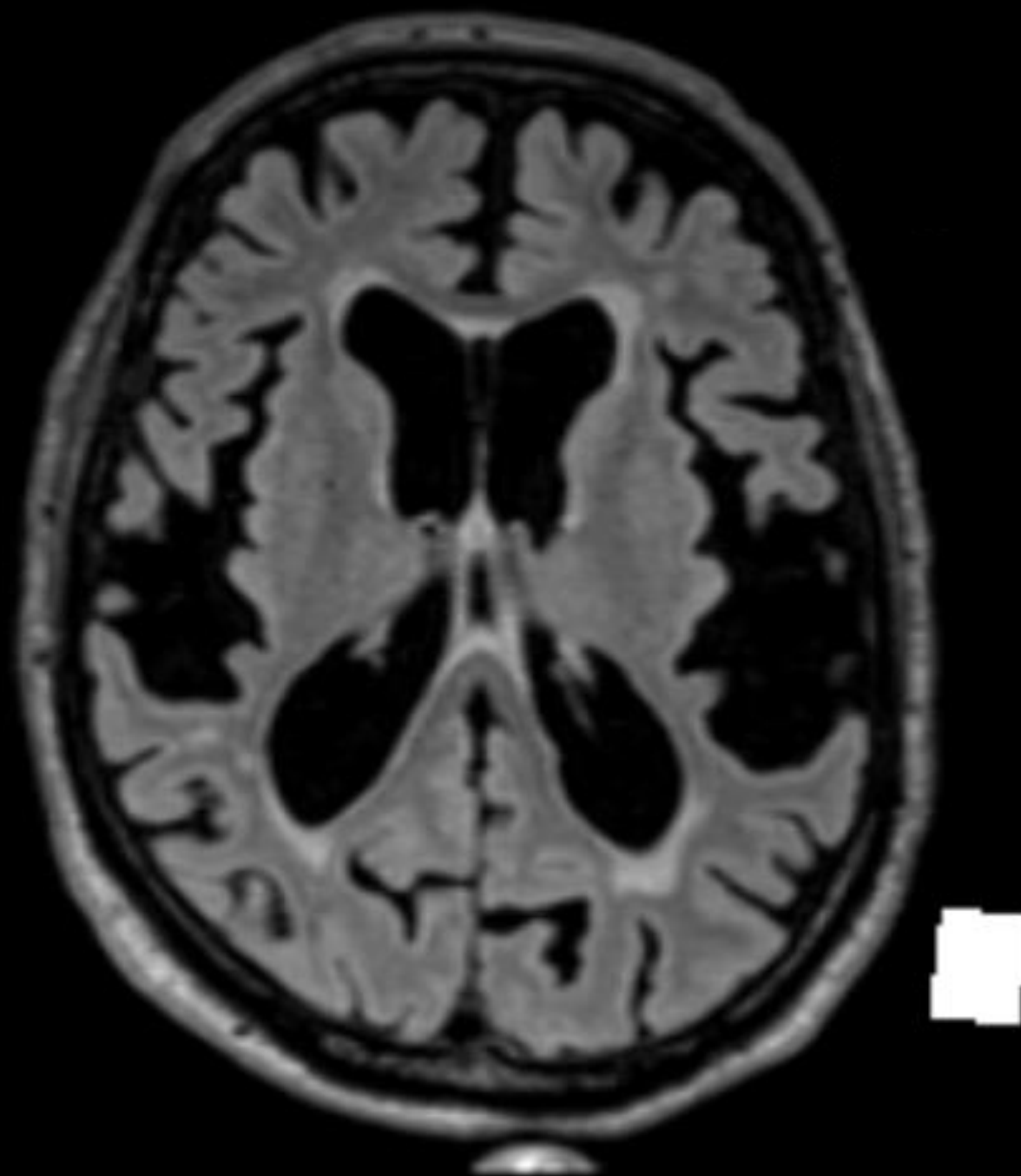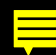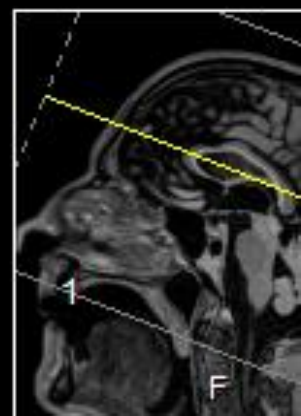

16

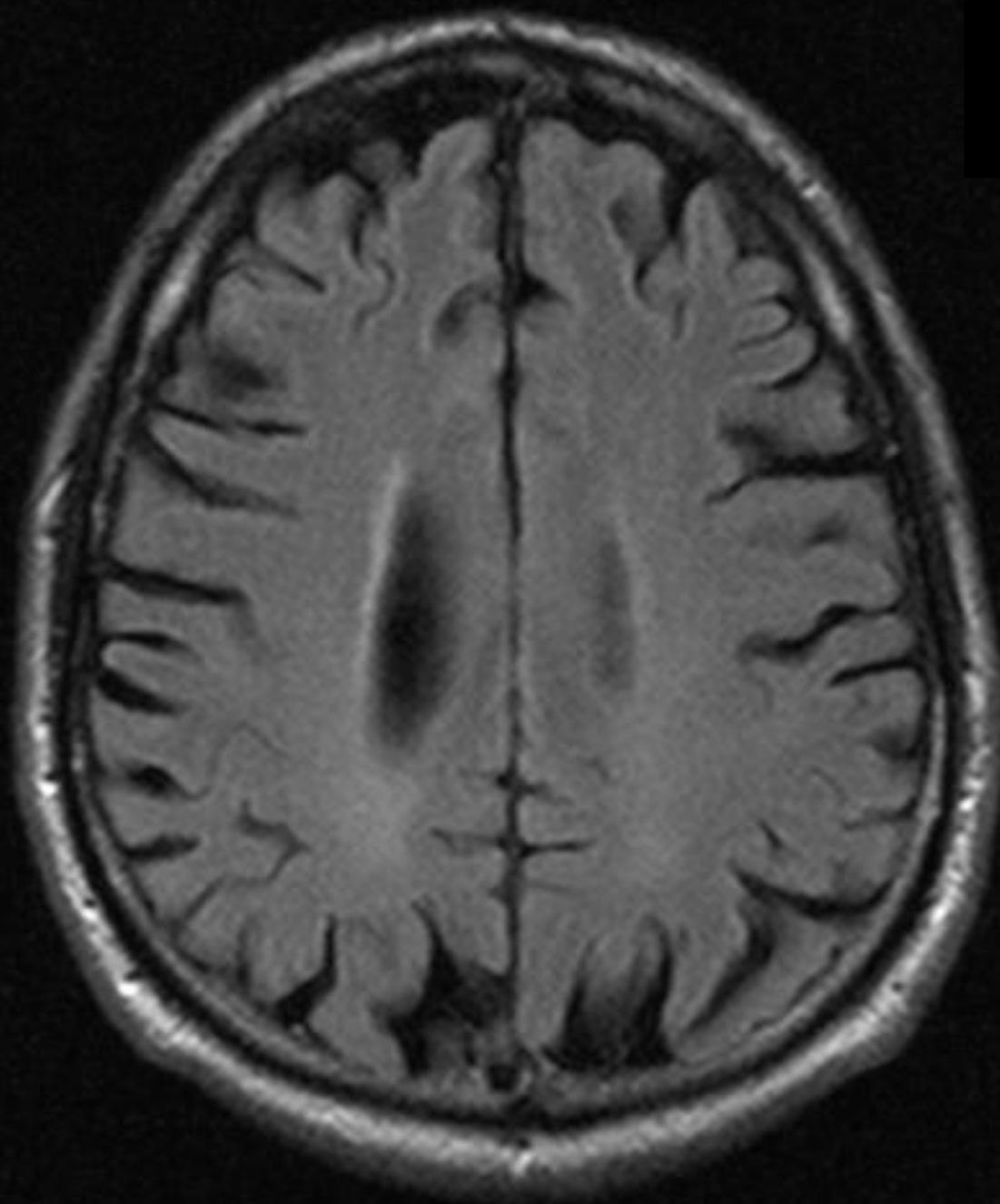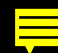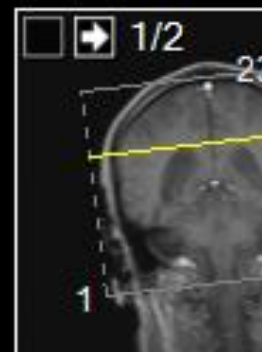

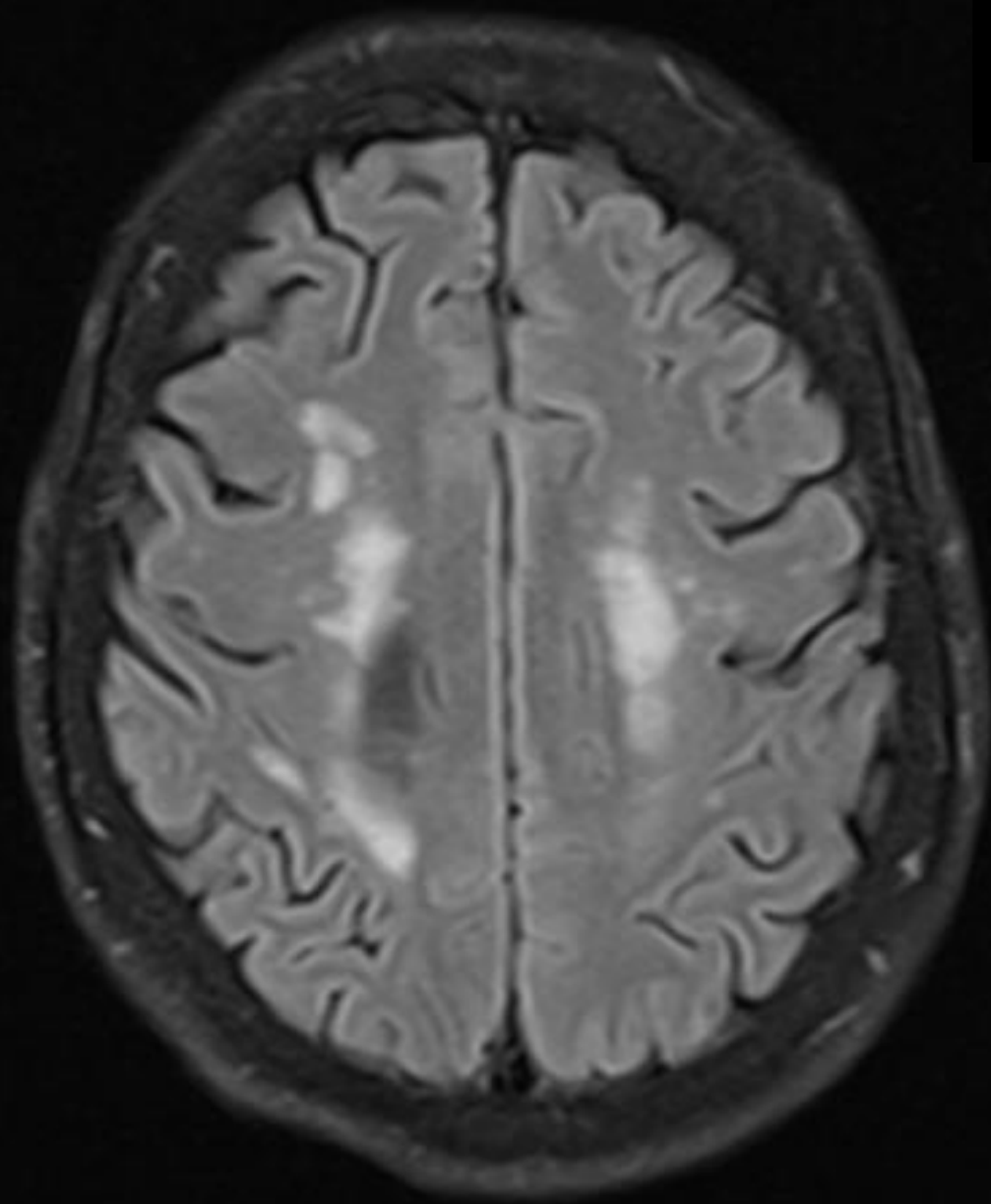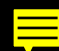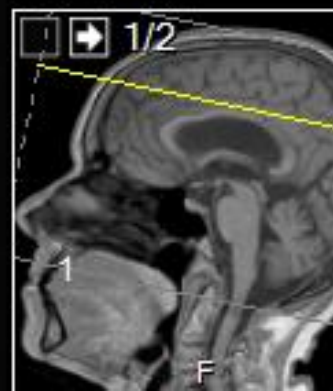

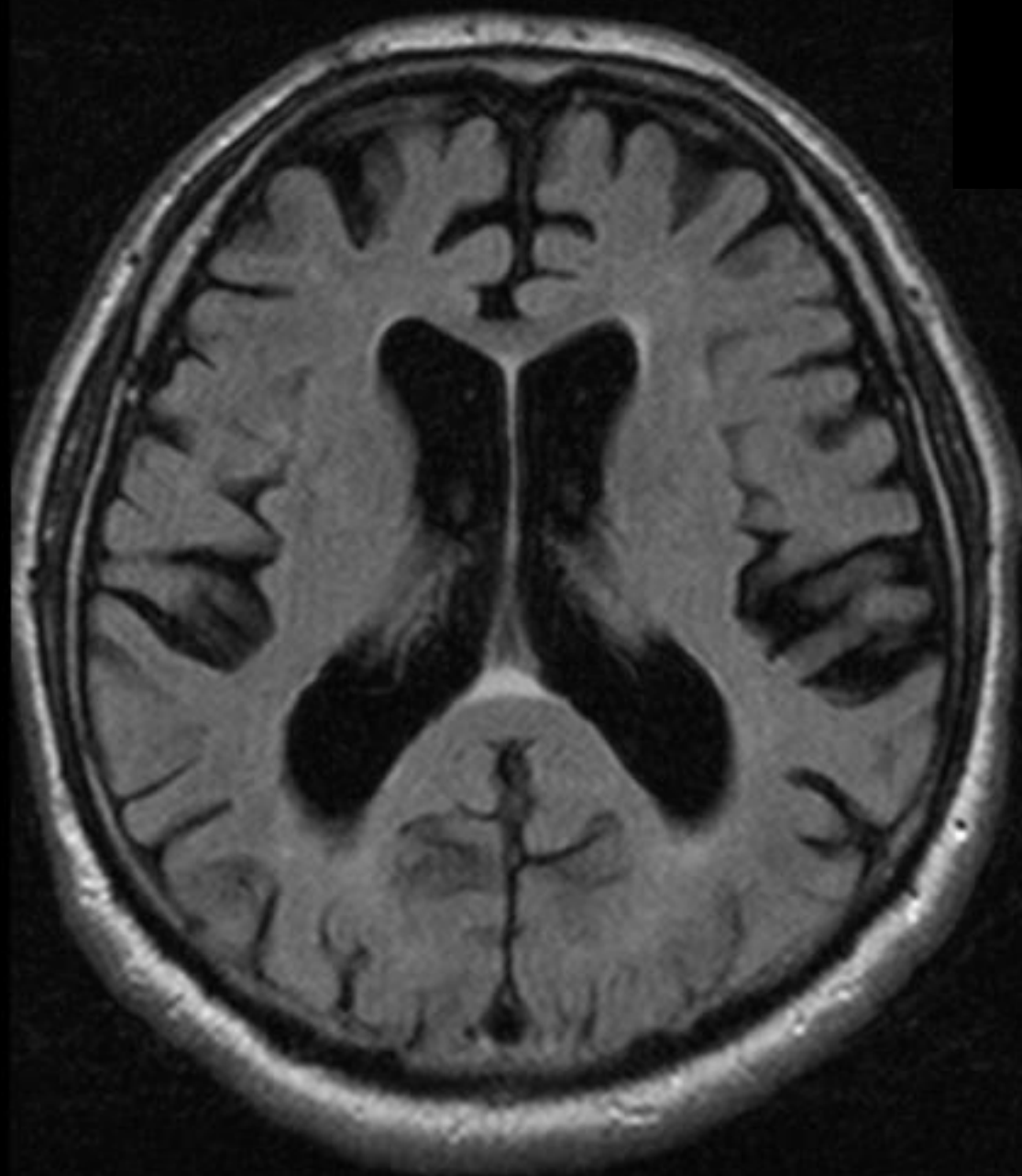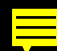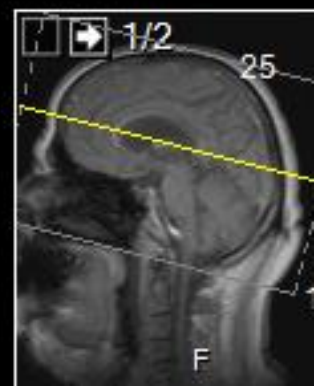

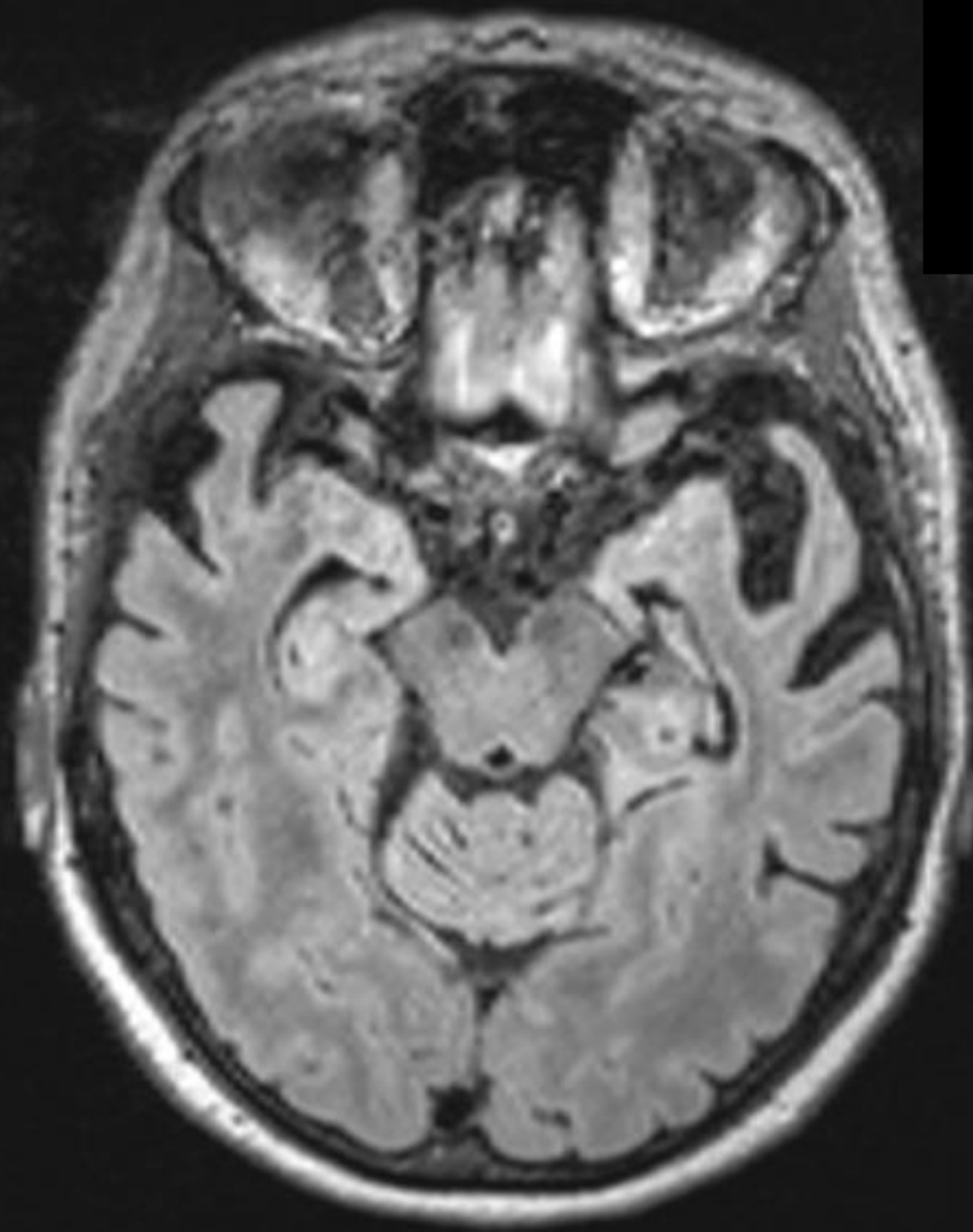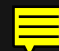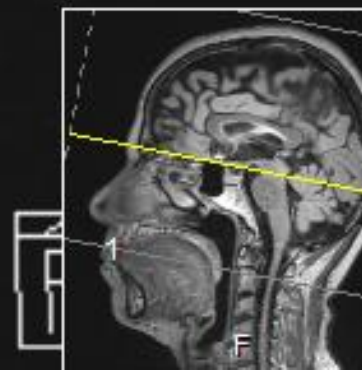

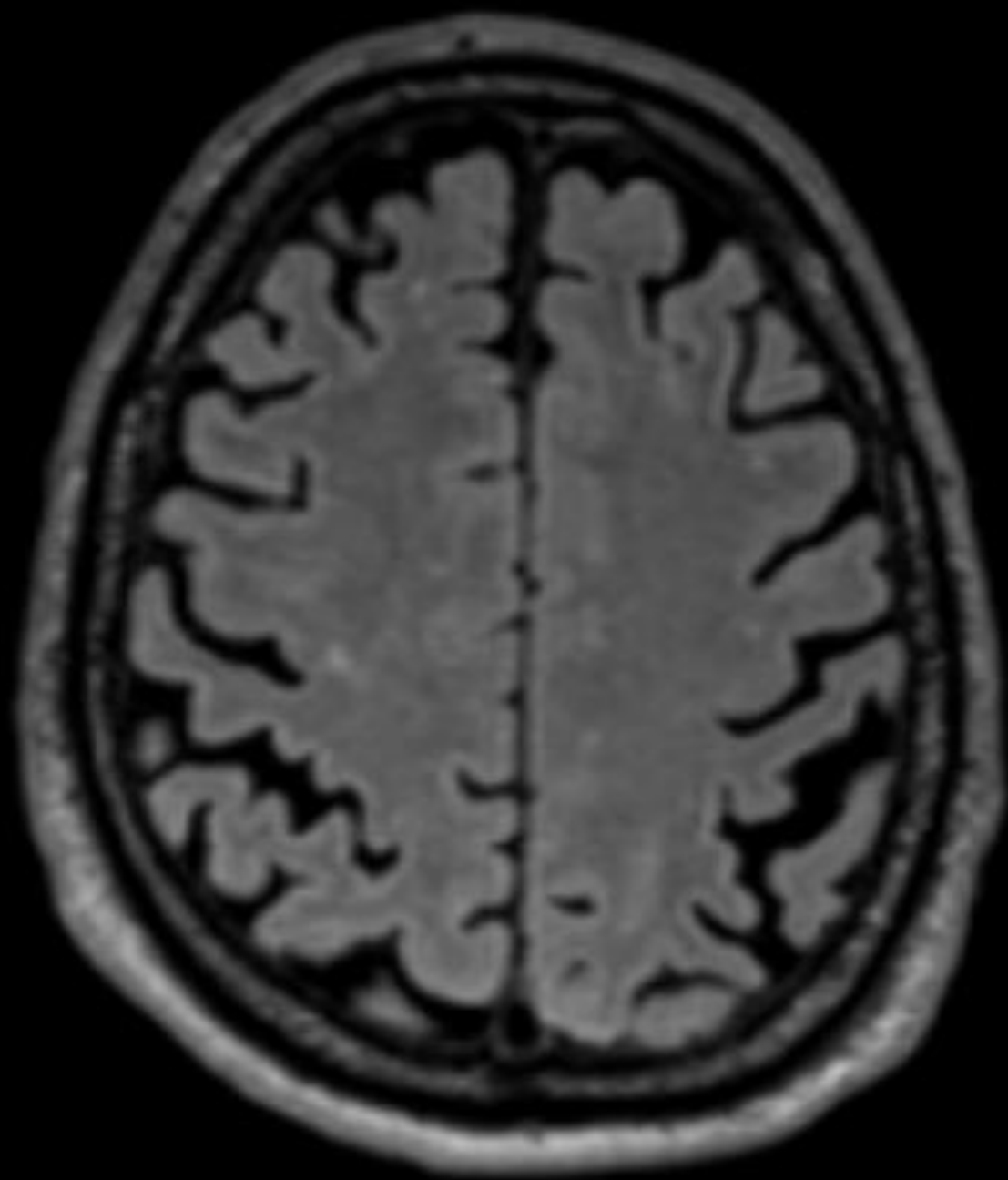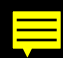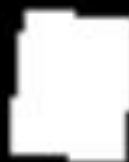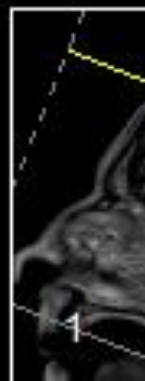

Supplement: Supplementary file 5 — (PDF 882 kb) [file 13244_2016_521_MOESM5_ESM.pdf]

1

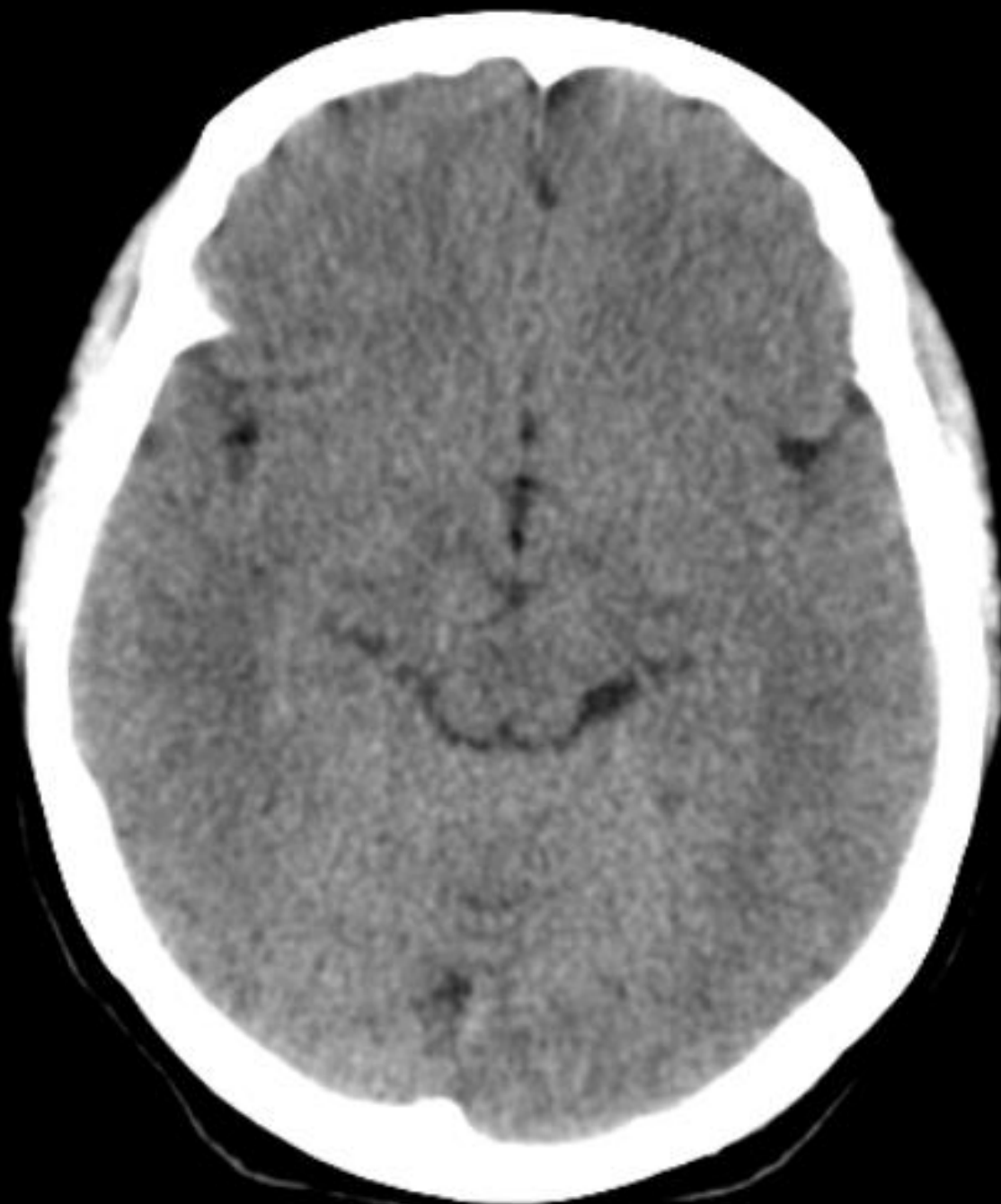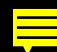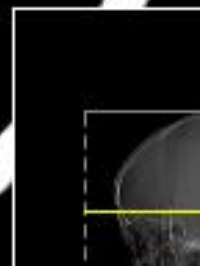

2

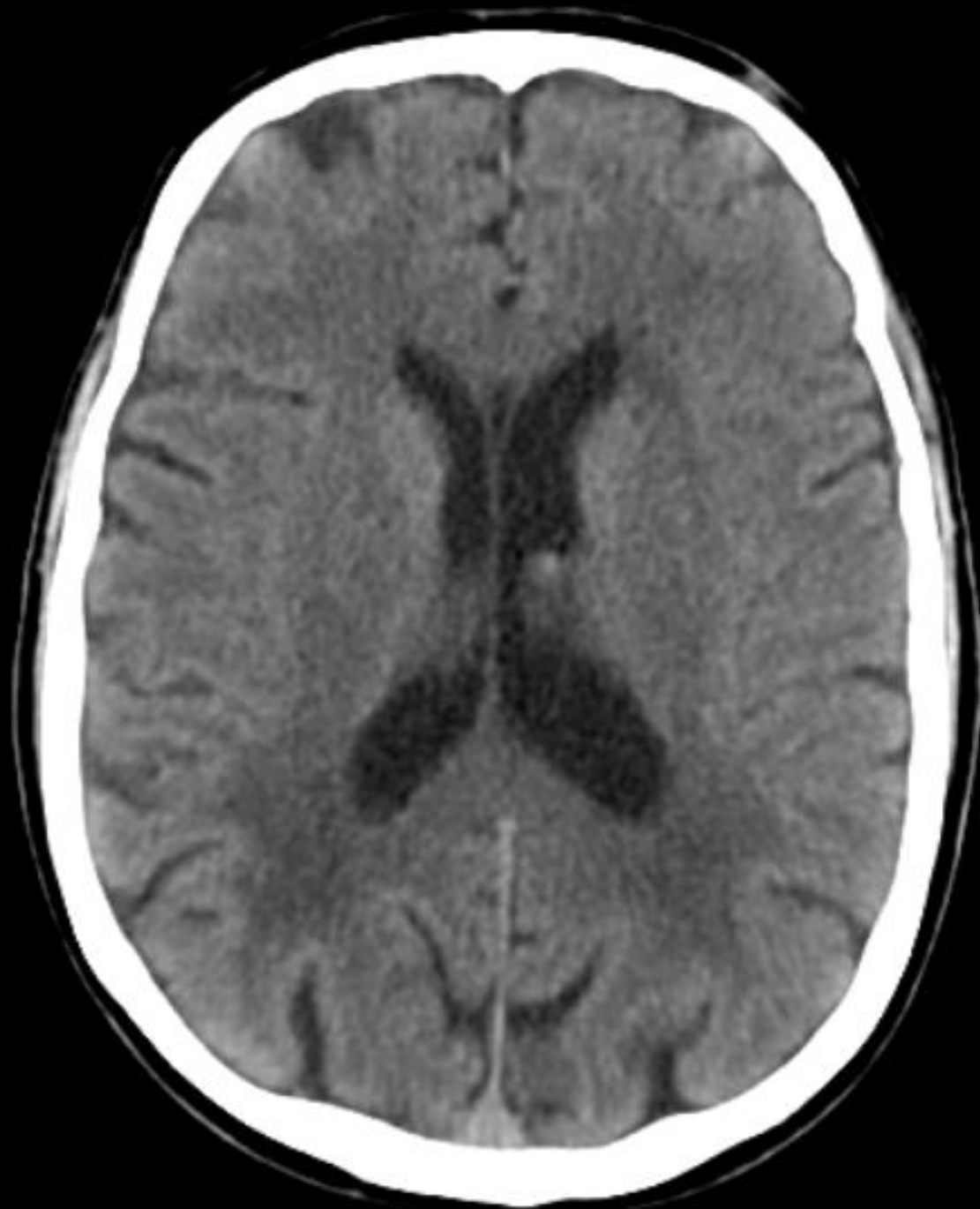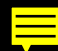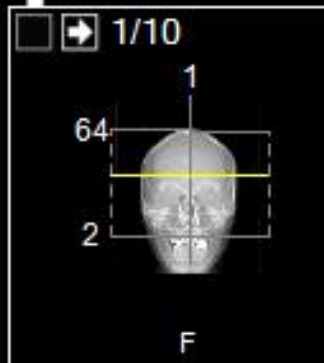

3

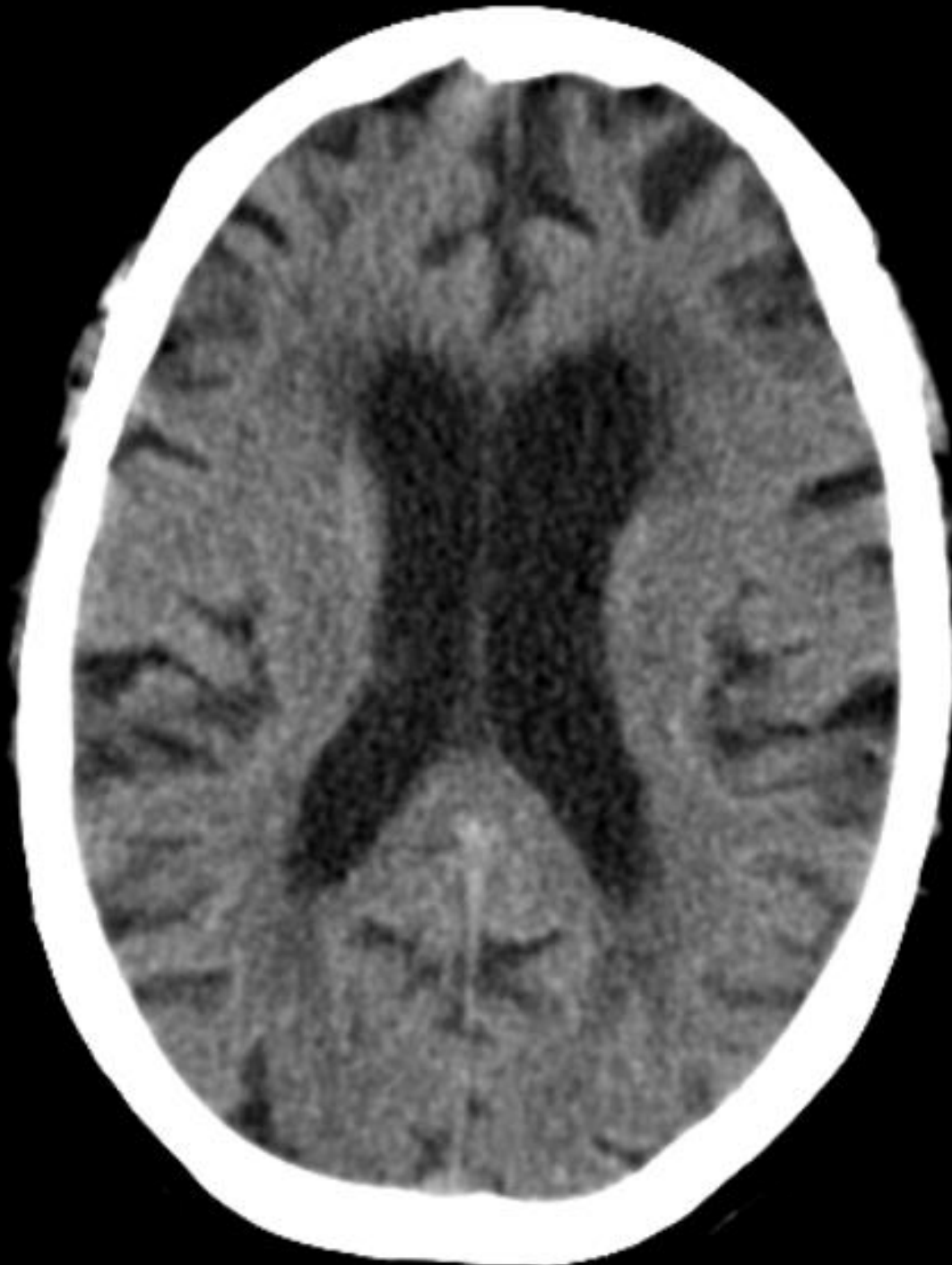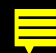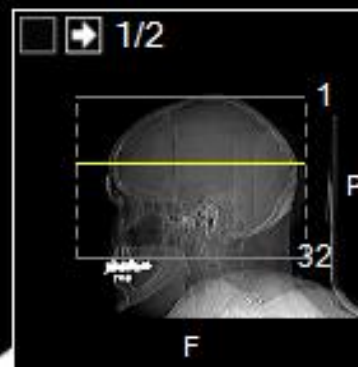

4

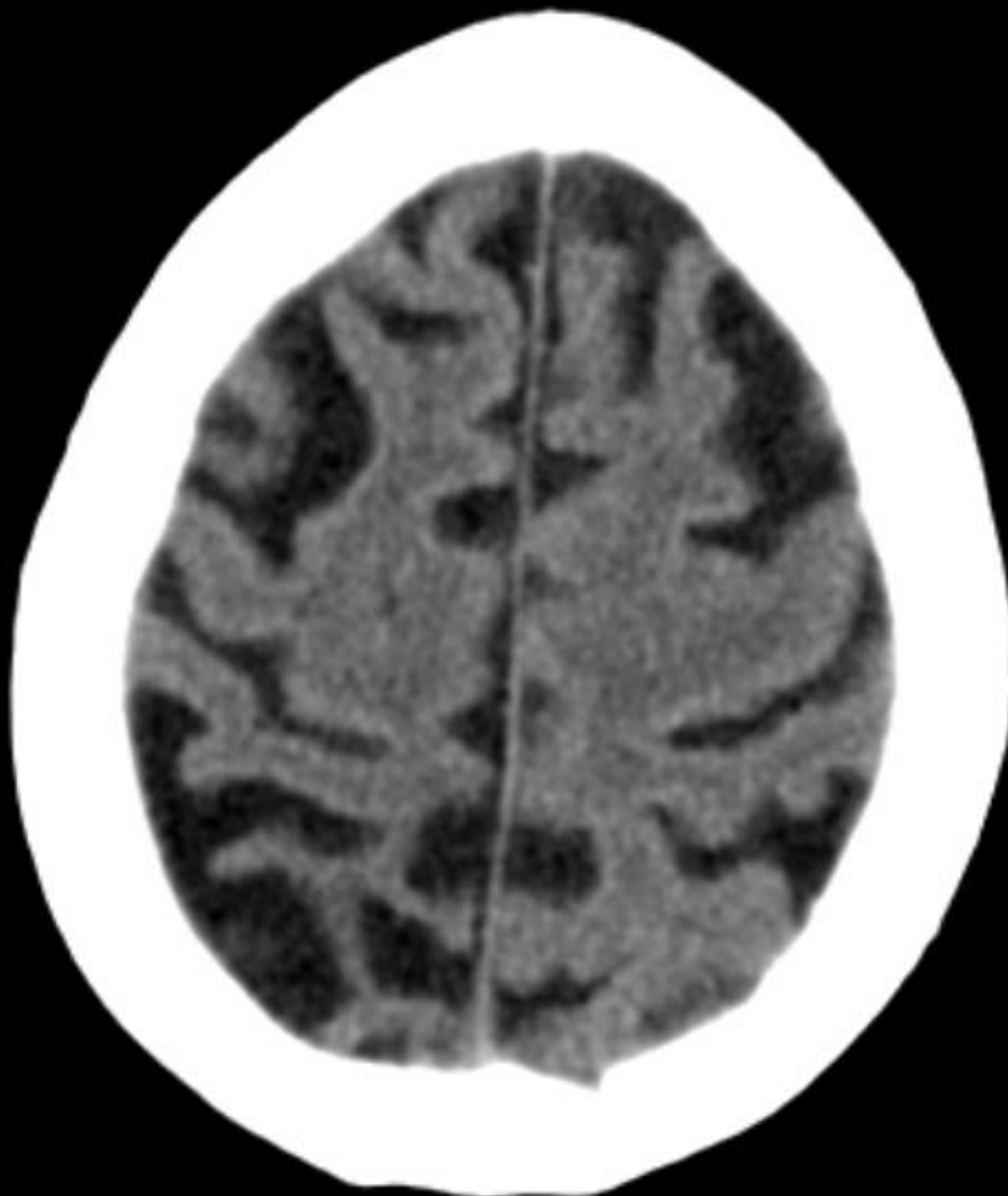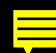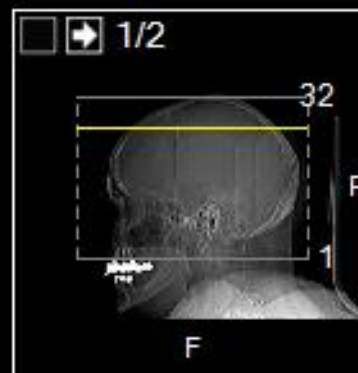

5

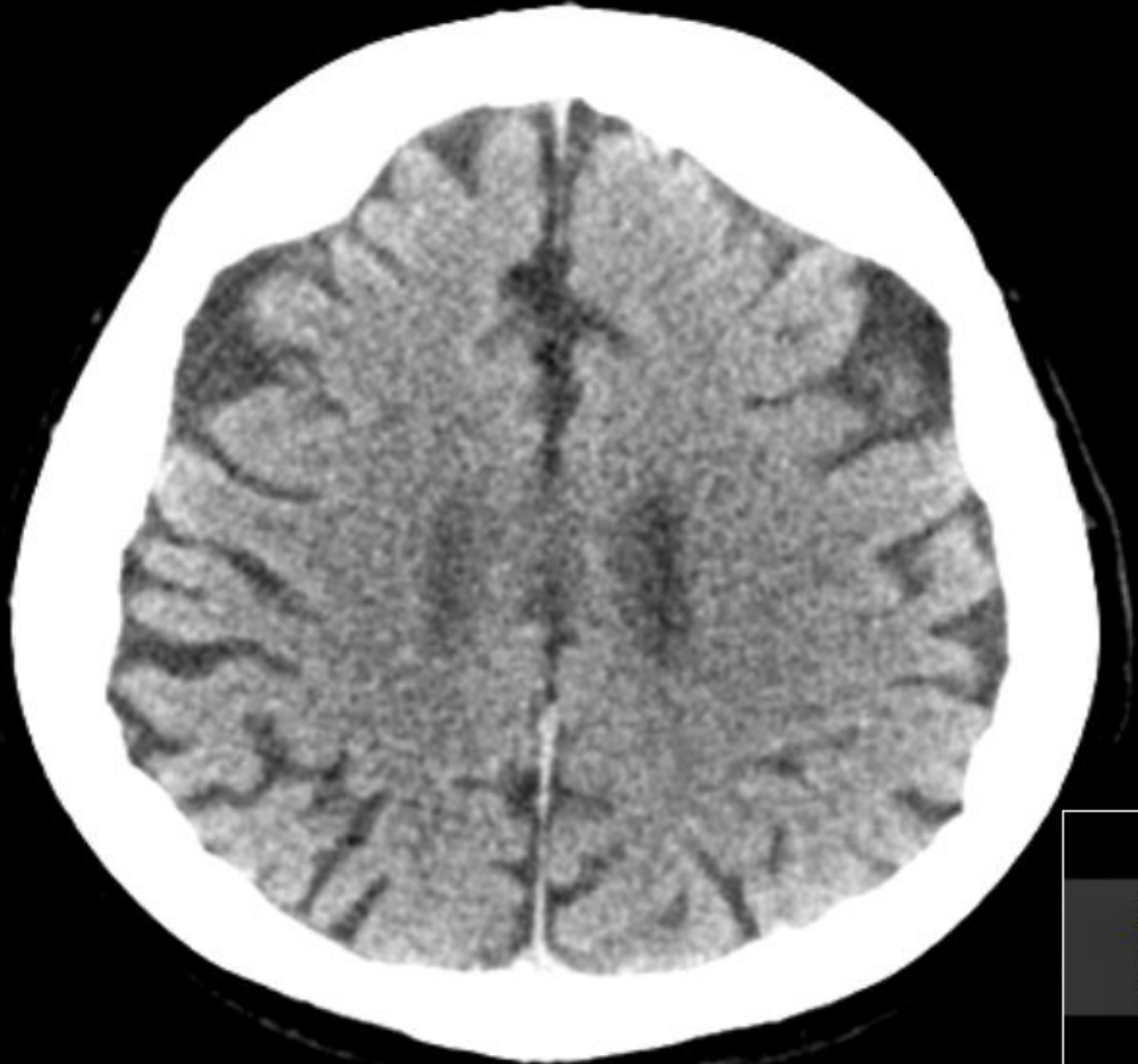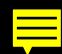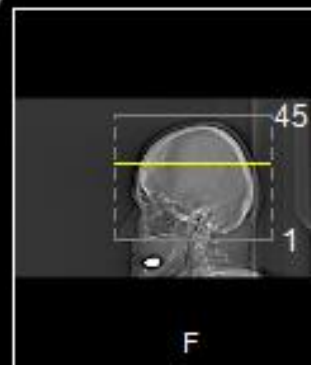

6

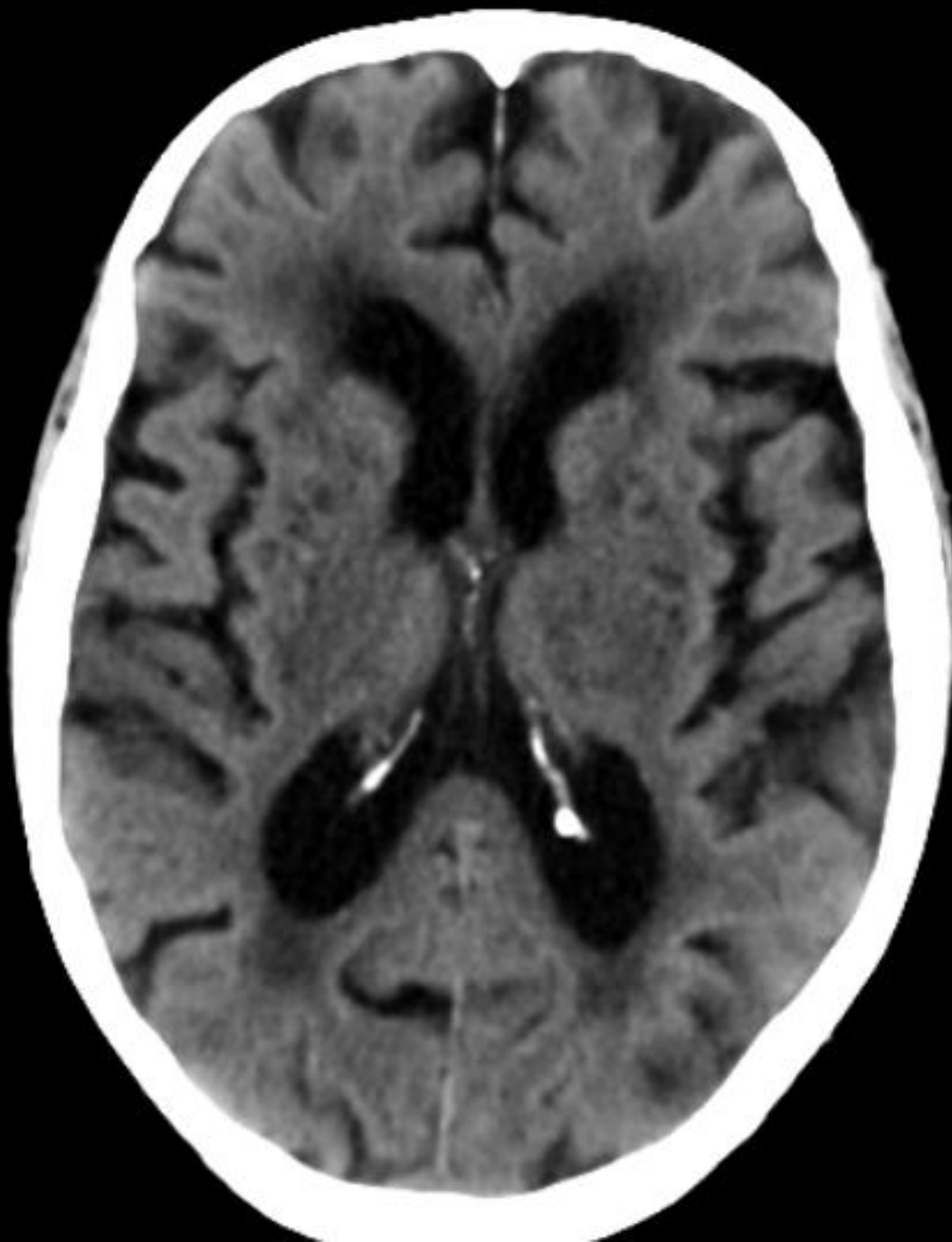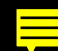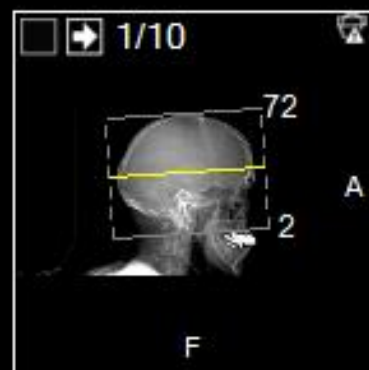

7

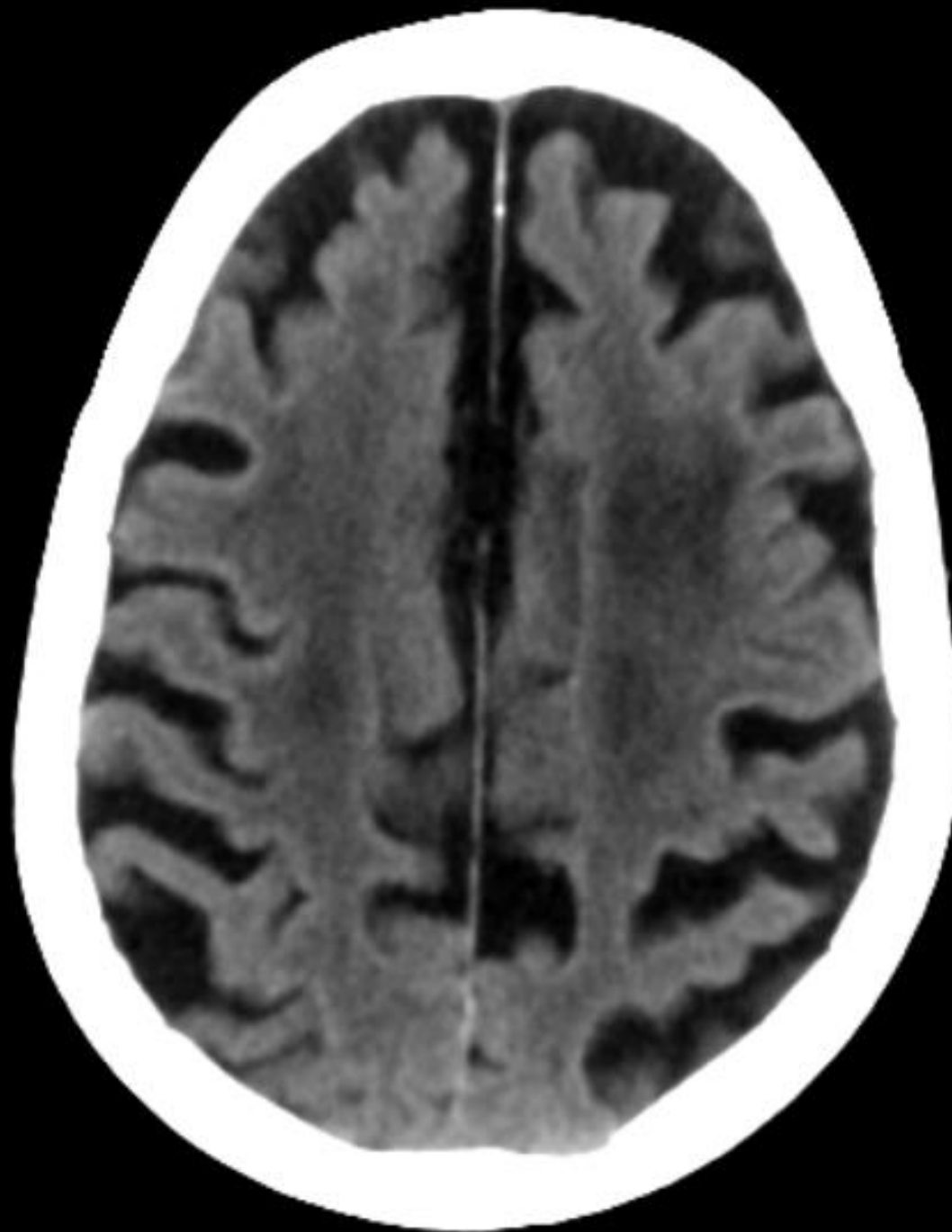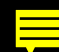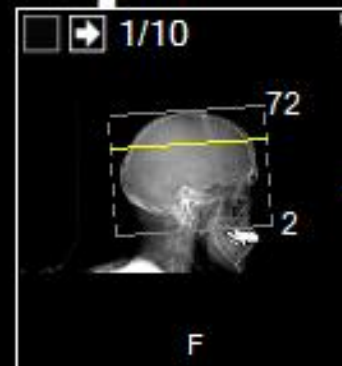

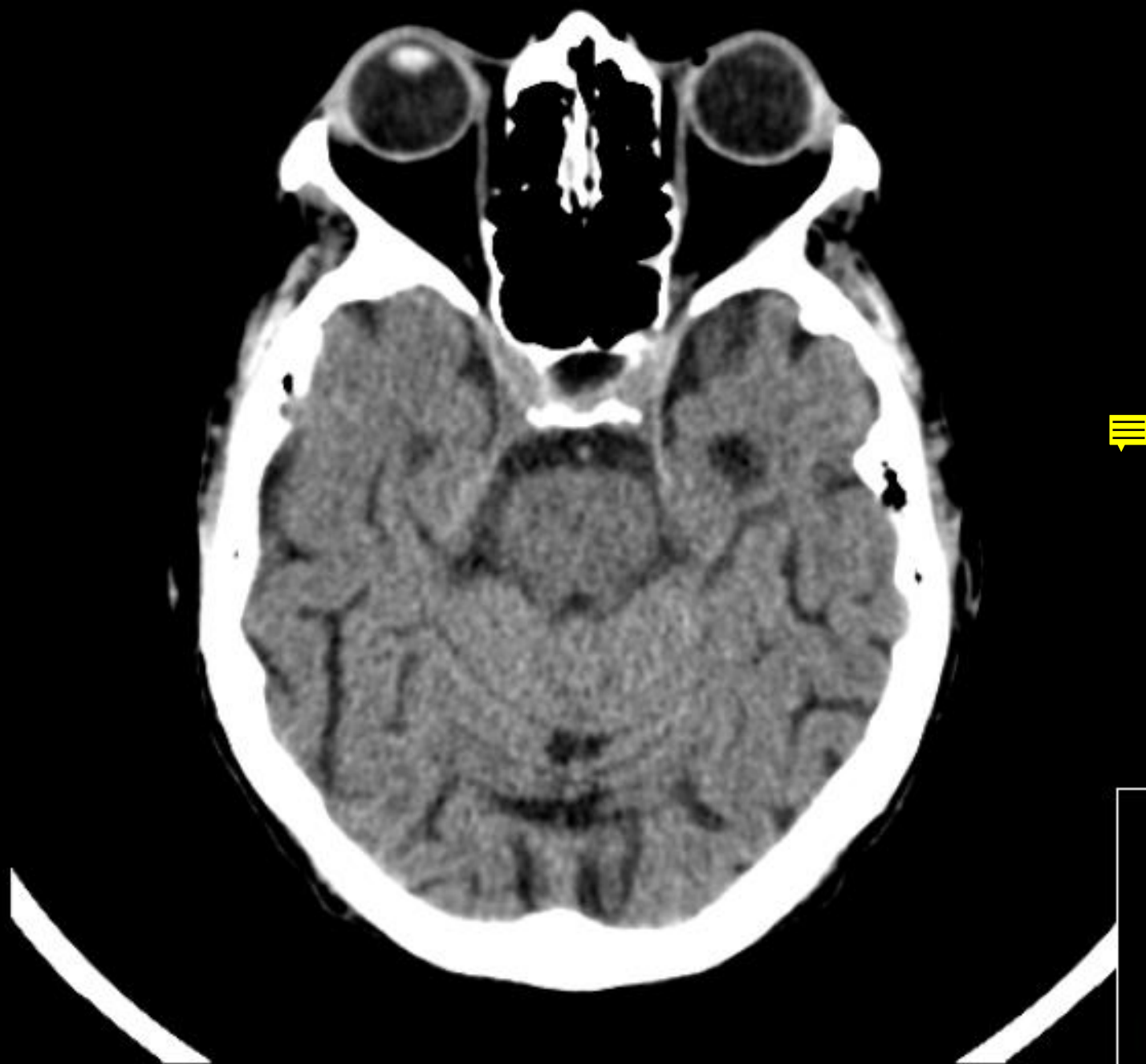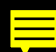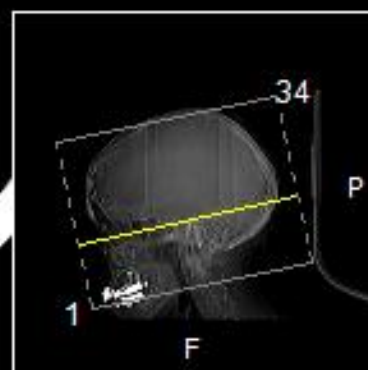

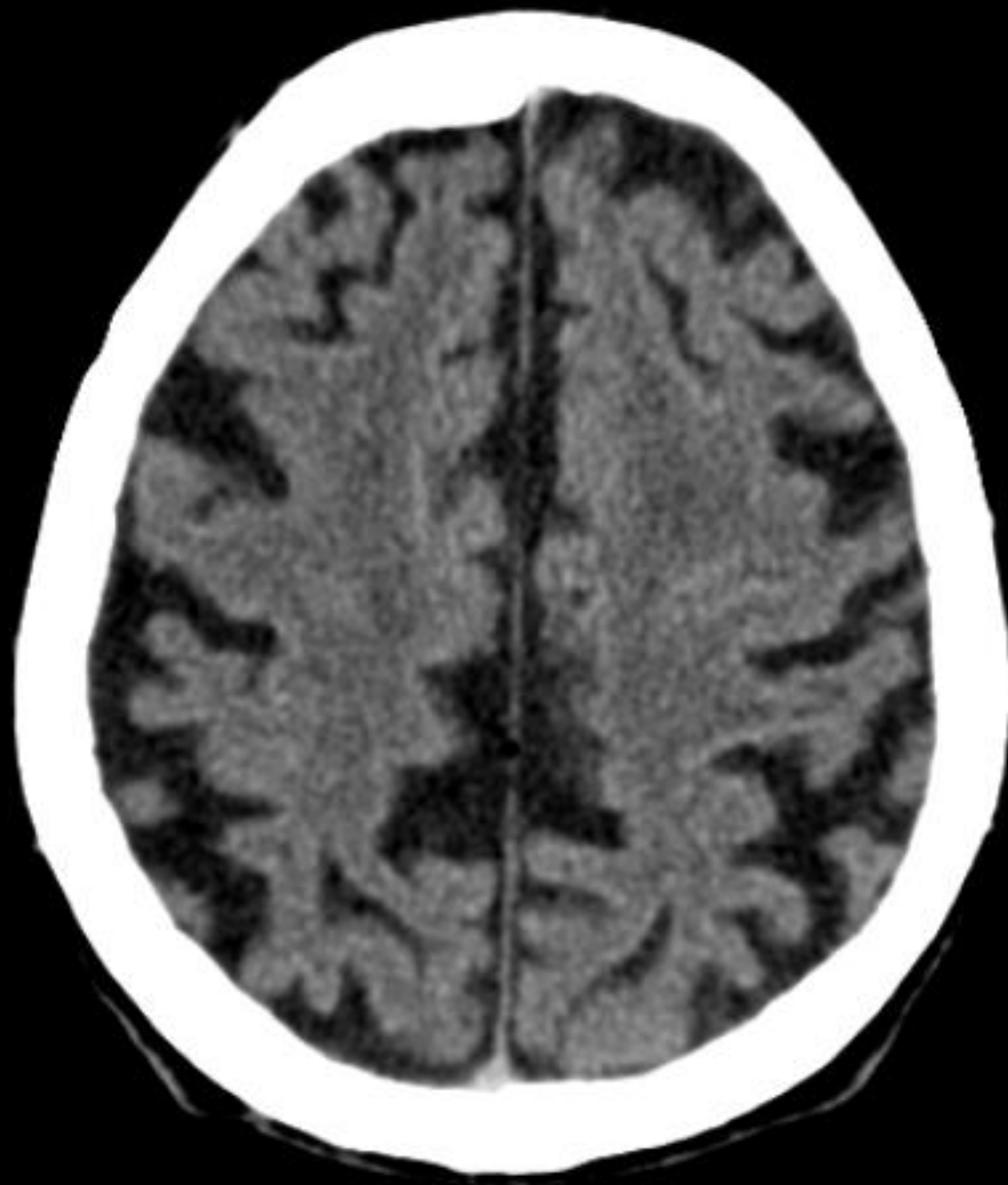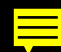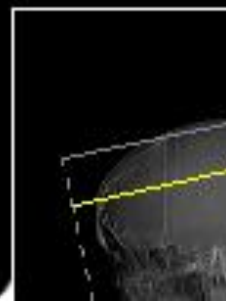

10

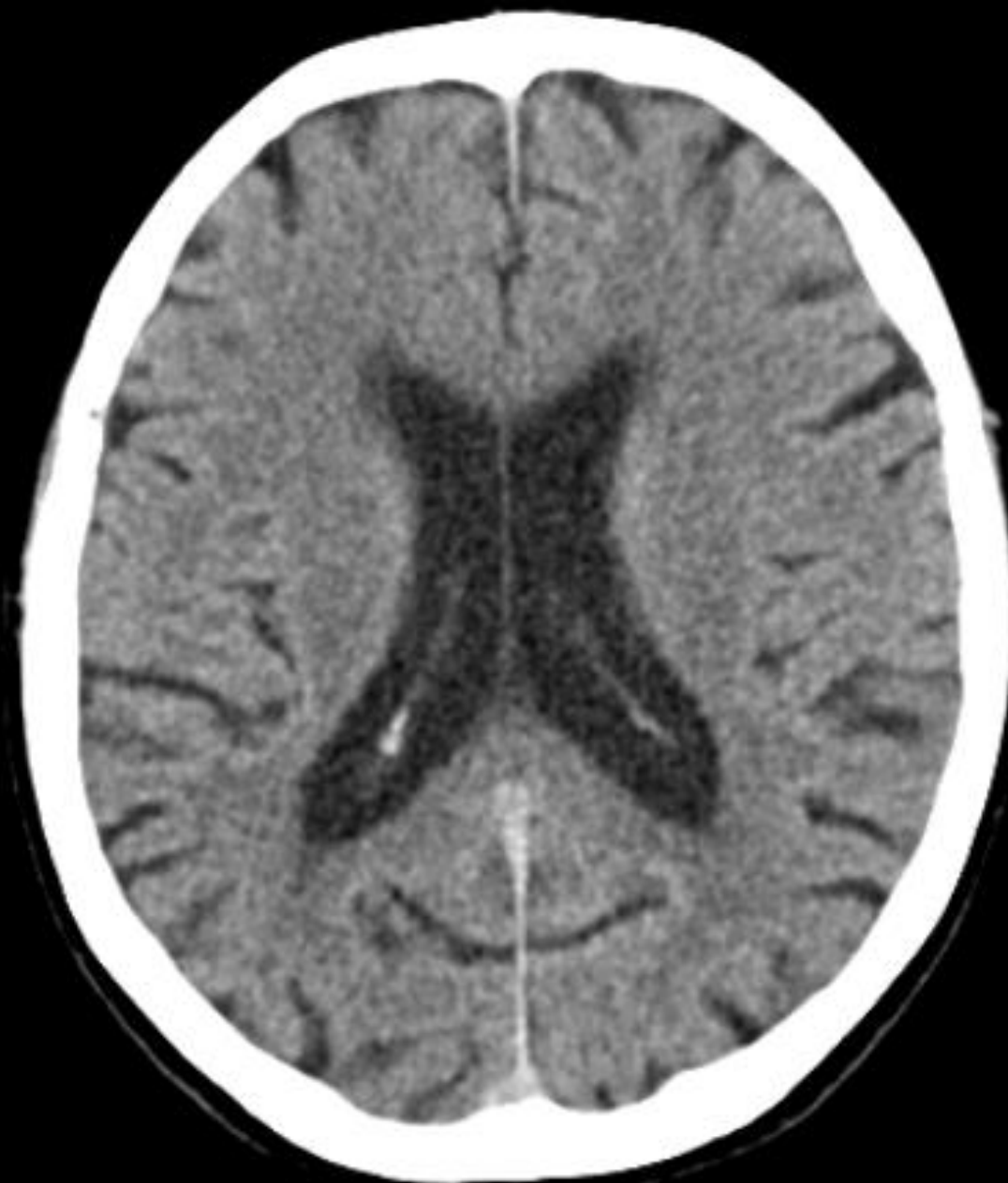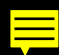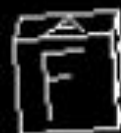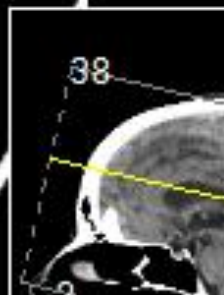

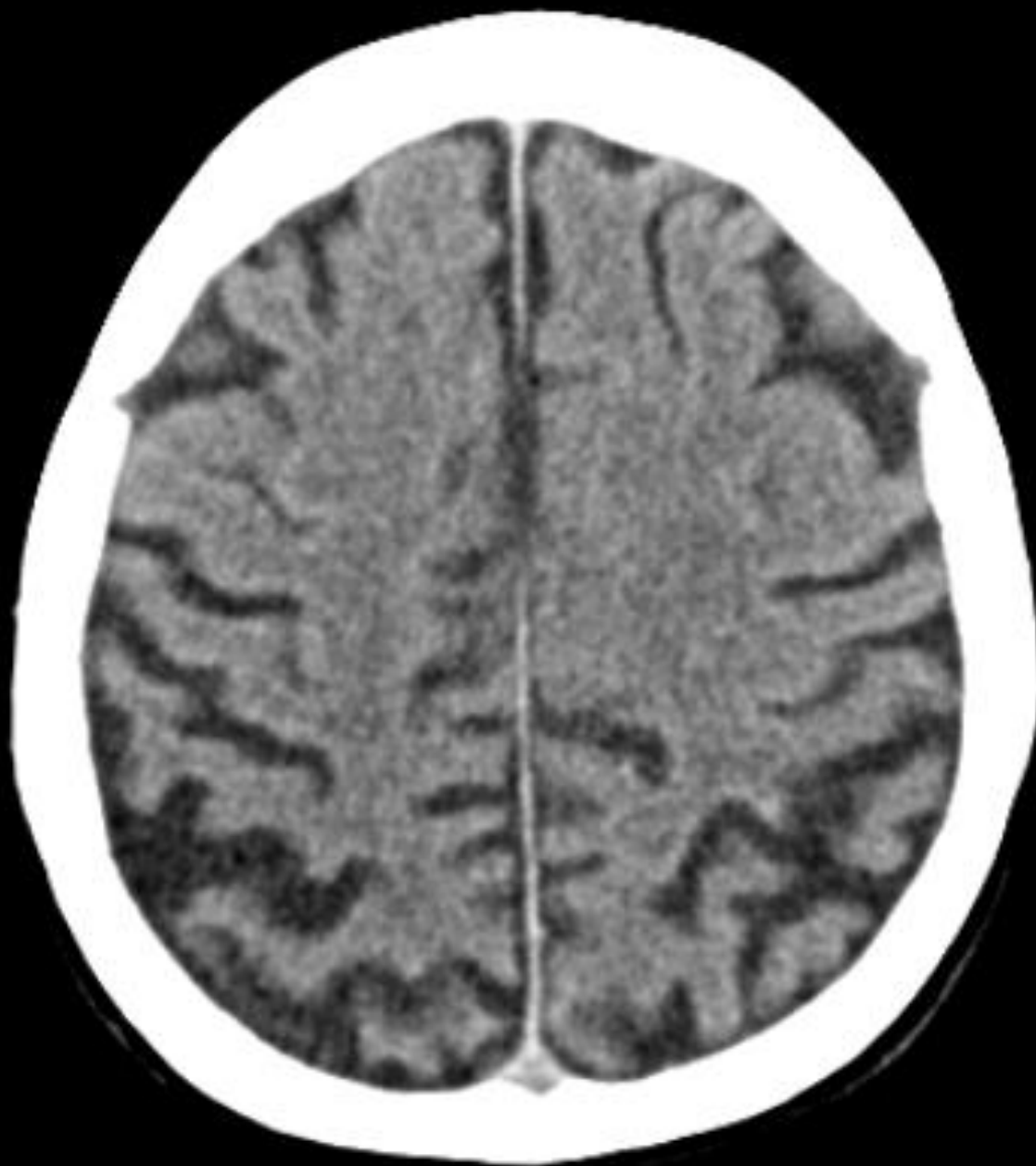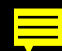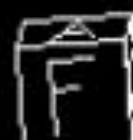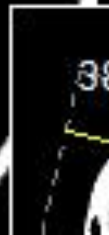

12

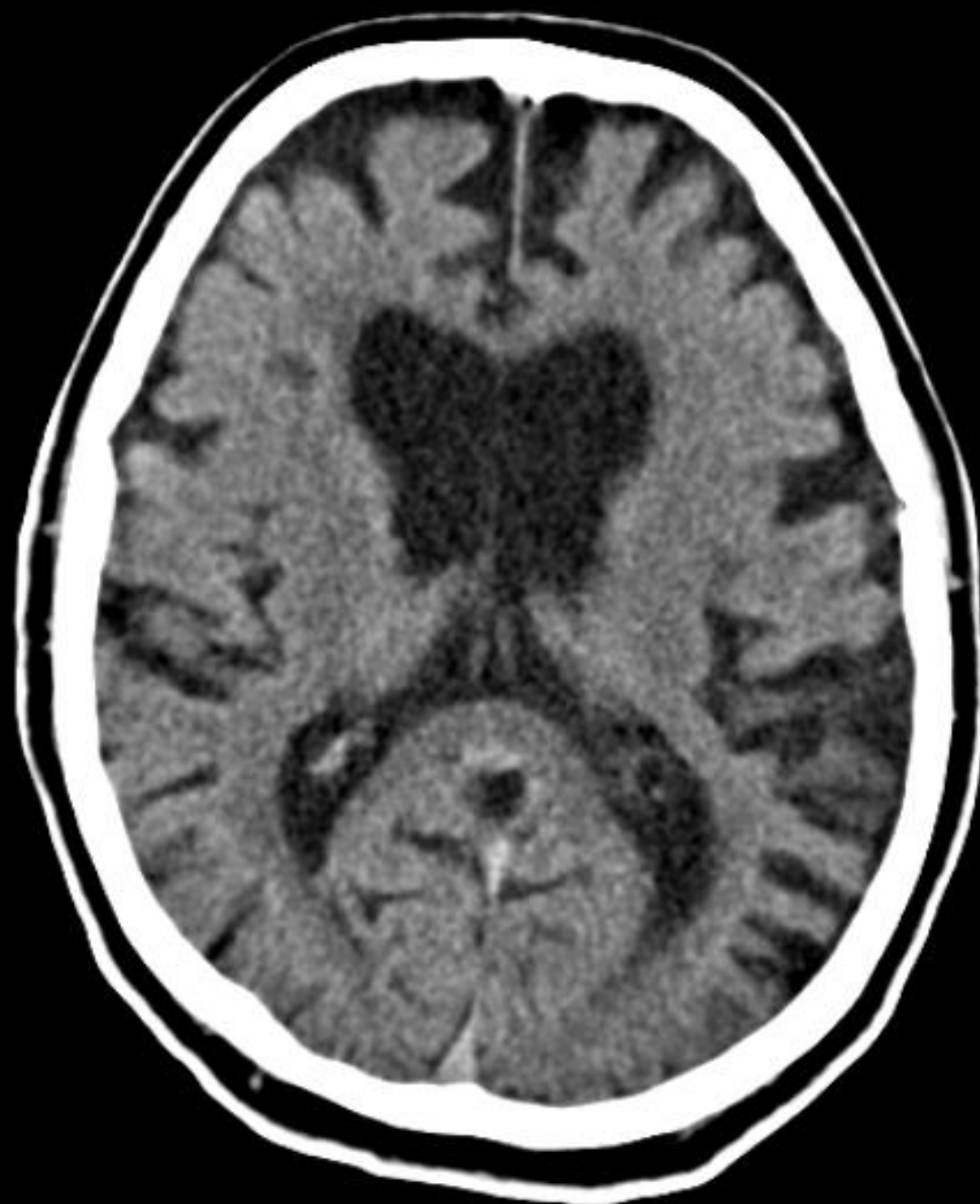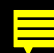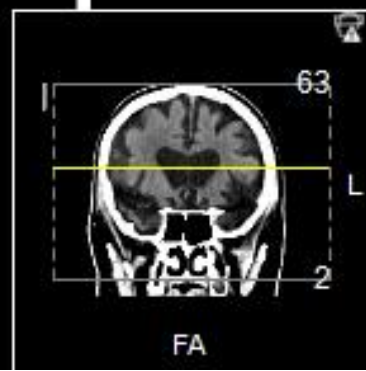

13

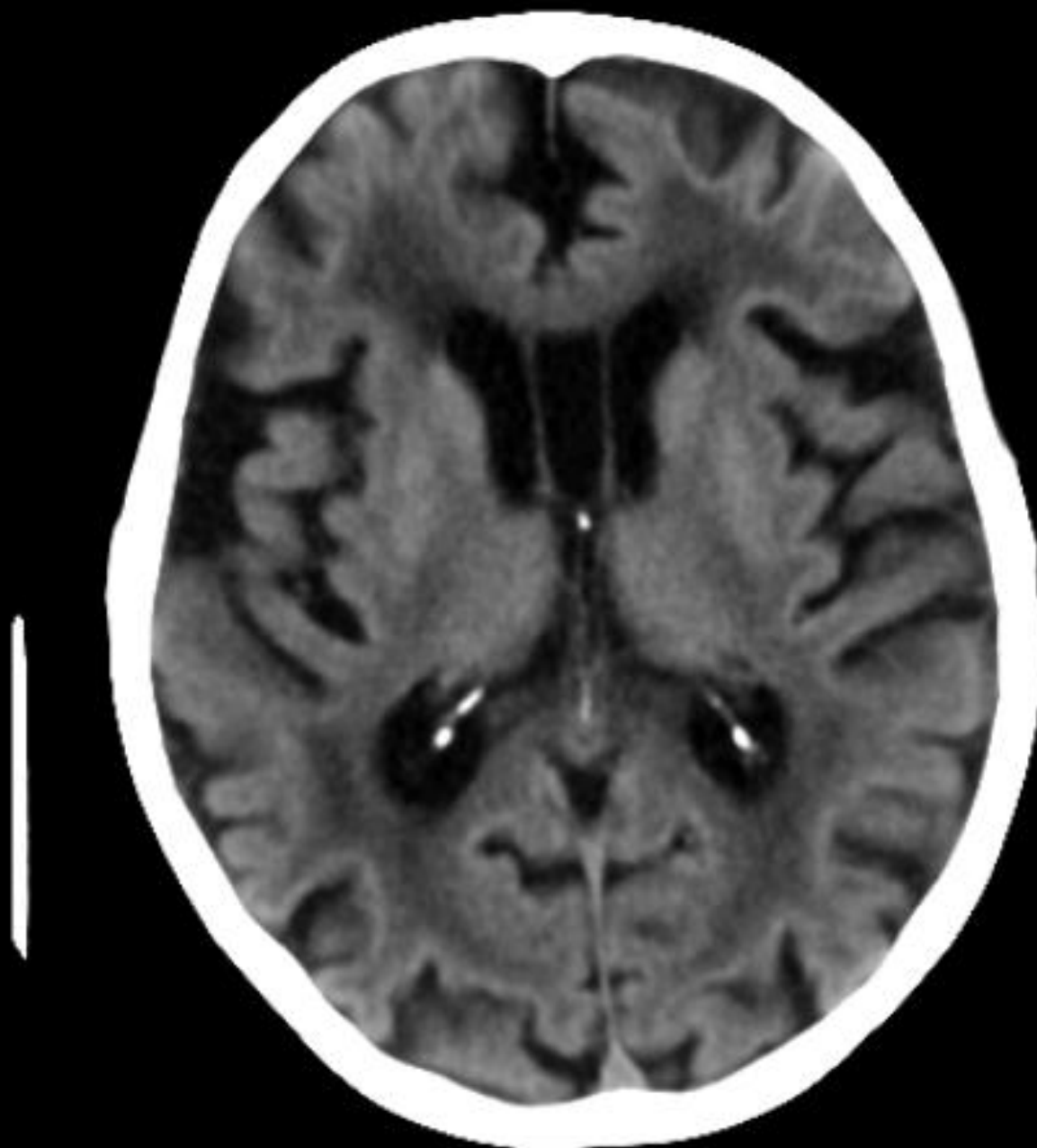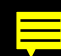

1/8

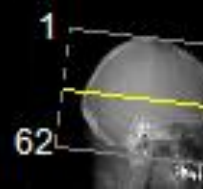

14

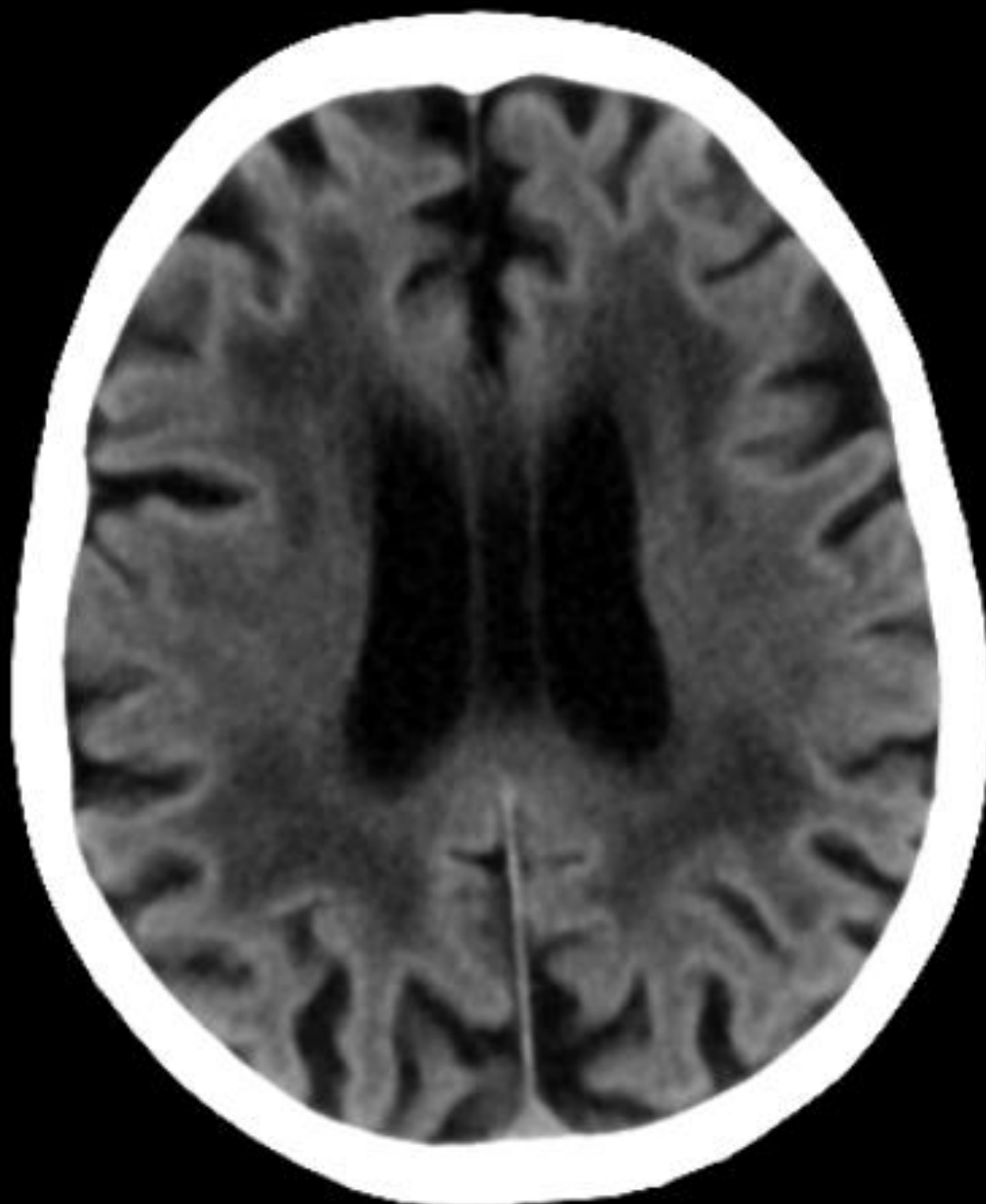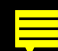

1/8

1

62

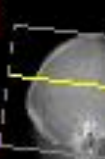

15

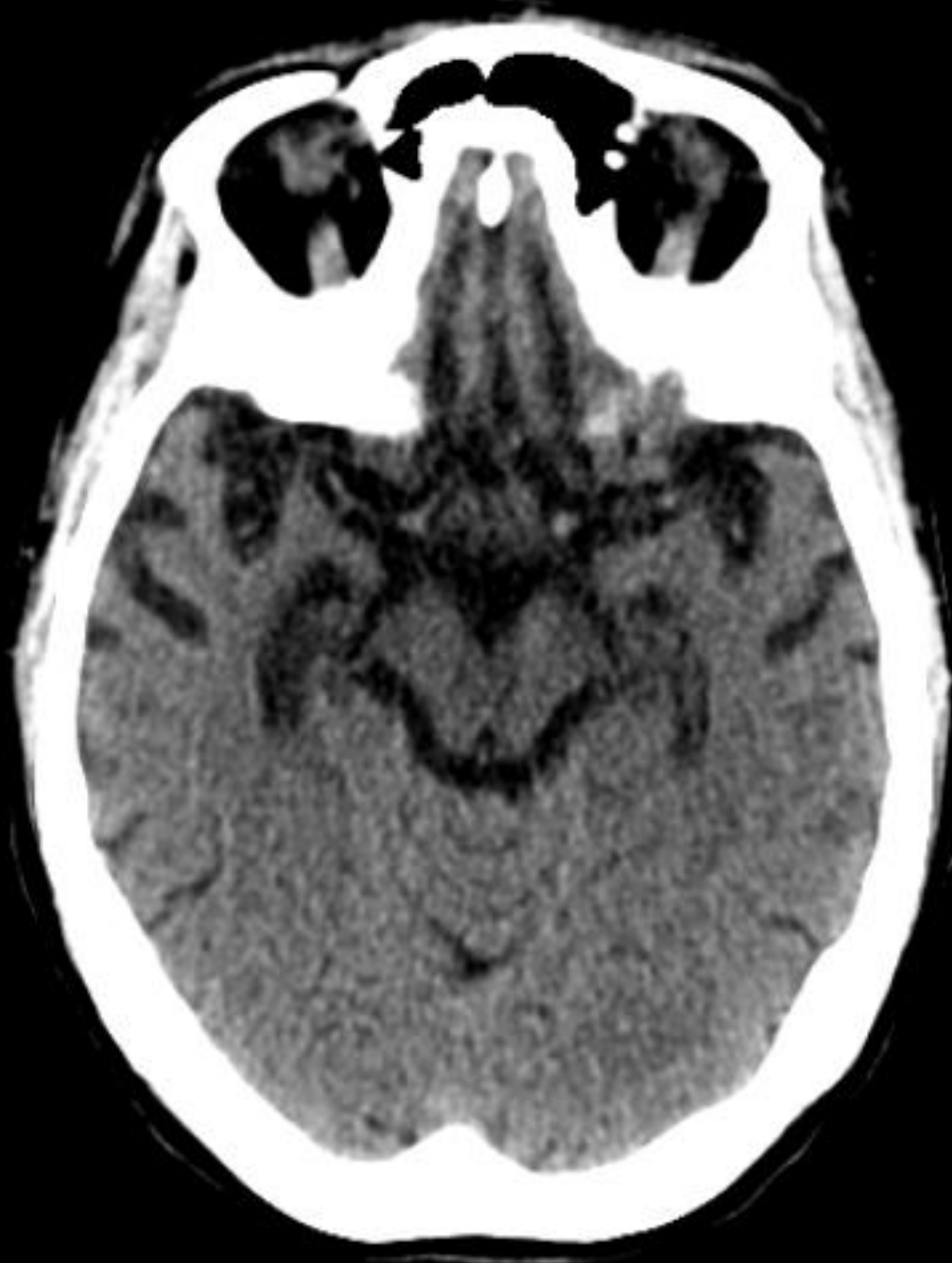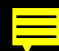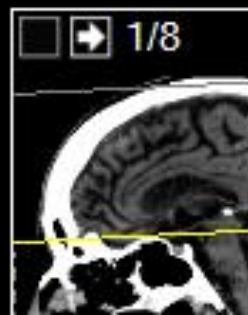

1/8

16

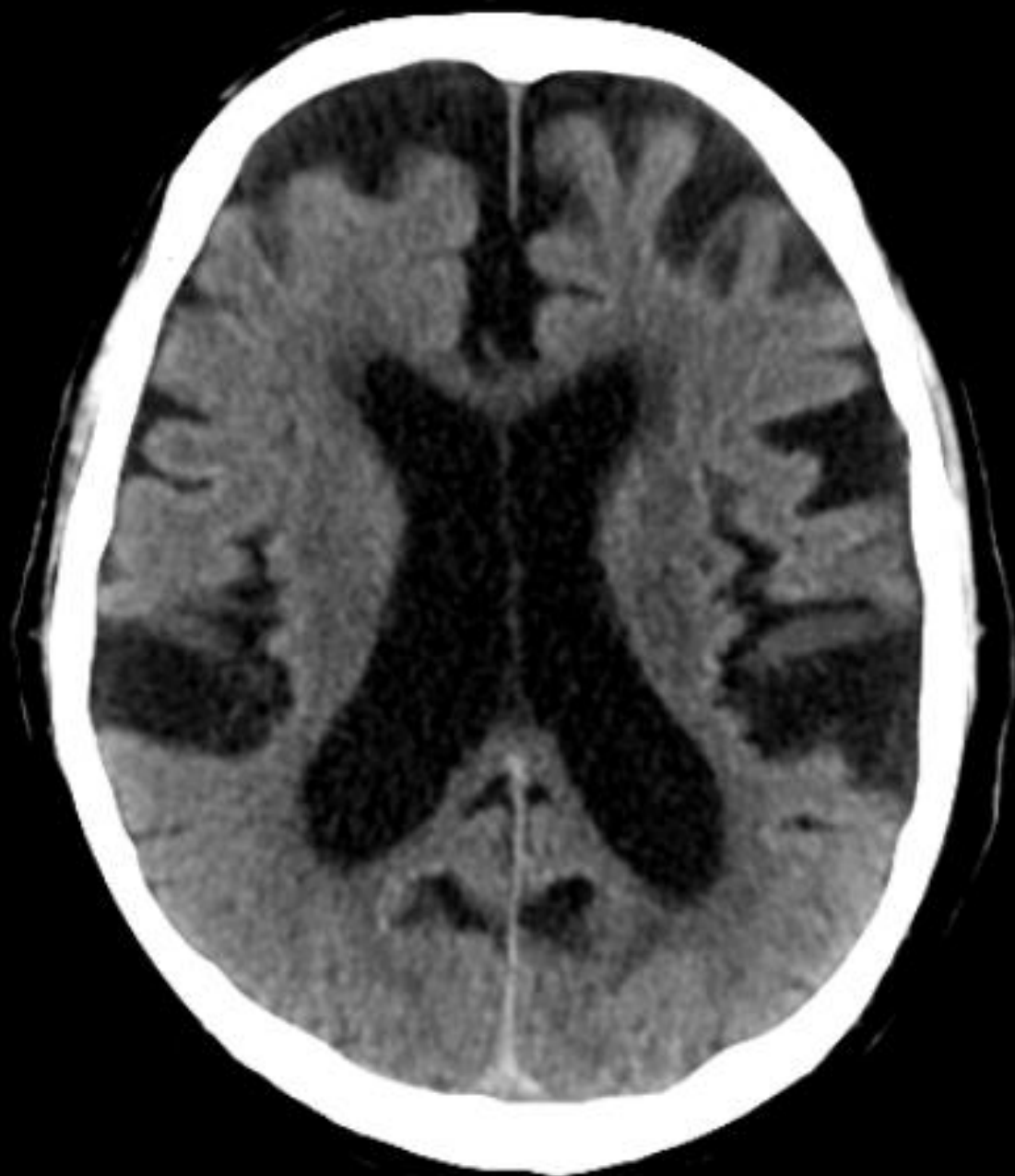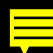

1/6

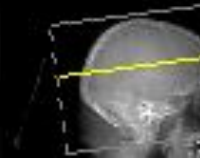

17

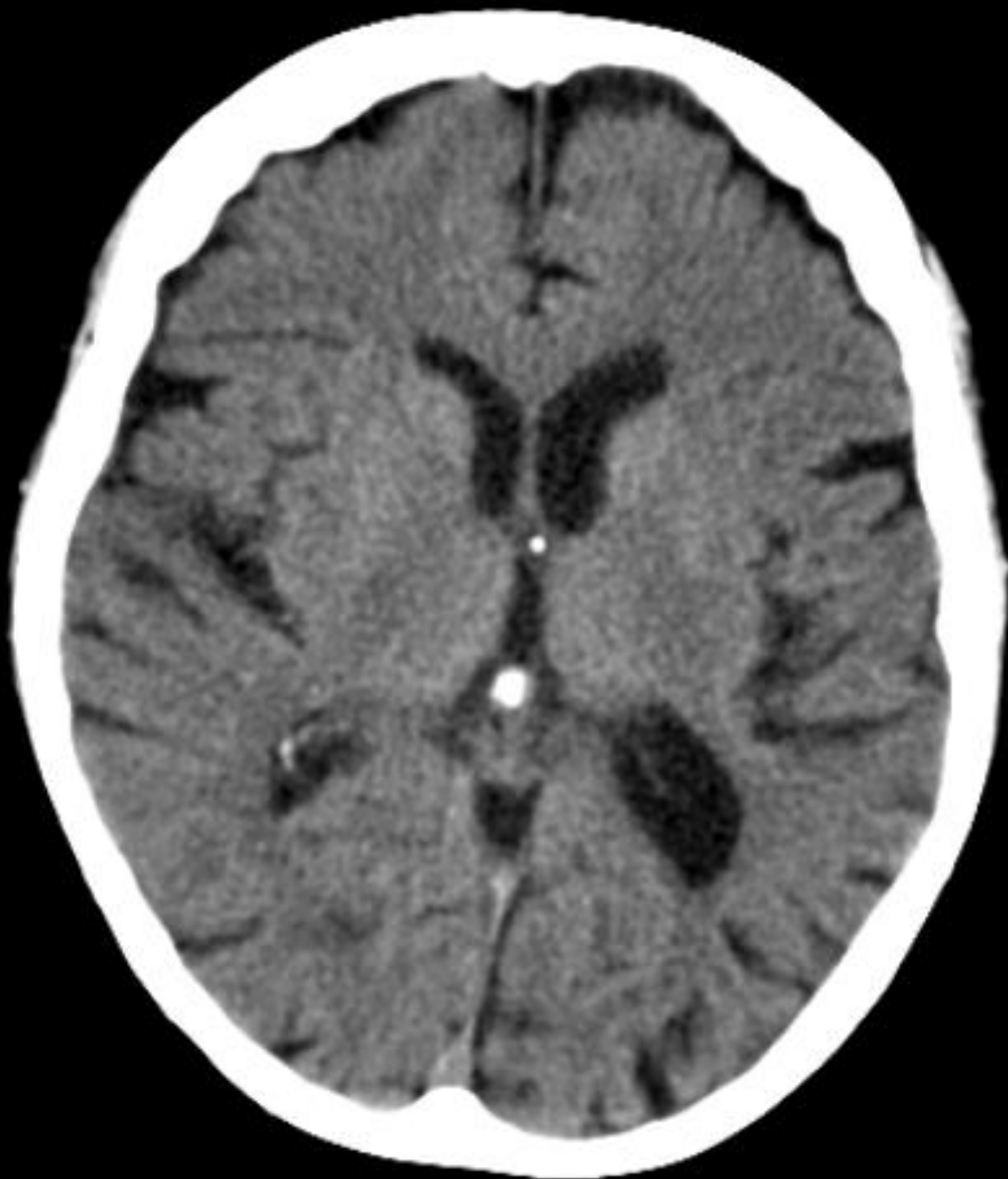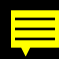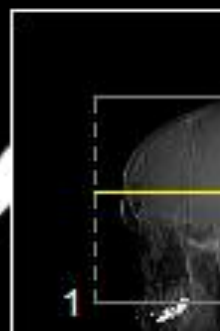

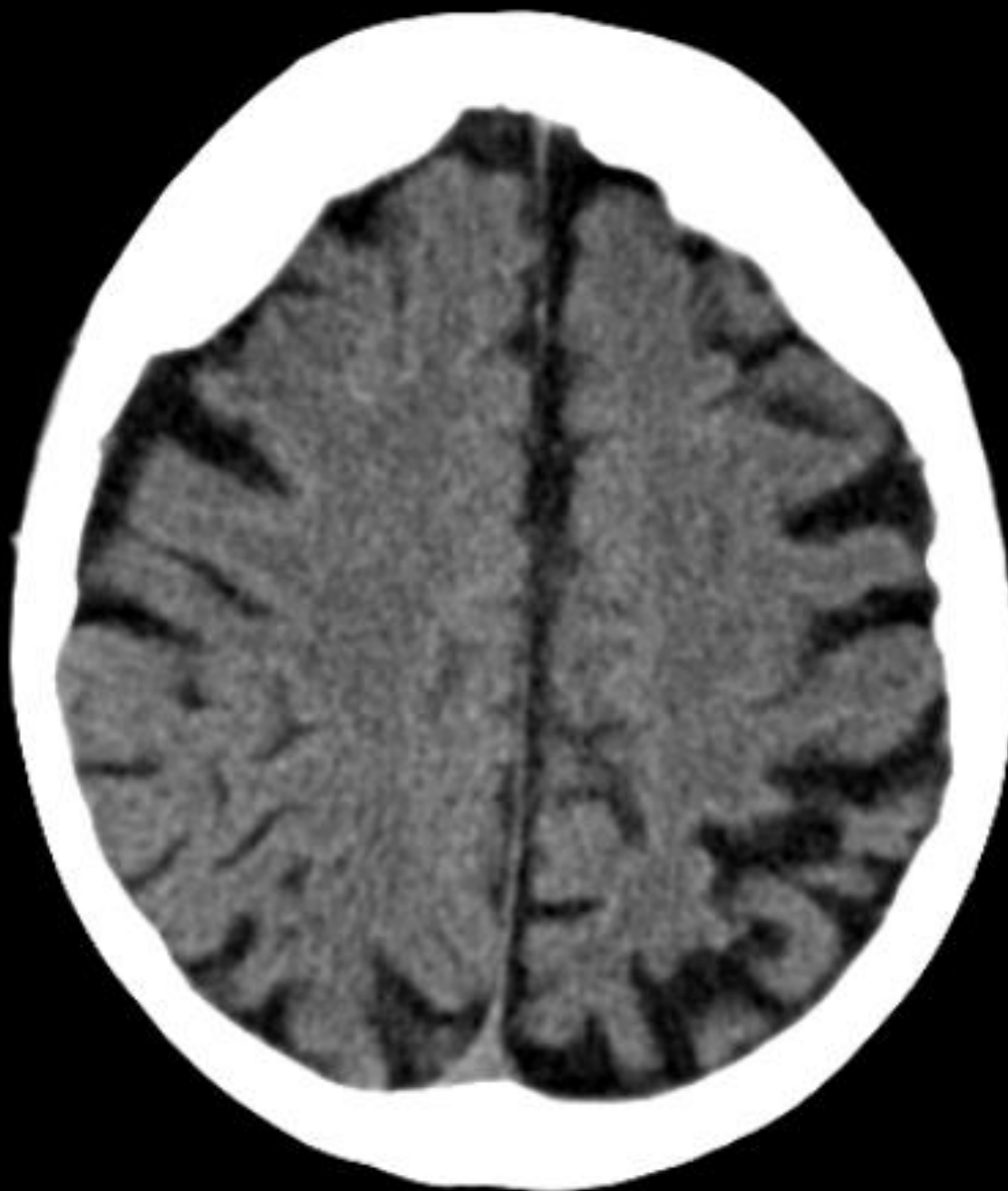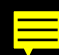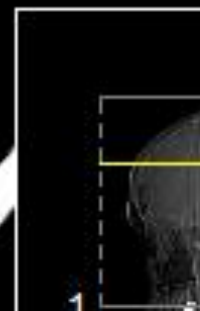

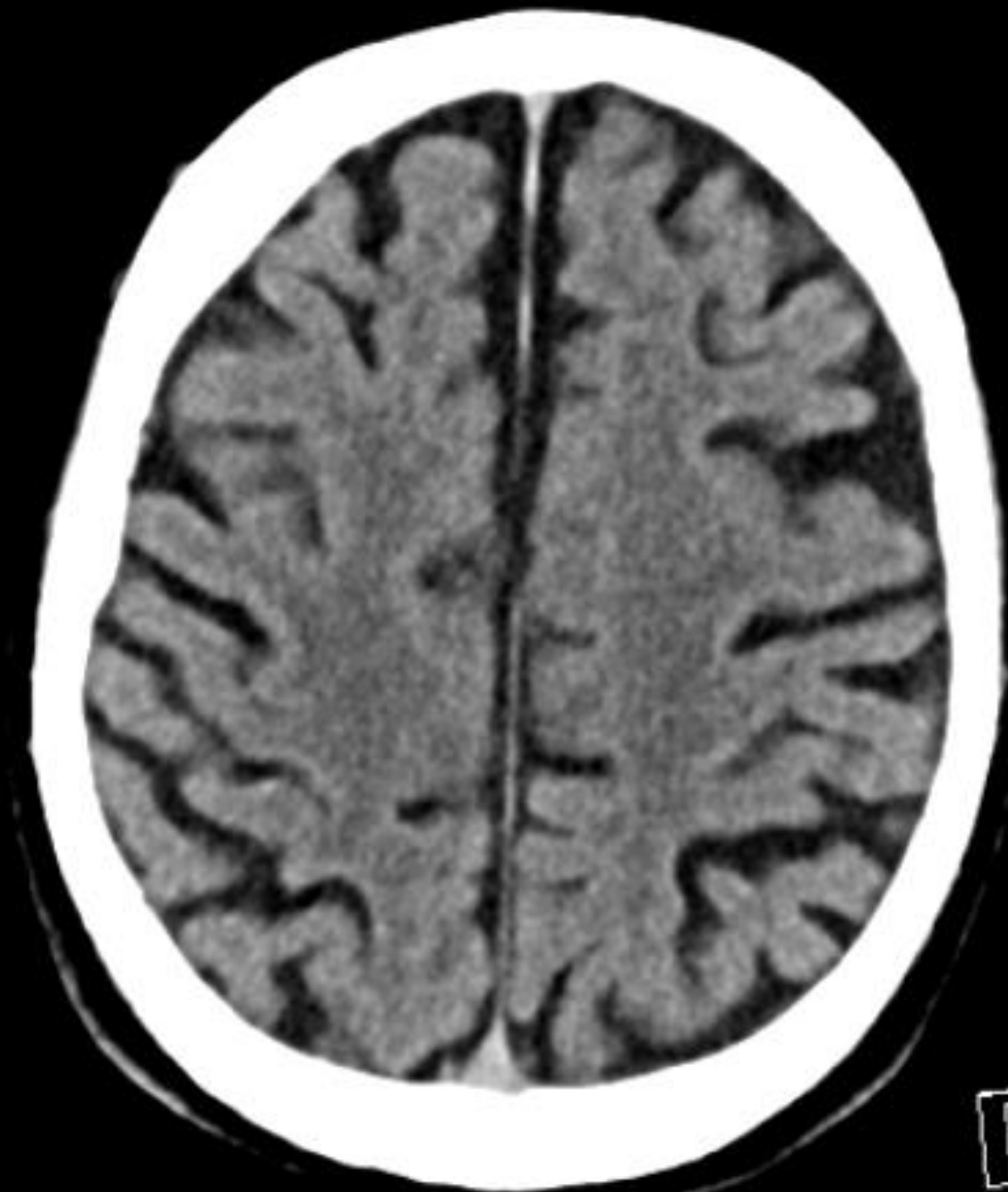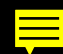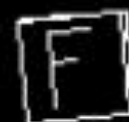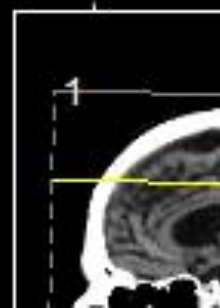

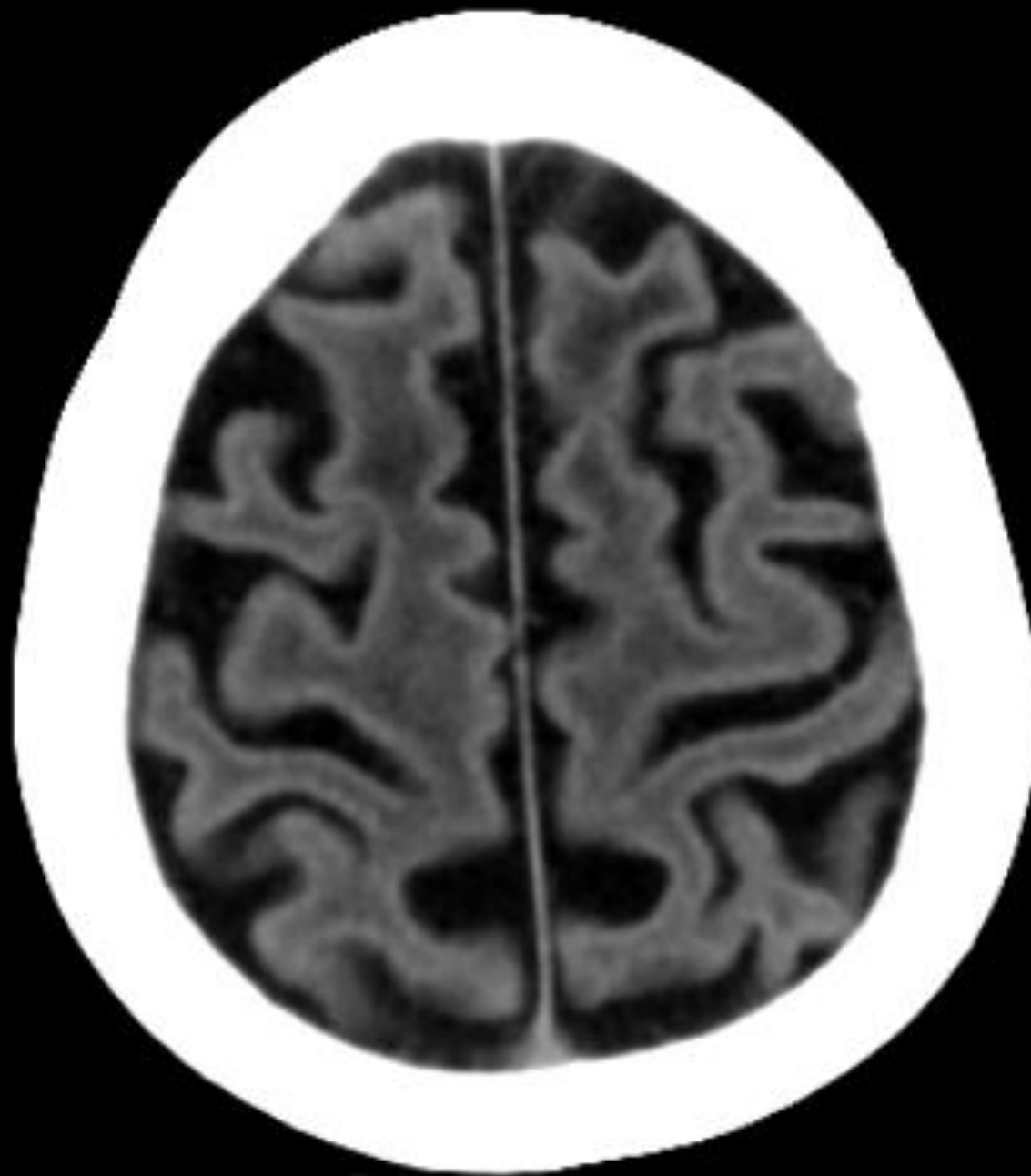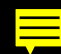

Supplement: Supplementary file 6 — (PDF 787 kb) [file 13244_2016_521_MOESM6_ESM.pdf]

1

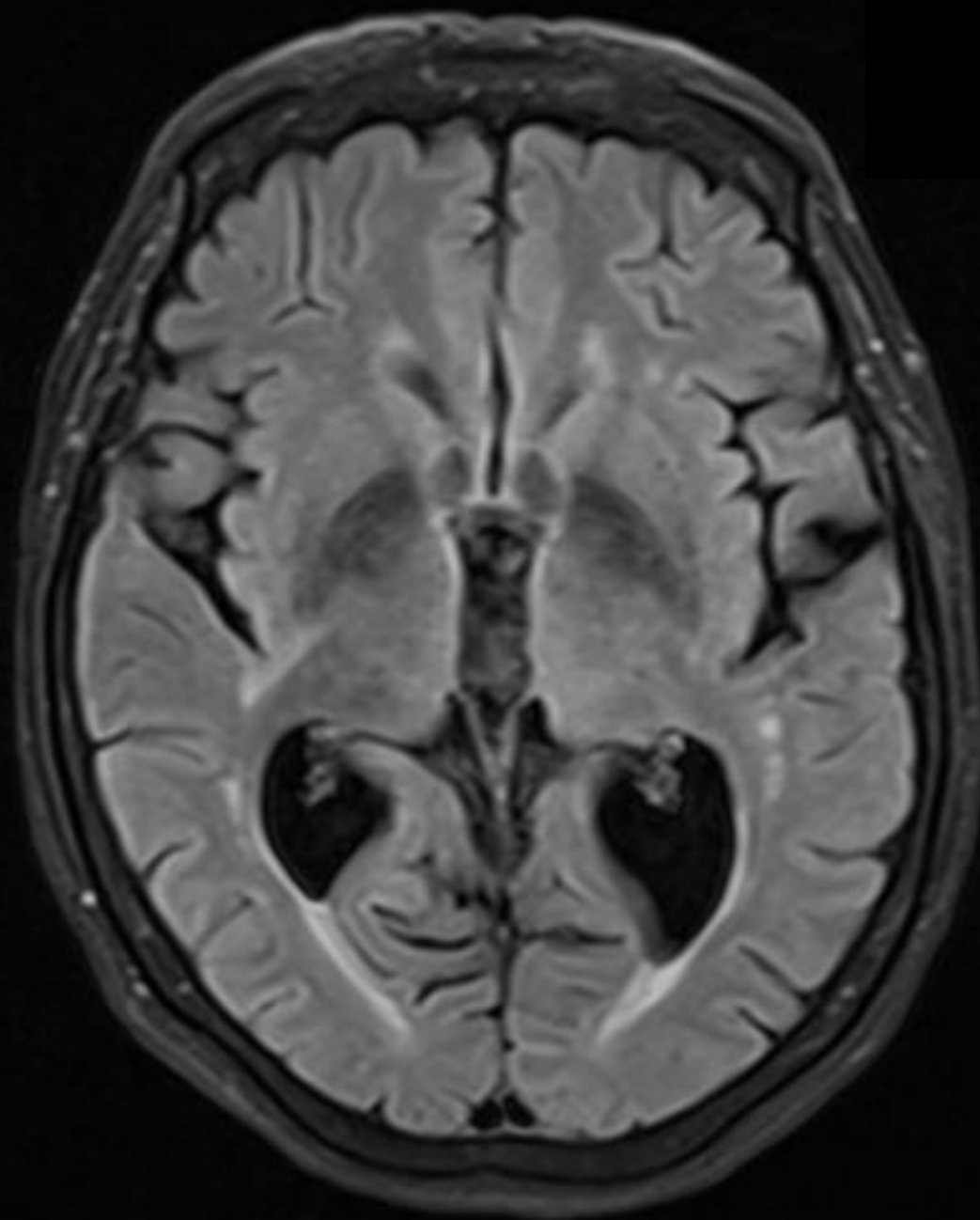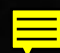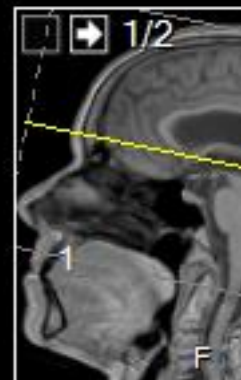

2

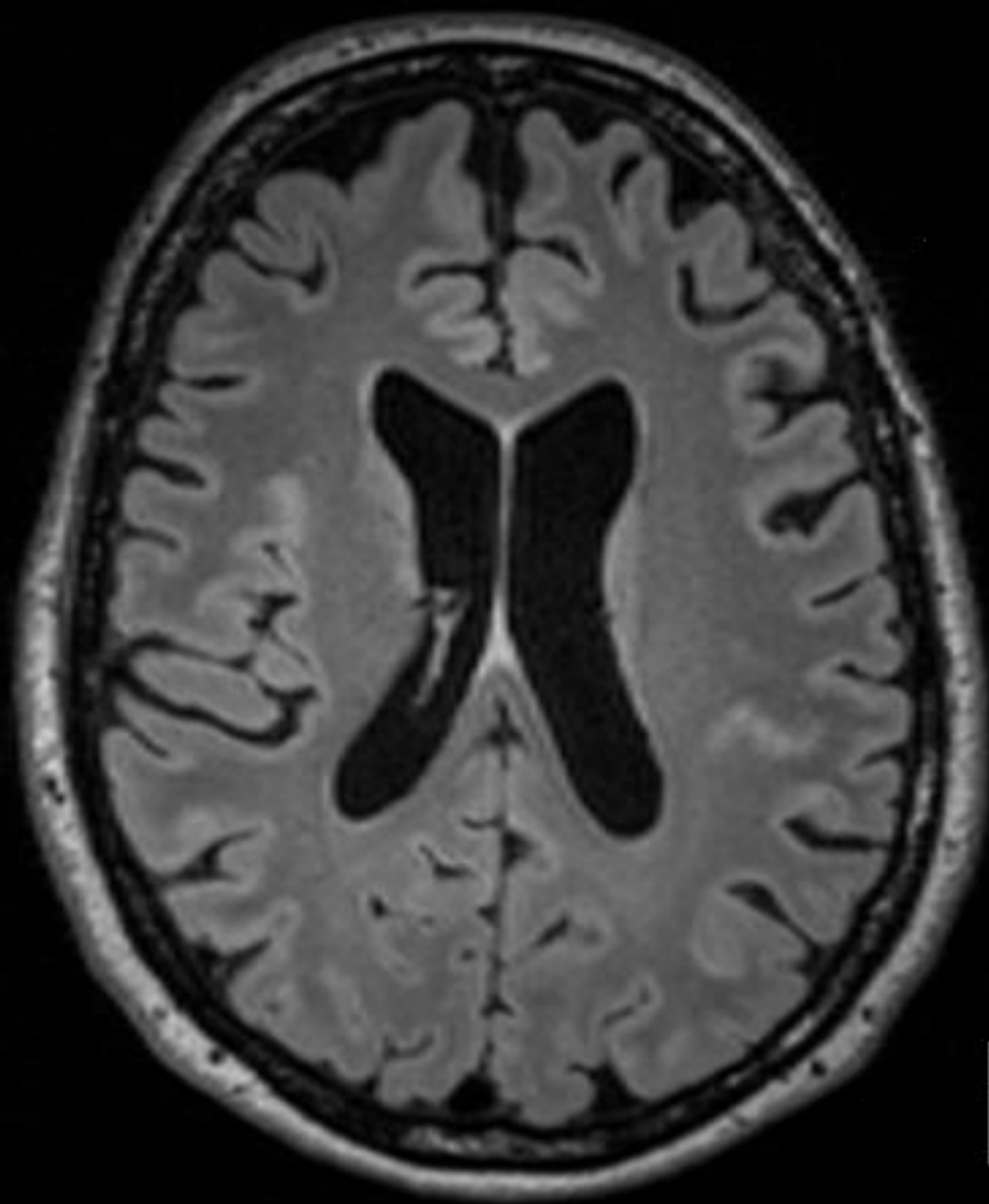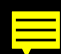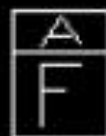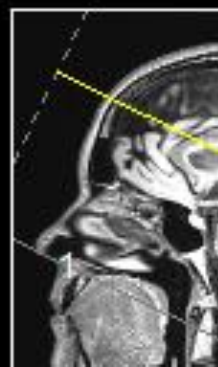

3

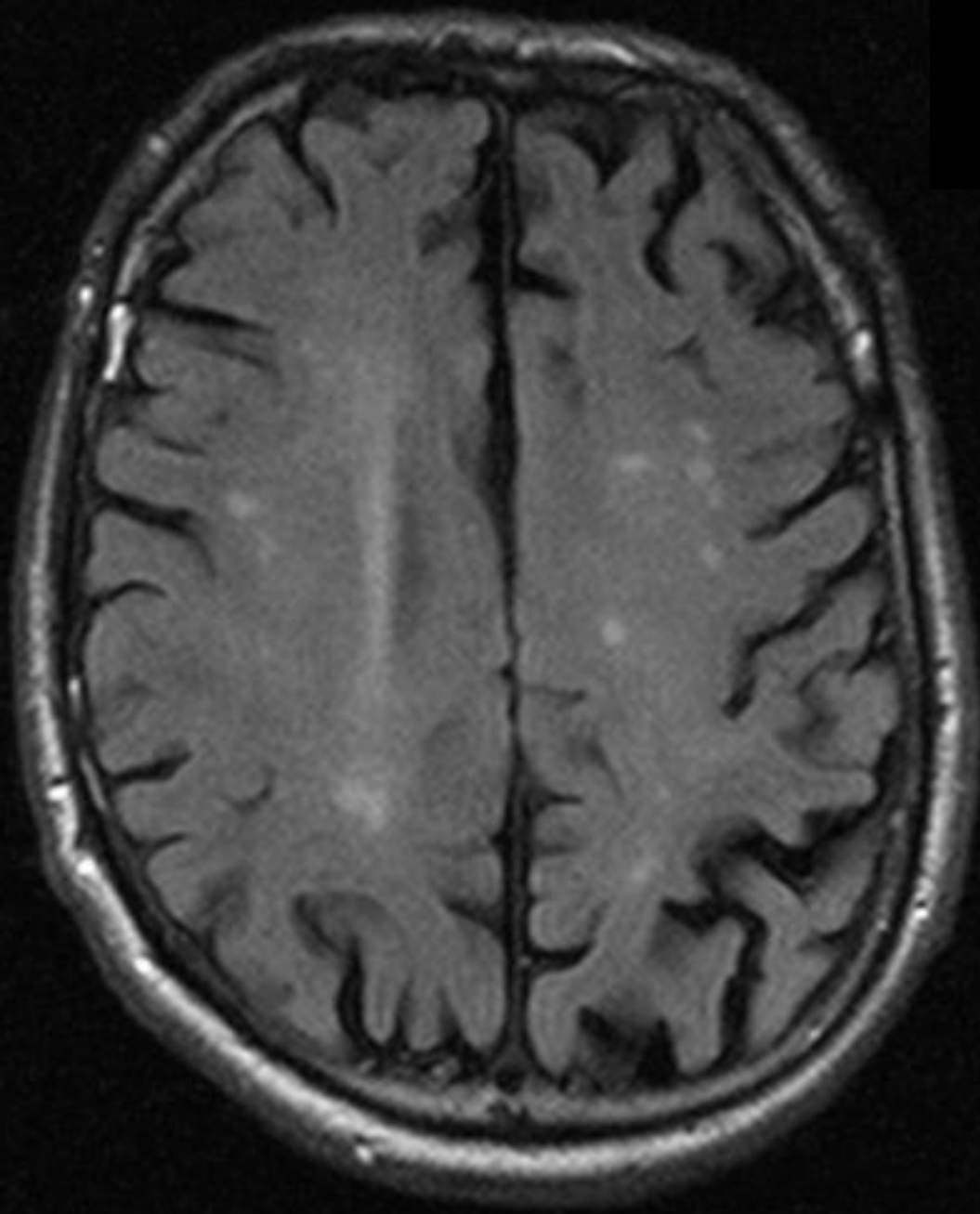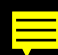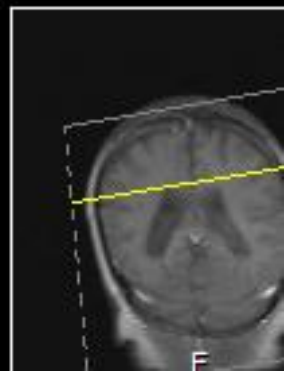

4

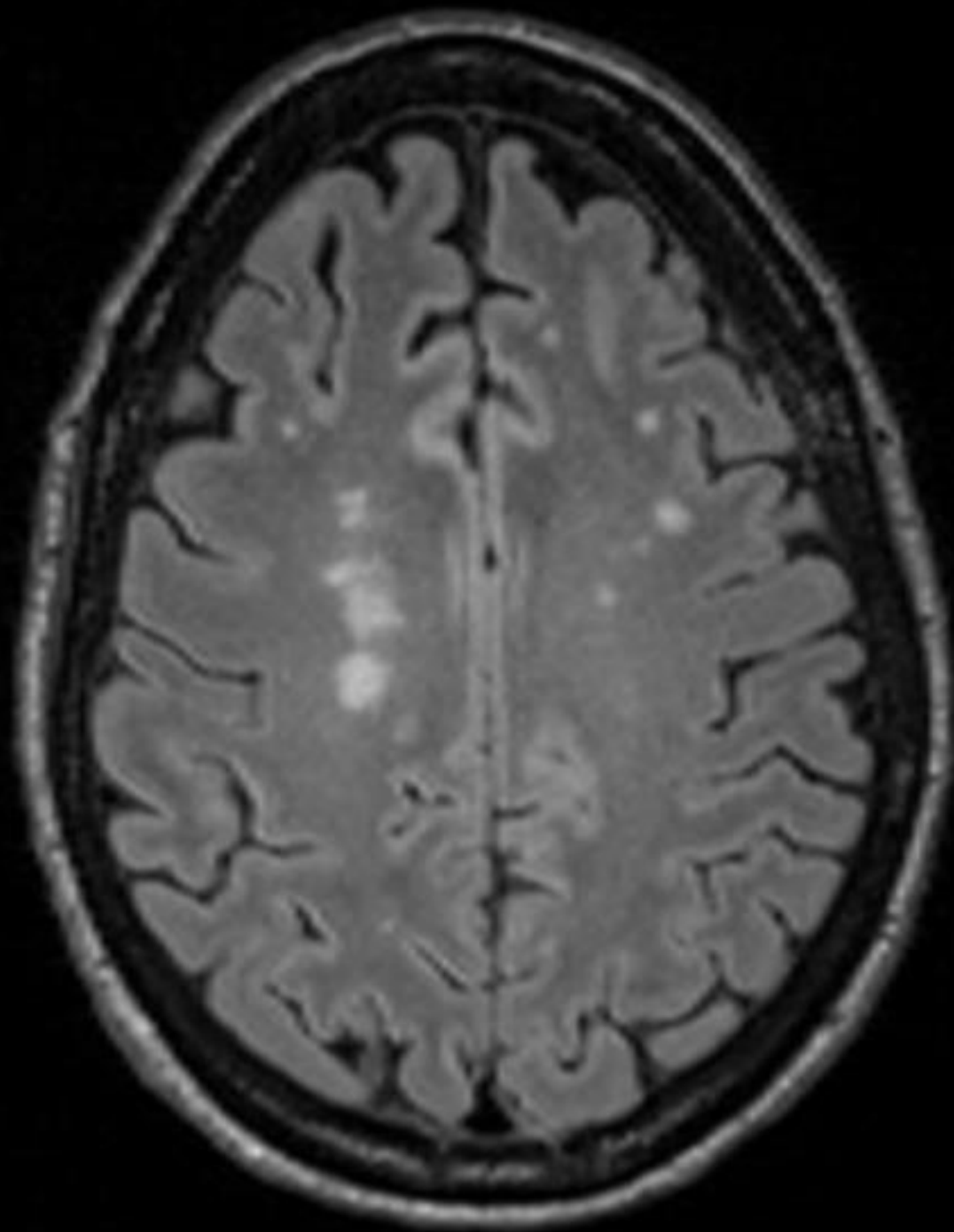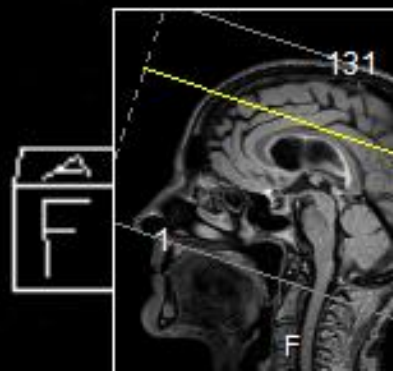

5

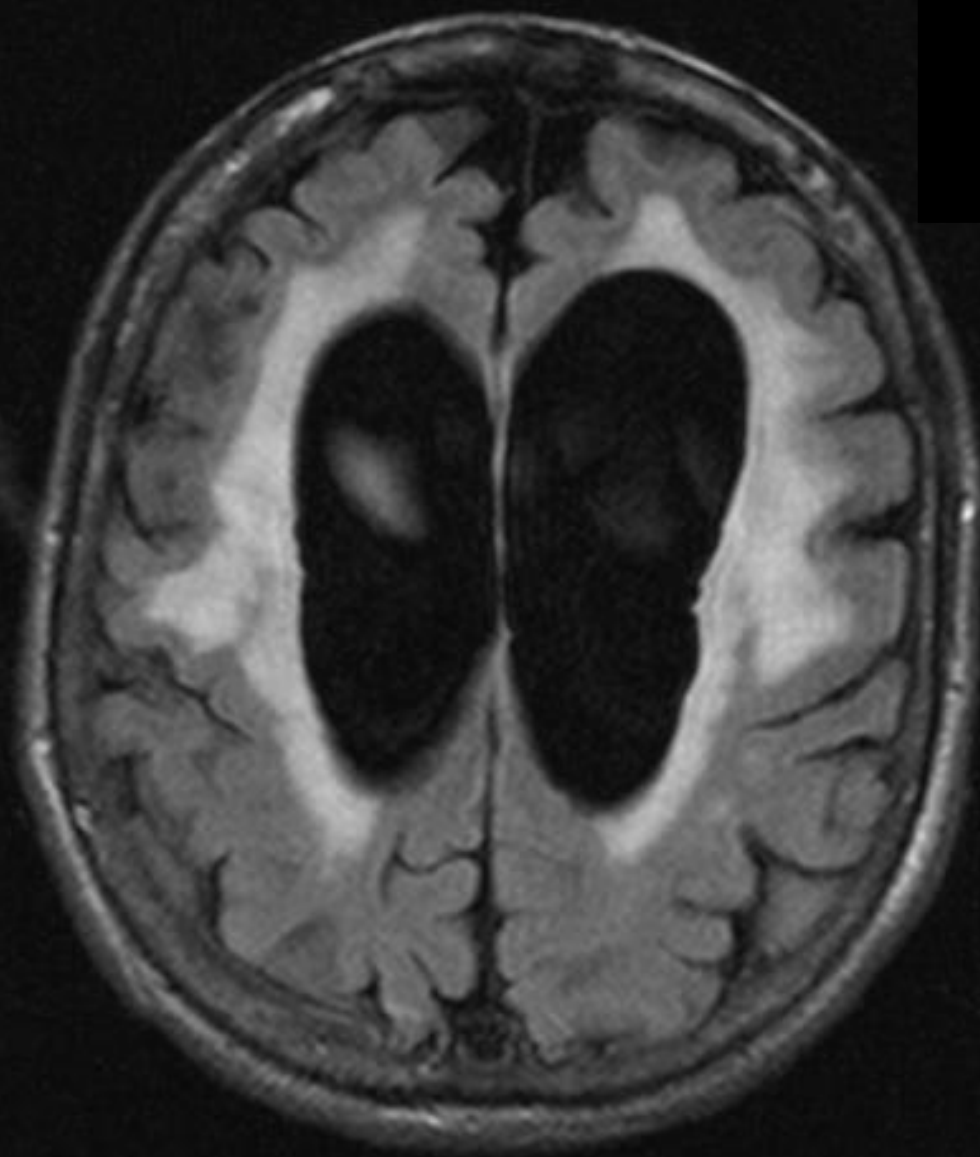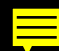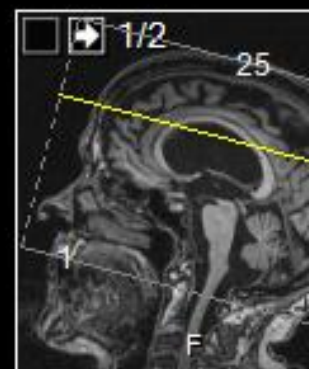

6

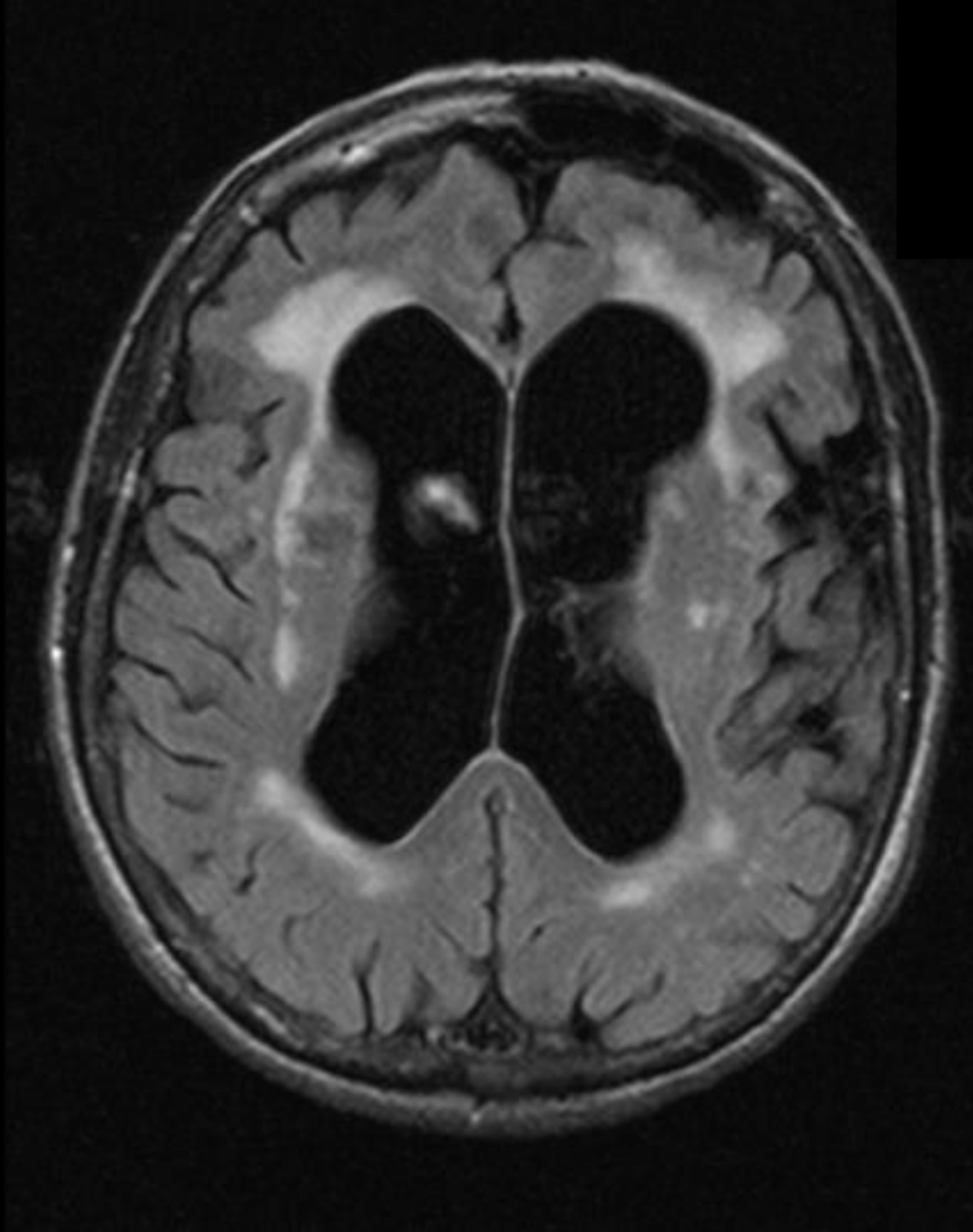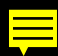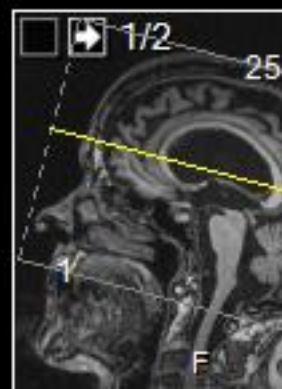

7

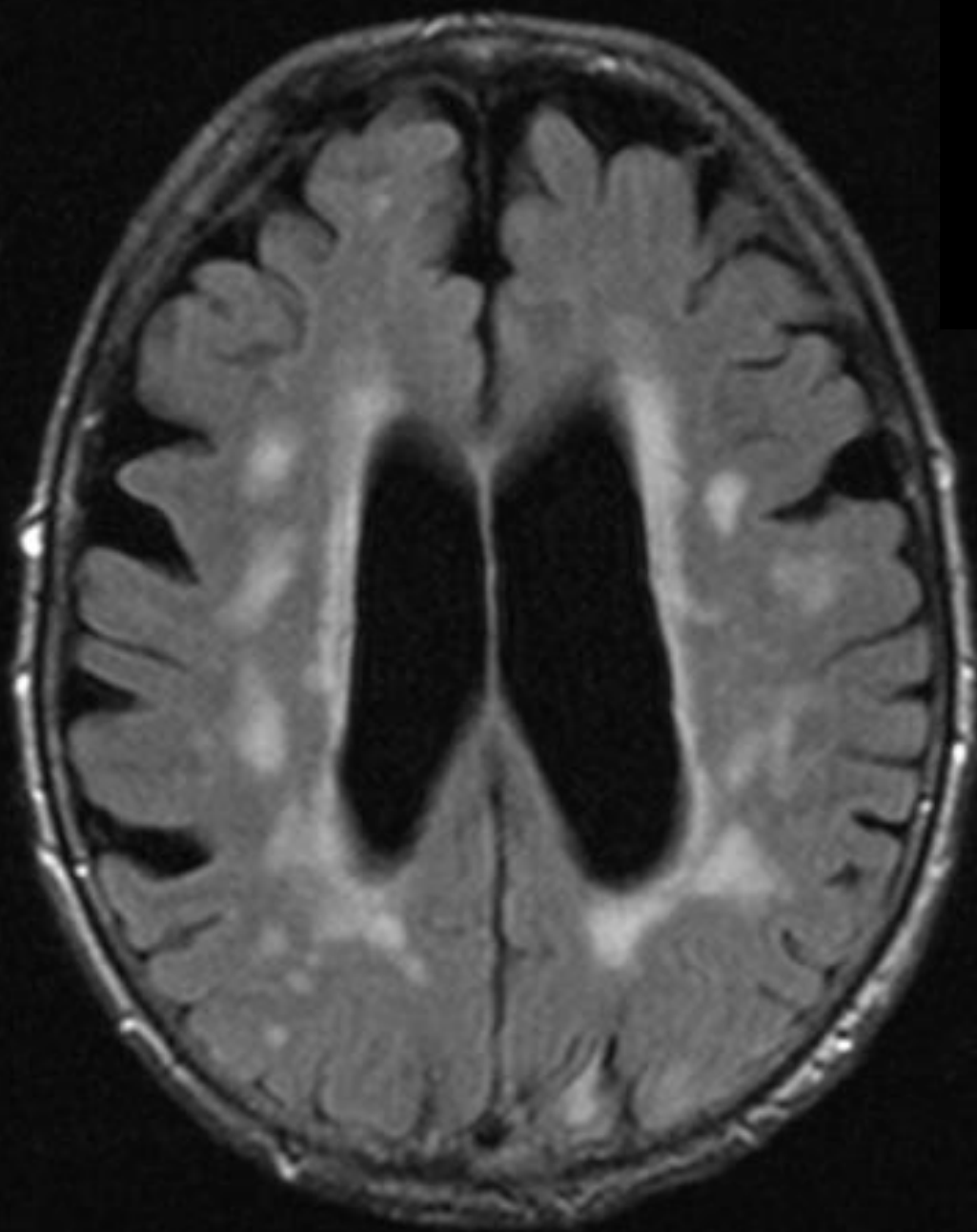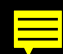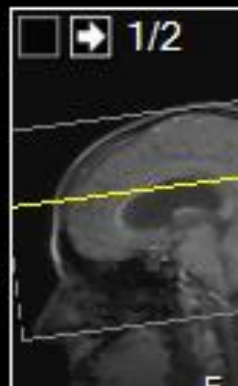

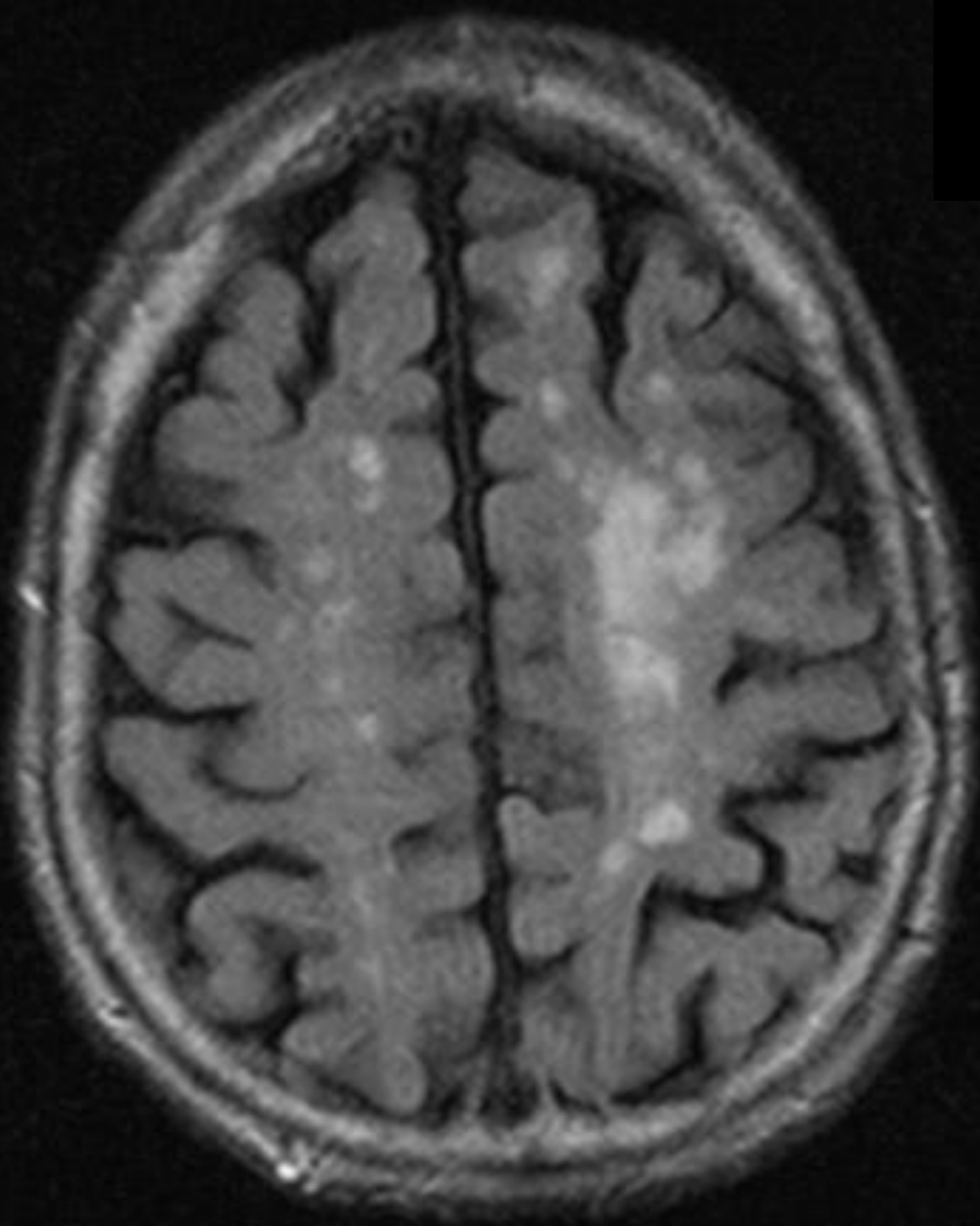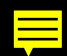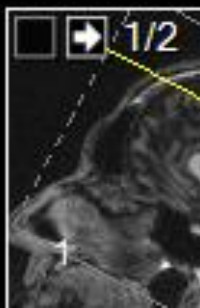

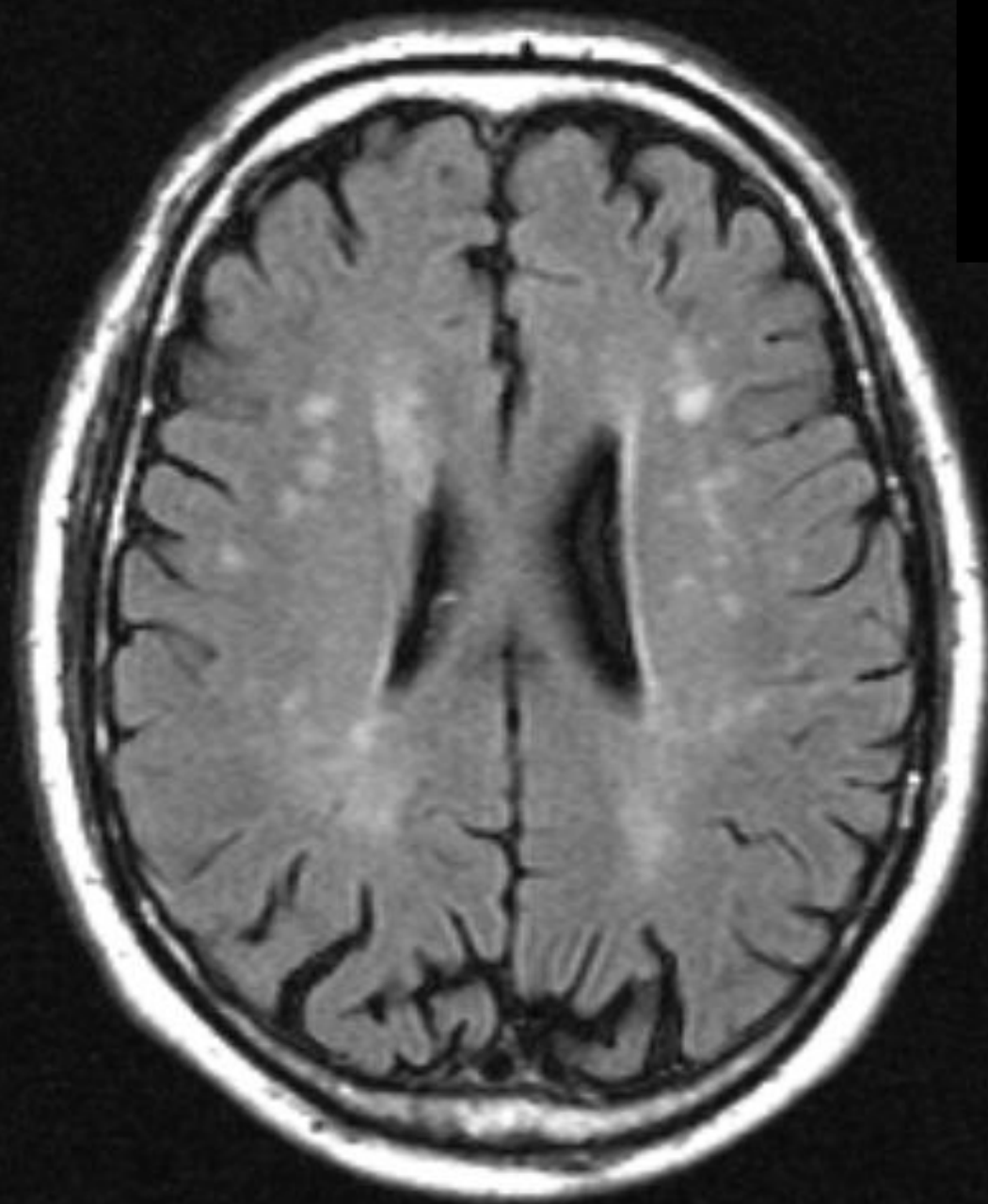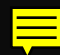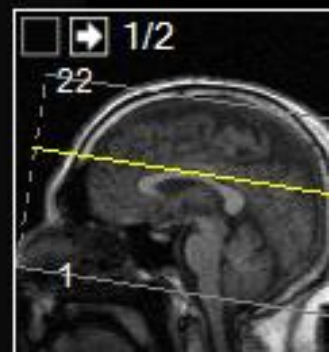

10

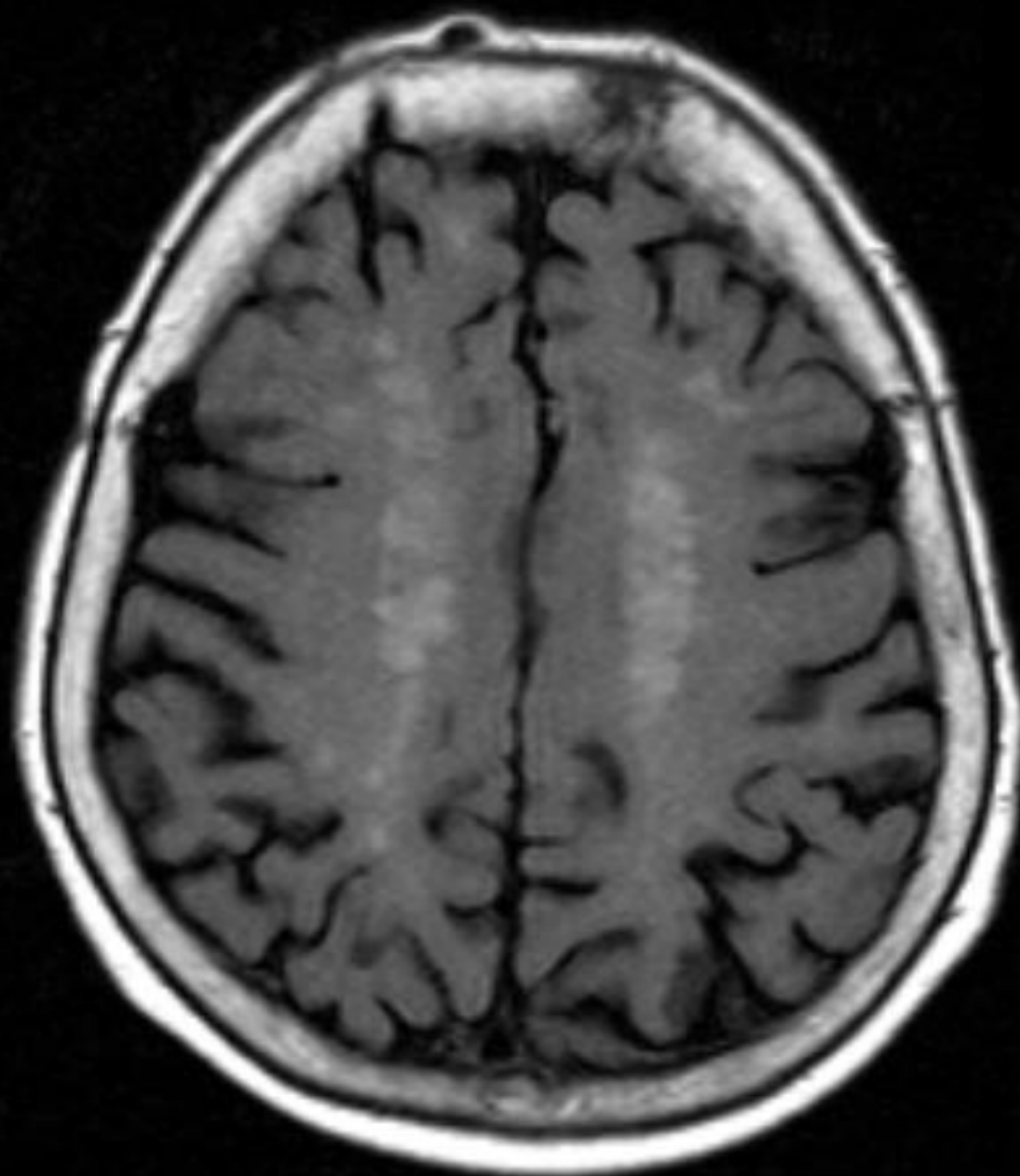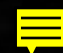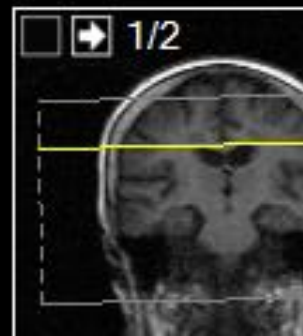

11

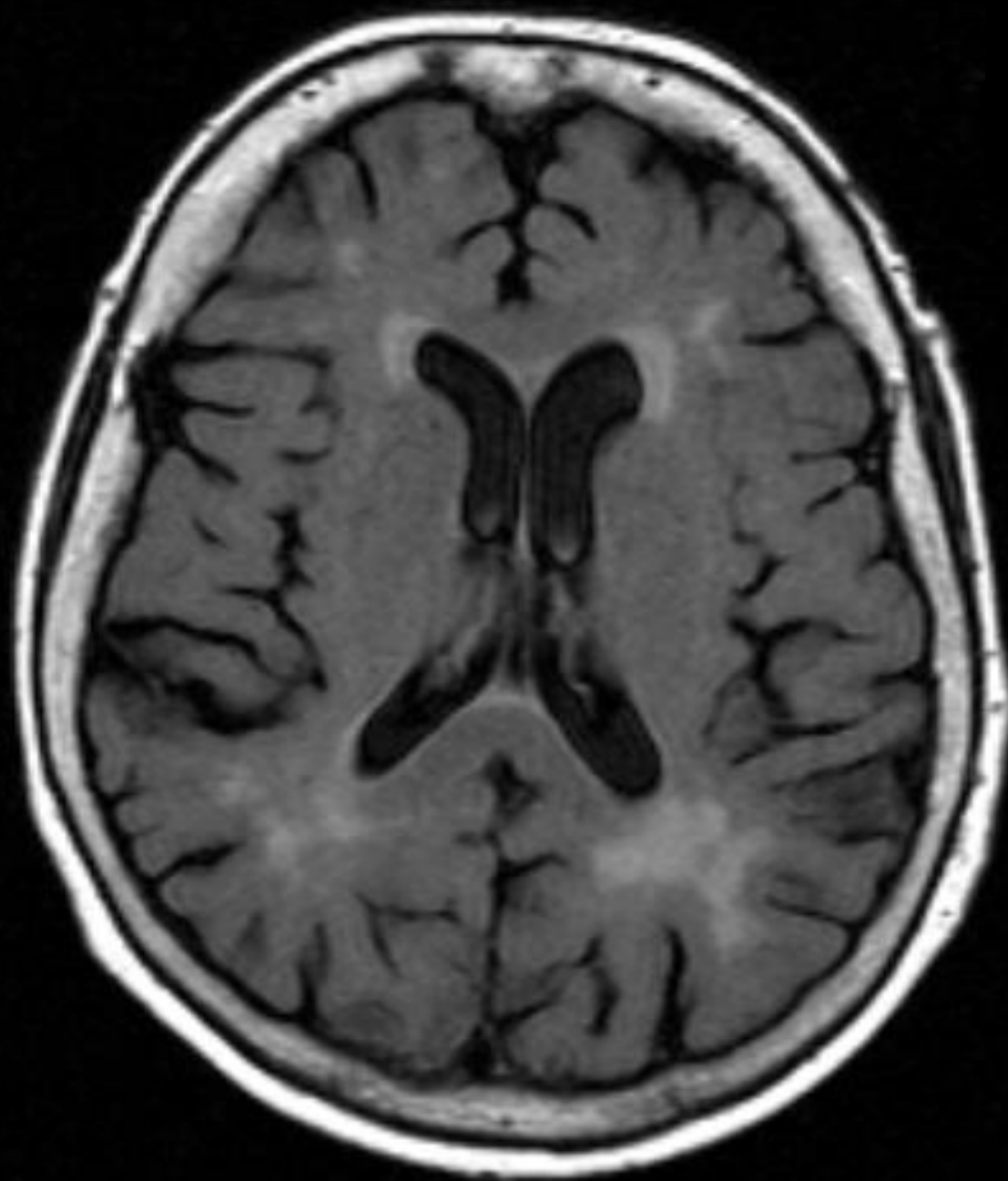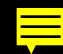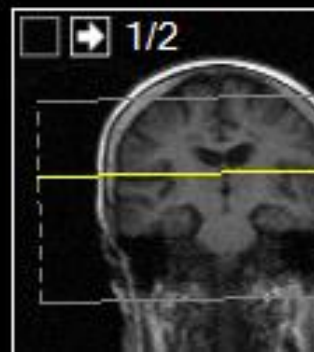

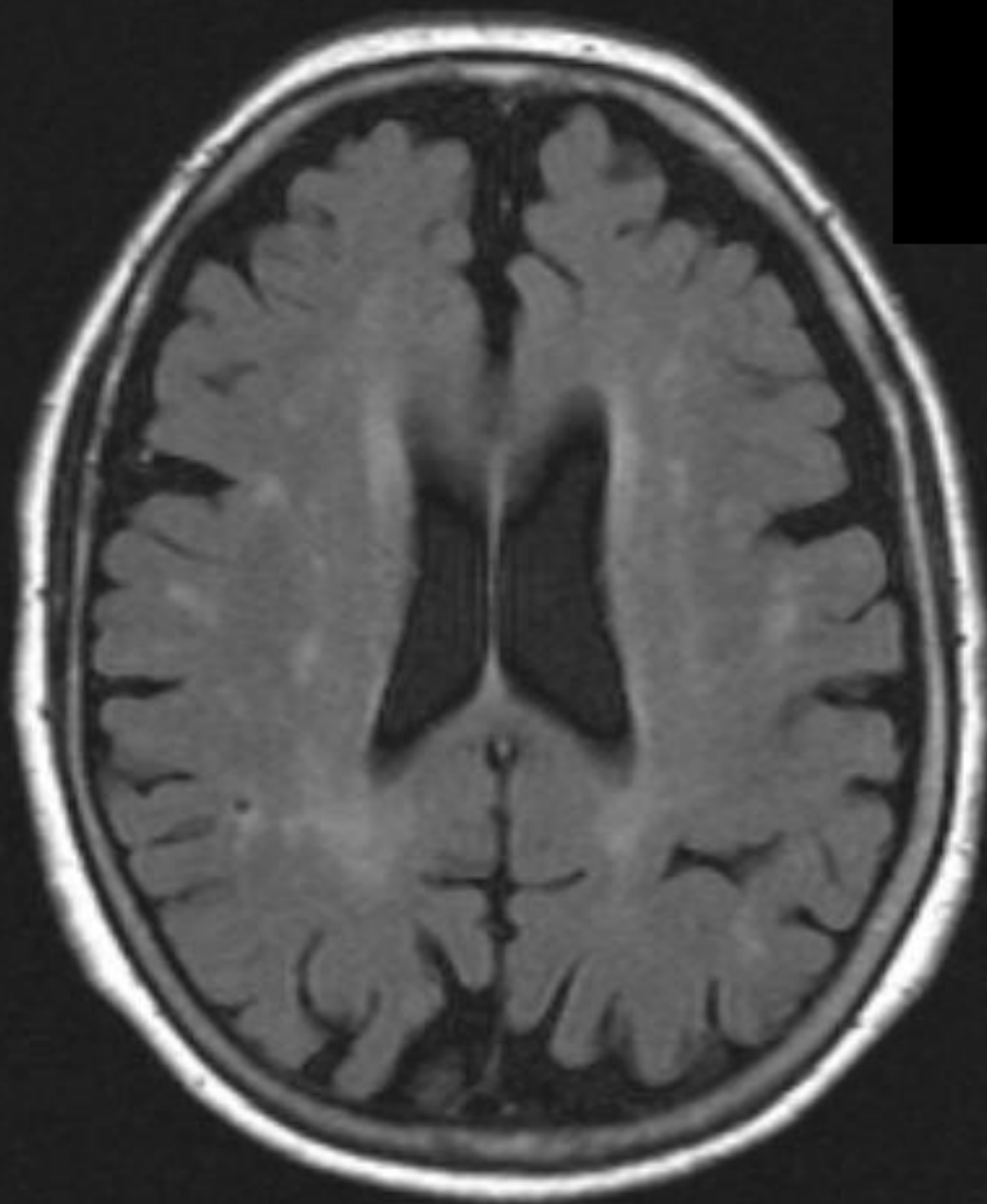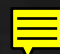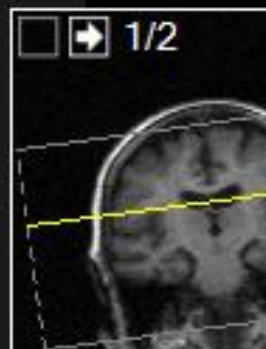

13

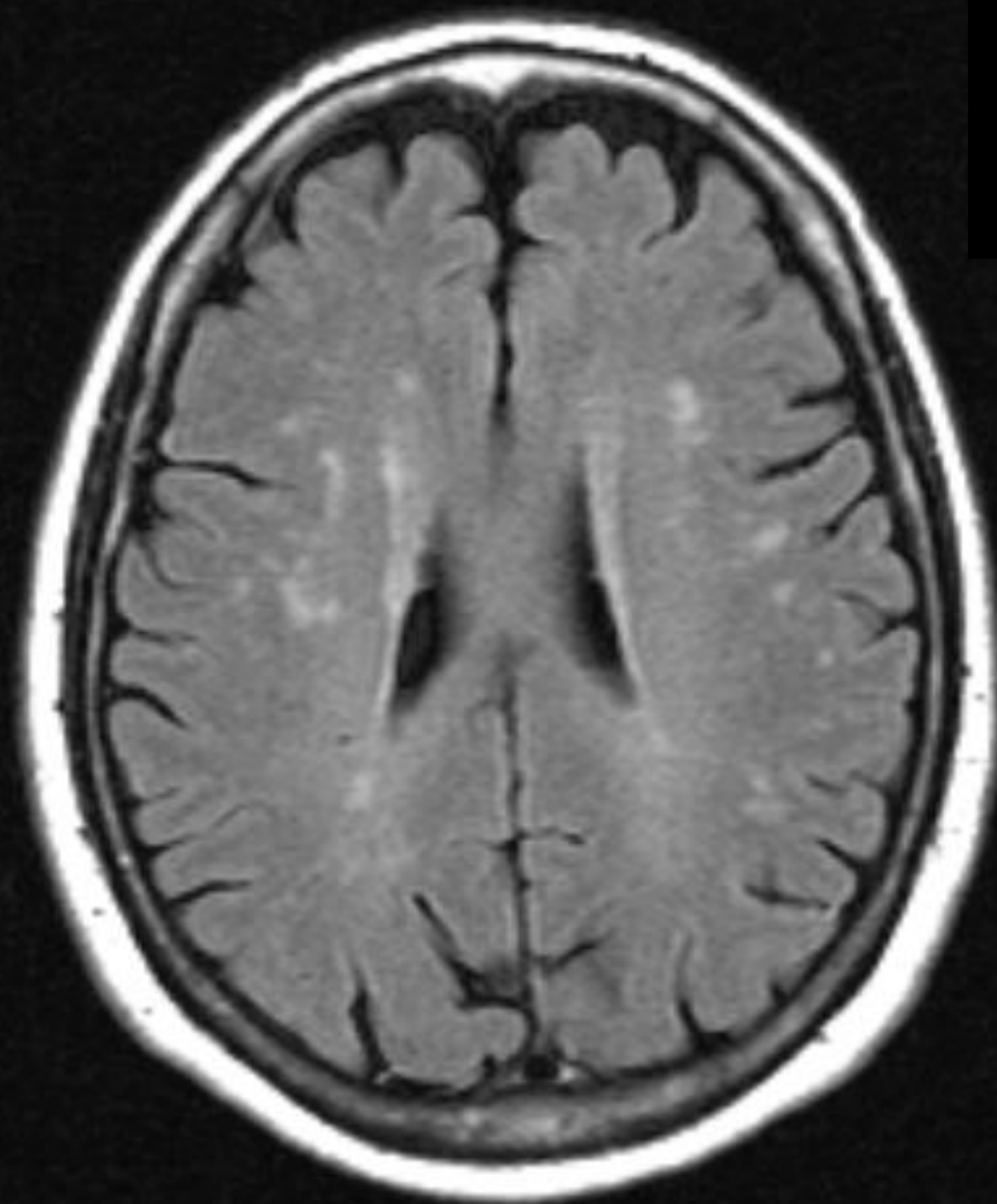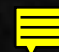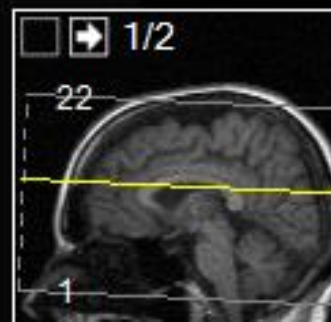

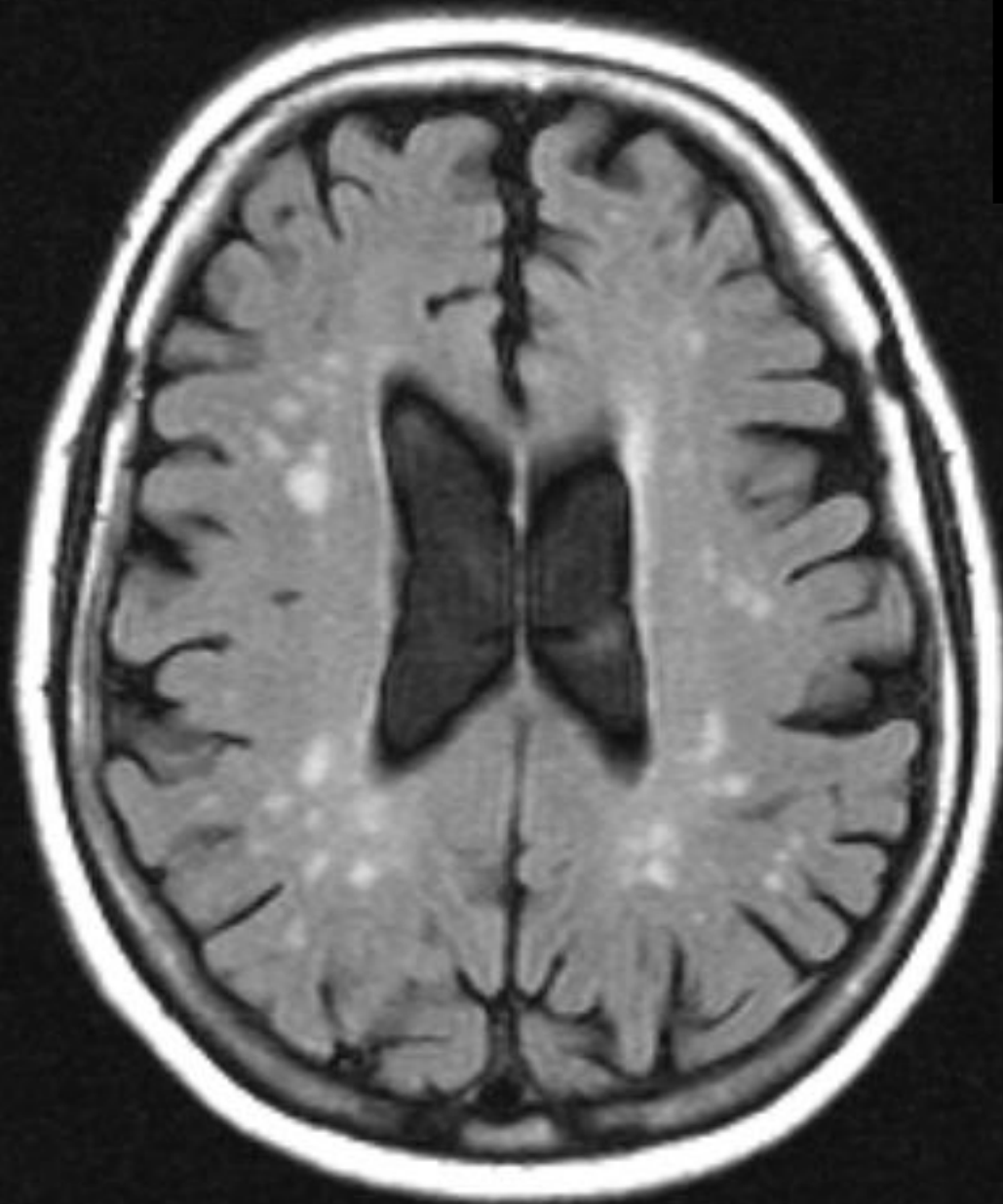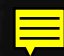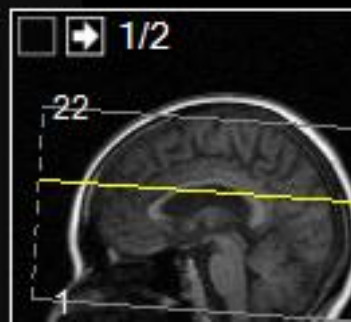

15

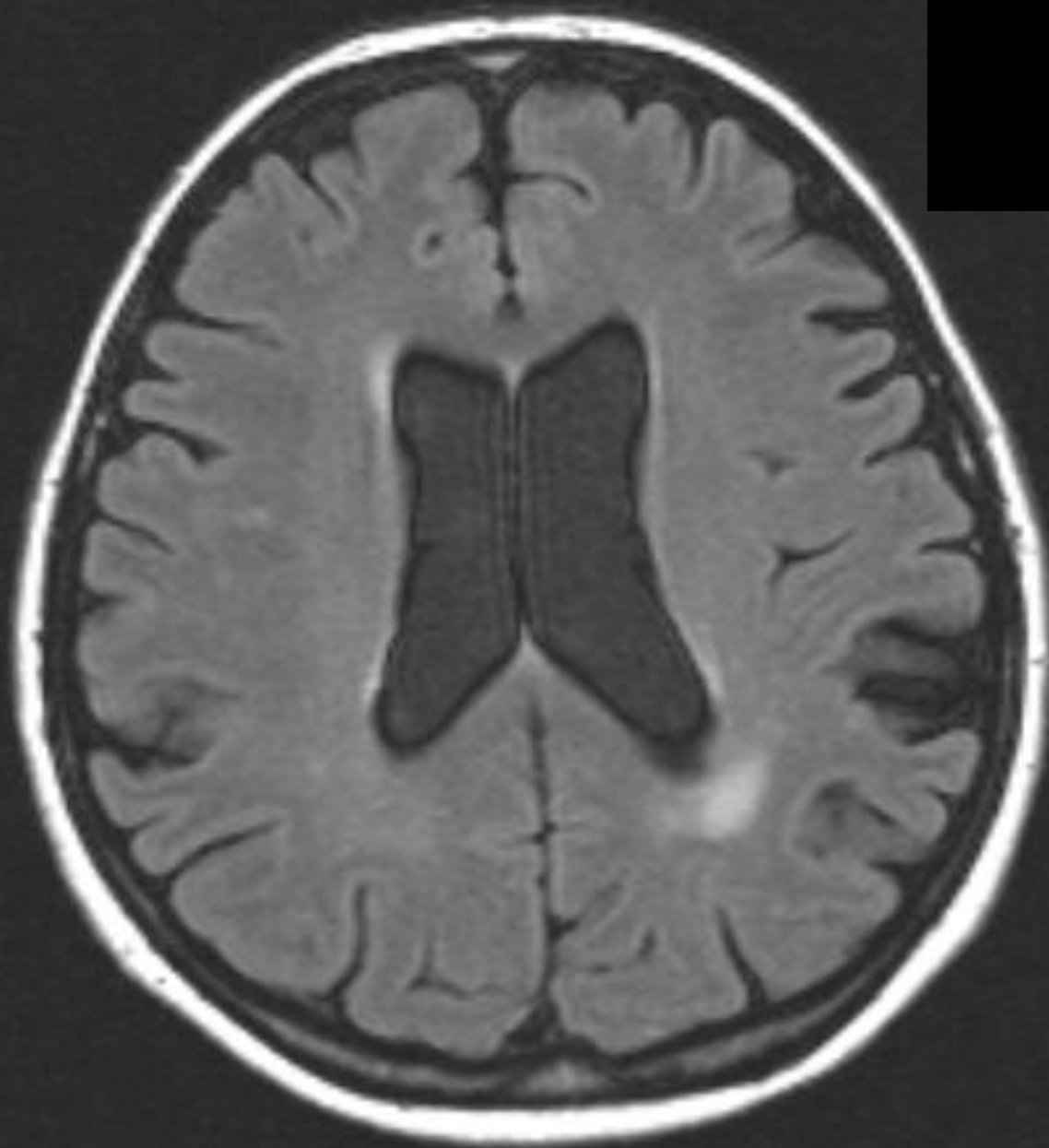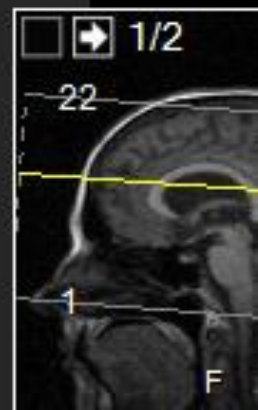

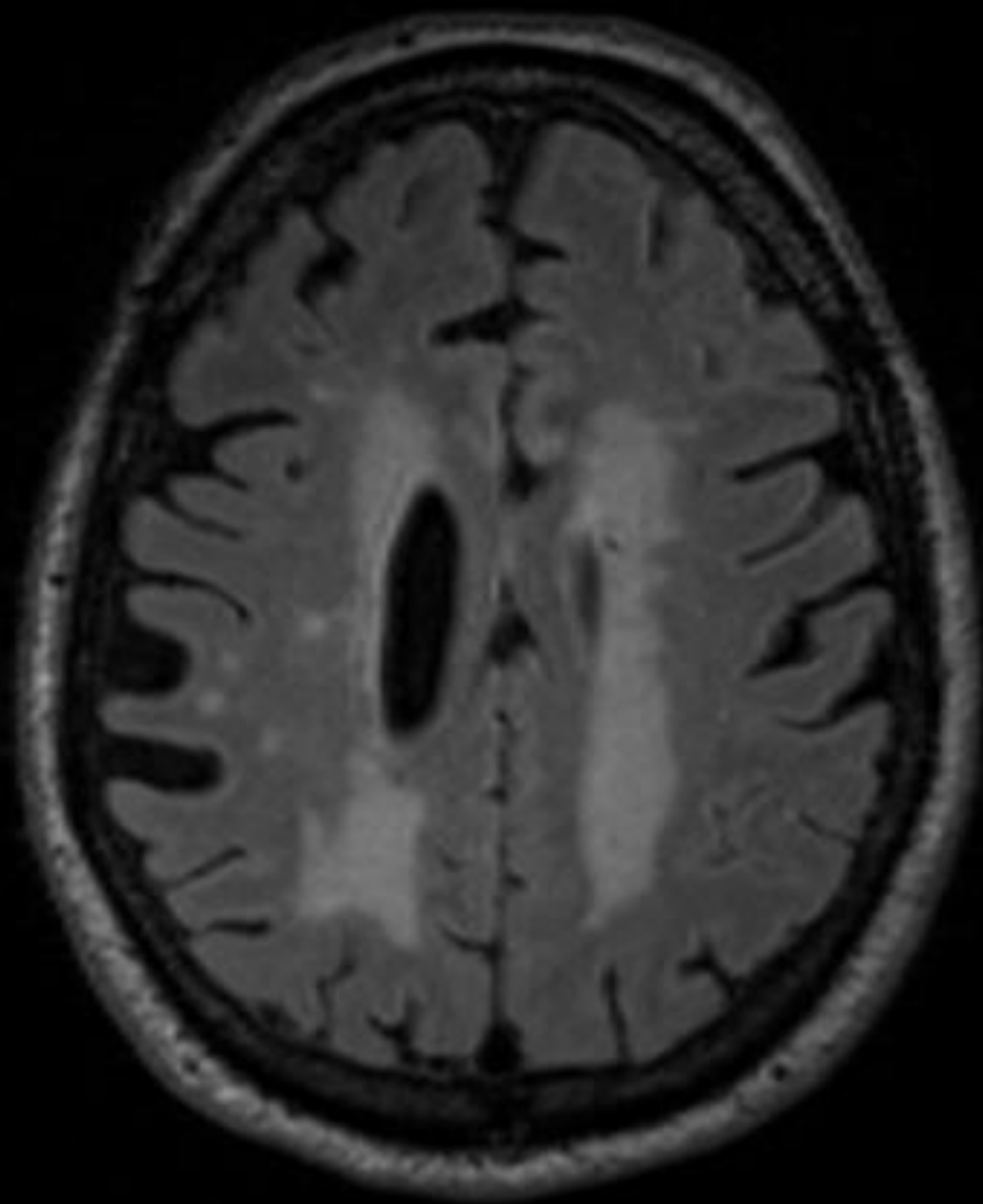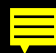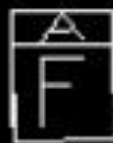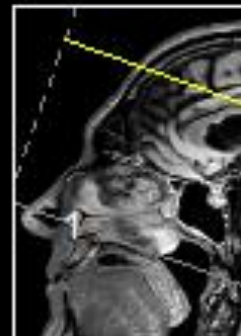

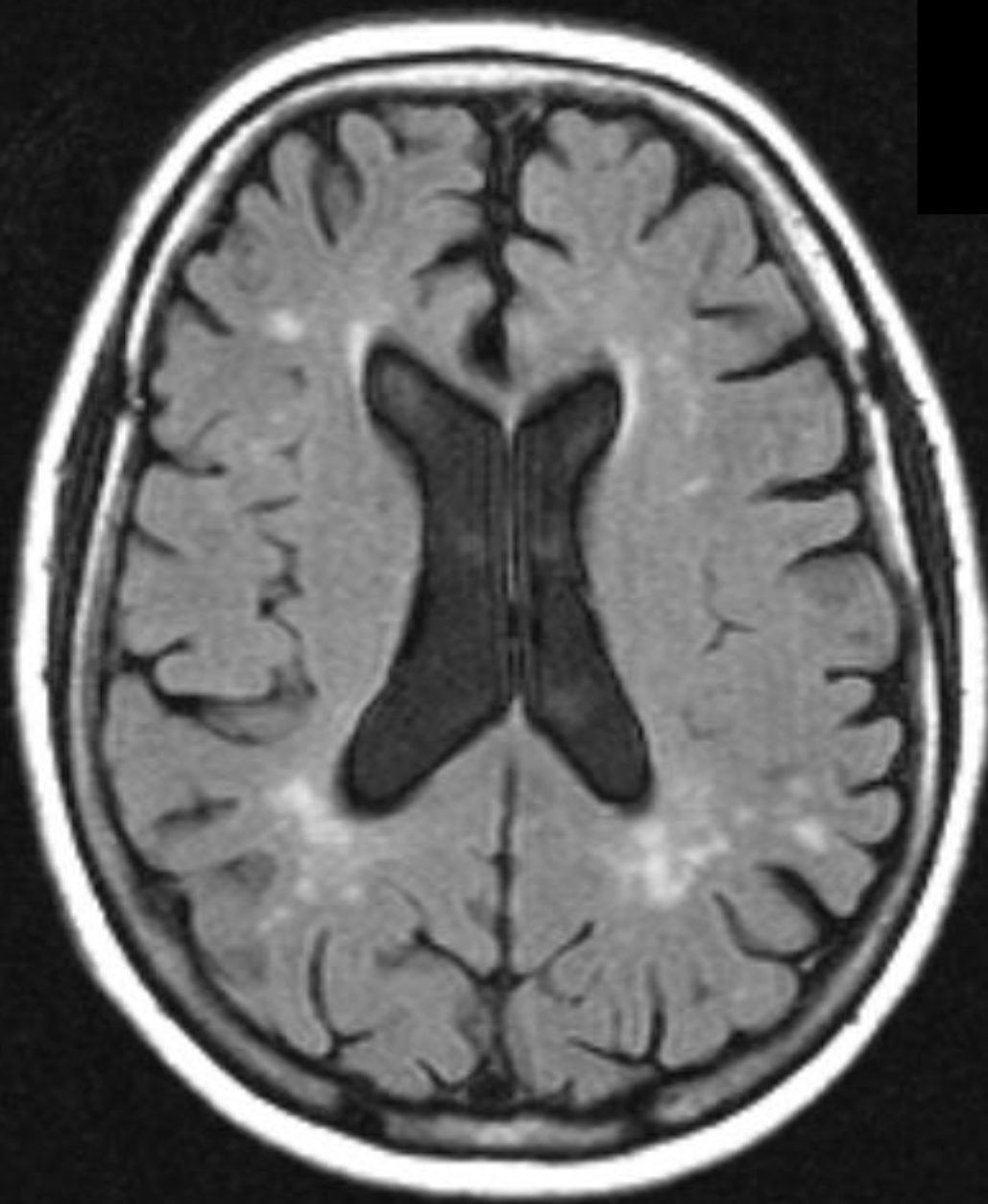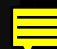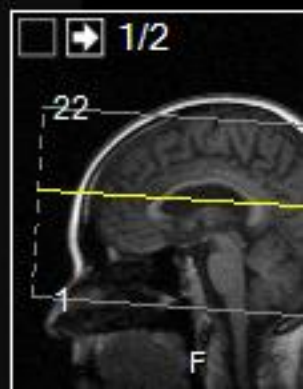

18

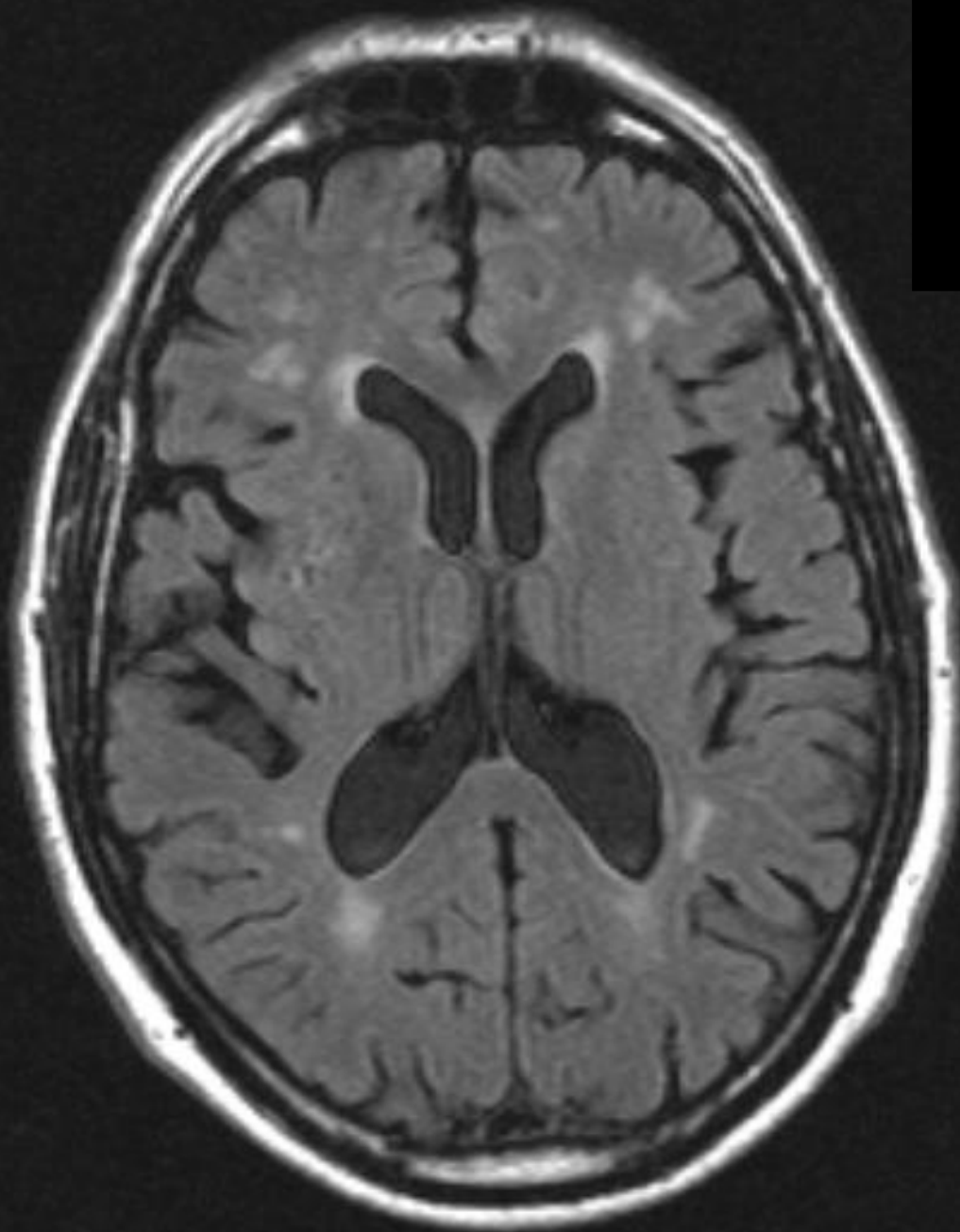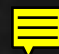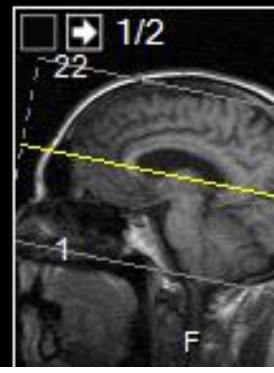

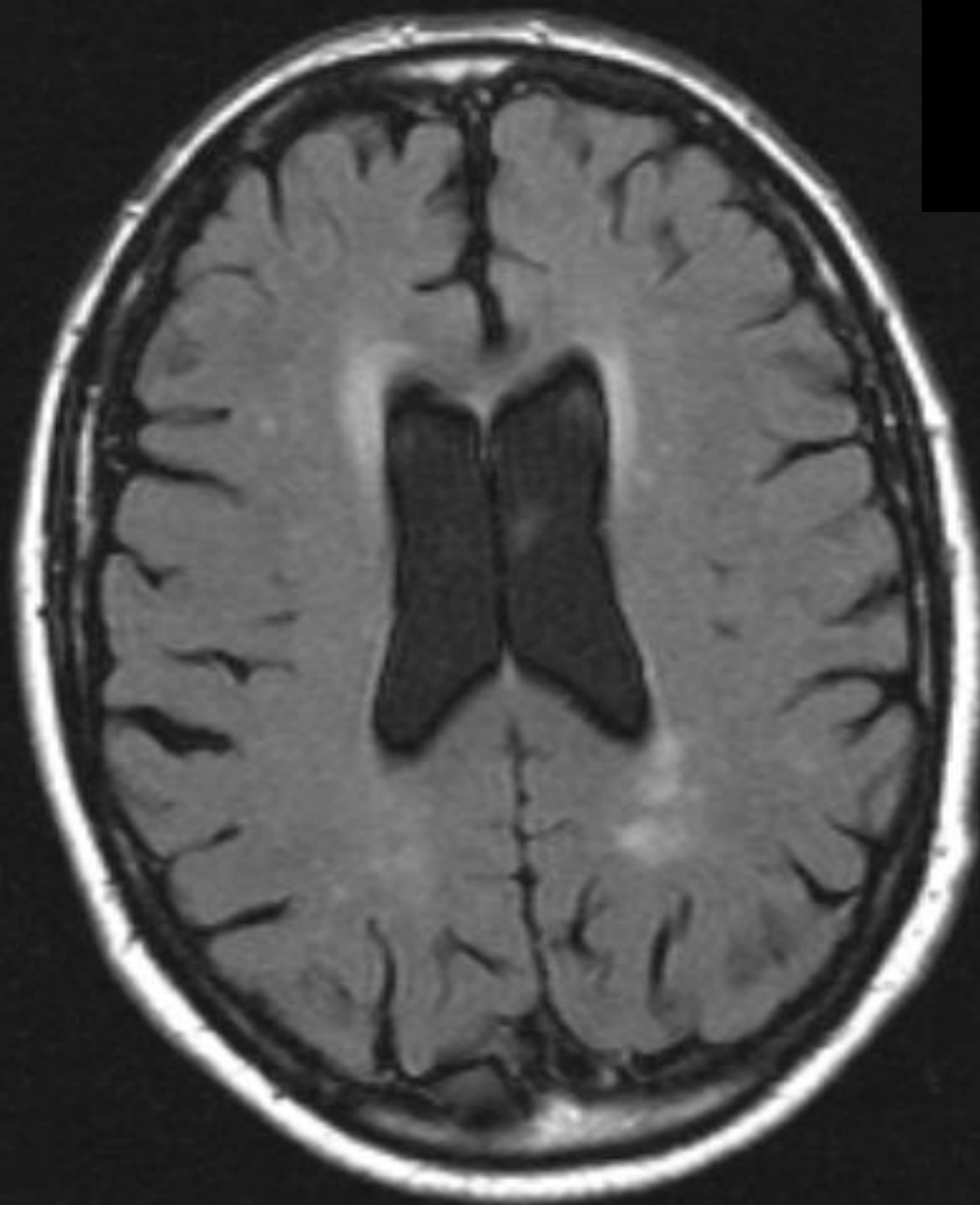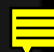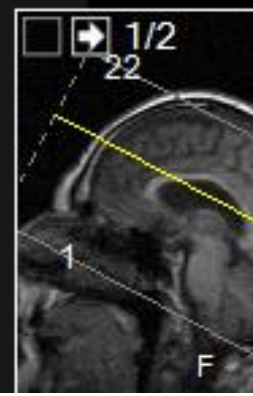

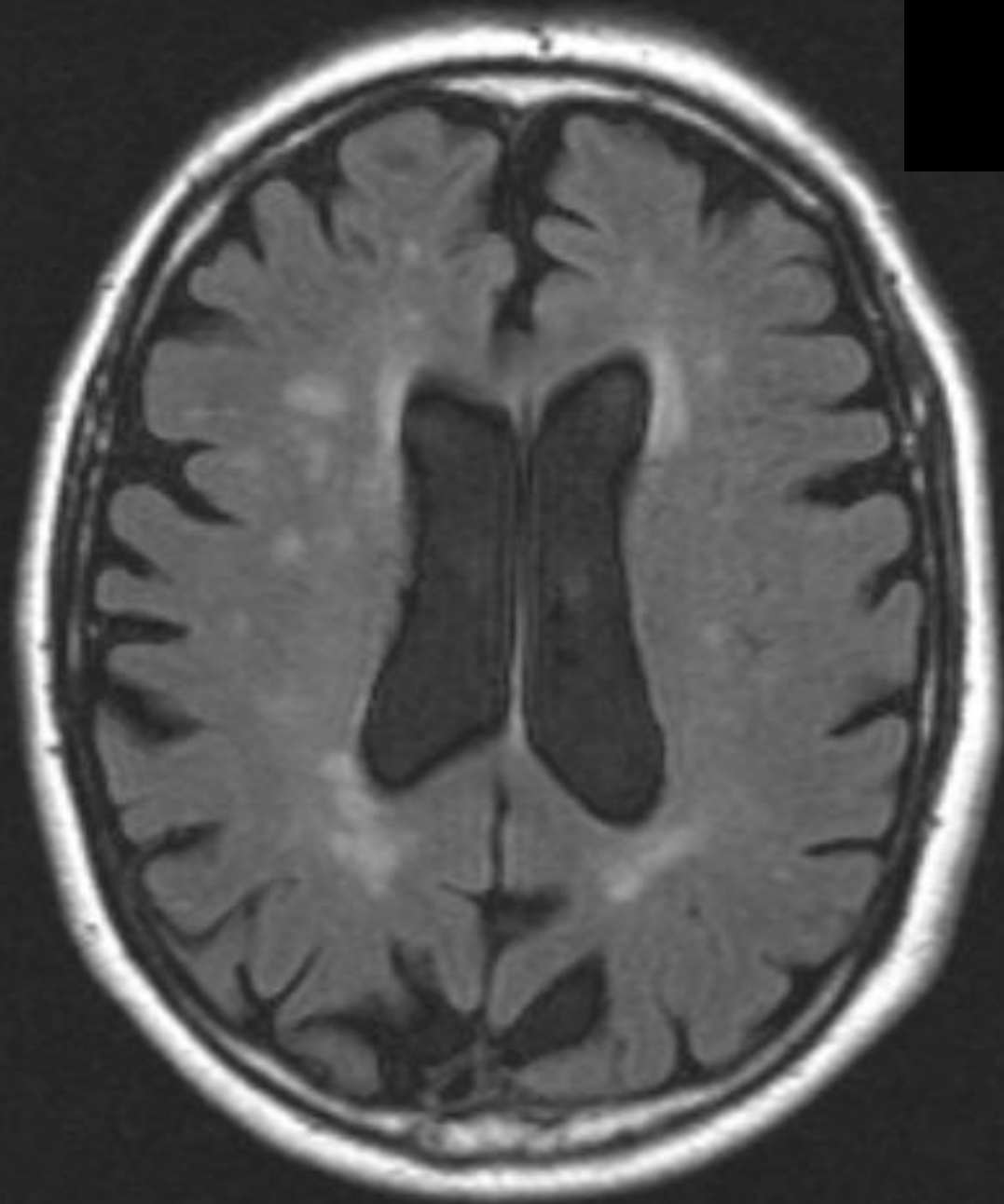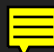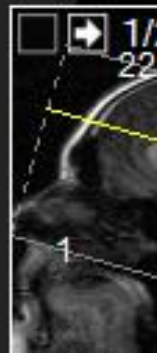

Supplement: Supplementary file 7 — (PDF 821 kb) [file 13244_2016_521_MOESM7_ESM.pdf]

1

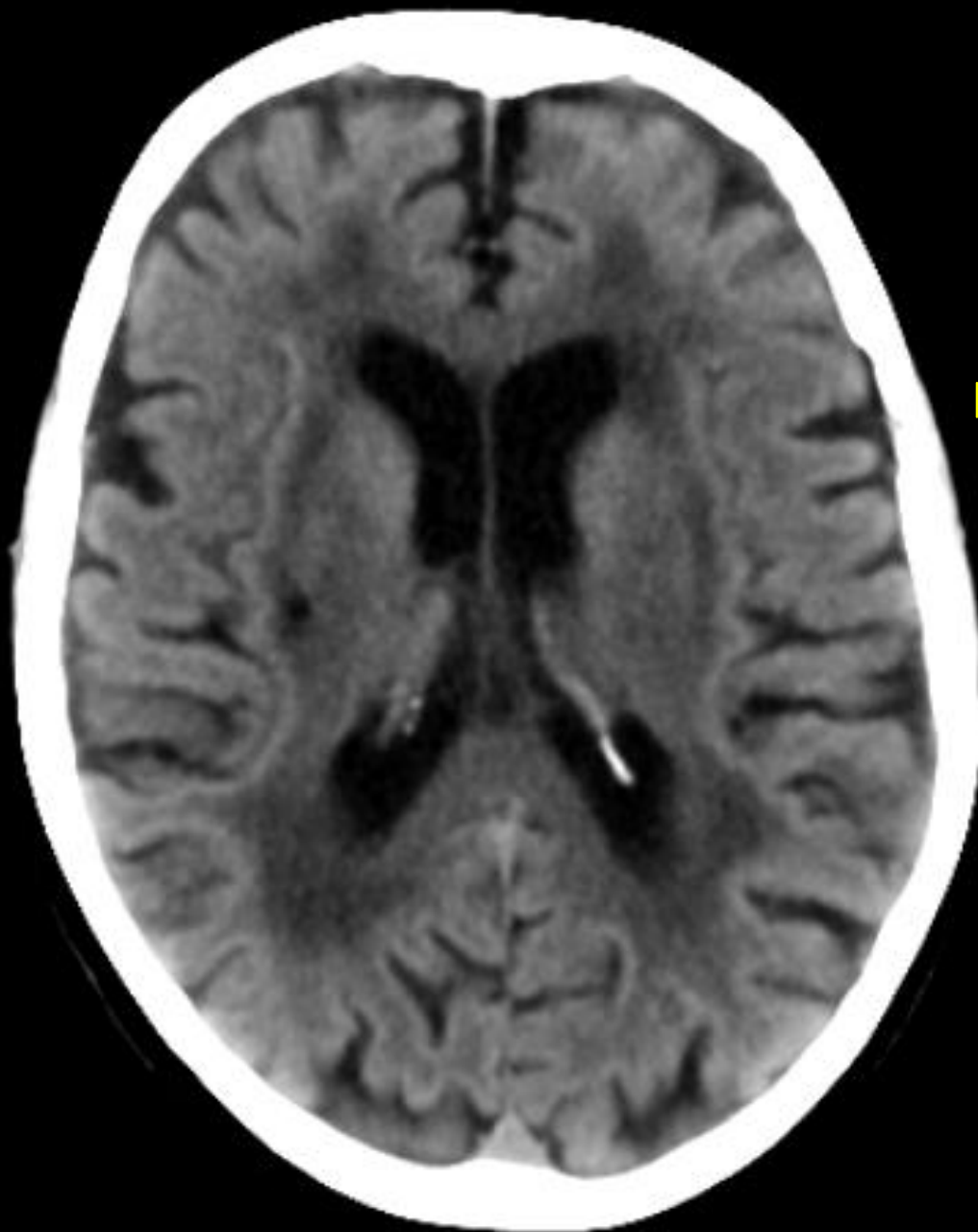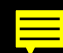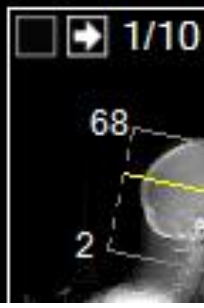

2

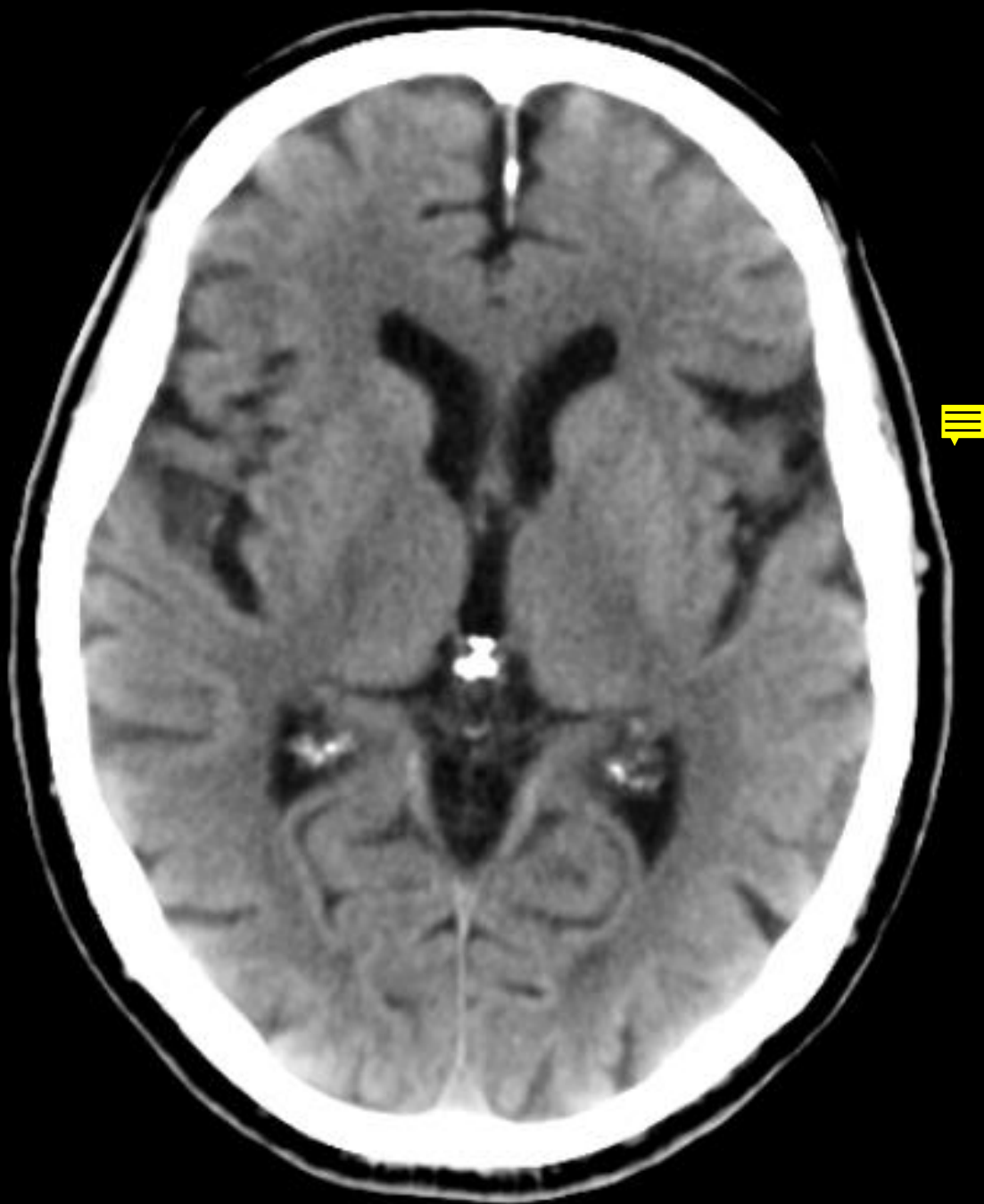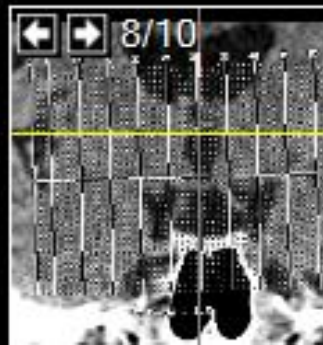

3

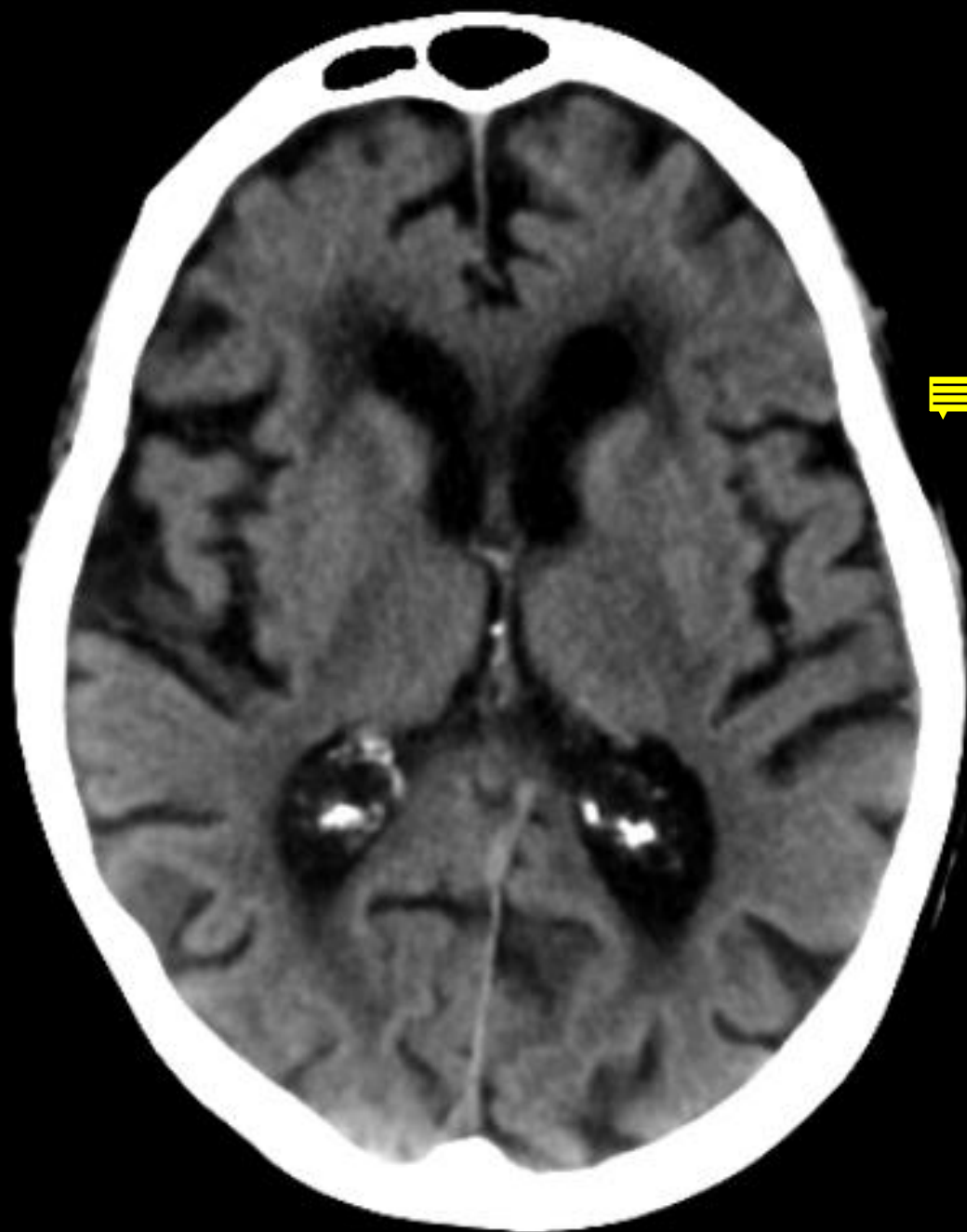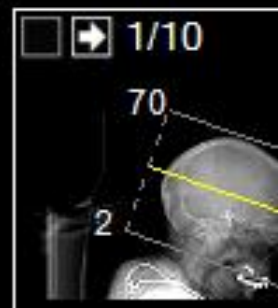

4

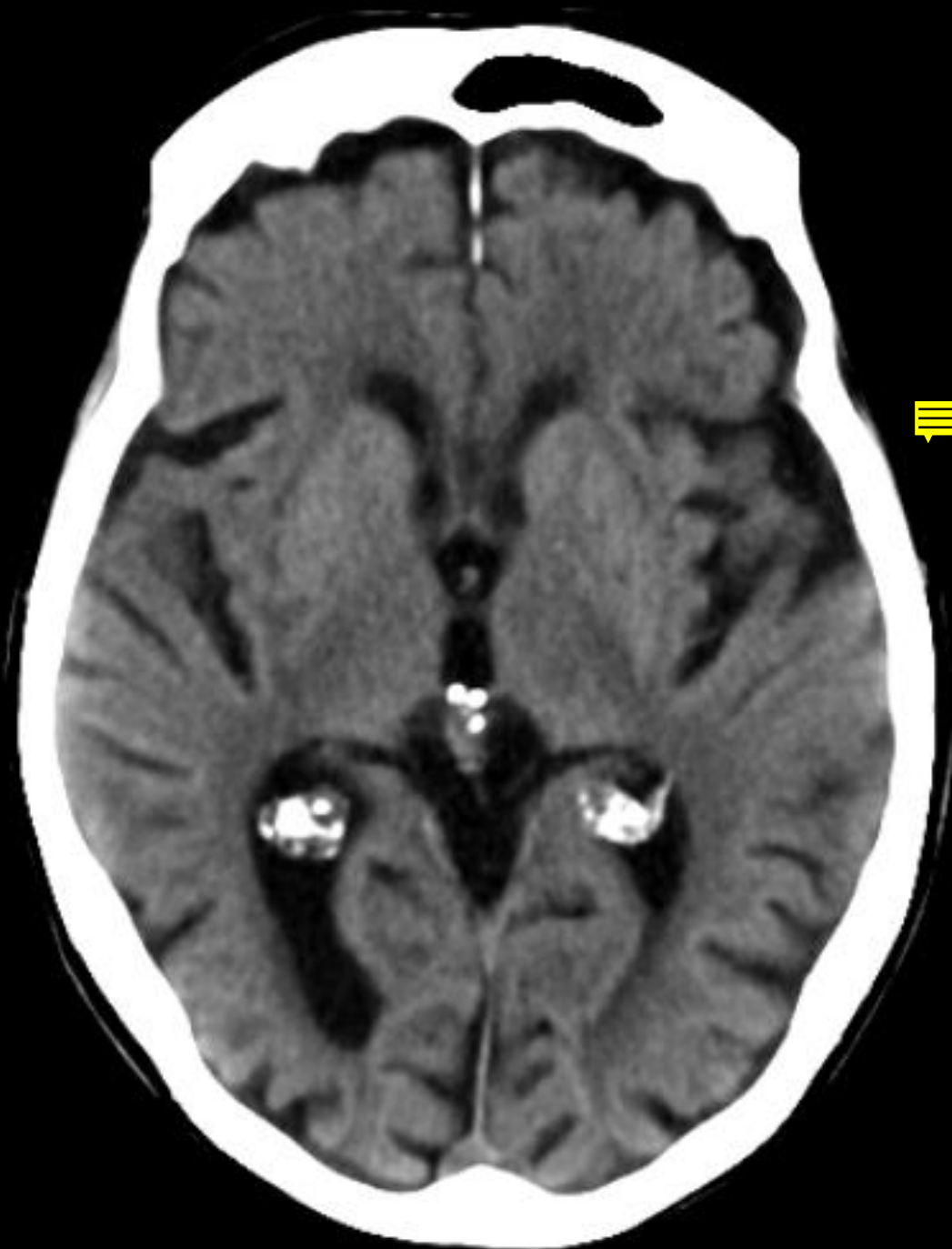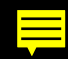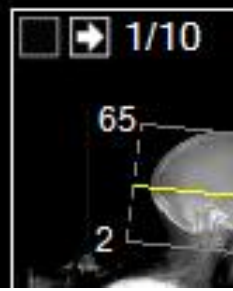

5

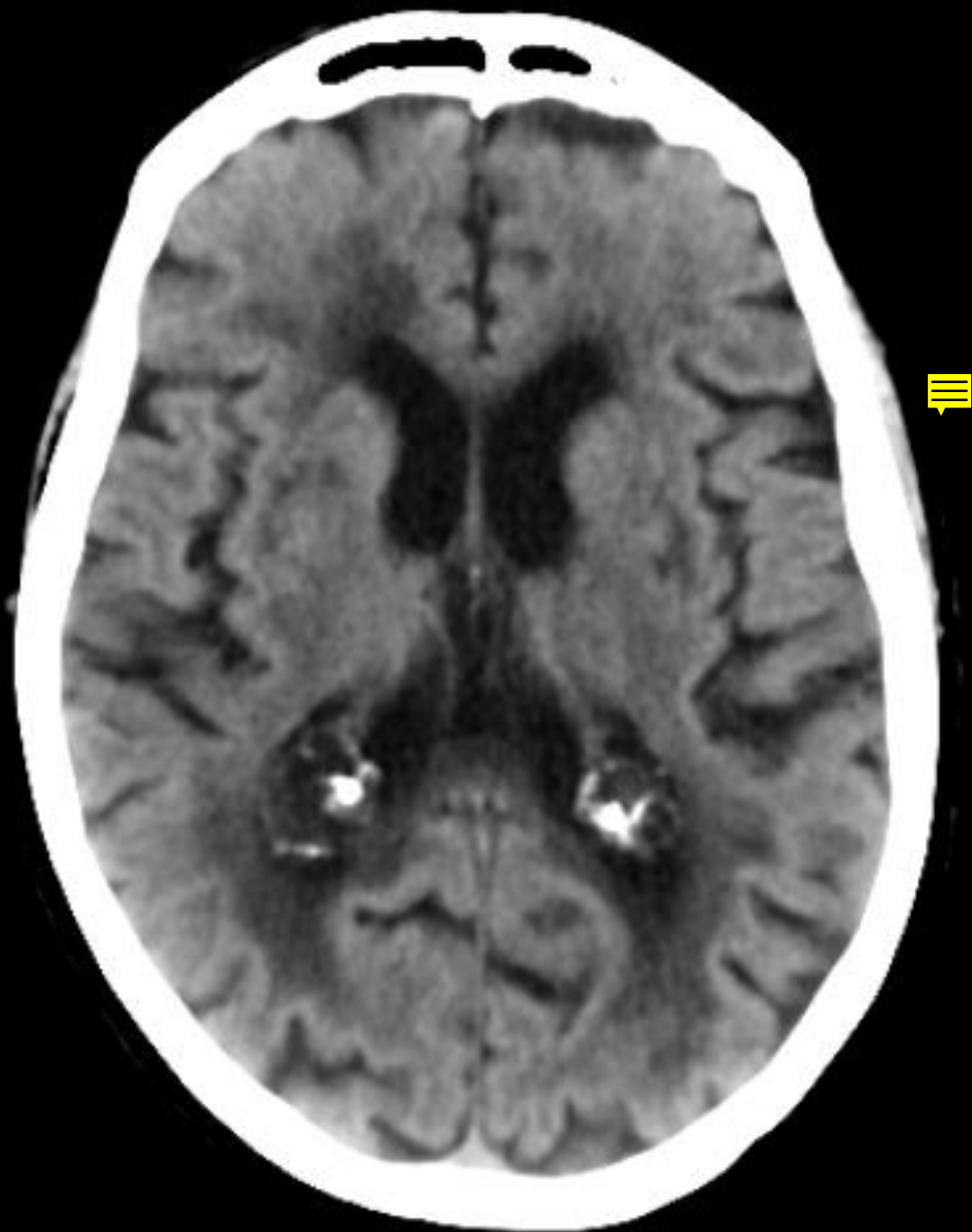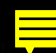

1/10

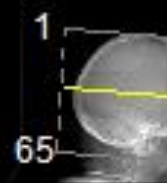

6

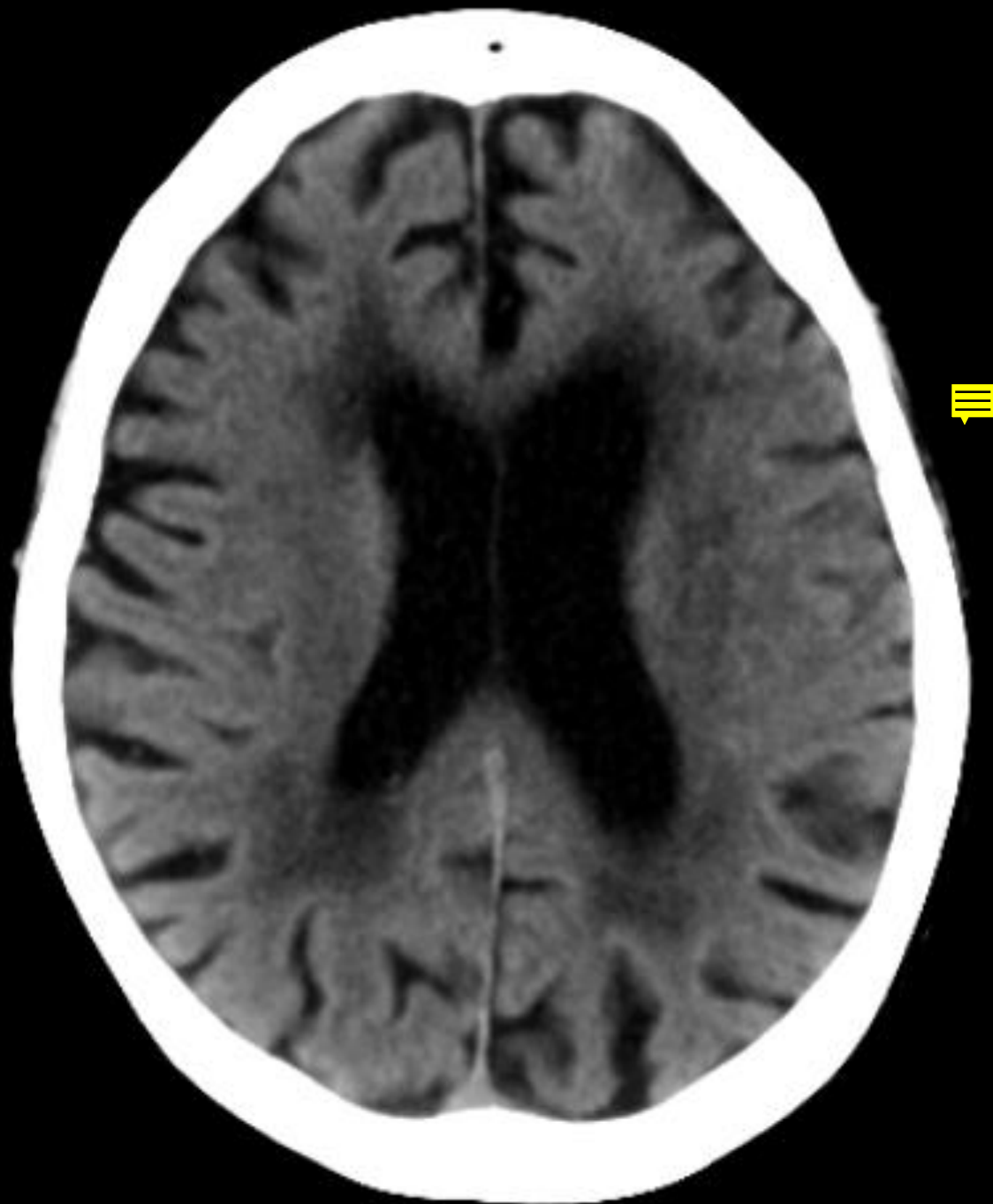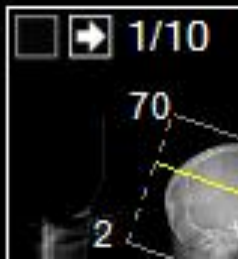

7

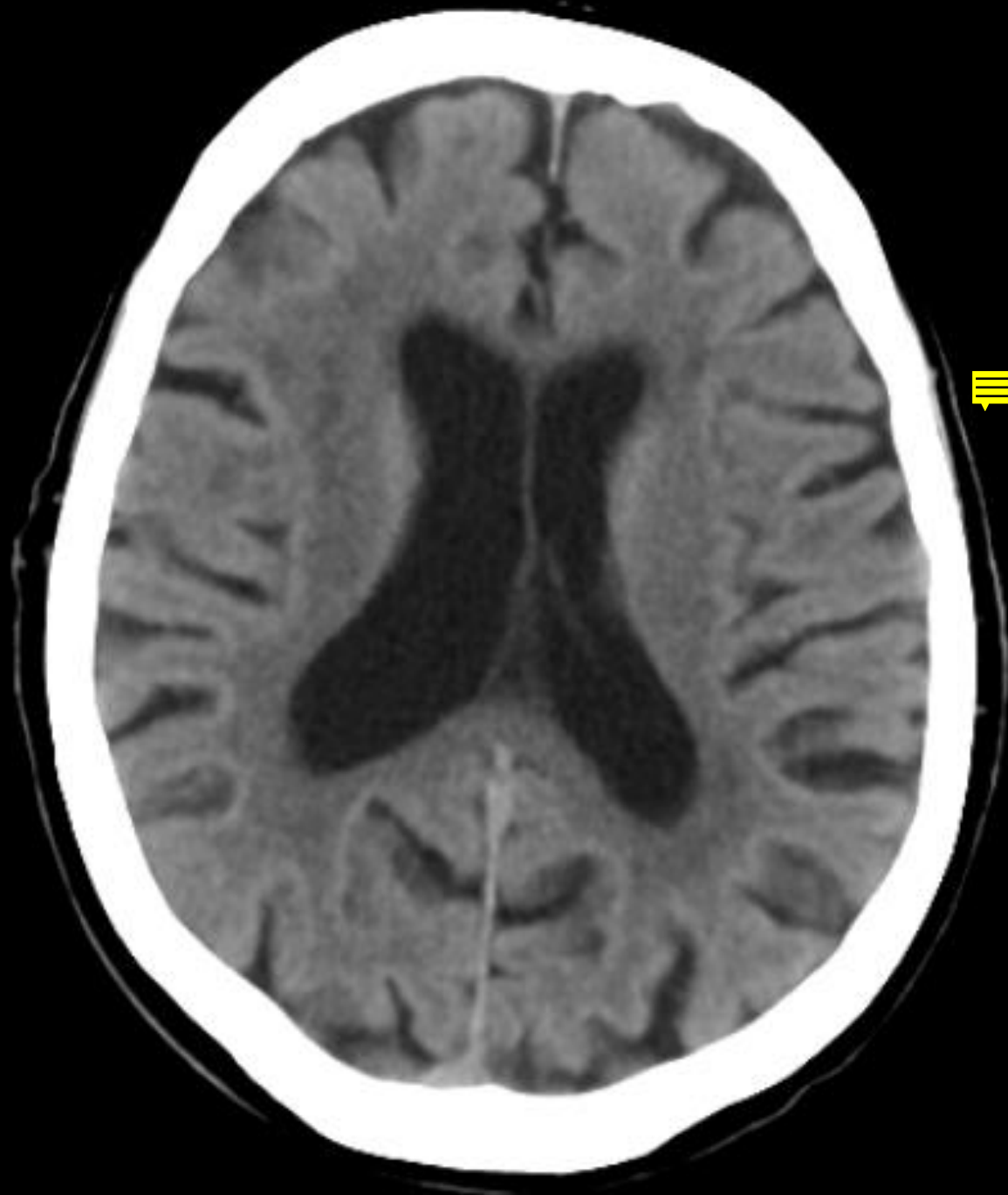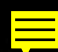

1/10

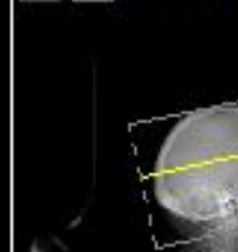

8

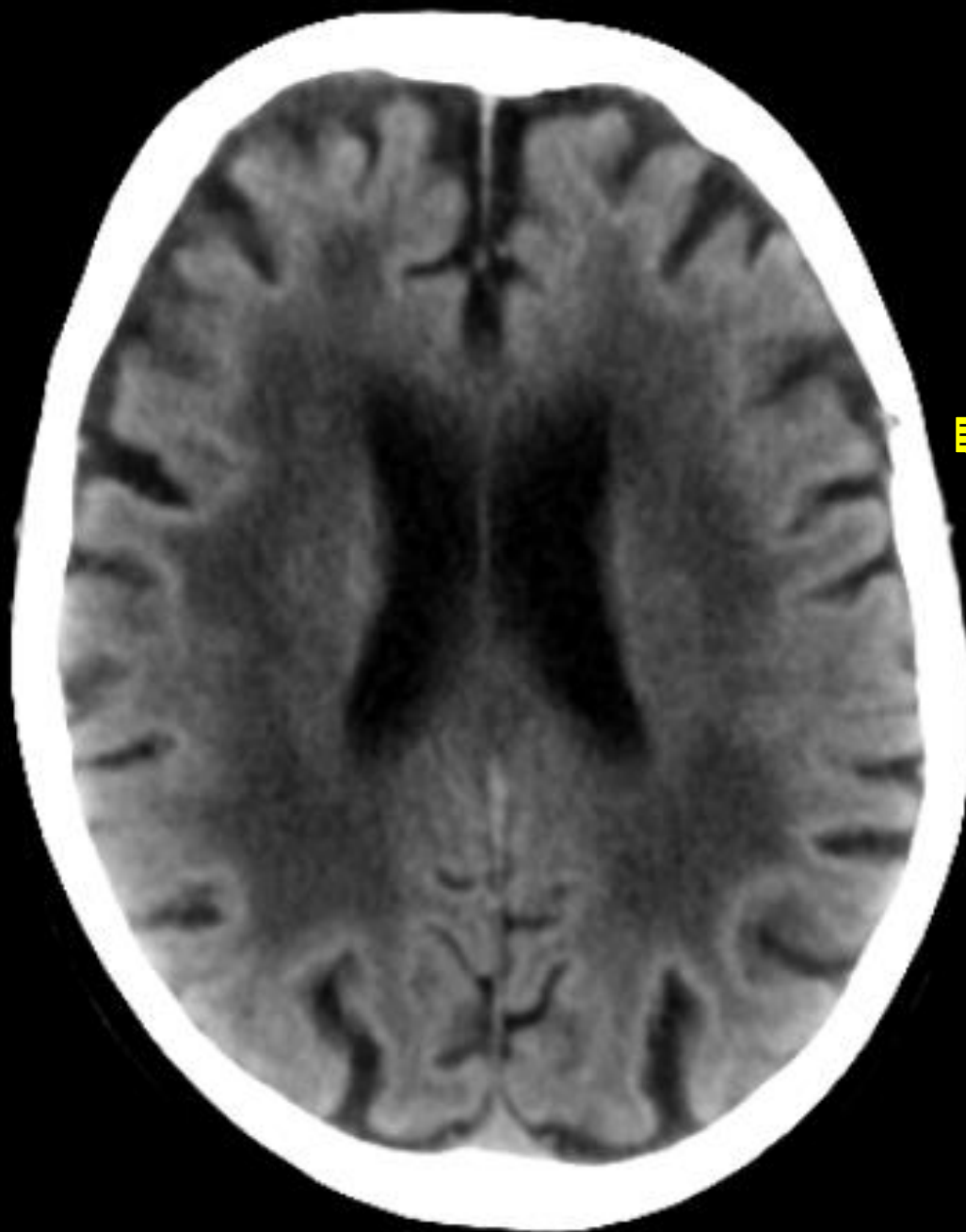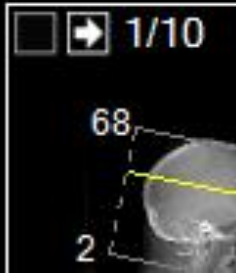

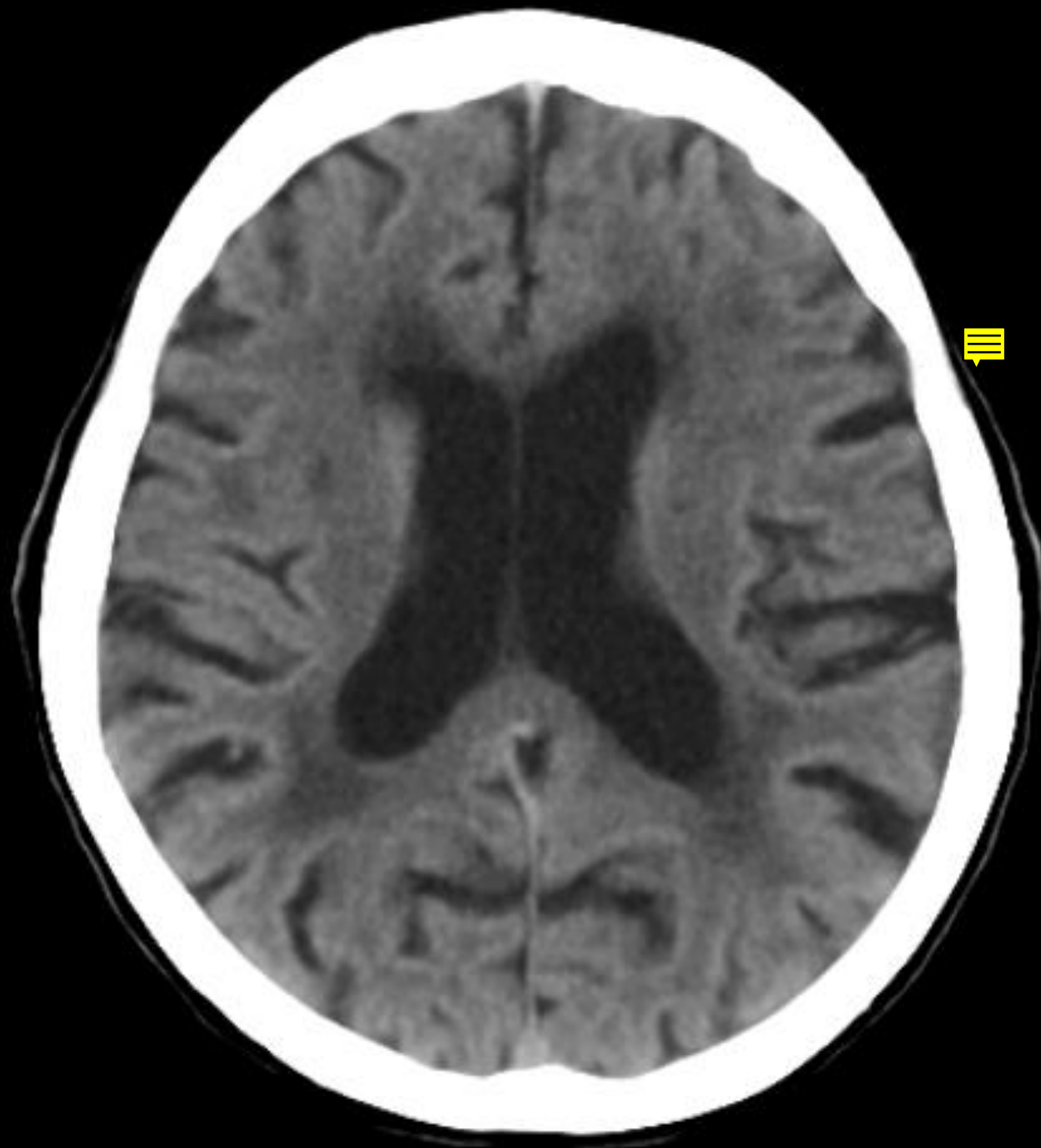

10

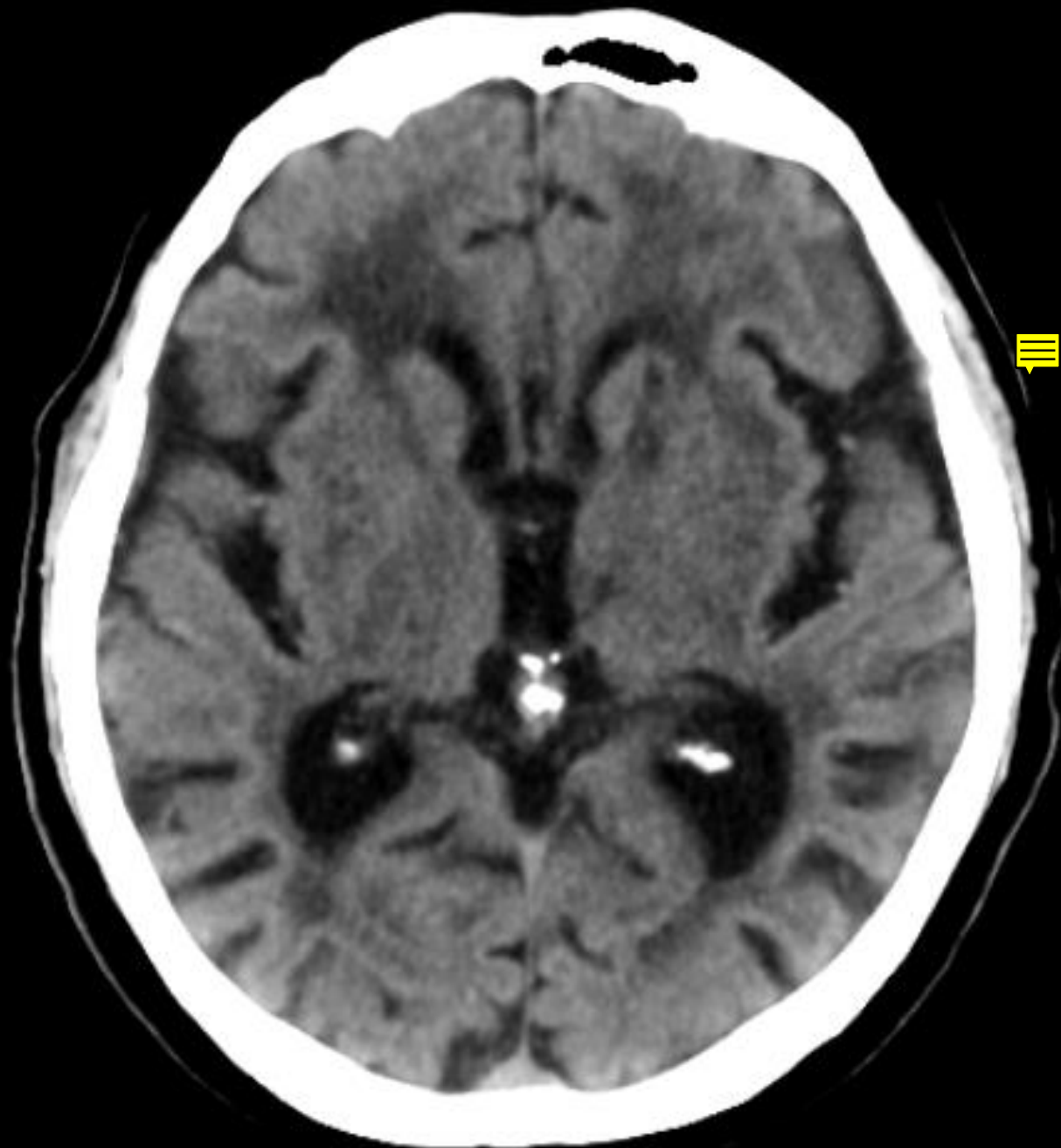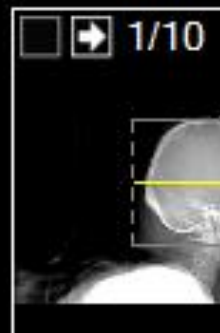

11

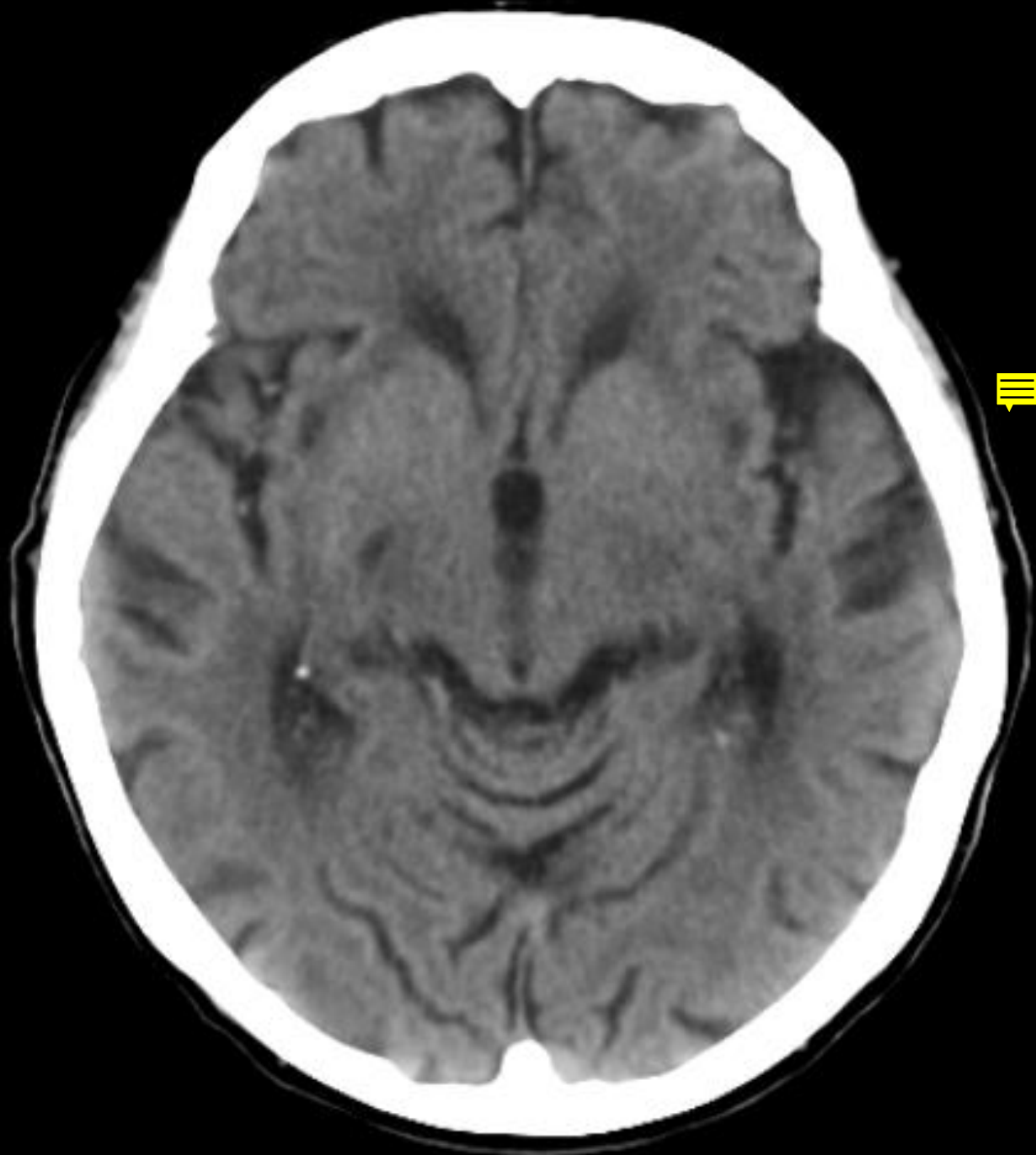

12

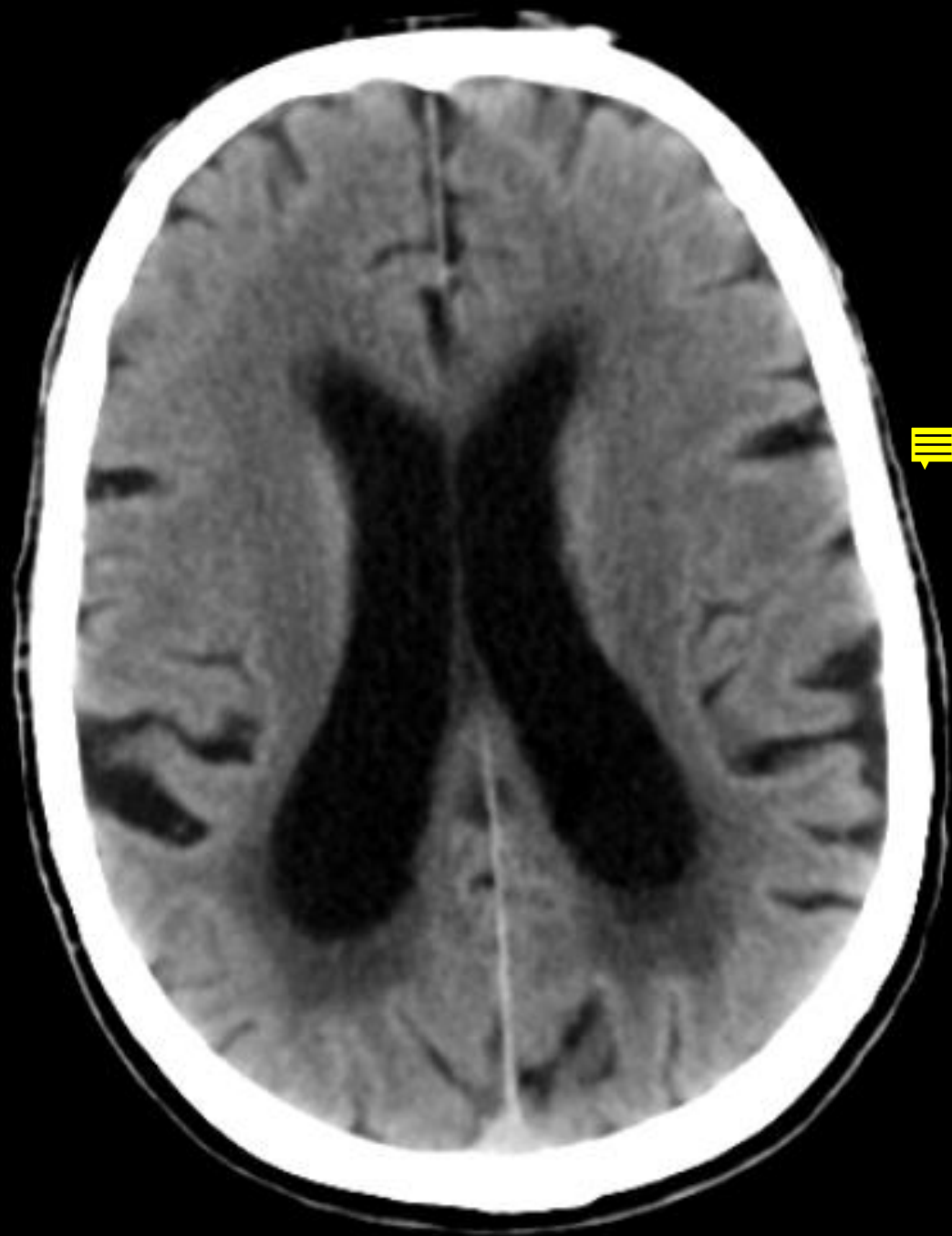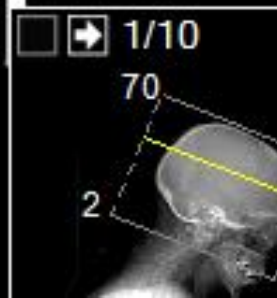

13

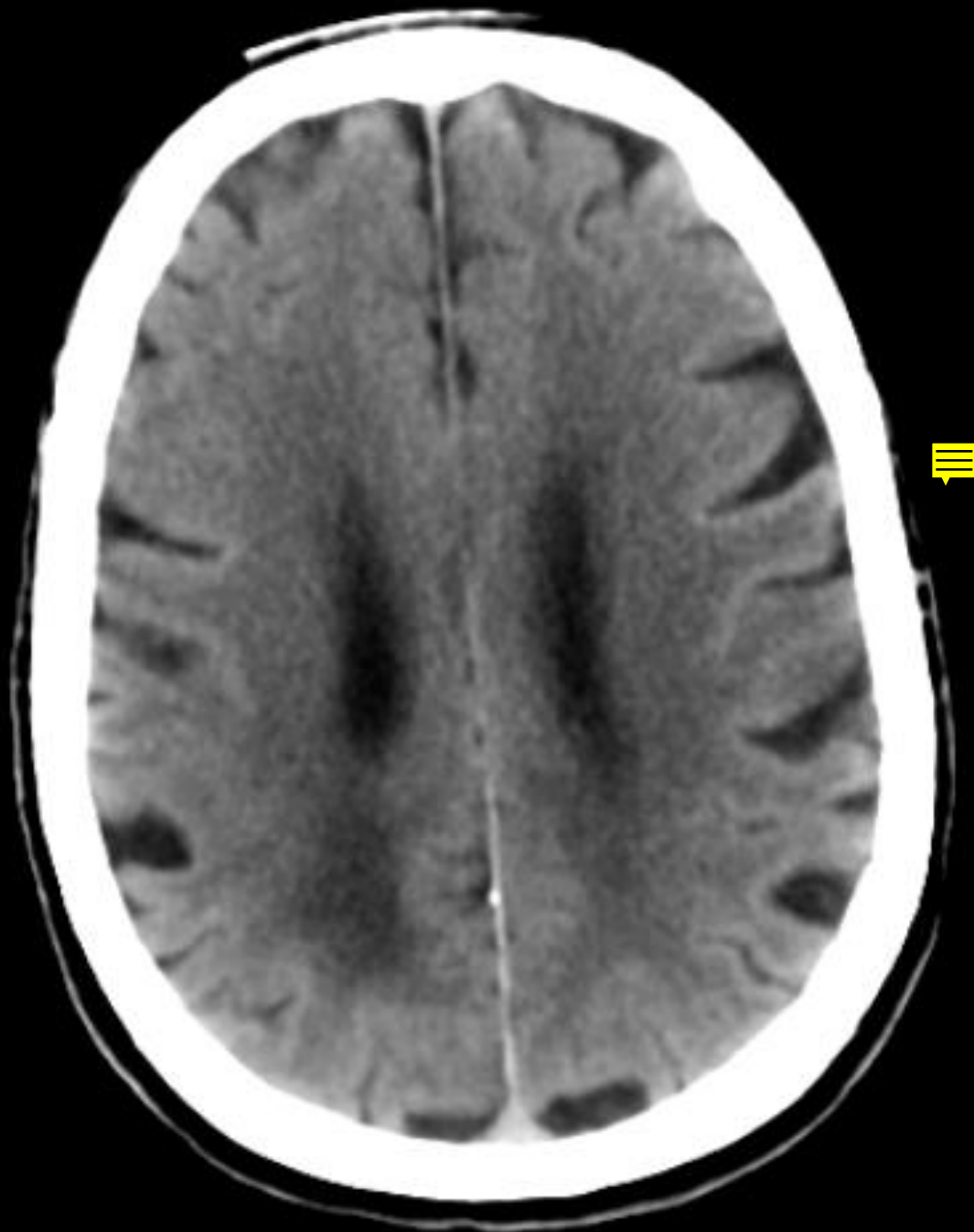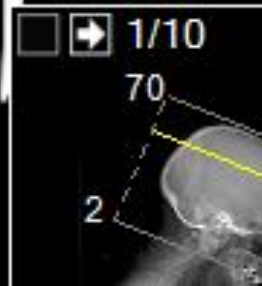

14

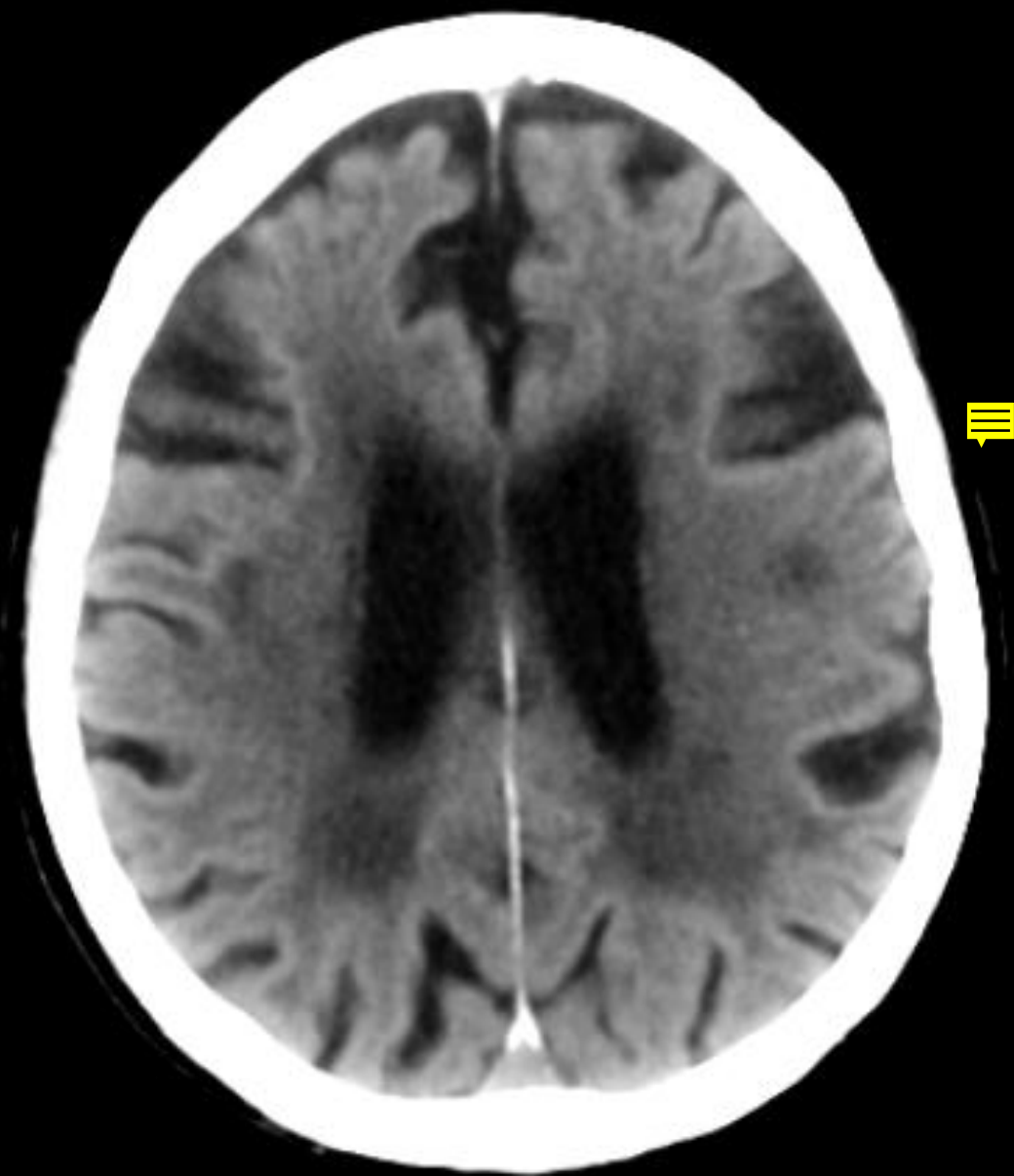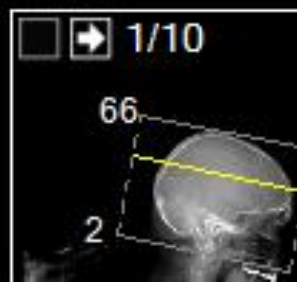

15

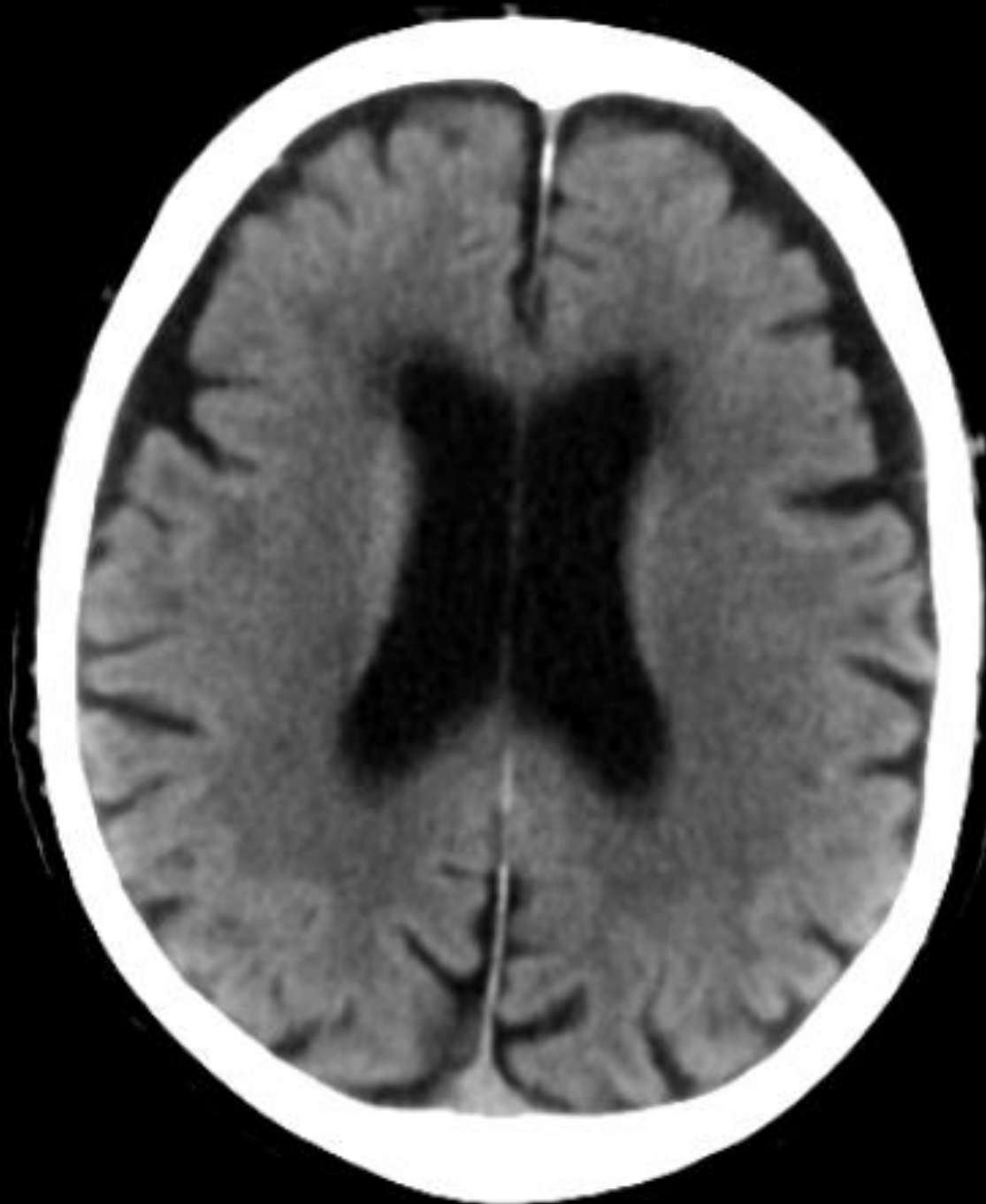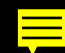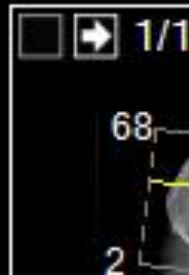

16

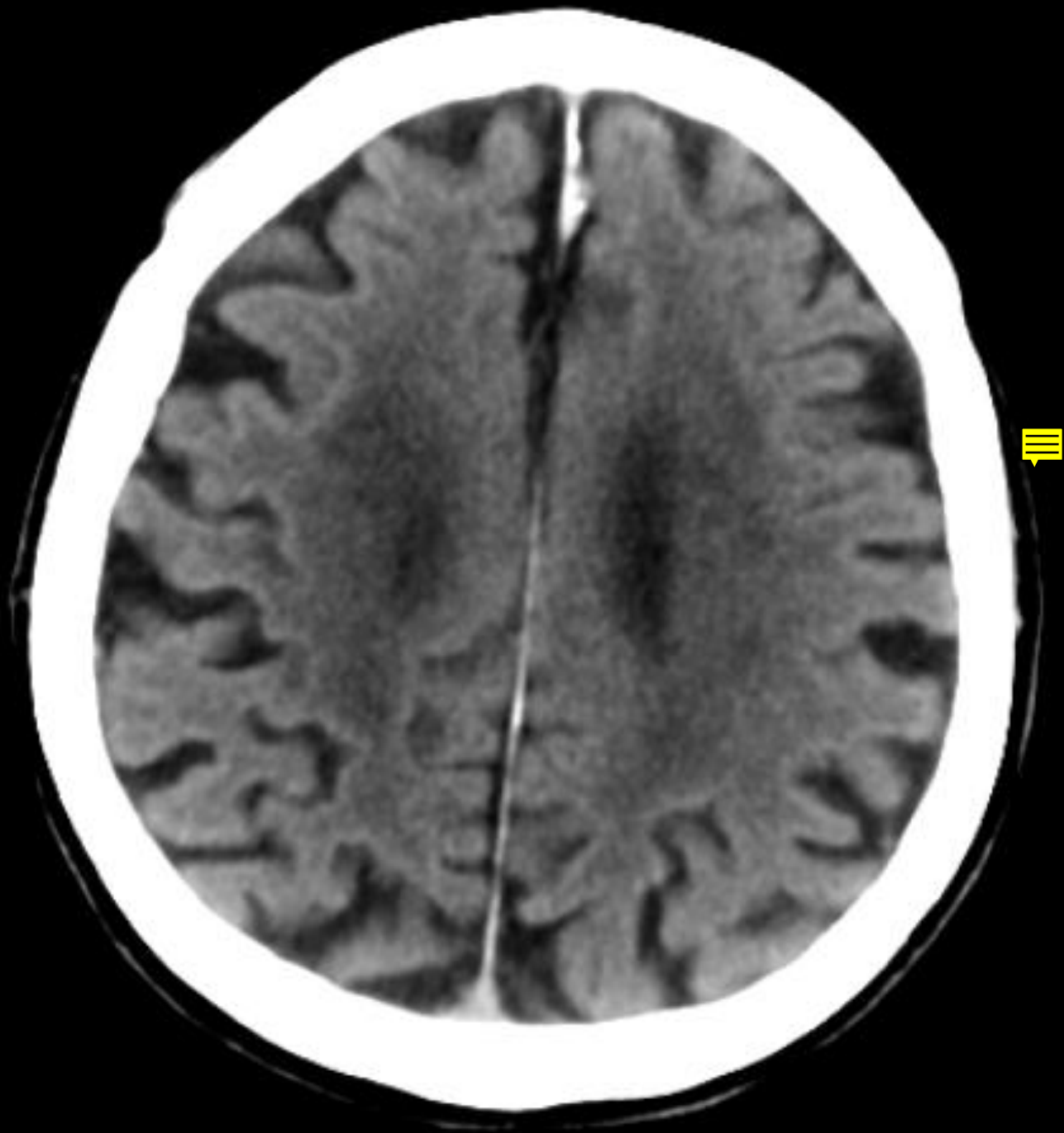

17

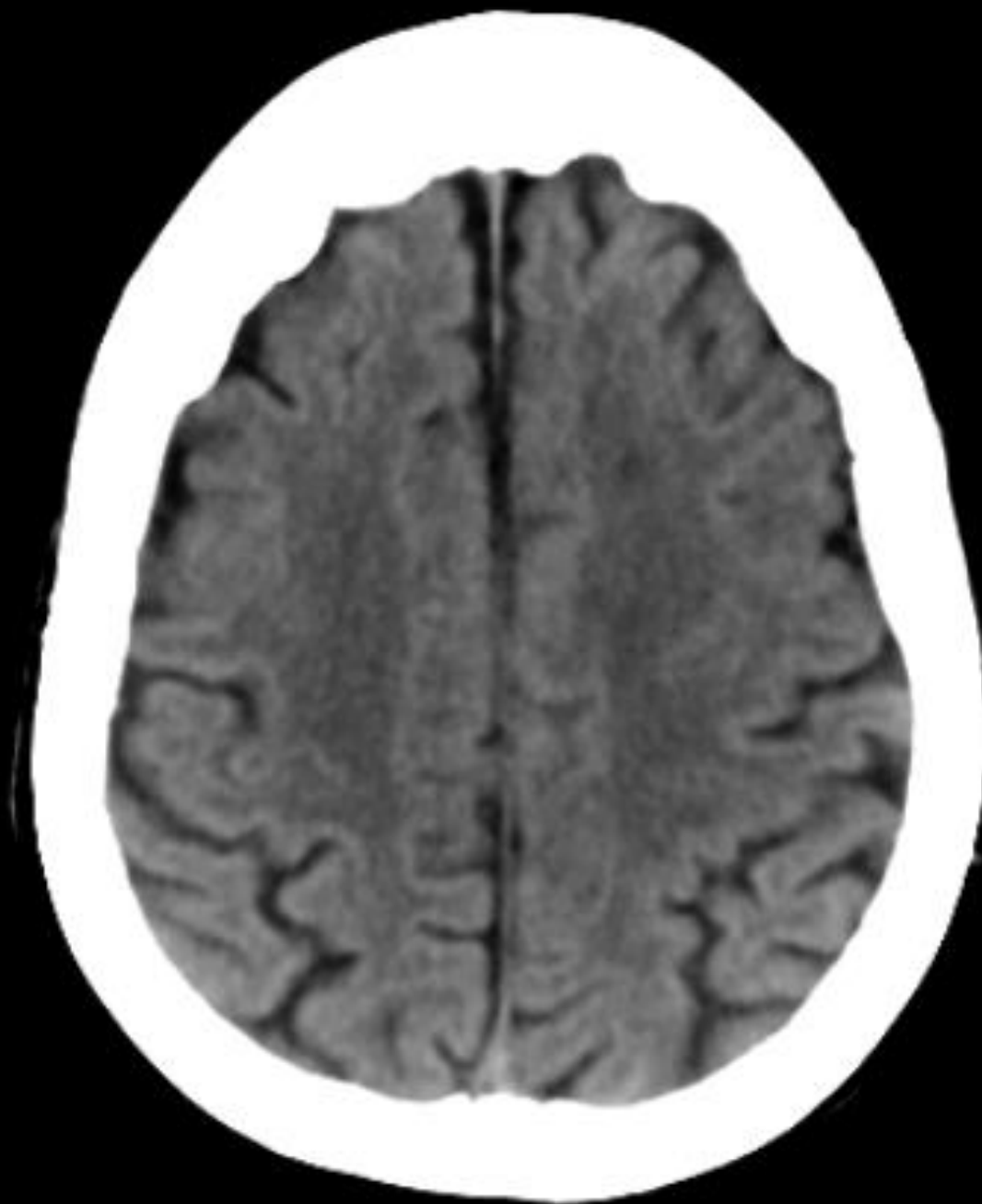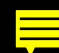

18

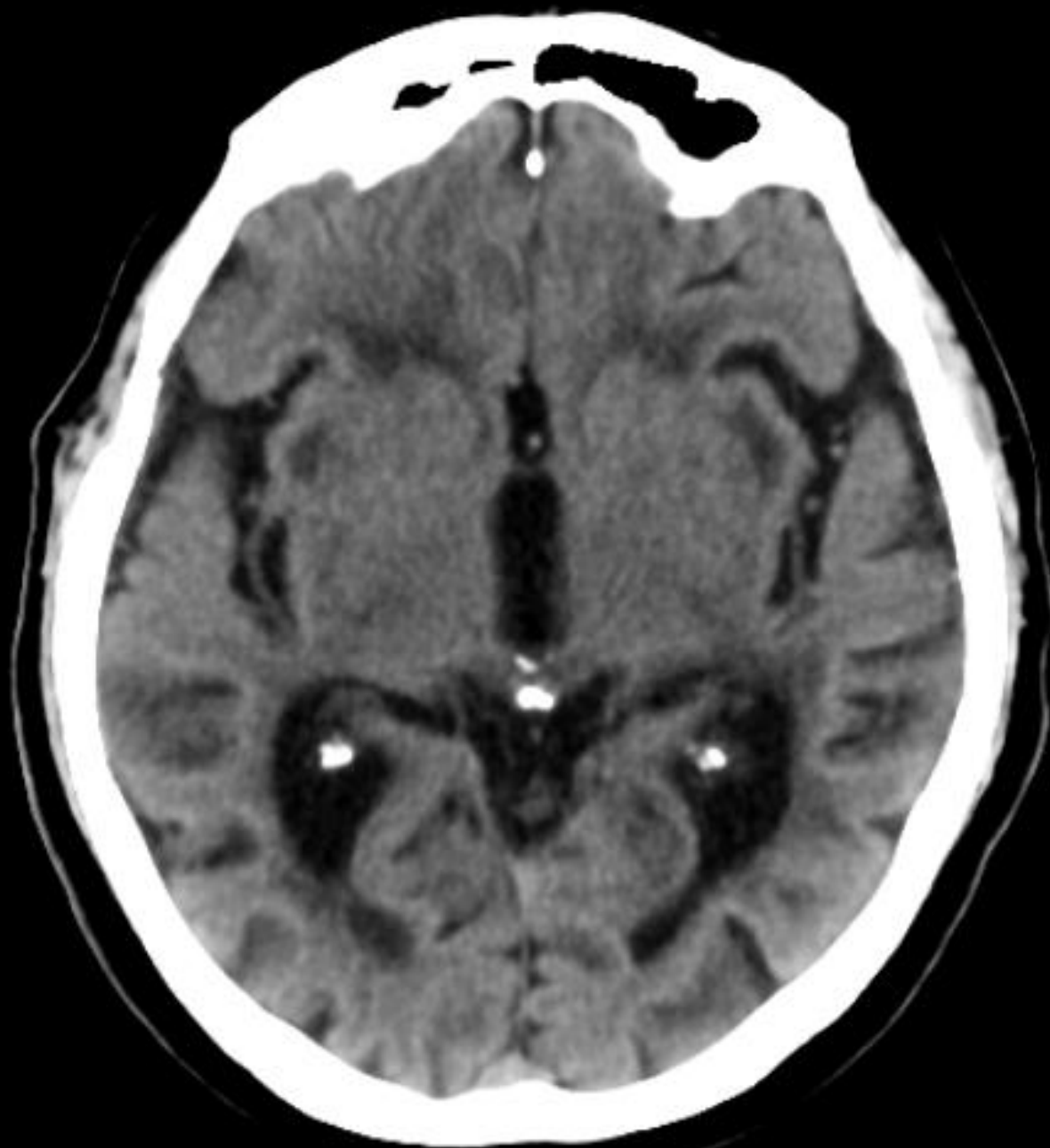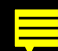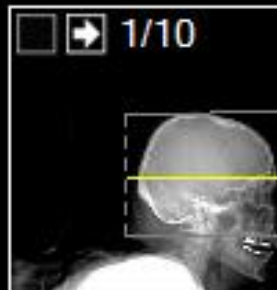

19

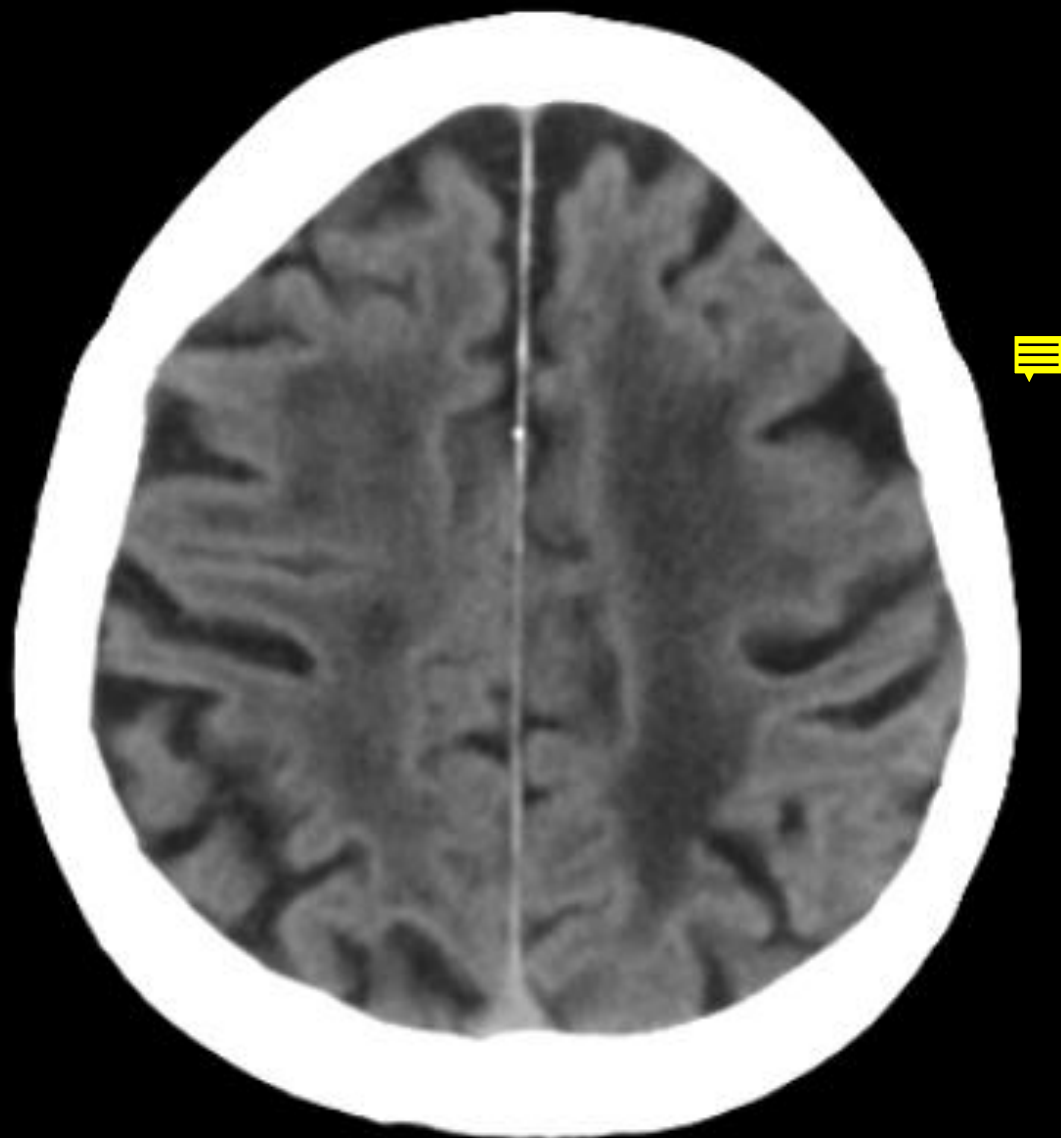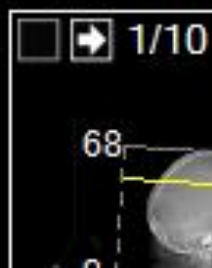

20

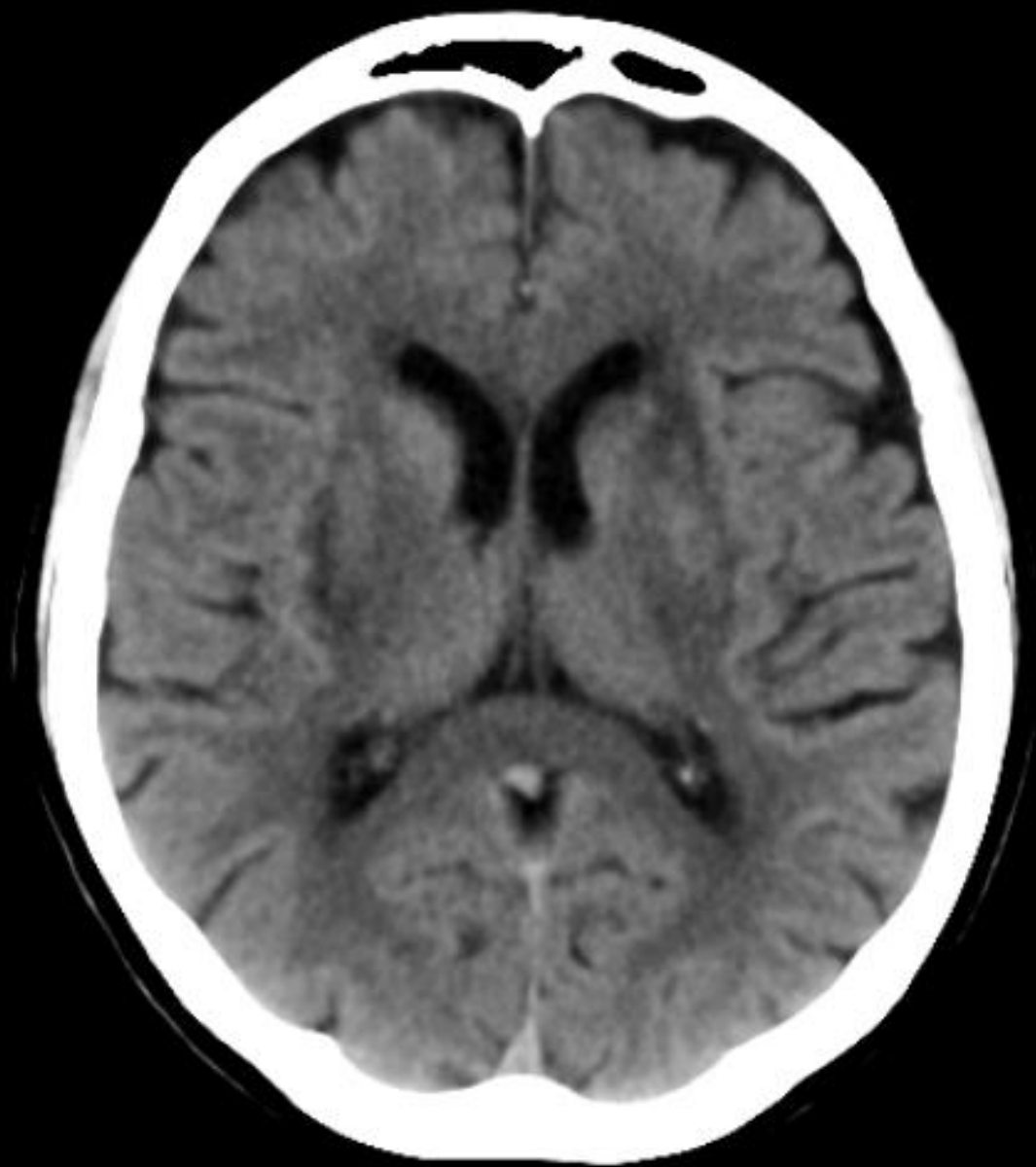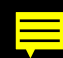

1/10

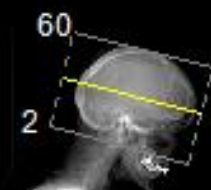

F

Supplement: Supplementary file 8 — (PDF 709 kb) [file 13244_2016_521_MOESM8_ESM.pdf]
